# ERK1/2 Signaling Plays an Important Role in Topoisomerase II Poison-Induced G2/M Checkpoint Activation

**DOI:** 10.1371/journal.pone.0050281

**Published:** 2012-11-16

**Authors:** Ryan H. Kolb, Patrick M. Greer, Phu T. Cao, Kenneth H. Cowan, Ying Yan

**Affiliations:** Eppley Institute for Research in Cancer and Allied Diseases, University of Nebraska Medical Center, Omaha, Nebraska, United States of America; University of South Alabama, United States of America

## Abstract

Topo II poisons, which target topoisomerase II (topo II) to generate enzyme mediated DNA damage, have been commonly used for anti-cancer treatment. While clinical evidence demonstrate a capability of topo II poisons in inducing apoptosis in cancer cells, accumulating evidence also show that topo II poison treatment frequently results in cell cycle arrest in cancer cells, which was associated with subsequent resistance to these treatments. Results in this report indicate that treatment of MCF-7 and T47D breast cancer cells with topo II poisons resulted in an increased phosphorylation of extracellular signal-regulated kinase 1 and 2 (ERK1/2) and an subsequent induction of G2/M cell cycle arrest. Furthermore, inhibition of ERK1/2 activation using specific inhibitors markedly attenuated the topo II poison-induced G2/M arrest and diminished the topo II poison-induced activation of ATR and Chk1 kinases. Moreover, decreased expression of ATR by specific shRNA diminished topo II poison-induced G2/M arrest but had no effect on topo II poison-induced ERK1/2 activation. In contrast, inhibition of ERK1/2 signaling had little, if any, effect on topo II poison-induced ATM activation. In addition, ATM inhibition by either incubation of cells with ATM specific inhibitor or transfection of cells with ATM specific siRNA did not block topo II poison-induced G2/M arrest. Ultimately, inhibition of ERK1/2 signaling greatly enhanced topo II poison-induced apoptosis. These results implicate a critical role for ERK1/2 signaling in the activation of G2/M checkpoint response following topo II poison treatment, which protects cells from topo II poison-induced apoptosis.

## Introduction

Topo II is a nuclear enzyme that has an important role in topological rearrangement of DNA during replication, transcription and resolution/separation of daughter chromosomes at mitosis [1]. Drugs that target topo II can be divided into two broad groups; topo II poisons that target DNA-topo II complexes and topo II inhibitors that directly inhibit the topo II catalytic activity [2]. Topo II poisons, such as the doxorubicin (DOX) and etoposide (ETOP), stabilize the covalent DNA-topo II intermediate by stimulating the cleavage reaction and/or inhibiting the religation step, which results in the accumulation of double-stranded DNA breaks [1]. In the past decade, topo II poisons have been commonly used in the treatment of numerous types of cancers, including blood, breast, ovarian and lung cancers. While therapy with topo II poisons can improve survival rates of cancer patients, their efficacy is largely limited by the rapid development of drug resistance to these agents. Evidence has shown that treatment of cancer cells with topo II poisons can result in apoptosis induction and/or cell cycle arrest [3], [4], [5]. The induction of cell cycle arrest, providing time for repairing the damaged DNA, has been shown to be associated with the resistance of cancer cells to topo II poison treatment [6], [7], [8], [9]. Thus, understanding the mechanism involved in the activation of cell cycle checkpoint following topo II poison treatment is necessary in order to improve the effectiveness of these anticancer agents.

Due to frequent mutation or alteration in genes involved in G1 checkpoint control, most cancer cells are defective in G1 checkpoint regulation and thus dependent on the intra-S and G2 checkpoints in response to DNA damage [10]. Because activation of the intra-S checkpoint results in slowing rather than complete arrest of the cell cycle, cancer cells bearing DNA damage may progress through the S-phase checkpoint and halt only at the G2 checkpoint [10]. Consistent with these findings, recent studies show that the cell cycle arrest observed in cancer cells treated with topo II poisons is primarily the G2/M arrest [11].

The G2 checkpoint is controlled by the Cdc2/Cyclin B complex, whose activity is required for G2/M transition of the cell cycle [12]. Previous studies have shown that Cdc2-Tyr15 phosphorylation is induced and maintained during radiation-induced G2/M arrest and that introduction of Cdc2-Y15F mutant, which cannot undergo phosphorylation at this site, abolished DNA damage-induced G2/M arrest [13], [14], [15]. Cdc2-Tyr15 is phosphorylated by Wee1 kinase, which phosphorylates Cdc2 at Tyr15, and Myt1 kinase which phosphorylates Cdc2 at Thr14 and, to a lesser extent, at Tyr15 [16], [17]. During normal cell cycle progression, Cdc2 is activated by dephosphorylation of Tyr15 residue by Cdc25 phosphatases [18]. In response to DNA damage, phosphorylation of Cdc25 phosphatase by Chk1 and Chk2 enhances binding of Cdc25 to SCF^βTrCP^ and subsequent proteolysis of Cdc25. In addition, phosphorylation of Cdc25A-Thr506 and Cdc25C-Ser216 following DNA damage enhances the binding of Cdc25A/C to 14-3-3, sequestering them from their substrates [19], [20], [21].

Upon cells transition from G2 to mitotic phase, histone H3 is phosphorylated at Ser10, which is associated with chromosome condensation prior to cell division [22]. Since both G2 and mitotic cells have *4N*-DNA content and are not distinguishable from each other by propidium iodide staining, phosphorylation of H3-Ser10 in *4N*-DNA content cells has been commonly used as a specific marker for mitotic cells [23], [24]. Furthermore, previous studies indicate that the initial phosphorylation of H3-Ser10 occurs in the late G2 phase but only on the pericentromeric chromatin. As cells progress through mitosis, the phosphorylation spreads along chromosomes and is completed at the end of prophase [25], [26]. Thus, there is a gradual increase in H3-Ser10 phosphorylation from the beginning of mitosis to the end of mitosis. In log phase growing cells, phosphorylation of H3-Ser10 in mitotic cells is detected in a wide range by flow cytometry analysis [27], [28]. In response to the activation of G2/M cell cycle checkpoint following DNA damage, the phosphorylation of H3-Ser10 is suppressed due to the blockage of the G2/M transition of the cell cycle [12], [27], [28].

Previous studies have shown that ATM and ATR are both phosphorylated and activated in response to double strand DNA breaks and play important roles in the activation of G2/M checkpoint response [29]. ATR activation results in the activation of Chk1 through phosphorylation at Ser317 and Ser345 [30], [31], while ATM activation induces Chk2 activity through phosphorylation at Thr68 [32], [33].

Involvement of p53 in the regulation of G2/M checkpoint has also been reported in several studies. A target gene of p53, 14-3-3σ, has been found be up-regulated following DNA damage, which prevents proper nuclear localization of Cdc2/Cyclin B after DNA damage [34]. Furthermore, another p53 target gene GADD45 has been shown to interact with Cdc2 and inhibits its activity [35]. Moreover, although p53 induced p21^Waf1/Cip1^ is a poor inhibitor of Cdc2 *in vitro* compared to other cyclin-dependent kinases [36], some evidence suggest p21^Waf1/Cip1^ may prevent interaction between Cdc2 and its activator Cdc25C phosphatase [37] as well as the Cdc2 activation by CAK kinase [38]. Finally, a recent report shows that, in H1299 non-small cell lung carcinoma cells, ectopic p53 expression promotes G2/M cell cycle arrest and which, in turn, suppresses DOX-induced mitotic cell death [9].

Mitogen-activated protein kinases (MAPKs) have been reported to play important roles in the cellular response to DNA damage [39], [40], [41], [42], [43]. A previous study has indicated that p38 mediated activation of Mitogen-activated protein kinase-activated protein kinase 2 (MK2) is necessary for DOX-induced G2/M arrest in U2OS human osteosarcoma cells [43], while others have demonstrated that ERK1/2 activation following topo II poison treatment is associated with G2/M cell cycle arrest [44]. In the present study, we examined the mechanisms involved in the regulation of the G2/M checkpoint response by ERK1/2 signaling following treatment of cells with topo II poisons. Results in this report indicate that ERK1/2 activation plays a critical role in the activation of G2/M checkpoint following topo II poison treatment. By abrogation of the G2/M checkpoint response and increase of apoptosis induction, inhibitors of ERK1/2 signaling may potentially be used to sensitize cancer cells to topo II poisons.

## Materials and Methods

### Cell culture and drug treatment

MCF-7 and T47D human breast cancer cells were obtained from ATCC (Manassas, VA) and maintained in Dulbecco's Modified Eagle's Medium (DMEM) containing 10% fetal bovine serum (FBS). For drug treatment, doxorubicin (DOX) (Invitrogen, Carlsbad, CA) was dissolved in water and etoposide (ETOP) (Calbiochem, San Diego, CA) dissolved in dimethyl sulfoxide (DMSO). The drugs were added to the cells at the indicated doses for 2 hr, washed with DMEM and then incubated in growth medium for the specified length of time. For studies involving the use of the MEK1/2 specific inhibitor U0126 [45] (LC Laboratories, Woburn MA), log-phase cells were incubated in medium containing the inhibitor, which was dissolved in DMSO. Control cells were incubated in medium containing the same amounts of vehicle. For experiments involving treatment with both MEK1/2 inhibitor and topo-II poison, cells were pre-incubated with MEK1/2 inhibitor for 1 hr prior to treatment with topo II poison. For experiments involving compounds dissolved in DMSO, the final concentration of DMSO in the medium was less than 0.1%.

### Short hairpin RNA and retroviral vectors

Retroviral vectors expressing short hairpin RNAs (shRNA) were obtained from Open Biosystems (Huntsville, AL). The sequence for shRNA targeting ATR is 5′-TGCT GTTG ACAG TGAG CGCC CAGA CCAG ATCA TTCA TTAT TAGT GAAG CCAC AGAT GTAA TAAT GAAT GATC TGGT CTGG TTGC CTAC TGCC TCGG A-3′ and 5′-TGCT GTTG ACAG TGAG CGCG CCGC TAAT CTTC TAAC ATTA TAGT GAAG CCAC AGAT GTAT AATG TTAG AAGA TTAG CGGC ATGC CTAC TGCC TCGG A-3′. The sequence for control shRNA targeting firefly luciferase is 5′-CCCG CCTG AAGT CTCT GATT AA-3′.

Retroviral vector expressing human papilloma virus type 16 viral protein E6 (HPV-E6) and relative control empty vector was obtained from Dr. Denise Galloway (Fred Hutchinson Cancer Research Center) [46].

Production of recombinant retroviral vectors and infection of MCF-7 cells was performed as described previously [47]. Briefly, Phoenix A retroviral packaging cells were transfected with retroviral vectors using MBS mammalian transfection kit (Strategene, La Jolla, CA) according to the manufacturer's direction. Medium containing amphotropic retrovirus was collected at 48 hr post transfection and filtered through a 0.4 µm filter. MCF-7 cells were infected with retroviral vectors in the presence of 4 µg/ml polybrene (Sigma-Aldrich, St Louis, MO). Clones stably expressing shRNA were selected in medium containing 2 µg/ml puromycin (Sigma-Aldrich, St Louis MO). Clones stably expressing HPV-E6 were selected in medium containing 800 µg/ml G418 (Invitrogen, Carlsbad, CA).

### Short interfering RNAs and transfection

Short interfering RNA duplexes (siRNA) were obtained from Dharmacon Research (Chicago, IL). Non-targeting control siRNA contains at least four mismatches to any human, mouse or rat gene, as previously determined by the manufacturer. The sequence for control siRNA is 5′-UAAGGCUAUGAAGAGAUAC-3′. SMARTpool siRNAs targeting ERK1/2 consists of eight siRNAs targeting multiple sites on ERK1/2 (ERK1/2-siRNAs). The sequences for siRNA targeting ERK1 are 5′-PUAAAGGUUAACAUCCGGUCUU-3′, 5′-PAACUUGUACAGGUCAGUCUUU-3′, 5′-PAGAGACUGUAGGUAGUUUCUU-3′, and 5′-PUACUGCAACUGCGUGUAGCUU-3′. The sequences for siRNA targeting ERK2 are 5′-PAAUAAGUCCAGAGCUUUGGUU-3′, 5′-PAGCUUGUAAAGAUCUGUUUUU-3′, 5′-PUUCUACUUCAAUCCUCUUGUU-3′ and 5′-PAAUUUCUGGAGCCCUGUACUU-3′. SMARTpool siRNAs targeting ATM consists of four siRNAs targeting multiple sites on ATM (ATM-siRNA). The sequences for ATM-siRNA are 5′-PAAUUCAGAAAGCAACAUUCUU-3′, 5′-PUUAUUUGGGAAUUCUGUCUUU-3′, 5′-PCUAACAAACAGGUGAUAUAUAUU-3′ and 5′-PAAAGGCCCAAGCUCCUCCUUU-3′.

Cells were transfected with 100 nM siRNA using DharmaFECT1 siRNA transfection reagent (Dharmacon Research, Chicago, IL) according to the manufacturer's direction. Transfected cells were incubated at 37°C for the additional indicated times, and analyzed for levels of the targeted protein by immunoblotting with specific antibodies. For experiments involving both siRNA transfection and topo II poison treatment, transfected cells were first incubated for the indicated times and then treated with topo II poison at the specified concentration. Treated cells were incubated at 37°C for the additional indicated times and analyzed for protein expression by immunoblotting and DNA content by fluorescence-activated cell sorting (FACS).

### Plasmids constructs and transfection

pCMV5 vector expressing p53-R175H dominant-negative mutant was kindly provided by Dr. Arnold J. Levine (Institute for Advanced Study, Princeton, NJ), which contains an arginine to histidine substitution at residue 175 of p53 polypeptide [48], [49]. MCF-7 cells were transiently transfected with the p53-R175H expressing vector or control empty vector using lipofectamine transfection reagent (Invitrogen, Carlsbad, CA) as described previously [50]. Following transfection, the cells were incubated for 48 hr prior to treatment with DOX.

### Antibodies and recombinant proteins

All antibodies were obtained from Santa Cruz Biotechnology (Santa Cruz, CA) unless indicated elsewhere. These include mouse IgG for Cdc2 (17), Chk1 (G4), Chk2 (B4), p38 (A12), p53 (D01) and phospho-ERK1/2 (E4); goat IgG for ATR (N-19), Actin (I-19), phospho-Cdc2 (Tyr-15), ERK1/2 (C14-G) and MAPKAPK2 (C18). Rabbit IgG for ATM (Ab-3) and PARP (Ab-2) were obtained from Calbiochem (San Diego, CA). Mouse IgG for Caspase 8 (1C12), Rabbit IgG for phospho-p38 and phospho-MAPKAPK2 was obtained from Cell Signaling Technology (Danvers, MA).

Recombinant p53 protein used in ATM and ATR kinase assays was a glutathione *S*-transferase fusion protein containing full-length human p53 (Addgene, Cambridge, MA). The Cdc25C recombinant protein used in Chk1 and Chk2 kinase assays was a glutathione *S*–transferase fusion protein containing residues 200–256 of human Cdc25C [kindly provided by Dr. Helen Piwnica-Worms (Washington University School of Medicine)]. Glutathione *S*-transferase recombinant protein was used as a control substrate in all kinase assays and was prepared according to the standard procedure (GE Healthcare Bio-Sciences, Pittsburgh, PA).

### Immunoblotting, immunoprecipitation and kinase assay

Immunoblotting and Immunoprecipitation were performed as described previously [28], [51]. ATM and ATR kinase activity was assayed as described previously using p53 recombinant protein as substrate [52], [53], [54], [55]. Briefly, ATM and ATR proteins were immunoprecipitated from 500 µg cell lysate using anti-ATM (AB-3) and anti-ATR (N-19) antibody respectively. The immune complexes were isolated with protein-A (for ATM) or protein-G (for ATR) agarose, washed three times with wash buffer (20 mM HEPES, pH 7.4, 50 mM NaCl, 2.5 mM MgCl_2_, 0.1 mM EDTA, 0.1% Triton X-100, and 0.1 mM sodium orthovanadate), and twice with kinase buffer (20 mM HEPES, pH 7.4; 10 mM MgCl_2_; 1 mM EDTA, 1 mM EGTA, 20 mM *p*-nitrophenyl phosphate, 20 mM β-glycerophosphate, 0.1 mM sodium orthovanadate, and 1 mM DTT). Resuspended immune complexes were incubated in kinase buffer with 5 μg of p53 recombinant protein, 40 μM ATP, and 5 μCi of [γ-^32^P]ATP (6000 Ci/mmol) for 50 min at 30°C. Chk1 and Chk2 kinase activity was assayed as described previously using Cdc25C recombinant protein as substrate [32], [56], [57], [58], [59]. Briefly, Chk1 and Chk2 were immunoprecipitated from 250 µg of cell extract, using anti-Chk1 (G4) and anti-Chk2 (B-4) antibody respectively. The immune complexes were isolated with protein-A (for Chk1) or protein-G (for Chk2) agarose, washed and assayed for kinase activity using 5 μg Cdc25C recombinant protein as substrate. The incubation time for Chk1 and Chk2 kinase reaction was 20 min at 30°C. The kinase reactions were stopped by adding 20 μl of 5× Laemmli SDS sample buffer and boiling for 3 min [60]. Substrate phosphorylation by kinase was analyzed by SDS-polyacrylamide gel electrophoresis and autoradiography.

### Cell cycle analysis

Cell cycle analysis was carried out by fluorescence-activated cell sorting (FACS) using a FACSCalibur instrument (Beckon Dickinson) as described in our previous study [51]. Each analysis was performed using 20,000 cells.

### Analysis for mitotic cells

Cells were treated with 1 μM DOX in the presence or absence of 50 μM U0126 for 2 hr and washed. The cells were incubated for an additional 22 hr in growth medium containing 100 ng/ml nocodazole (Acros Organics, Geel, Belgium) [61], with or without the presence of U0126. Cells were then fixed and analyzed by FACS for mitotic cells, which contain both *4N* DNA content and phospho-Histone H3-Ser10, as described previously [28].

### DAPI staining

Cell death was assessed by 6′, 6-diamidino-2-phenylindole (DAPI) staining as described previously [62]. Briefly, cells (50,000) re-suspended in 100 µl of 30% FBS in PBS were added to a cytocentrifuge and spun at 1000 rpm for 5 min. The resulting slides were air dried, washed two times with PBS and then fixed for 1 hr in 4% paraformaldehyde at room temperature. Following fixation, cells were washed with PBS then stained with DAPI (2.5 µg/ml in PBS) for 1 hr in dark at room temperature. Stained cells were washed with PBS and analyzed by fluorescent microscopy. Apoptotic cells were identified by condensation and fragmentation of nuclei [63]. The percentage of apoptotic cells was calculated as the ratio of apoptotic cells to total cells counted. At least 800 cells were counted per sample.

## Results

### Topo II poison induces G2/M cell cycle arrest and ERK1/2 activation in MCF-7 human breast cancer cells

**Figure 1:**
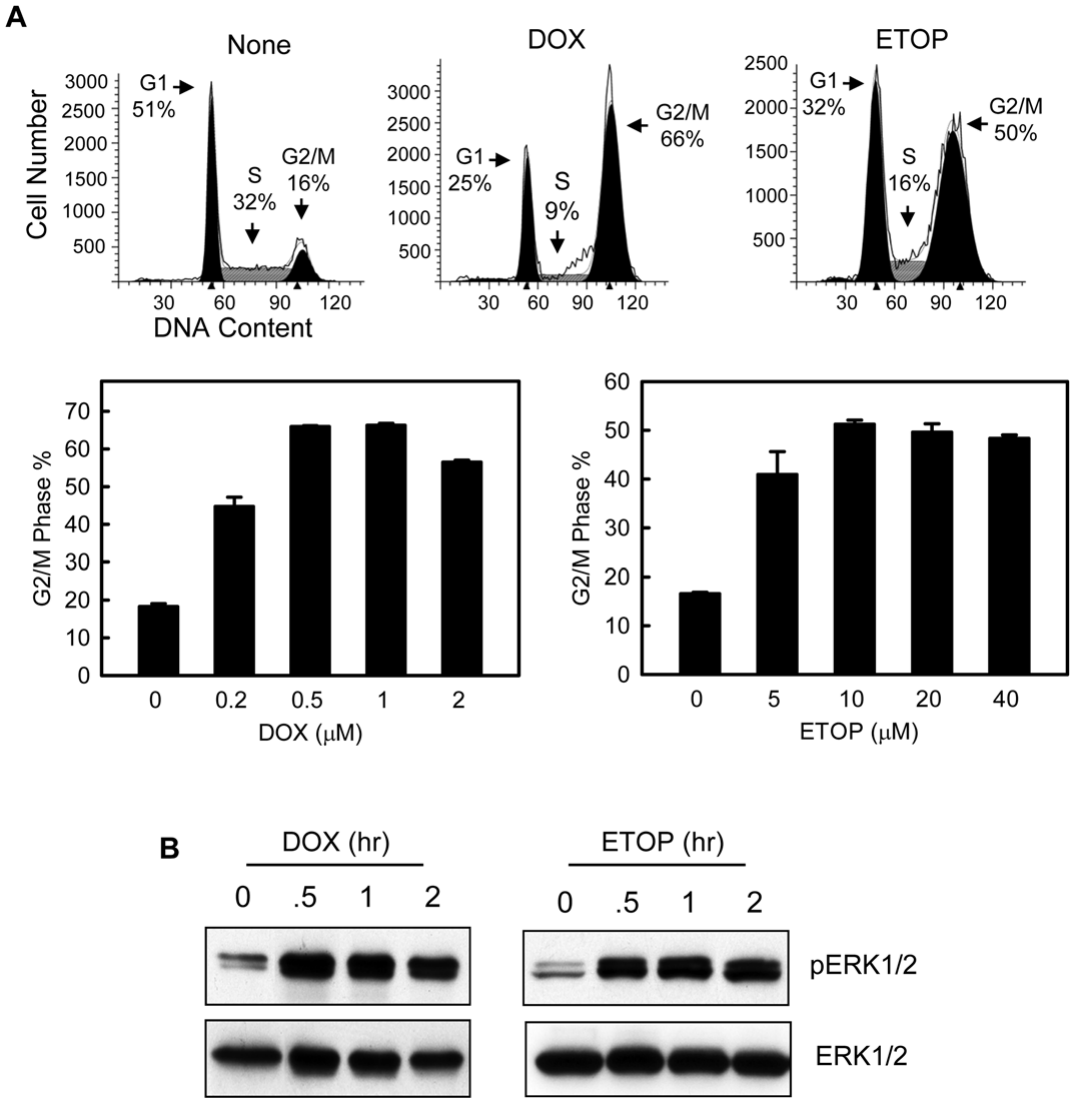
DOX and ETOP induce G2/M arrest and ERK1/2 activation in MCF-7 breast cancer cells. (A) Log-phase MCF-7 cells were treated with DOX or ETOP at the indicated doses as described in *Materials and Methods* and incubated for 24 hr. The cells were analyzed for DNA content by FACS. Upper panel: histograms shown are cells treated with none, 1 µM DOX or 10 µM ETOP. Cell cycle phases are indicated. Lower panel: Graphs depict the percentage of cells with 4*N*-DNA content, indicative of G2/M phase of the cell cycle, and represent the mean ± s.d. of two sets of experiment with duplicate samples. (B) MCF-7 cells were incubated in the presence of 0.5 µM DOX or 10 µM ETOP for the hours indicated and analyzed for phospho-ERK1/2 and total-ERK1/2 by immunoblotting.

To study the effect of topo II poison on cell cycle checkpoint response, MCF-7 cells were treated with increasing doses of DOX or ETOP and analyzed for DNA content after incubation for 24 hr following treatment. As shown in Figure 1A, both DOX and ETOP resulted in a dose-dependent increase in the percentage of cells with *4N*-DNA content, indicative of G2/M cell cycle arrest [12]. Relative to the control untreated cells, there was a maximum 4-fold increase in cells with *4N*-DNA content following treatment of MCF-7 cells with 0.5 µM DOX and a 3-fold increase following treatment with 10 µM ETOP.

We next assessed the changes in ERK1/2 phosphorylation in cells treated with DOX or ETOP. As shown in Figure 1B, treatment with DOX or ETOP resulted in a rapid increase in ERK1/2 phosphorylation in MCF-7 cells. Furthermore, as shown in supplemental Figure S1, topo II poison-induced ERK1/2 phosphorylation was observed within 15 min following incubation with drug and remained detectable for at least 24 hr after drug treatment.

**Figure 2:**
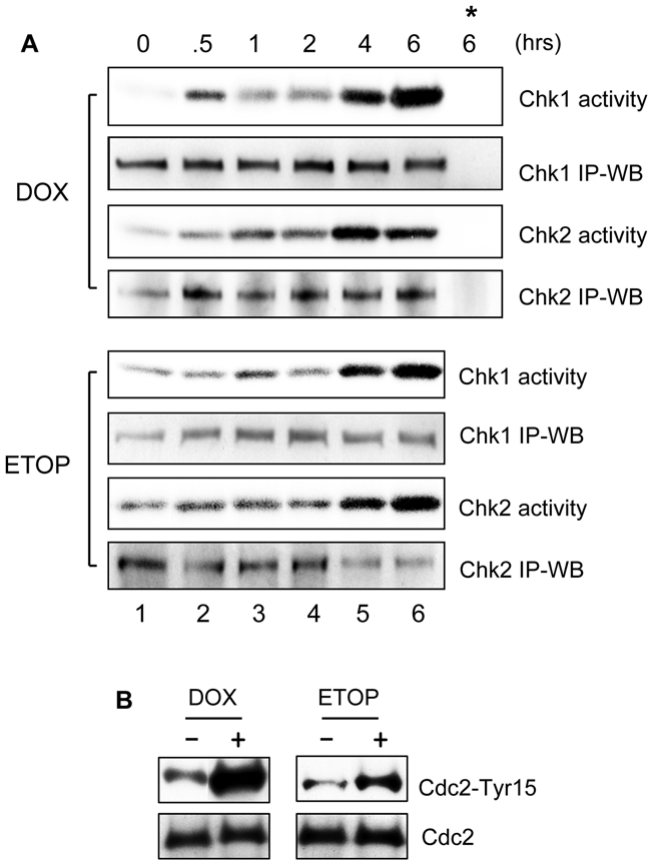
DOX and ETOP induce activation of Chk1 and Chk2 kinases and inhibition of Cdc2 kinase. (A) MCF-7 Cells were incubated with 1 µM DOX (upper panel) or 10 µM ETOP (lower panel) for the indicated times for up to 2 hr (lanes 1–4). For the 4 hr and 6 hr time points (lanes 5–6), the cells were incubated for 2 hr with DOX or ETOP, washed with DMEM and incubated for additional 2 hr and 4 hr, respectively, in regular culture medium. Following treatment, Chk1 and Chk2 kinases were respectively immunoprecipitated from cell lysates and examined for kinase activity as described in *Materials and Methods* (*Chk1 Activity* and *Chk2 Activity*). Levels of Chk1 and Chk2 in the immunoprecipitates were determined by immunoblotting (*Chk1 IP-WB* and *Chk2 IP-WB*). *, as a negative control, kinase assay was carried out using immunoprecipitates obtained by incubating DOX-treated cell sample (6 hr time point) with non-immunized IgG. (B) Cells were treated as described above and incubated for 2 hr. Cdc2 was immunoprecipitated from cell lysate and analyzed for levels of Cdc2-Tyr15 phosphorylation by immunoblotting (*Cdc2-Tyr15*). As a control, Cdc2 in the immunoprecipitates was assessed by immunoblotting (*Cdc2*).

Previous studies have shown that the activation of Chk1 and Chk2 kinases following DNA damage plays an important role in the activation of G2/M checkpoint response [64]. We therefore examined the changes in Chk1 and Chk2 activities following treatment of MCF–7 cells with DOX and ETOP. As shown in Figure 2A, both Chk1 and Chk2 activities were markedly induced following treatment with DOX or ETOP. A maximum activation of Chk1 and Chk2 activity were detected at 6 hr following incubation with either drug (*Chk1 activity* and *Chk2 activity*, lane 6 *vs*. 1). Furthermore, the observed increase in kinase activity was not associated with changes in protein levels of Chk1 or Chk2 (Figure 2A, *Chk1 activity vs*. *Chk1 IP-WB, Chk2 activity vs.Chk2 IP-WB*).

It has been shown that activation of Chk1 and Chk2 results in inhibition of Cdc2/Cyclin B activity [13], [15], [18], we next examined changes in Cdc2-Tyr15 phosphorylation, which is indicative of Cdc2 kinase inhibition [15], following treatment with DOX or ETOP. As shown in Figure 2B, treatment with either DOX or ETOP did result in a large increase in Cdc2-Tyr15 phosphorylation (*Cdc2-Tyr15*), determined at 4 hr following drug treatment. Furthermore, the increase in Cdc2-Tyr15 phosphorylation was not associated with changes in Cdc2 protein levels (*Cdc2-Tyr15 vs*. *Cdc2*).

Collectively, results of these studies indicate that topo-II poison treatment of MCF-7 cells induced ERK1/2 activation, which was associated with an activation of Chk1 and Chk2 kinases and concomitant phosphorylation of Cdc2-Tyr15. The net effect of these changes resulted in decreased Cdc2 activity and G2/M arrest in MCF-7 cells.

### Inhibition ERK1/2 activation attenuates topo II poison-induced G2/M cell cycle arrest

**Figure 3:**
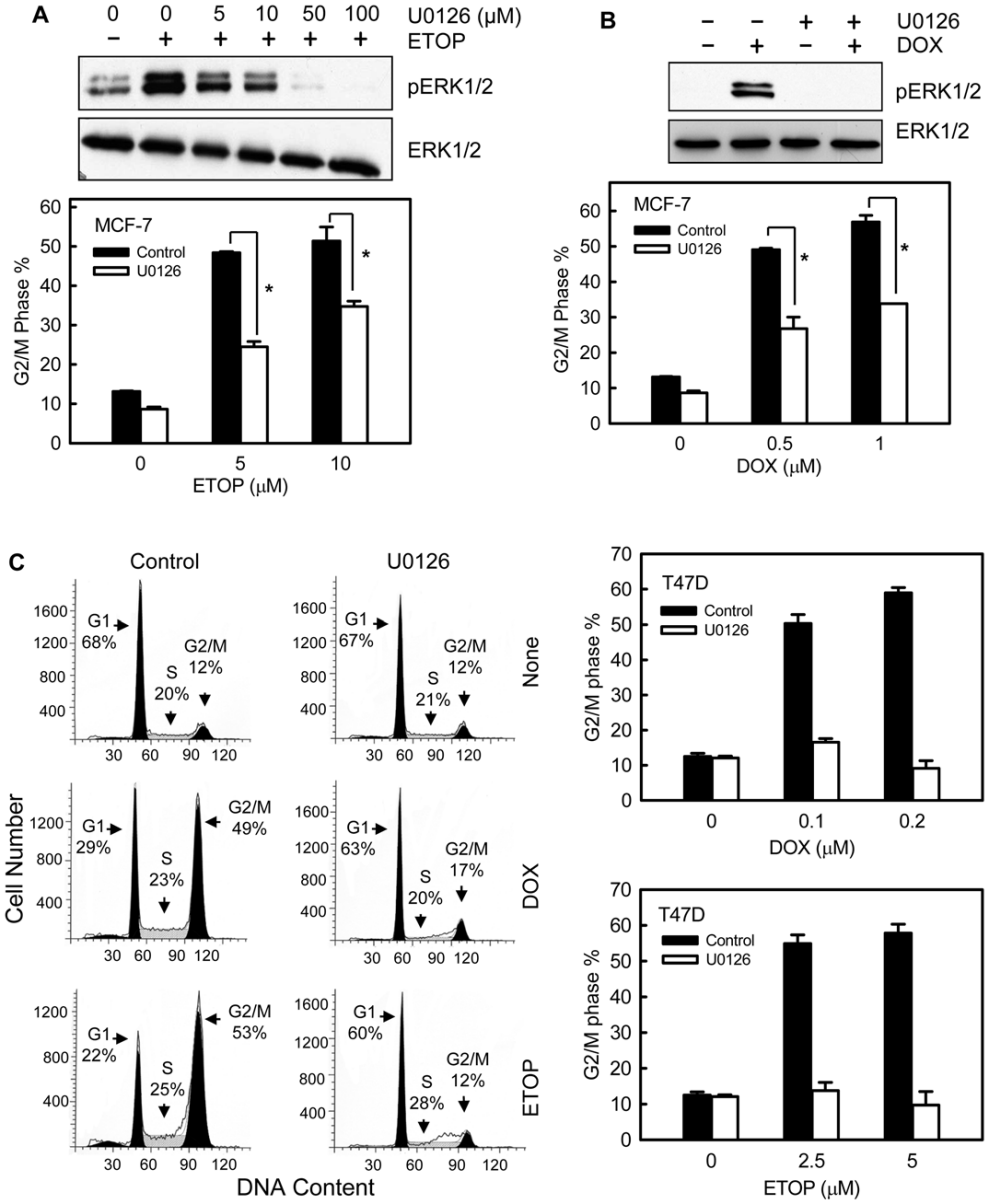
Inhibition of ERK1/2 attenuates topo II poison-induced G2/M arrest in MCF-7 cells. (A) Upper panel: MCF-7 cells were incubated with U0126 at the indicated doses for 1 hr and then treated with 10 µM ETOP for 2 hr with the presence of U0126. The cells were lysed and analyzed for phospho- and total-ERK1/2. Lower panel: in the presence or absence of 50 µM U0126, MCF-7 cells were treated with ETOP at the indicated doses for 2 hr. The cells were washed, incubated for additional 24 hr in the presence or absence of U0126, and analyzed for DNA content by FACS. Bar graphs depict the percentage of cells with *4N*-DNA content (G2/M phase cells) and represent the average of two independent experiments in duplicate. *p<0.001 (n = 4). (B) Upper panel: in the presence or absence of 50 µM U0126, cells were treated with/without 0.5 µM DOX for 2 hr and analyzed for phospho- and total-ERK1/2. Lower panel: MCF-7 cells were treated with DOX at the indicated doses for 2 hr, in the presence or absence of 50 µM U0126. The cells were washed, incubated for additional 24 hr with/without U0126 and analyzed for DNA content. Bar graphs depict the percentage of cells with *4N*-DNA content and represent the average of two independent experiments in duplicate. *p<0.001 (n = 4). (C) Left panel: T47D cells were treated with/without 0.1 µM DOX or 5 µM ETOP in the presence or absence of U0126 and incubated for 24 hr. Histograms shown are FACS analyses of the resulting cells. Cell cycle phases are indicated. Right panels: Cells were treated with DOX (upper panel) or ETOP (lower panel) at the indicated doses in the presence or absence of U0126. After 24 hr incubation, the cells were analyzed for DNA content. Bar graphs depict the percentage of G2/M phase cells and represent the average of two independent experiments in duplicate.

We next examined the effects of mitogen-activated protein kinase kinase 1 and 2 (MEK1/2) specific inhibitor U0126 on topo II-induced G2/M checkpoint. We have previously reported that incubation with 50 μM U0126 resulted in maximal inhibition of ERK1/2 phosphorylation in log-phase growing MCF-7 cells [51]. As shown in Figure 3A and 3B, incubation of MCF-7 cells with 50 μM U0126 completely blocked both ETOP- and DOX-induced ERK1/2 activation, and significantly attenuated the induction of G2/M cell cycle arrest following ETOP or DOX treatment. Incubation of MCF-7 cells with U0126 by itself had no effect on the cell cycle distribution compared to the untreated cells (Figure S2).

We also assessed the effect of ERK1/2 inhibition on DOX- and ETOP-induced G2/M arrest in T47D human breast cancer cells. As shown in Figure 3C, incubation of T47D cells with U0126 resulted in a complete attenuation of DOX- or ETOP-induced G2/M arrest.

**Figure 4:**
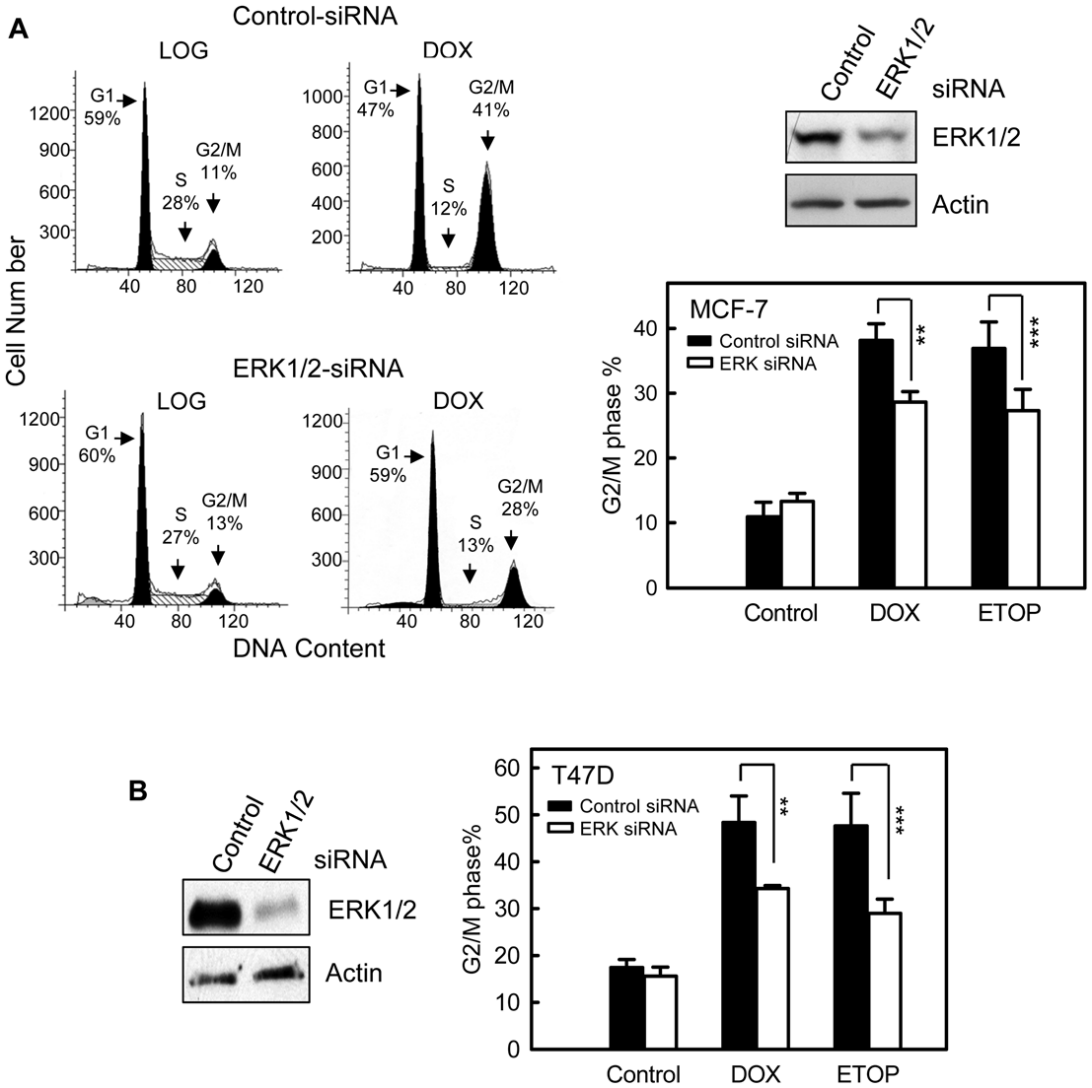
Inhibition of ERK1/2 by specific siRNA diminishes topo II poison-induced G2/M arrest. (A) MCF-7 cells were transfected with ERK1/2 specific siRNA or control non-targeting siRNA and incubated for 2 days. The cells were then treated with 0.5 µM DOX or 5 µM ETOP, incubated for 24 hr and analyzed for DNA content by FACS. Left panel: histograms shown are DNA content analyses for the indicated cell samples. Upper right panel: levels of ERK1/2 in siRNA-transfected cells were determined by Western blotting. Lower right panel: bar graph depicts the percentage of cells in G2/M phase and presented as mean ± s.d. of three independent experiments in duplicate. ** *p*<0.005 (n = 6), *** *p*<0.01 (n = 6), significant difference from cells transfected with control siRNA. (B) T47D cells were transfected with siRNA targeting ERK1/2 or control siRNA, incubated for 2 days and treated with 0.2 µM DOX or 5 µM ETOP. Left panel: levels of ERK1/2 in siRNA-transfected cells were determined by Western blotting. Right panel: the treated cell were incubated for additional 24 hr and analyzed for DNA content by FACS. Bar graph depicts the percentage of cells in G2/M phase and presented as mean ± s.d. of two independent experiments in duplicate. ** *p*<0.005 (n = 4), *** *p*<0.01 (n = 4), significant difference from cells transfected with control siRNA.

To confirm the effect of ERK1/2 inhibition on topo II poison-induced G2/M arrest, MCF-7 cells were transfected with ERK1/2 specific or control non-targeting siRNA and then treated with topo II poisons. As shown in Figure 4A (upper right panel), there was an approximate 50% reduction in ERK1/2 protein in MCF-7 cells transfected with ERK1/2 specific siRNA compared to control siRNA transfected cells. As shown in Figure 4A (left and lower right panels), transfection of MCF-7 cells with ERK1/2 specific siRNA significantly attenuated both DOX- and ETOP-induced G2/M arrest compared to control siRNA transfected cells. In contrast, transfection with control siRNA had no noticeable effect on ERK1/2 protein level as well as on DOX-induced G2/M arrest compared to untransfected cells (supplemental Figure S3). Consistent with the results obtained from MCF-7 cells, transfection of T47D cells with ERK1/2 specific siRNA, resulting in a near 80% reduction in ERK1/2 protein compared to control siRNA transfected cells (Figure 4B, left panel), also significantly attenuated DOX- or ETOP-induced G2/M arrest in T47D cells (Figure 4B, right panel).

**Figure 5:**
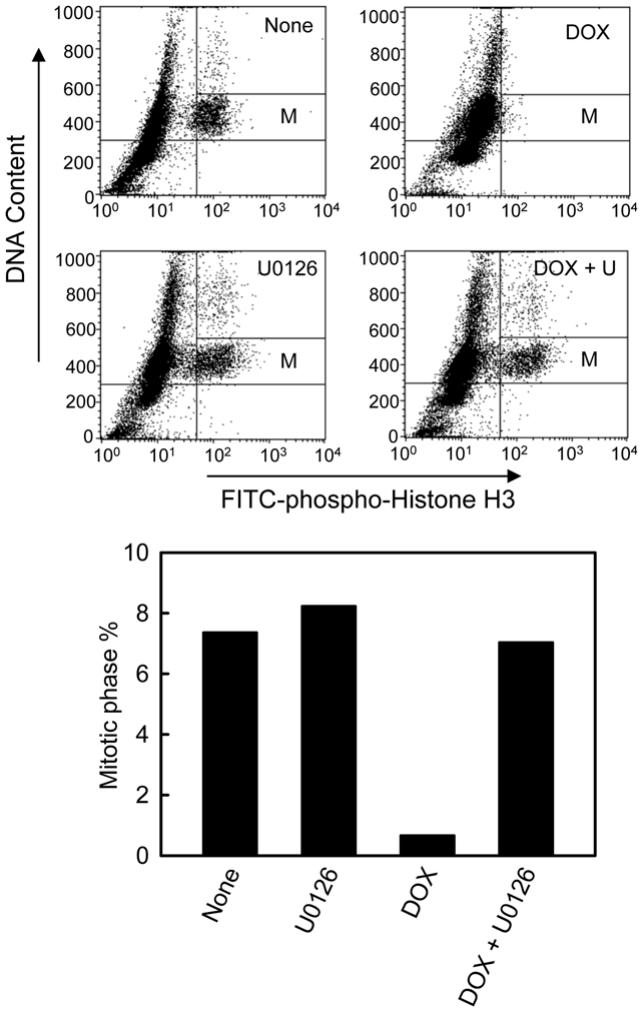
ERK1/2 inhibition abrogates IR-induced G2/M checkpoint activation. MCF-7 cells were treated with or without 1 μM DOX in the presence or absence of 50 μM U0126 for 2 hr and washed. The cells were incubated in regular growth medium for additional 22 hr, in the presence of 100 ng/ml nocodazole, and analyzed for mitotic cells by FACS, which contain both *4N*-DNA content and Histone H3-Ser10 phosphorylation, as described in *Materials and Methods*. Upper panel: the histograms shown are representative FACS analyses for mitotic cells in samples treated with/without DOX in the presence or absence of U0126. The location of mitotic cells in each sample is indicated (*M*). Lower panel: the bar graph compares percentage of mitotic cells presented in the indicated cell samples. Results shown are representative of two separate experiments.

Using histone-H3 phosphorylation as a marker of cells in mitosis [65], we examined the effect of ERK1/2 signaling on the proportion of cells in mitosis following treatment with DOX. As shown in Figure 5, incubation with DOX resulted in a marked decrease in the proportion of cells in mitosis in MCF-7 cell population. At 24 hr post DOX treatment of MCF-7 cells, there was >90% decrease in mitotic cells relative to control none-treated cells (Figure 5, bar graph: *DOX vs. None*). In contrast, incubation of cells with U0126 blocked the effect of DOX, resulting in a large increase in the proportion of mitotic cells in DOX-treated cells compared to the cells treated with DOX alone (Figure 5, Bar graph: *DOX+U0126 vs. DOX*). Incubation of cells with U0126 alone resulted in a slight increase in the amount of mitotic cells compared to the control none-treated cells (Figure 5, Bar graph: *U0126 vs*. *None*).

### ERK1/2 inhibition abrogates topo II poison-induced activation of ATR but not ATM signaling

Activation of ATM and ATR signaling following DNA damage has been shown to play an important role in the activation of the G2/M checkpoint response [66]. We next examined the effect of ERK1/2 inhibition on topo II poison-induced activation of ATM and ATR signaling.

**Figure 6:**
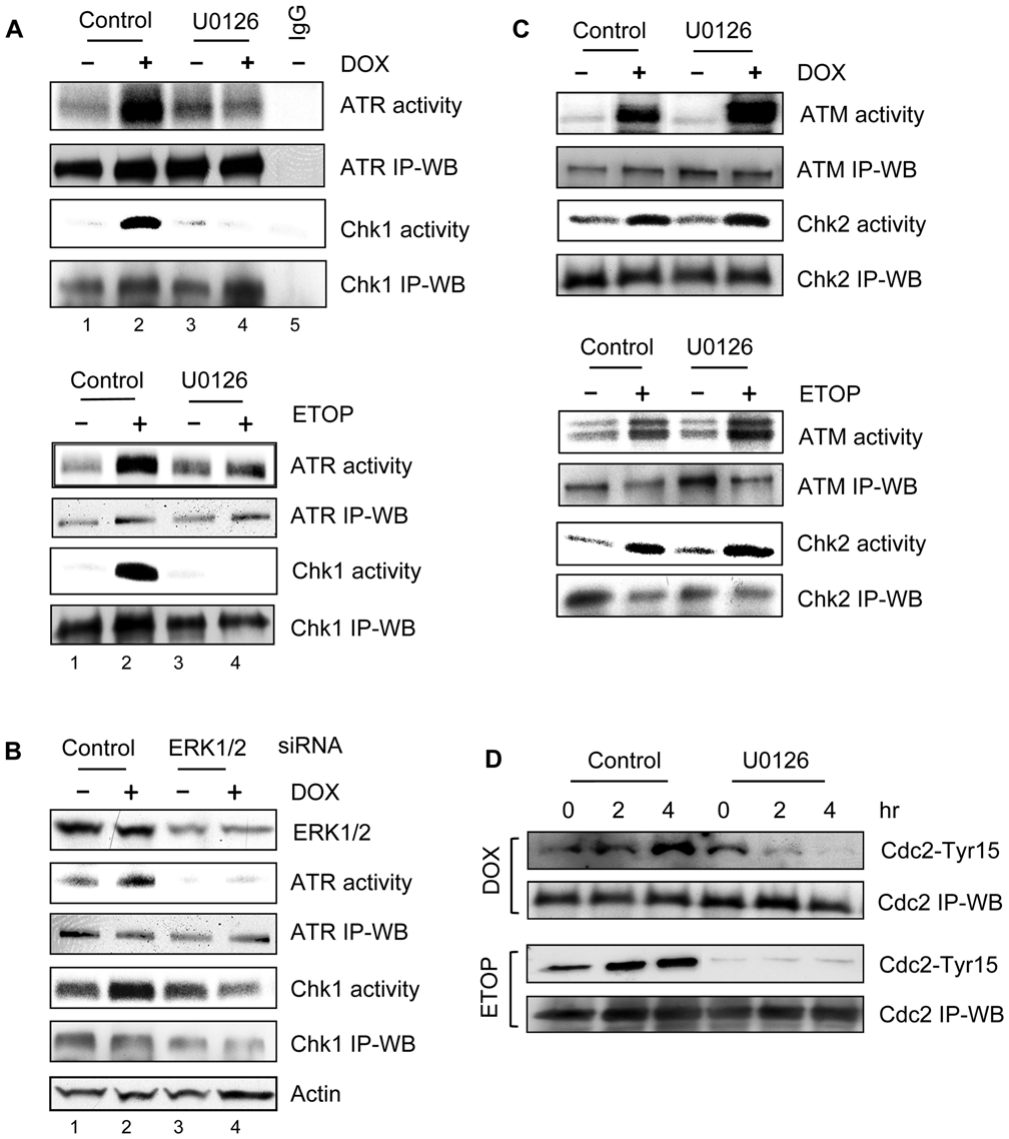
Effect of ERK1/2 inhibition on topo II poison-induced ATR and ATM signaling activation. (A) MCF-7 cells were treated for 2 hr with 1 µM DOX (upper panel) or 10 µM ETOP (lower panel) in the presence or absence of 50 µM U0126. The cells were washed and incubated in growth medium for additional 2 hr with the presence or absence of U0126. ATR and Chk1 kinase were respectively immunoprecipitated from the resulting cell lysates and assayed for kinase activity. ATR and Chk1 levels in immunoprecipitates were determined by immunoblotting (*ATR IP-WB* and *Chk1 IP-WB*). *IgG*, as a negative control, kinase assay was carried out using immunoprecipitates obtained by incubating control untreated cell lysate with non-immunized IgG. (B) Cells transfected with ERK1/2 specific or control siRNA were incubated for 2 days and treated with or without 0.5 µM DOX, as described above. ATR and Chk1 were respectively immunoprecipitated from the cell lysates and examined for kinase activity (*ATR activity* and *Chk1 activity*). ATR and Chk1 protein levels in immunoprecipitates were determined by immunoblotting (*ATR IP-WB* and *Chk1 IP-WB*). Levels of ERK1/2 and Actin in cell lysates were analyzed by immunoblotting (*ERK1/2* and *Actin*). (C) Cells were treated as described in (A). ATM and Chk2 were immunoprecipitated from the cell lysates and assayed for kinase activity (*ATM activity* and *Chk2 activity*). ATM and Chk2 levels in immunoprecipitates were determined by immunoblotting (*ATM IP-WB* and *Chk2 IP-WB*). (D) MCF-7 cells were treated as described in (A) and incubated for the times indicated. Cdc2 was immunoprecipitated from cell lysate and analyzed for Cdc2-Tyr15 phosphorylation by immunoblotting (*Cdc2-Tyr15*). Cdc2 in the immunoprecipitates was quantified by immunoblotting (*Cdc-2 IP-WB*).

As shown Figure 6A, treatment with either DOX (upper panel) or ETOP (lower panel) resulted in a marked activation of ATR kinase and its downstream target Chk1 kinase in MCF-7 cells (lane 2 *vs*. 1). However, the activation of ATR and Chk1 was largely inhibited by the incubation of cells with U0126 (Figure 6A: lane 4 *vs*. 2). The changes in ATR and Chk1 activities following treatment with DOX or ETOP in the presence or absence of U0126 were not associated with changes in ATR and Chk1 protein levels, as Western blot analyses indicate that relative equal amounts of ATR and Chk1 protein were presented in the immunoprecipitates used for kinase assays (Figure 6A, *ATR activity vs. ATR IP-WB* and *Chk1 activity vs. Chk1 IP-WB*). The detection of ATR or Chk1 activity was specific, as either kinase activity was not detected by the control kinase assay, which was carried out using non-specific immunoprecipitates obtained by incubating non-immunized IgG with untreated cell lysate (Figure 6A, upper panel: lane 5).

To verify the effect of ERK1/2 inhibition on topo II poison-induced ATR and Chk1 activation, MCF-7 cells were transfected with either ERK1/2 specific or control siRNA and then treated with DOX. As shown in Figure 6B, ERK1/2 siRNA transfected cells exhibited a marked diminution in both ATR and Chk1 activation following DOX treatment compared to the cells transfected with control siRNA (*ATR activity* and *Chk1 activity*: lane 4 *vs*. 2). In contrast, transfection of control siRNA had no effect on DOX-induced activation of ATR and Chk1 kinases relative to non-transfected cells (data not shown). These data support a role for ERK1/2 in topo II poison-induced activation of ATR and Chk1 kinases.

We next examined the effect of ERK1/2 inhibition on topo II poison-induced ATM and Chk2 activation. As shown in Figure 6C, while treatment of MCF-7 cells with either DOX (upper panel) or ETOP (lower panel) resulted in a marked activation in ATM kinase activity, incubation of cells with U0126 did not block the activation of ATM by DOX or ETOP (*ATM activity*). In fact, the ATM activation by DOX or ETOP was further enhanced by incubation with U0126 (Figure 6C, *ATM activity*). Consistent with the effect of U0126 on ATM activity in DOX- or ETOP-treated cells, incubation with U0126 also did not block the Chk2 activation following DOX or ETOP treatment (Figure 6C, *Chk2 activity*).

Collectively, these results suggest that ERK1/2 signaling is required for topo II poison-induced activation of ATR/Chk1 signaling. However, ERK1/2 signaling is not involved in the topo II poison-induced activation of ATM/Chk2 signaling.

### ERK1/2 inhibition abolishes topo II poison-induced Cdc2-Tyr15 phosphorylation

G2/M transition of the cell cycle requires the activity of Cdc2/Cyclin B complex [12] and, in Figure 2B, we show that treatment with topo II poison results in an increase in Cdc2-Tyr15 inhibitory phosphorylation in MCF-7 cells (Figure 2B). We thus examined the effect of ERK1/2 inhibition on Cdc2-Tyr15 phosphorylation in DOX- or ETOP-treated MCF-7 cells. As shown in Figure 6D, both DOX- and ETOP-induced increase in Cdc2-Tyr15 phosphorylation was abolished by the incubation of cells with U0126 (*Cdc2-Tyr15*).

### ATR but not ATM is required for Dox-induced G2/M arrest

The data presented above indicate that ERK1/2 signaling plays an important role in the regulation of topo II poison-induced G2/M checkpoint activation and that inhibition of ERK1/2 signaling specifically blocks topo II poison-induced activation of ATR kinase (Figure 3–6). Since previous studies have indicated that both ATM and ATR may be involved in the DNA damage induced G2/M checkpoint response [66], we further explored the roles of ATR and ATM in topo II poison-induced G2/M arrest in MCF-7 cells.

**Figure 7:**
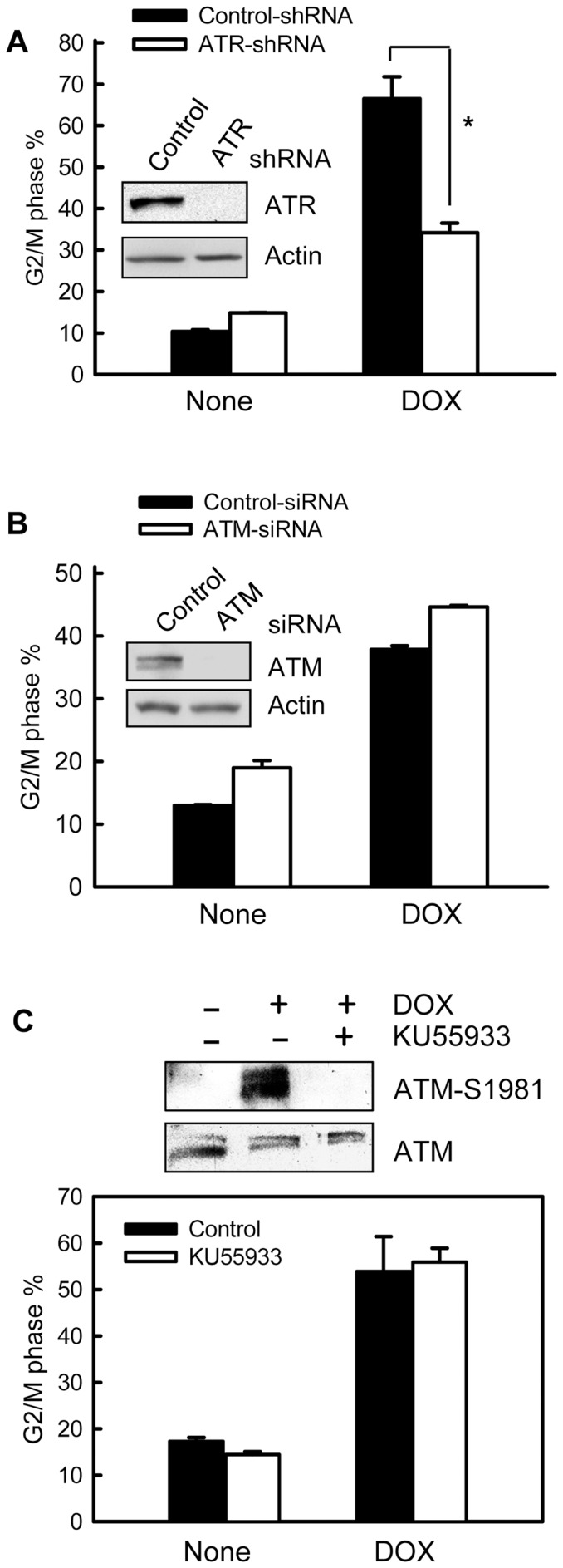
Effect of ATR and ATM expression on DOX-induced G2/M arrest. (A) MCF-7 cells stably expressing ATR specific shRNA (*ATR-shRNA*) or control shRNA (*Control-shRNA*) were treated with or without 0.5 µM DOX, incubated for 24 hr and analyzed for DNA content by FACS. Bar graph depicts the percentage of cells with *4N*-DNA content and represents the mean ± s.d of two independent experiments with duplicate samples. *p<0.001 (n = 4), significant difference from the Control-shRNA expressing cells. Inset: levels of ATR and Actin in Control- and ATR-shRNA expressing cells were determined by immunoblotting. (B) MCF-7 cells were transfected with ATM specific (*ATM-siRNA*) or control siRNA (*Control-siRNA*), incubated for 2 days and treated with or without 0.5 µM DOX. The cells were incubated for 24 hr and analyzed for DNA content by FACS. Graph depicts percentage of cells with *4N*-DNA content and represents the mean ± s.d of two separate experiments with duplicate samples. Inset: levels of ATM and Actin in the transfected cells were determined by immunoblotting. (C) MCF-7 cells were pre-incubated for 1 hr in the presence or absence of 10 µM KU55933 and then treated with 1 µM DOX for 2 hr in the presence or absence of KU55933. Upper panel: the cells were lysed and analyzed for ATM-Ser1981 phosphorylation and total ATM by immunoblotting. Lower panel: the treated cells were incubated for additional 24 hr in the presence or absence of KU55933 and analyzed for DNA content by FACS. Bar graph depicts the percentage of cells with *4N*-DNA content and represents the mean ± s.d of two independent experiments in duplicate.

To assess the role of ATR kinase in topo II poison-induced G2/M arrest, MCF-7 cells were stably infected with retroviral vector expressing ATR specific shRNA or control firefly luciferase shRNA. As shown in Figure 7A (inset), cells expressing ATR-shRNA exhibited a marked reduction in ATR protein levels compared to the control cells (*ATR*). Furthermore, as shown in Figure 7A, DOX-induced G2/M arrest was diminished by approximate 50% in ATR-shRNA expressing cells compared to Control-shRNA expressing cells. In contrast, infection of MCF-7 cells with control retrovirus had no detectable effect on DOX-induced G2/M arrest compared to uninfected cells (data not shown).

To explore the role of ATM in topo II poison-induced G2/M arrest, MCF-7 cells were transfected with ATM specific or control siRNA. As shown in Figure 7B (inset), ATM protein levels were largely reduced following transfection of MCF-7 cells with ATM specific siRNA compared to control siRNA transfected cells (*ATM*). As shown in Figure 7B (open bar), cell cycle analysis indicated that decreased expression of ATM did not result in attenuation of DOX-induced G2/M arrest. To further verify the effect of ATM on DOX-induced G2/M arrest, ATM specific inhibitor KU55933 was used to inhibit DOX-induced ATM activation. As shown in Figure 7C (upper panel), incubation of cells with KU55933 completely inhibited DOX-induced ATM-S1981 phosphorylation. However, the incubation did not block the induction of G2/M arrest following DOX treatment (lower panel).

Taken together, these results suggest that ATR but not ATM plays an essential role in DOX-induced G2/M checkpoint activation.

### Decreased ATR expression by shRNA has no effect on DOX-induced ERK1/2 activation

**Figure 8:**
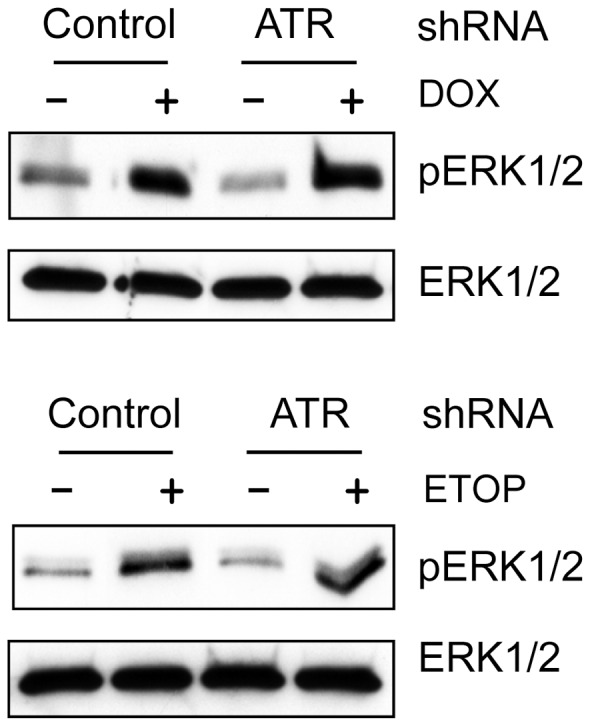
Decrease of ATR level by shRNA had no effect on DOX-induced ERK1/2 activation. MCF-7 cells expressing ATR specific or control shRNA were treated with or without 1 µM DOX (upper panel) or 10 µM ETOP (lower panel) for 2 hr and analyzed for phospho-ERK1/2 (*pERK1/2*) and total ERK1/2 (*ERK1/2*) by immunoblotting.

The studies presented above indicate that inhibition of ERK1/2 blocked the topo II poison-induced ATR activation (Figure 6A and B). Further studies provide evidence supporting an essential role for ATR in DOX-induced G2/M arrest (Figure 7A). We next examined role of ATR in topo II poison-induced ERK1/2 activation. As shown in Figure 8, expression of ATR shRNA, which markedly decreased ATR protein levels in MCF-7 cells (Figure 7A), had no effect on the increase of ERK1/2 phosphorylation in MCF-7 cells following treatment with DOX or ETOP. These results indicate that ATR is not required for topo II poison-induced ERK1/2 activation.

### The regulation of DOX-induced G2/M arrest by p53 is independent of ERK1/2 signaling

While results in Figure 3 and 4 indicate that ERK1/2 inhibition attenuated topo II poison-induced G2/M arrest in both MCF-7 and T47D cells, the attenuation observed in T47D cells is more complete than that in MCF-7 cells, which suggests the involvement of additional mechanisms other than ERK1/2 signaling in the regulation of topo II poison-induced G2/M checkpoint response. It has been previously demonstrated that MCF-7 cells express wild-type p53 whereas T47D cells contain a mutant dysfunctional p53 [67]. We thus investigated the effect of p53 expression on DOX-induced G2/M arrest in MCF-7 cells in the presence or absence of ERK1/2 inhibition.

**Figure 9:**
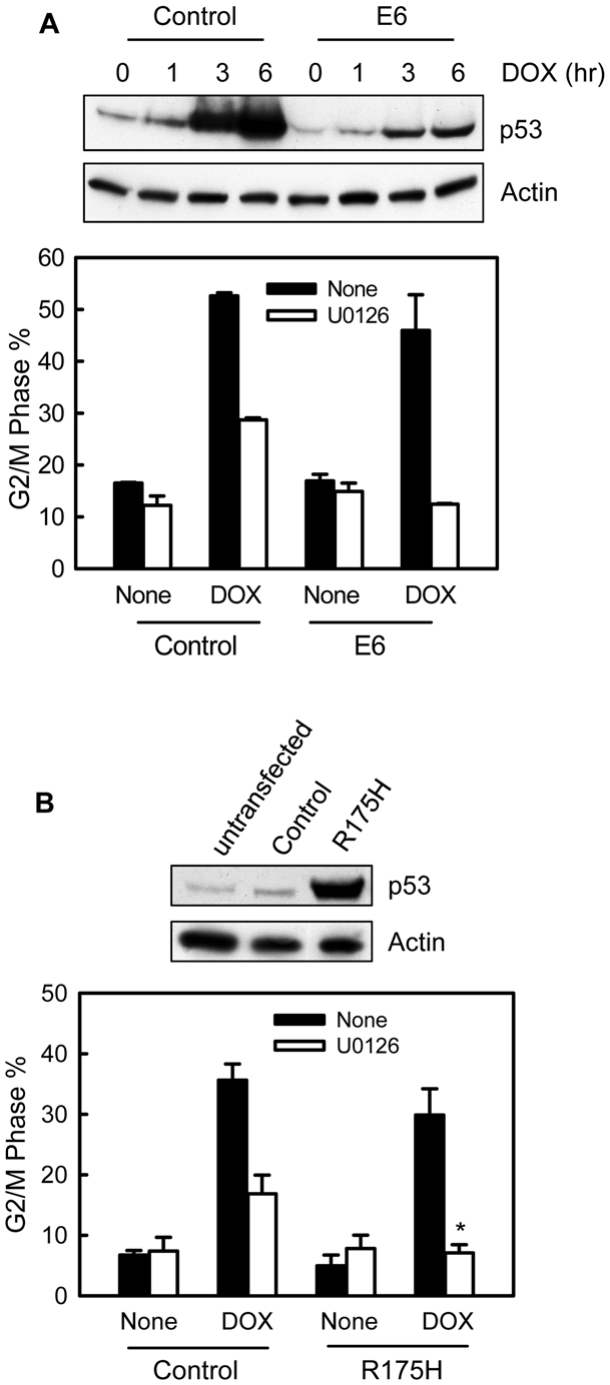
Effect of p53 expression on DOX-induced G2/M arrest. (A) Upper panel: MCF-7 cells stably expressing HPV-E6 (*E6*) and control cells (*Control*) were treated with 1 µM DOX for the indicated hours and analyzed for levels of p53 and Actin by immunoblotting. Lower panel: HPV-E6 expressing and control cells were treated with or without 1 µM DOX in the presence or absence of 50 µM U0126, incubated for 24 hr and analyzed for DNA content by FACS. Bar graphs depict the percentage of cells with *4N*-DNA content (G2/M phase cells) and represent the mean ± s.d of two independent experiments in duplicate. (B) MCF-7 cells were transfected with either vector expressing p53-R175H (*R175H*) dominant negative mutant or control empty vector (*Control*) and incubated for 48 hr. Upper panel: levels of p53 and Actin in the transfected or unstransfected cells were compared by immunoblotting. Lower panel: p53-R175H transfected and control cells were treated with 0.25 µM DOX in the presence or absence of U0126, incubated for 24 hr and analyzed for DNA content by FACS. Bar graphs depict the percentage of cells with *4N*-DNA content and represent the mean ± s.d. of two independent experiments in duplicate. *p = 0.001 (n = 4), significant difference from the control transfected cells treated with DOX in the presence of U0126.

We first used HPV-E6 to examine the effect of p53 on DOX-induced G2/M arrest in MCF-7 cells. It has been previously shown that HPV-E6 functions as ubiquitin E3 ligase that induces proteasome-mediated p53 degradation [46]. As shown in Figure 9A (upper panel), DOX treatment of control MCF-7 cells resulted in a time-dependent marked induction in p53 protein, which is consistent with previous studies [68]. In contrast, in the presence of HPV-E6 expression, treatment of MCF-7 cells with DOX only led to a subtle increase in p53 protein (Figure 9A, upper panel). As shown in Figure 9A (lower panel), while incubation of control cells with U0126 resulted in an incomplete attenuation in DOX-induced G2/M arrest, incubation of HPV-E6 expressing cells with U0126 completely abrogated DOX-induced G2/M arrest. In the absence of ERK1/2 inhibition, the DOX treatment induced slight less G2/M arrest in the HPV-E6 expressing cells compared to control MCF-7 cells (Figure 9A, lower panel). However, the difference was not statistically significant.

To verify the results obtained using HPV-E6, MCF-7 cells expressing p53-R175H dominant negative mutant were used for studies. As shown in Figure 9B (upper panel), cells transfected with p53-R175H expressing vector showed a large increase in total p53 levels compared to vector transfected or non-transfected cells. As shown Figure 9B (lower panel), while incubation of control transfected cells with U0126 resulted in an incomplete attenuation in DOX-induced G2/M arrest, incubation of p53-R175H expressing cells with U0126 completely abolished the DOX-induced G2/M arrest. Furthermore, similar to the results obtained from the studies using HPV-E6, in the absence of ERK1/2 inhibition, DOX treatment also induced slight less G2/M arrest in p53-R175H expressing cells compared to control cells (Figure 9B, lower panel).

Collectively, these results suggest that p53 has a positive regulation on DOX-induced G2/M arrest. However, this role of p53 is independent of ERK1/2.

### p38 and MK2 kinases have no effect on topo II poison-induced G2/M checkpoint response

Previous studies have reported a role of p38-mediated MK2 activation in DOX-induced G2/M checkpoint activation in U2OS osteosarcoma cells [43]. We therefore investigated the possible contribution of p38 and MK2 to the topo II poison-induced G2/M checkpoint response in MCF-7 cells.

**Figure 10:**
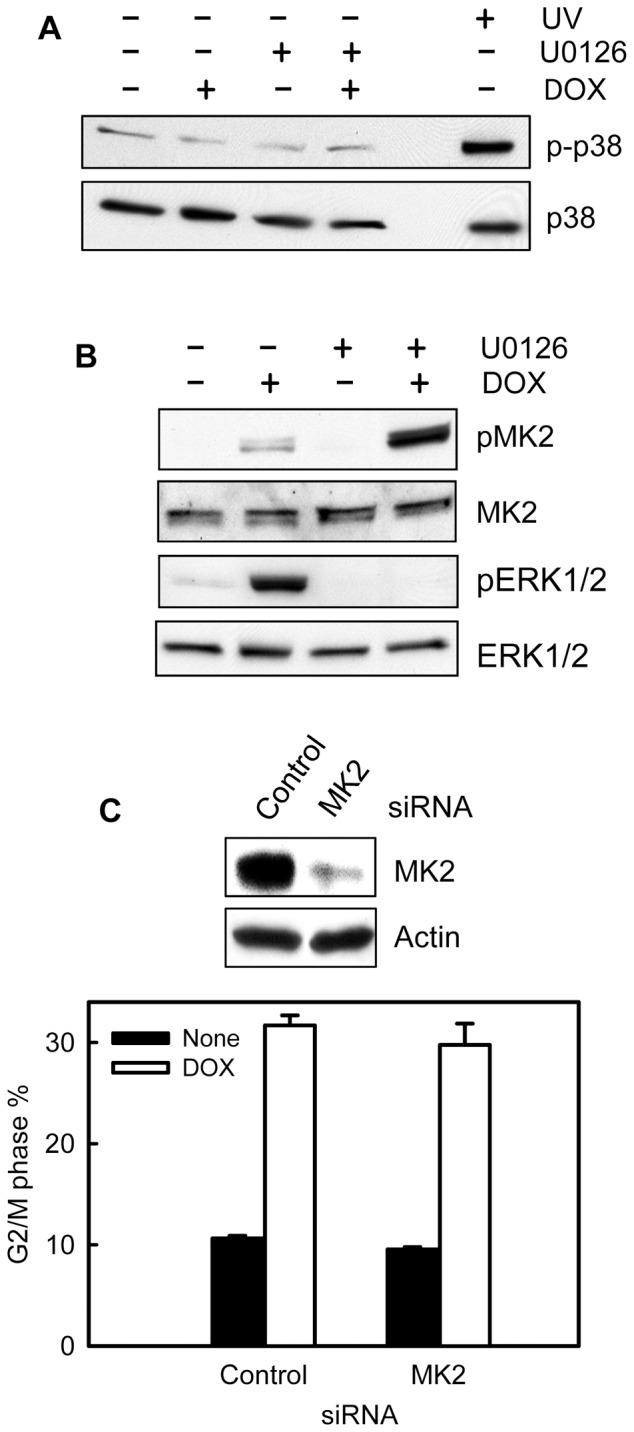
DOX-induced G2/M checkpoint activation does not involve the p38 and MK2 kinases. (A) MCF-7 cells were treated for 2 hr with or without 1 μM DOX in the presence or absence of 50 μM U0126. As a positive control for p38 activation, a cell sample was exposed to UV at 100 J/m^2^ and incubated for 1 hr at 37°C. The resulting cells were analyzed for levels of phospho-p38 (*p-p38*) and total p38 (*p38*) by immunoblotting. (B) The cell samples obtained above were analyzed for levels of phospho-MK2 (*pMK2*), total MK2 (*MK2*), phospho-ERK1/2 (*pERK1/2*) and total ERK1/2 (*ERK1/2*) by immunoblotting. (C) MCF-7 cells were transfected with control (*Control*) or MK2 specific siRNA (*MK2*) and incubated for 2 days at 37°C. Upper panel: levels of MK2 and Actin in the transfected cells were determined by immunoblotting. Lower panel: The transfected cells were treated with 1 μM DOX, incubated for 24 hr and analyzed for DNA content by FACS. Graph depicts percentage of cells with *4N*-DNA content and represents the mean ± s.d of two separate experiments with duplicate samples.

As shown in Figure 10A, treatment of cells with DOX did not result in p38 activation, as determined by Western blot analysis of p38 phosphorylation (*p-p38*). As a positive control, exposure of MCF-7 cells to UV did result in a marked increase in p38 phosphorylation (Figure 10A, *p-p38*).

We next examined the role of MK2 in DOX-induced G2/M checkpoint response in MCF-7 cells. As shown in Figure 10B, treatment with DOX markedly increased MK2 phosphorylation in MCF-7 cells. Furthermore, incubation of MCF-7 cells with the MEK1/2 inhibitor U0126, which abrogated DOX-induced ERK1/2 activation (Figure 10B, *pERK1/2*), resulted in a further increase in MK2 phosphorylation in MCF-7 cells (Figure 10B, *pMK2*). Thus, DOX-induced MK2 phosphorylation is not dependent on ERK1/2 activation. However, the results presented in Figure 10C show that transfection of MCF-7 cells with MK2 specific siRNA, resulting in a marked decrease in MK2 protein (upper panel), had no effect on DOX-induced G2/M arrest compared to control siRNA transfected cells (open bars). Thus, MK2 activation apparently does not play a role in DOX-induced G2/M arrest in MCF-7 cells.

We also examined the effect of DOX treatment on p38 and MK2 in T47D cells. As results shown in Supplemental Figure S4, treatment with DOX did not result in activation of either p38 or MK2 in T47D cells. In contrast, exposure of T47D cells to UV irradiation greatly activated both p38 and MK2. Thus, p38 and MK2 apparently are not involved in the regulation of DOX-induced G2/M checkpoint response in T47D cells.

### Inhibition of ERK1/2 signaling enhances topo II poison-induced apoptosis

**Figure 11:**
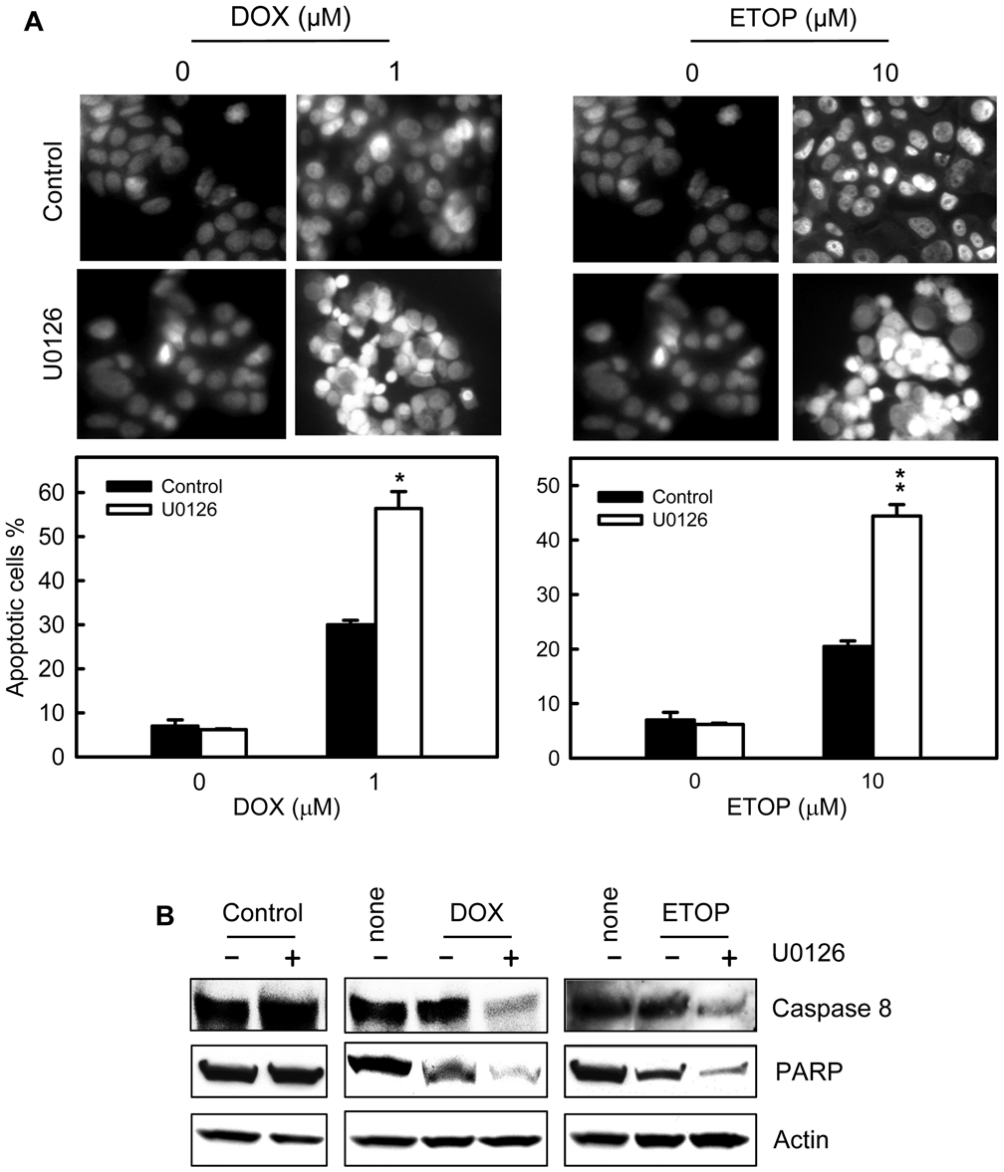
Inhibition of ERK1/2 signaling increases topo II poison-induced apoptosis. (A) MCF-7 cells were treated for 2 hr with or without 1 µM DOX or 10 µM ETOP in the presence or absence of 50 µM U0126. Following treatment, the cells were washed, incubated for 3 days with/without presence of U0126 and analyzed for apoptosis by DAPI staining and fluorescence microcopy. Upper panels: images shown are the DAPI staining of the resulting cells. Lower panels: the percentage of apoptotic cells is shown as mean ± s.d of quadruplicate samples. *p<0.01 (n = 4), significant difference from cells treated with DOX alone. **p<0.005 (n = 4), significant difference from cells treated with ETOP alone. (B) The cells obtained above were analyzed for levels of full-length PARP and Caspase 8 by Western blot analysis. The protein loading were assessed by immunoblotting for Actin levels (*Actin*).

We next examined the effect of ERK1/2 inhibition on cell survival following treatment with DOX or ETOP. As shown in Figure 11A (left panels), treatment of cells with DOX alone resulted in a 4-fold increase in apoptotic cells compared to control non-treated cells (solid bars), whereas treatment with DOX in the presence of U0126 resulted in a 9-fold increase in apoptotic cells compared to control untreated cells (open bars). Inhibition of ERK1/2 also had a similar effect on ETOP-induced apoptosis. As shown in Figure 11A (right panels), while treatment with ETOP alone resulted in a 3-fold increase in apoptotic cells compared to control untreated cells (solid bars), treatment with ETOP in the presence of U1026 resulted in a 7-fold increase in apoptotic cells relative to control untreated cells (open bars). Incubation of MCF-7 cells with U0126 by itself had no noticeable effect on the amount of apoptotic cells compared to control untreated cells (Figure 11A).

To confirm the induction of apoptosis in these cells, a parallel set of cell samples were analyzed by Western blotting to assess the integrity of Caspase 8 and PARP protein. The cleavage of caspases and PARP, hallmarks of apoptosis, occurs during the execution phase of programmed cell death [62], [69], [70]. As shown in Figure 11B, while treatment of MCF-7 cells with DOX or ETOP alone resulted in only a subtle decrease in levels of full-length Caspase 8 compared to control untreated cells, treatment with DOX and ETOP in the presence of U0126 resulted in a marked decrease in full-length Caspase 8. Relative to control untreated cells, a 72% decrease in full-length Caspase 8 was detected in cells treated with both DOX and U0126 and a 56% decrease detected in the cells treated with both ETOP and U0126 (Figure 11B, *Caspase 8*). Similar results were also obtained from examining amount of intact PARP in these samples. Using an antibody recognizing full-length PARP, the results in Figure 11B showed that the levels of intact PARP was respectively decreased by 44% in cells treated with DOX and by 47% in cells treated with ETOP (*PARP*). In the presence of U0126, treatment with DOX or ETOP resulted in a further decrease in levels of intact PARP. Relative to the control non-treated cells, an 89% decrease in level of intact PARP was observed in cells treated with both DOX and U0126 and an 81% decrease observed in cells treated with both ETOP and U0126 (Figure 11B, *PARP*). In contrast, treatment of cells with U0126 alone failed to lower the levels of either intact Caspase 8 or PARP in MCF-7 cells (Figure 11B).

## Discussion

Previous studies have indicated that DNA damage induced G2/M checkpoint activation involves the activation of ATM and ATR signaling. This in turn leads to the activation of Chk1 and Chk2 kinases, inhibition of Cdc2/Cyclin B and subsequently G2/M cell cycle arrest [29], [71]. Consistent with these findings, the results in this report indicate that topo II poison-induced G2/M arrest in MCF-7 cells is also associated with an activation of Chk1 and Chk2 kinases (see Figure 2A), and an inhibition of Cdc2 kinase (see Figure 2B). Furthermore, results in this report demonstrate that topo II poison-induced G2/M arrest is associated with a rapid activation of ERK1/2 (see Figure 1B) and that ERK1/2 activation is persistent up to 24 hr post drug treatment (supplemental Figure S1). Using a MEK1/2 specific inhibitor, as well as siRNA targeting ERK1/2, results in this report indicate that inhibition of ERK1/2 singling abrogates topo II poison-induced activation of ATR and Chk1 kinases and attenuates the induction of G2/M arrest following topo II poison treatment (see Figure 3–6). These results implicate an important role for ERK1/2 signaling in topo II poison-induced G2/M checkpoint activation.

A recent study reports a role of ERK1/2 expression in ETOP-induced G2/M arrest in MCF-7 cells. Furthermore, the study also notes an increase of ATM-S1981 phosphorylation following ETOP treatment and that is correlated with an ETOP-induced G2/M cell cycle arrest [72]. However, whether ATM actually plays a role in ETOP-induced G2/M arrest in MCF-7 cells has not been examined by the study. Results in the present report indicate that ERK1/2 inhibition abolishes the topo II poison-induced ATR and Chk1 activation (see Figure 6A and B), but has little effect on the topo II poison-induced ATM and Chk2 activation (see Figure 6C). Furthermore, while decrease of ATR expression by shRNA results in significant attenuation of DOX-induced G2/M arrest (see Figure 7A), decreased expression or inhibition of ATM fail to block the induction of G2/M arrest following DOX treatment (see Figure 7B and C). These results are consistent with previous studies, which indicate that Chk1 but not Chk2 was critical for DOX-induced G2/M cell cycle arrest in HeLa human cervical cancer cells [73].

Results in this report demonstrate that both ERK1/2 and ATR are essential for topo II poison-induced G2/M arrest and ERK1/2 inhibition abrogates the ATR activation following topo II poison treatment. However, decreased ATR expression (see Figure 8) or incubation of cells with caffeine (data not shown), which inhibits both ATM and ATR kinases [54], does not block the topo II poison-induced ERK1/2 activation. These results suggest that ERK1/2 activation is up-stream of ATR activation following topo II poison-induced DNA damage.

While ERK1/2 inhibition attenuates topo II poison-induced G2/M arrest in both MCF-7 and T47D cells, it should be noted that the attenuation observed in T47D cells is more complete than in MCF-7 cells. These results suggest that, in MCF-7 cells, there may be additional pathway(s) other than ERK1/2 signaling involved in the control of G2/M checkpoint response following topo II poison treatment. One possible mechanism might involve tumor suppressor p53. It has been shown that MCF-7 cells express wild-type p53 whereas T47D cells have lost p53 function [74]. In addition, a recent report shows that, in H1299 non-small cell lung carcinoma cells, ectopic expression of p53 promotes DOX-induced G2/M cell cycle arrest, which involves the induction of p21^Waf1/Cip1^ [9]. In the present studies, we have investigated the role of p53 in DOX-induced G2/M arrest in MCF-7 cells in the presence or absence of ERK1/2 inhibition. The results of these studies indicate that disruption of p53 function in MCF-7 cells, either by the expression of HPV-E6 or p53-R175H dominant negative mutant, results in a further attenuation in DOX-induced G2/M arrest in MCF-7 cells incubated with U0126 (see Figure 9). These results suggest that, other than ERK1/2 signaling, p53 also has a role in promoting G2/M arrest following topo II poison treatment. However, this role of p53 appears to operate independently of ERK1/2 signaling.

Previous studies show that DOX treatment of U2OS human osteosarcoma cells results in the activation of p38 and MK2 kinases and that this is essential for the activation of G2/M checkpoint response in U2OS cells following DOX treatment [43]. Furthermore, these studies also demonstrate that the role of p38/MK2 signaling on the regulation of G2/M checkpoint activation is independent of Chk1 [43]. In the present studies, we have examined the potential contribution of p38 and MK2 to DOX-induced G2/M checkpoint response in MCF-7 breast cancer cells. The results presented in this report indicate that DOX treatment of MCF-7 cells results in an activation of MK2 kinase but not p38 kinase (see Figure 10A and 10B). Additional studies in this report indicate that decreased MK2 expression in MCF-7 cells has no effect on DOX-induced G2/M arrest (see Figure 10C). Furthermore, similar studies in T47D breast cancer cells indicate that DOX treatment of these cells does not activate either p38 or MK2 (see supplemental Figure S4), but induces G2/M arrest (see Figure 3). These results suggest that the regulation of p38 and MK2 kinases on DOX-induced G2/M checkpoint response is cell type specific. While these kinases apparently play a role in topo II poison-induced G2/M arrest in U2OS osteosarcoma cells, the results in this report indicate they are not involved in DOX-induced G2/M arrest in MCF-7 and T47D breast cancer cells.

Previous studies have shown that ERK1/2 signaling inhibition enhances topo II poison-induced cytotoxicity in human epidermoid carcinoma and gastric cancer cells [75], [76]. In this report, we have investigated the role of ERK1/2 signaling in topo II poison-induced apoptosis. Results obtained from this study are consistent with the previous finding, indicating that ERK1/2 inhibition markedly increases topo II poison-induced apoptosis in MCF-7 breast cancer cells (see Figure 11). On the other hand, it had been reported that inhibition of ERK1/2 signaling in NIH3T3 mouse fibroblast cells results in diminution in DOX- and ETOP-induced apoptosis [44], [77]. Thus the effect of ERK1/2 inhibition on topo II poison sensitivity is also apparently cell type specific.

While results of the present studies suggest an important role of ERK1/2 signaling in topo II poison induced G2/M cell cycle arrest, it remains undetermined by what mechanism ERK1/2 signaling is activated in response to topo II poison treatment. A previous study by Navarro *et* *al* [78] suggests a involvement of superoxide anions in DOX-induced ERK1/2 activation in hypatocytes, which are probably generated by DOX metabolism. We will examine this possibility in the future studies.

It has been previously demonstrated that topo II poison treatment primarily results in a prolonged cell cycle arrest in most breast cancer cell lines studied [79]. Additional studies in various types of cancer cells show that a portion of the cells that survive the topo II poison treatment ultimately develop a senescence-like phenotype (e.g. 79% of Doxorubicin-treated HT1080 cells) [80]. This phenotype includes an enlarged and flattened morphology, expression of the senescence marker SA-β-Galactosidase and increased granularity. Thus, a progression toward senescence-like terminal growth arrest is one of the destinies for cells arrested in G2 phase following topo II poison treatment. As for the other portion of cells, which also survive the topo II poison treatment but do not develop a senescence-like phenotype, their fate(s) has not yet been clearly determined by the current studies.

In summary, results presented in this report suggest an important role of ERK1/2 signaling in the regulation of G2/M checkpoint activation following topo II poison treatment. The studies presented in this report indicate that topo II poison-induced ERK1/2 signaling is upstream of ATR and Chk1 activation, which, in turn, results in decreased Cdc2 activity and subsequent G2/M cell cycle arrest. Ultimately, the results of the present studies suggest an important role of ERK1/2 signaling in protecting cells from topo II poison-induced apoptosis.

## Supporting Information

Figure S1 **DOX and ETOP induce ERK1/2 activation in MCF-7 breast cancer cells.** MCF-7 cells were incubated in the presence of 1 µM DOX or 10 µM ETOP for the hours indicated and analyzed for phospho-ERK1/2 and total-ERK1/2 by immunoblotting.
(TIF)

Figure S2 **Transfection of non-targeting control siRNA had no effect on DOX-induced G2/M cell cycle arrest in MCF-7 cells.** MCF-7 cells were transfected with control non-targeting siRNA or left untransfected and incubated for 2 days. (A) Left panel: the cells were analyzed for protein levels of ERK1/2 and Actin by Western blotting. Right panel: Immunoblot densities of ERK1/2 and Actin were quantified using ImageJ software and relative ERK1/2 expression versus Actin determined. (B) The cells were then treated with 0.5 µM DOX, incubated for 24 hr and analyzed for DNA content by FACS. Histograms shown are DNA content analyses for the indicated cell samples.
(TIF)

Figure S3 **Treatment with U0126 has no effect on the cell cycle profile of MCF-7 cells.** MCF-7 cells were incubated in the presence or absence of U0126 for 24 hr and analyzed for DNA content by FACS. Histograms shown are DNA content analyses for the indicated cell samples.
(TIF)

Figure S4 **Treatment of T47D breast cancer cells with DOX does not activate p38 and MK2 kinases.** T47D cells were treated for 2 hr with or without 1 μM DOX in the presence or absence of 50 μM U0126. As a positive control for p38 and MK2 activation, a cell sample was exposed to UV at 100 J/m^2^ and incubated for 1 hr at 37°C. The resulting cells were analyzed for levels of phospho-p38 (*p-p38*), total p38 (*p38*), phospho-MK2 (*pMK2*) and total MK2 (*MK2*) by immunoblotting.
(TIF)

## References

[1] van Gijn R, Lendfers R, Schellens J, Bult A, Beijnen J (2000) Dual topoisomerase I/II inhibitors. Journal of Oncology Pharmacy Practice 6: 92–108.

[2] Nitiss JL (2009) Targeting DNA topoisomerase II in cancer chemotherapy. Nat Rev Cancer 9: 338–350. 19377506 10.1038/nrc2607 PMC2748742

[3] Potter AJ, Rabinovitch PS (2005) The cell cycle phases of DNA damage and repair initiated by topoisomerase II-targeting chemotherapeutic drugs. Mutat Res 572: 27–44. 15790488 10.1016/j.mrfmmm.2004.11.018

[4] Kim HS, Lee YS, Kim DK (2009) Doxorubicin exerts cytotoxic effects through cell cycle arrest and Fas-mediated cell death. Pharmacology 84: 300–309. 19829019 10.1159/000245937

[5] Baldwin EL, Osheroff N (2005) Etoposide, topoisomerase II and cancer. Curr Med Chem Anticancer Agents 5: 363–372. 16101488 10.2174/1568011054222364

[6] Lee TK, Lau TC, Ng IO (2002) Doxorubicin-induced apoptosis and chemosensitivity in hepatoma cell lines. Cancer Chemother Pharmacol 49: 78–86. 11855756 10.1007/s00280-001-0376-4

[7] Jin Z-H, Kurosu T, Yamaguchi M, Arai A, Miura O (2005) Hematopoietic cytokines enhance Chk1-dependent G2//M checkpoint activation by etoposide through the Akt//GSK3 pathway to inhibit apoptosis. Oncogene 24: 1973–1981. 15674326 10.1038/sj.onc.1208408

[8] Jiang H (2005) Cdk5 activator – binding protein C53 regulates apoptosis induced by genotoxic stress via modulating the G2/M DNA damage checkpoint. Journal of Biological Chemistry 280: 20651–20659. 15790566 10.1074/jbc.M413431200

[9] Lin YC, Wang FF (2008) Mechanisms underlying the pro-survival pathway of p53 in suppressing mitotic death induced by adriamycin. Cell Signal 20: 258–267. 18006273 10.1016/j.cellsig.2007.10.017

[10] Bucher N (2008) G2 checkpoint abrogation and checkpoint kinase-1 targeting in the treatment of cancer. British journal of cancer 98: 523–528. 18231106 10.1038/sj.bjc.6604208 PMC2243162

[11] Montecucco A, Biamonti G (2007) Cellular response to etoposide treatment. Cancer Lett 252: 9–18. 17166655 10.1016/j.canlet.2006.11.005

[12] Smits VA, Medema RH (2001) Checking out the G(2)/M transition. Biochim Biophys Acta 1519: 1–12. 11406266 10.1016/s0167-4781(01)00204-4

[13] Rhind N, Furnari B, Russell P (1997) Cdc2 tyrosine phosphorylation is required for the DNA damage checkpoint in fission yeast. Genes Dev 11: 504–511. 9042863 10.1101/gad.11.4.504

[14] Kharbanda S, Saleem A, Datta R, Yuan ZM, Weichselbaum R, et al (1994) Ionizing radiation induces rapid tyrosine phosphorylation of p34cdc2. Cancer Res 54: 1412–1414. 8137239

[15] O'Connell MJ, Raleigh JM, Verkade HM, Nurse P (1997) Chk1 is a wee1 kinase in the G2 DNA damage checkpoint inhibiting cdc2 by Y15 phosphorylation. Embo J 16: 545–554. 9034337 10.1093/emboj/16.3.545 PMC1169658

[16] Lundgren K, Walworth N, Booher R, Dembski M, Kirschner M, et al (1991) mik1 and wee1 cooperate in the inhibitory tyrosine phosphorylation of cdc2. Cell 64: 1111–1122. 1706223 10.1016/0092-8674(91)90266-2

[17] Parker LL, Atherton-Fessler S, Piwnica-Worms H (1992) p107wee1 is a dual-specificity kinase that phosphorylates p34cdc2 on tyrosine 15. Proc Natl Acad Sci U S A 89: 2917–2921. 1372994 10.1073/pnas.89.7.2917 PMC48774

[18] Bulavin DV, Higashimoto Y, Demidenko ZN, Meek S, Graves P, et al (2003) Dual phosphorylation controls Cdc25 phosphatases and mitotic entry. Nat Cell Biol 5: 545–551. 12766774 10.1038/ncb994

[19] Chen MS, Ryan CE, Piwnica-Worms H (2003) Chk1 kinase negatively regulates mitotic function of Cdc25A phosphatase through 14-3-3 binding. Mol Cell Biol 23: 7488–7497. 14559997 10.1128/MCB.23.21.7488-7497.2003 PMC207598

[20] Peng CY, Graves PR, Thoma RS, Wu Z, Shaw AS, et al (1997) Mitotic and G2 checkpoint control: regulation of 14-3-3 protein binding by phosphorylation of Cdc25C on serine-216. Science 277: 1501–1505. 9278512 10.1126/science.277.5331.1501

[21] Graves PR, Lovly CM, Uy GL, Piwnica-Worms H (2001) Localization of human Cdc25C is regulated both by nuclear export and 14-3-3 protein binding. Oncogene 20: 1839–1851. 11313932 10.1038/sj.onc.1204259

[22] Prigent C, Dimitrov S (2003) Phosphorylation of serine 10 in histone H3, what for? J Cell Sci 116: 3677–3685. 12917355 10.1242/jcs.00735

[23] Goto H, Tomono Y, Ajiro K, Kosako H, Fujita M, et al (1999) Identification of a novel phosphorylation site on histone H3 coupled with mitotic chromosome condensation. The Journal of biological chemistry 274: 25543–25549. 10464286 10.1074/jbc.274.36.25543

[24] Xu B, Kastan MB (2004) Analyzing cell cycle checkpoints after ionizing radiation. Methods Mol Biol 281: 283–292. 15220537 10.1385/1-59259-811-0:283

[25] Hendzel MJ, Wei Y, Mancini MA, Van Hooser A, Ranalli T, et al (1997) Mitosis-specific phosphorylation of histone H3 initiates primarily within pericentromeric heterochromatin during G2 and spreads in an ordered fashion coincident with mitotic chromosome condensation. Chromosoma 106: 348–360. 9362543 10.1007/s004120050256

[26] Sauve DM, Anderson HJ, Ray JM, James WM, Roberge M (1999) Phosphorylation-induced rearrangement of the histone H3 NH2-terminal domain during mitotic chromosome condensation. J Cell Biol 145: 225–235. 10209020 10.1083/jcb.145.2.225 PMC2133119

[27] Xu B, Kim ST, Lim DS, Kastan MB (2002) Two molecularly distinct G(2)/M checkpoints are induced by ionizing irradiation. Mol Cell Biol 22: 1049–1059. 11809797 10.1128/MCB.22.4.1049-1059.2002 PMC134638

[28] Yan Y, Black CP, Cowan KH (2007) Irradiation-induced G2/M checkpoint response requires ERK1/2 activation. Oncogene 26: 4689–4698. 17297454 10.1038/sj.onc.1210268

[29] O'Connell MJ, Cimprich KA (2005) G2 damage checkpoints: what is the turn-on? J Cell Sci 118: 1–6. 15615778 10.1242/jcs.01626

[30] Liu Q, Guntuku S, Cui XS, Matsuoka S, Cortez D, et al (2000) Chk1 is an essential kinase that is regulated by Atr and required for the G(2)/M DNA damage checkpoint. Genes Dev 14: 1448–1459. 10859164 PMC316686

[31] Zhao H, Piwnica-Worms H (2001) ATR-mediated checkpoint pathways regulate phosphorylation and activation of human Chk1. Mol Cell Biol 21: 4129–4139. 11390642 10.1128/MCB.21.13.4129-4139.2001 PMC87074

[32] Ward IM, Wu X, Chen J (2001) Threonine 68 of Chk2 is phosphorylated at sites of DNA strand breaks. J Biol Chem 276: 47755–47758. 11668173 10.1074/jbc.C100587200

[33] Ahn JY, Schwarz JK, Piwnica-Worms H, Canman CE (2000) Threonine 68 phosphorylation by ataxia telangiectasia mutated is required for efficient activation of Chk2 in response to ionizing radiation. Cancer Res 60: 5934–5936. 11085506

[34] Hermeking H, Lengauer C, Polyak K, He TC, Zhang L, et al (1997) 14-3-3 sigma is a p53-regulated inhibitor of G2/M progression. Mol Cell 1: 3–11. 9659898 10.1016/s1097-2765(00)80002-7

[35] Mitchell C, Hamed HA, Cruickshanks N, Tang Y, Bareford MD, et al (2011) Simultaneous exposure of transformed cells to SRC family inhibitors and CHK1 inhibitors causes cell death. Cancer Biol Ther 12: 215–228. 21642769 10.4161/cbt.12.3.16218 PMC3230482

[36] Harper JW, Elledge SJ, Keyomarsi K, Dynlacht B, Tsai LH, et al (1995) Inhibition of cyclin-dependent kinases by p21. Mol Biol Cell 6: 387–400. 7626805 10.1091/mbc.6.4.387 PMC301199

[37] Charrier-Savournin FB, Chateau MT, Gire V, Sedivy J, Piette J, et al (2004) p21-Mediated nuclear retention of cyclin B1-Cdk1 in response to genotoxic stress. Mol Biol Cell 15: 3965–3976. 15181148 10.1091/mbc.E03-12-0871 PMC515331

[38] Smits VA, Klompmaker R, Vallenius T, Rijksen G, Makela TP, et al (2000) p21 inhibits Thr161 phosphorylation of Cdc2 to enforce the G2 DNA damage checkpoint. J Biol Chem 275: 30638–30643. 10913154 10.1074/jbc.M005437200

[39] Cui W, Yazlovitskaya EM, Mayo MS, Pelling JC, Persons DL (2000) Cisplatin-induced response of c-jun N-terminal kinase 1 and extracellular signal–regulated protein kinases 1 and 2 in a series of cisplatin-resistant ovarian carcinoma cell lines. Mol Carcinog 29: 219–228. 11170260

[40] Dent P, Yacoub A, Fisher PB, Hagan MP, Grant S (2003) MAPK pathways in radiation responses. Oncogene 22: 5885–5896. 12947395 10.1038/sj.onc.1206701

[41] Wang X, Martindale JL, Holbrook NJ (2000) Requirement for ERK activation in cisplatin-induced apoptosis. J Biol Chem 275: 39435–39443. 10993883 10.1074/jbc.M004583200

[42] Pumiglia KM, Decker SJ (1997) Cell cycle arrest mediated by the MEK/mitogen-activated protein kinase pathway. Proc Natl Acad Sci U S A 94: 448–452. 9012803 10.1073/pnas.94.2.448 PMC19532

[43] Reinhardt HC, Aslanian AS, Lees JA, Yaffe MB (2007) p53-Deficient Cells Rely on ATM- and ATR-Mediated Checkpoint Signaling through the p38MAPK/MK2 Pathway for Survival after DNA Damage. Cancer Cell 11: 175–189. 17292828 10.1016/j.ccr.2006.11.024 PMC2742175

[44] Tang D, Wu D, Hirao A, Lahti JM, Liu L, et al (2002) ERK activation mediates cell cycle arrest and apoptosis after DNA damage independently of p53. J Biol Chem 277: 12710–12717. 11821415 10.1074/jbc.M111598200

[45] Favata MF, Horiuchi KY, Manos EJ, Daulerio AJ, Stradley DA, et al (1998) Identification of a novel inhibitor of mitogen-activated protein kinase kinase. J Biol Chem 273: 18623–18632. 9660836 10.1074/jbc.273.29.18623

[46] Foster SA, Demers GW, Etscheid BG, Galloway DA (1994) The ability of human papillomavirus E6 proteins to target p53 for degradation in vivo correlates with their ability to abrogate actinomycin D-induced growth arrest. J Virol 68: 5698–5705. 8057451 10.1128/jvi.68.9.5698-5705.1994 PMC236972

[47] Yan Y, Black CP, Cao PT, Haferbier JL, Kolb RH, et al (2008) Gamma-irradiation-induced DNA damage checkpoint activation involves feedback regulation between extracellular signal-regulated kinase 1/2 and BRCA1. Cancer Res 68: 5113–5121. 18593910 10.1158/0008-5472.CAN-07-5818 PMC3674789

[48] Nigro JM, Baker SJ, Preisinger AC, Jessup JM, Hosteller R, et al (1989) Mutations in the p53 gene occur in diverse human tumour types. Nature 342: 705–708. 2531845 10.1038/342705a0

[49] Willis A, Jung EJ, Wakefield T, Chen X (2004) Mutant p53 exerts a dominant negative effect by preventing wild-type p53 from binding to the promoter of its target genes. Oncogene 23: 2330–2338. 14743206 10.1038/sj.onc.1207396

[50] Yan Y, Shay JW, Wright WE, Mumby MC (1997) Inhibition of protein phosphatase activity induces p53-dependent apoptosis in the absence of p53 transactivation. J Biol Chem 272: 15220–15226. 9182545 10.1074/jbc.272.24.15220

[51] Yan Y, Spieker RS, Kim M, Stoeger SM, Cowan KH (2005) BRCA1-mediated G2/M cell cycle arrest requires ERK1/2 kinase activation. Oncogene 24: 3285–3296. 15735702 10.1038/sj.onc.1208492

[52] Gatei M, Scott SP, Filippovitch I, Soronika N, Lavin MF, et al (2000) Role for ATM in DNA damage-induced phosphorylation of BRCA1. Cancer Res 60: 3299–3304. 10866324

[53] Canman CE, Lim DS, Cimprich KA, Taya Y, Tamai K, et al (1998) Activation of the ATM kinase by ionizing radiation and phosphorylation of p53. Science 281: 1677–1679. 9733515 10.1126/science.281.5383.1677

[54] Sarkaria JN, Busby EC, Tibbetts RS, Roos P, Taya Y, et al (1999) Inhibition of ATM and ATR kinase activities by the radiosensitizing agent, caffeine. Cancer Res 59: 4375–4382. 10485486

[55] Hall-Jackson CA, Cross DA, Morrice N, Smythe C (1999) ATR is a caffeine-sensitive, DNA-activated protein kinase with a substrate specificity distinct from DNA-PK. Oncogene 18: 6707–6713. 10597277 10.1038/sj.onc.1203077

[56] Yarden RI, Pardo-Reoyo S, Sgagias M, Cowan KH, Brody LC (2002) BRCA1 regulates the G2/M checkpoint by activating Chk1 kinase upon DNA damage. Nat Genet 30: 285–289. 11836499 10.1038/ng837

[57] Yu Q, La Rose J, Zhang H, Takemura H, Kohn KW, et al (2002) UCN-01 inhibits p53 up-regulation and abrogates gamma-radiation-induced G(2)-M checkpoint independently of p53 by targeting both of the checkpoint kinases, Chk2 and Chk1. Cancer Res 62: 5743–5748. 12384533

[58] McGowan CH, Russell P (1993) Human Wee1 kinase inhibits cell division by phosphorylating p34cdc2 exclusively on Tyr15. Embo J 12: 75–85. 8428596 10.1002/j.1460-2075.1993.tb05633.x PMC413177

[59] McGowan CH, Russell P (1995) Cell cycle regulation of human WEE1. Embo J 14: 2166–2175. 7774574 10.1002/j.1460-2075.1995.tb07210.x PMC398322

[60] Yan Y, Mumby MC (1999) Distinct roles for PP1 and PP2A in phosphorylation of the retinoblastoma protein. PP2a regulates the activities of G(1) cyclin-dependent kinases. J Biol Chem 274: 31917–31924. 10542219 10.1074/jbc.274.45.31917

[61] Wu W, Sato K, Koike A, Nishikawa H, Koizumi H, et al (2010) HERC2 Is an E3 Ligase That Targets BRCA1 for Degradation. Cancer Research 70: 6384–6392. 20631078 10.1158/0008-5472.CAN-10-1304

[62] Yan Y, Haas JP, Kim M, Sgagias MK, Cowan KH (2002) BRCA1-induced apoptosis involves inactivation of ERK1/2 activities. J Biol Chem 277: 33422–33430. 12082091 10.1074/jbc.M201147200

[63] Wang X, Gorospe M, Huang Y, Holbrook NJ (1997) p27Kip1 overexpression causes apoptotic death of mammalian cells. Oncogene 15: 2991–2997. 9416843 10.1038/sj.onc.1201450

[64] Reinhardt HC, Yaffe MB (2009) Kinases that control the cell cycle in response to DNA damage: Chk1, Chk2, and MK2. Current Opinion in Cell Biology 21: 245–255. 19230643 10.1016/j.ceb.2009.01.018 PMC2699687

[65] Xu B, Kim S, Kastan MB (2001) Involvement of Brca1 in S-phase and G(2)-phase checkpoints after ionizing irradiation. Mol Cell Biol 21: 3445–3450. 11313470 10.1128/MCB.21.10.3445-3450.2001 PMC100266

[66] Abraham RT (2001) Cell cycle checkpoint signaling through the ATM and ATR kinases. Genes Dev 15: 2177–2196. 11544175 10.1101/gad.914401

[67] Lacroix M, Toillon R-A, Leclercq G (2006) p53 and breast cancer, an update. Endocrine-Related Cancer 13: 293–325. 16728565 10.1677/erc.1.01172

[68] Andres JL, Fan S, Turkel GJ, Wang JA, Twu NF, et al (1998) Regulation of BRCA1 and BRCA2 expression in human breast cancer cells by DNA-damaging agents. Oncogene 16: 2229–2241. 9619832 10.1038/sj.onc.1201752

[69] Lazebnik YA, Kaufmann SH, Desnoyers S, Poirier GG, Earnshaw WC (1994) Cleavage of poly(ADP-ribose) polymerase by a proteinase with properties like ICE. Nature 371: 346–347. 8090205 10.1038/371346a0

[70] Liu WH, Chang LS (2011) Fas/FasL-dependent and -independent activation of caspase-8 in doxorubicin-treated human breast cancer MCF-7 cells: ADAM10 down-regulation activates Fas/FasL signaling pathway. Int J Biochem Cell Biol 43: 1708–1719. 21854868 10.1016/j.biocel.2011.08.004

[71] Iliakis G, Wang Y, Guan J, Wang H (2003) DNA damage checkpoint control in cells exposed to ionizing radiation. Oncogene 22: 5834–5847. 12947390 10.1038/sj.onc.1206682

[72] Wei F, Xie Y, Tao L, Tang D (2010) Both ERK1 and ERK2 kinases promote G2/M arrest in etoposide-treated MCF7 cells by facilitating ATM activation. Cell Signal 22: 1783–1789. 20637859 10.1016/j.cellsig.2010.07.007

[73] Ho CC, Siu WY, Chow JP, Lau A, Arooz T, et al (2005) The relative contribution of CHK1 and CHK2 to Adriamycin-induced checkpoint. Exp Cell Res 304: 1–15. 15707569 10.1016/j.yexcr.2004.10.016

[74] O'Connor PM, Jackman J, Bae I, Myers TG, Fan S, et al (1997) Characterization of the p53 Tumor Suppressor Pathway in Cell Lines of the National Cancer Institute Anticancer Drug Screen and Correlations with the Growth-Inhibitory Potency of 123 Anticancer Agents. Cancer Research 57: 4285–4300. 9331090

[75] Brantley-Finley C, Lyle CS, Du L, Goodwin ME, Hall T, et al (2003) The JNK, ERK and p53 pathways play distinct roles in apoptosis mediated by the antitumor agents vinblastine, doxorubicin, and etoposide. Biochem Pharmacol 66: 459–469. 12907245 10.1016/s0006-2952(03)00255-7

[76] Liu SQ, Yu JP, Yu HG, Lv P, Chen HL (2006) Activation of Akt and ERK signalling pathways induced by etoposide confer chemoresistance in gastric cancer cells. Dig Liver Dis 38: 310–318. 16527552 10.1016/j.dld.2006.01.012

[77] Lee ER, Kim JY, Kang YJ, Ahn JY, Kim JH, et al (2006) Interplay between PI3K/Akt and MAPK signaling pathways in DNA-damaging drug-induced apoptosis. Biochim Biophys Acta 1763: 958–968. 16905201 10.1016/j.bbamcr.2006.06.006

[78] Navarro R, Busnadiego I, Ruiz-Larrea MB, Ruiz-Sanz JI (2006) Superoxide Anions Are Involved in Doxorubicin-Induced ERK Activation in Hepatocyte Cultures. Annals of the New York Academy of Sciences 1090: 419–428. 17384286 10.1196/annals.1378.045

[79] Gewirtz DA (2000) Growth arrest and cell death in the breast tumor cell in response to ionizing radiation and chemotherapeutic agents which induce DNA damage. Breast Cancer Research and Treatment 62: 223–235. 11072787 10.1023/a:1006414422919

[80] Chang BD, Xuan Y, Broude EV, Zhu H, Schott B, et al (1999) Role of p53 and p21waf1/cip1 in senescence-like terminal proliferation arrest induced in human tumor cells by chemotherapeutic drugs. Oncogene 18: 4808–4818. 10490814 10.1038/sj.onc.1203078

