# Correction: ERK1/2 Signaling Plays an Important Role in Topoisomerase II Poison-Induced G2/M Checkpoint Activation

**DOI:** 10.1371/journal.pone.0292423

**Published:** 2023-09-28

**Authors:** Ryan H. Kolb, Patrick M. Greer, Phu T. Cao, Kenneth H. Cowan, Ying Yan

Following publication of this article [1], errors were identified affecting western blots in Figs 1, 2, 4, 6, 8, 11, and S3. During figure preparation some lanes were removed from the original blots when preparing the figures, but this was not clearly indicated in the figures, and some figures were assembled using the incorrect blot images. With this notice, the authors provide updated Figs 1, 2, 4, 6, 8, 11, and S3 in which the issues have been addressed using the correct data from the original experiments. Detailed descriptions of the concerns identified for each figure are provided below.

The available underlying data for Figs 1, 2, 6, 8, 11, and S3 are provided in Supporting Information Files S1–S6. Annotations in several Supporting Information files indicate which lanes are included in the figures. For the Fig 6A ETOP ATR activity panel, 6B ATR activity panel, and 6C DOX Chk2 activity panel, only the cropped images are available; however, the authors’ expectation is that activity would be detected only within the cropped region because the activity assays detect purified ^32^P-labeled proteins as a single band using a Phosphorimager.

The data were reviewed by a member of the *PLOS ONE* Editorial Board who advised that the blots appear to support the reported results in the article. Underlying data for the rest of the figures in the article were not provided for editorial evaluation; however, they are available on request from the authors.

## Detailed concerns about the original published figures

The following issues are clarified by the original data and corrected in the updated figures provided with this notice. See the Supporting Information Files and updated Figures.

### Fig 1B

In the originally published Fig 1B, in the left-hand DOX pERK1/2 and ERK 1/2 panels, an additional lane for time point 0.25 hr was removed between lanes 1 and 2. In the right-hand ETOP pERK 1/2 panel, the 2 hr band was removed in error, and lane 4 incorrectly shows the 3 hr band. Here the authors provide a corrected Fig 1.

**Fig 1:**
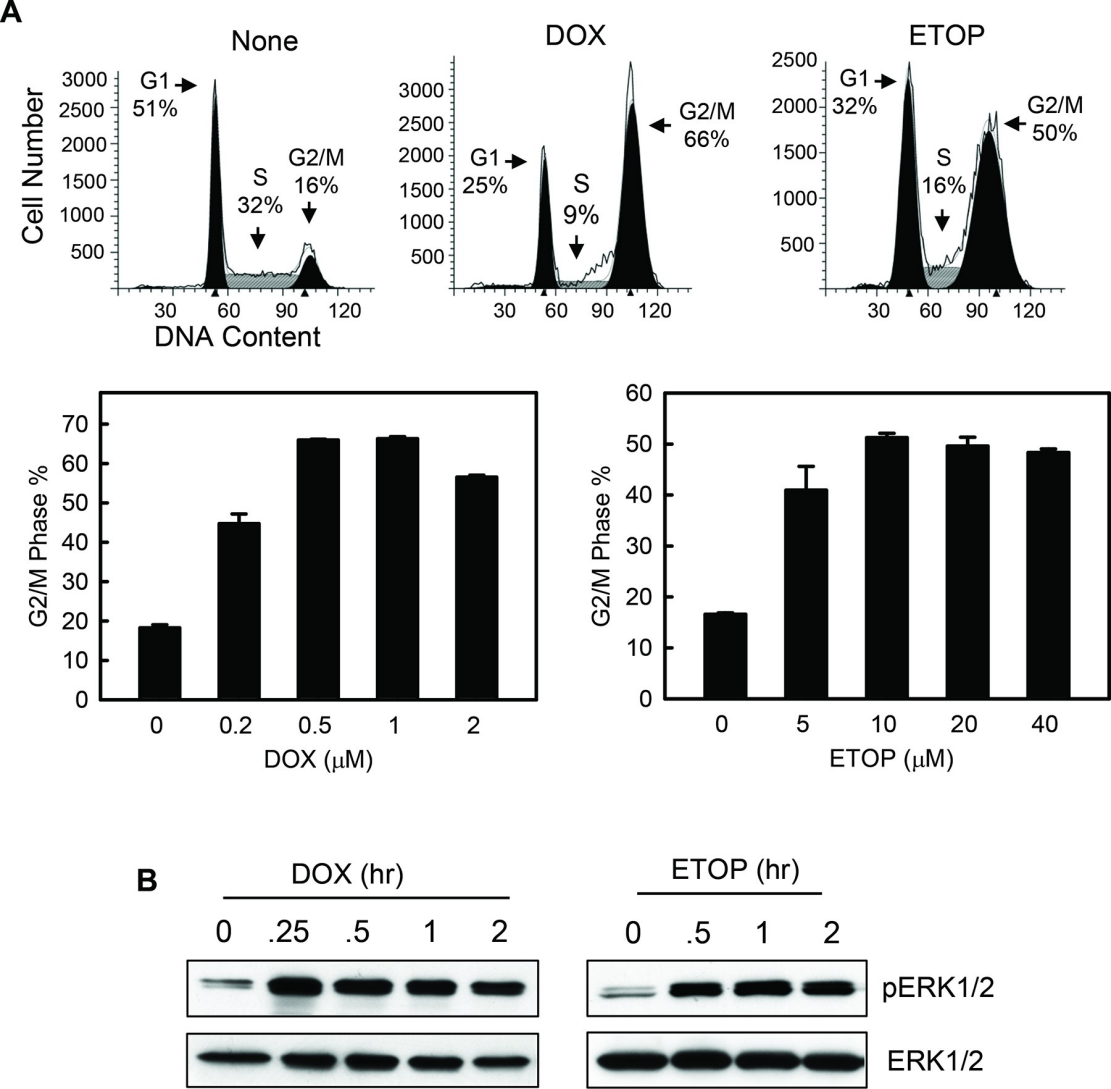
DOX and ETOP induce G2/M arrest and ERK1/2 activation in MCF-7 breast cancer cells. (A) Log-phase MCF-7 cells were treated with DOX or ETOP at the indicated doses as described in *Materials and Methods* and incubated for 24 hr. The cells were analyzed for DNA content by FACS. Upper panel: histograms shown are cells treated with none, 1 µM DOX or 10 µM ETOP. Cell cycle phases are indicated. Lower panel: Graphs depict the percentage of cells with 4*N*-DNA content, indicative of G2/M phase of the cell cycle, and represent the mean ± s.d. of two sets of experiment with duplicate samples. (B) MCF-7 cells were incubated in the presence of 0.5 µM DOX or 10 µM ETOP for the hours indicated and analyzed for phospho-ERK1/2 and total-ERK1/2 by immunoblotting.

### Fig 2

In the originally published Fig 2, in the DOX panels of Fig 2A, lanes were removed from the original blot images; however, in each panel, the incorrect lane was removed, resulting in errors in lane labelling.

In all panels of Fig 2B, a middle lane (the 1h time point) was removed, but the splice line was not marked between the first and third lanes (0h and 2h time points). Additionally, the ETOP Cdc2 panel is incorrect and appears to be a duplicate of lanes 2–3 of the Fig 6D DOX Cdc2 panel.

Here the authors provide a corrected Fig 2 which now includes all three lanes (0h, 1h, and 2h time points) presented on the original blots.

**Fig 2:**
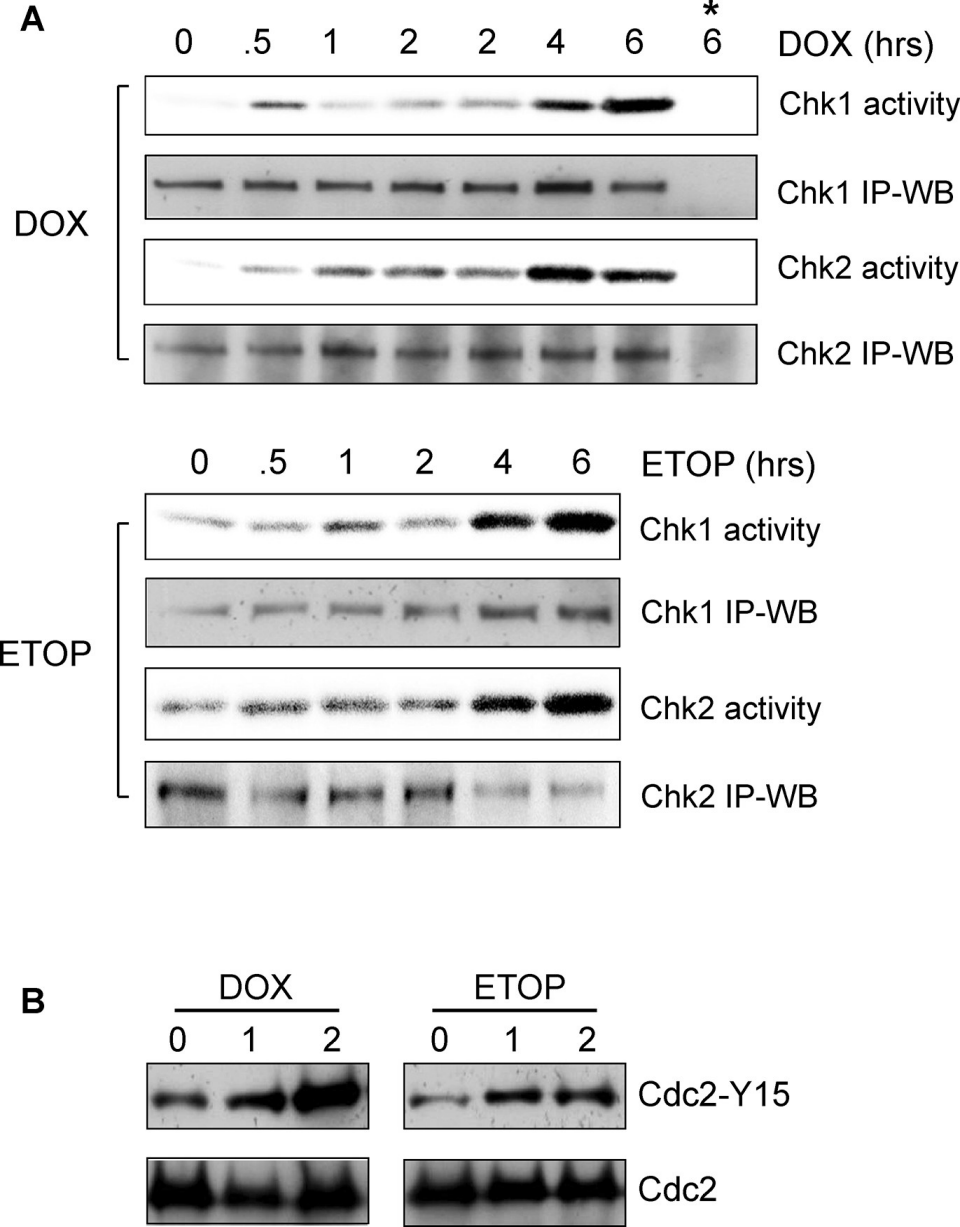
DOX and ETOP induce activation of Chk1 and Chk2 kinases and inhibition of Cdc2 kinase. (A) MCF-7 Cells were incubated with 1 µM DOX (upper panel) or 10 µM ETOP (lower panel) for the indicated times for up to 2 hr. For the 4 hr and 6 hr time points, the cells were incubated for 2 hr with DOX or ETOP, washed with DMEM and incubated for additional 2 hr and 4 hr, respectively, in regular culture medium. Following treatment, Chk1 and Chk2 kinases were respectively immunoprecipitated from cell lysates and examined for kinase activity as described in *Materials and Methods* (*Chk1 Activity* and *Chk2 Activity*). Levels of Chk1 and Chk2 in the immunoprecipitates were determined by immunoblotting (*Chk1 IP-WB* and *Chk2 IP-WB*). *, as a negative control, kinase assay was carried out using immunoprecipitates obtained by incubating DOX-treated cell sample (6 hr time point) with non-immunized IgG. (B) Cells were treated as described above and incubated for 0, 1 and 2 hr. Cdc2 was immunoprecipitated from cell lysate and analyzed for levels of Cdc2-Tyr15 phosphorylation by immunoblotting (*Cdc2-Tyr15*). As a control, Cdc2 in the immunoprecipitates was assessed by immunoblotting (*Cdc2*).

### Fig 4A

The western blot in the originally published Fig 4A is incorrect. Readers are referred to the revised version of Fig 6B for the correct results of this experiment. Here the authors provide a revised Fig 4 with the western blot panel of Fig 4A removed.

**Fig 4:**
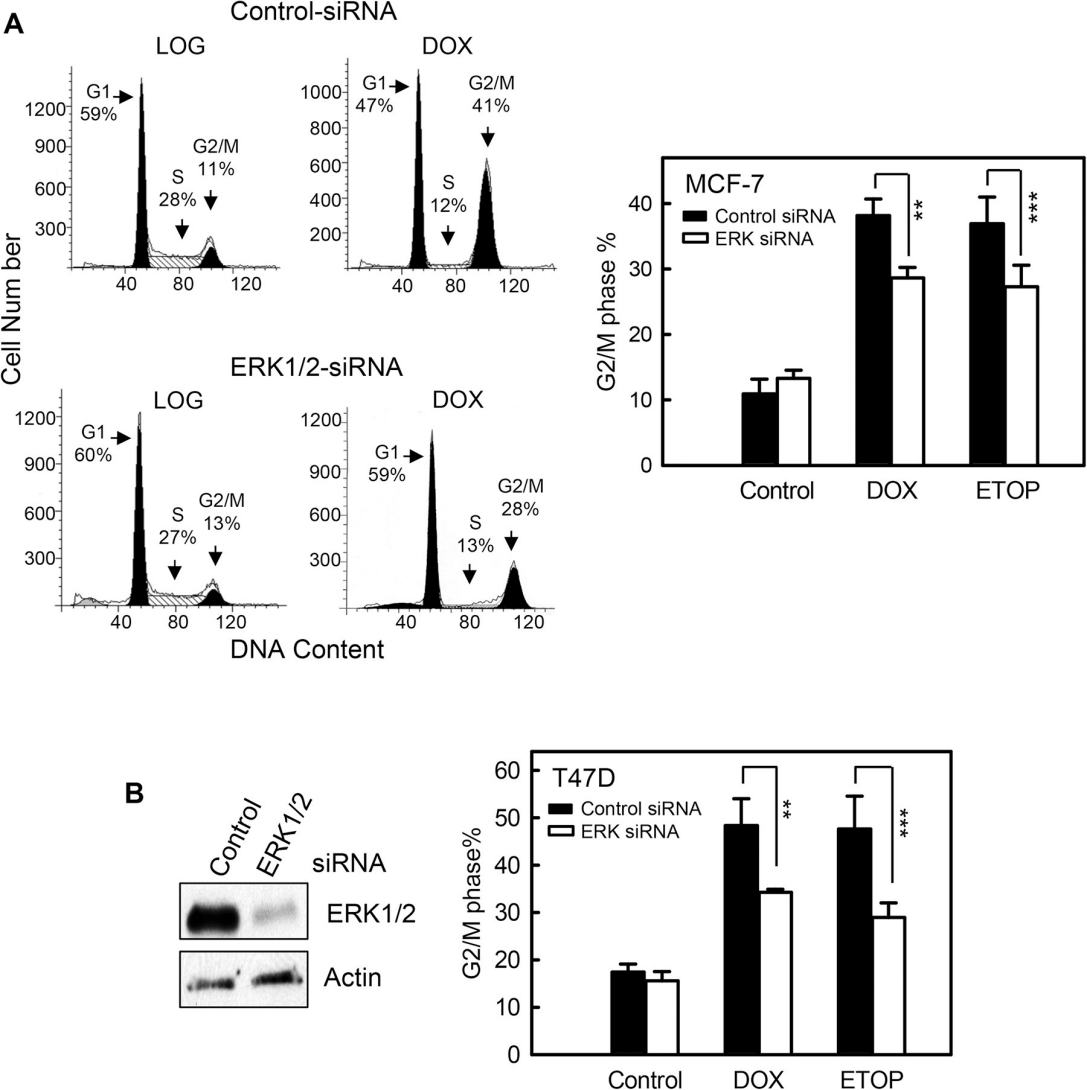
Inhibition of ERK1/2 by specific siRNA diminishes topo II poison-induced G2/M arrest. (A) MCF-7 cells were transfected with ERK1/2 specific siRNA or control non-targeting siRNA and incubated for 2 days. The cells were then treated with 0.5 µM DOX or 5 µM ETOP, incubated for 24 hr and analyzed for DNA content by FACS. Left panel: histograms shown are DNA content analyses for the indicated cell samples. Right panel: bar graph depicts the percentage of cells in G2/M phase and presented as mean ± s.d. of three independent experiments in duplicate. ** *p*<0.005 (n  =  6), *** *p*<0.01 (n  =  6), significant difference from cells transfected with control siRNA. (B) T47D cells were transfected with siRNA targeting ERK1/2 or control siRNA, incubated for 2 days and treated with 0.2 µM DOX or 5 µM ETOP. Left panel: levels of ERK1/2 in siRNA-transfected cells were determined by Western blotting. Right panel: the treated cell were incubated for additional 24 hr and analyzed for DNA content by FACS. Bar graph depicts the percentage of cells in G2/M phase and presented as mean ± s.d. of two independent experiments in duplicate. ** *p*<0.005 (n  =  4), *** *p*<0.01 (n  =  4), significant difference from cells transfected with control siRNA.

### Fig 6

In Fig 6B, the ATR IP-WB blot is incorrect and lanes 2–4 are similar to lanes 2–4 of panel ATR IP-WB in Fig 6A when flipped horizontally.

In Fig 6C, DOX ATM activity panel, lanes were removed between lanes 1 and 2 and between lanes 3 and 4 and the DOX Chk2 IP-WB blot is incorrect.

In Fig 6D ETOP Cdc2 IP-WB panel, the U0126 lanes are incorrect and in the Cdc2-Tyr15 panel a lane was removed between lanes 3 and 4.

Here the authors provide a corrected Fig 6.

**Fig 6:**
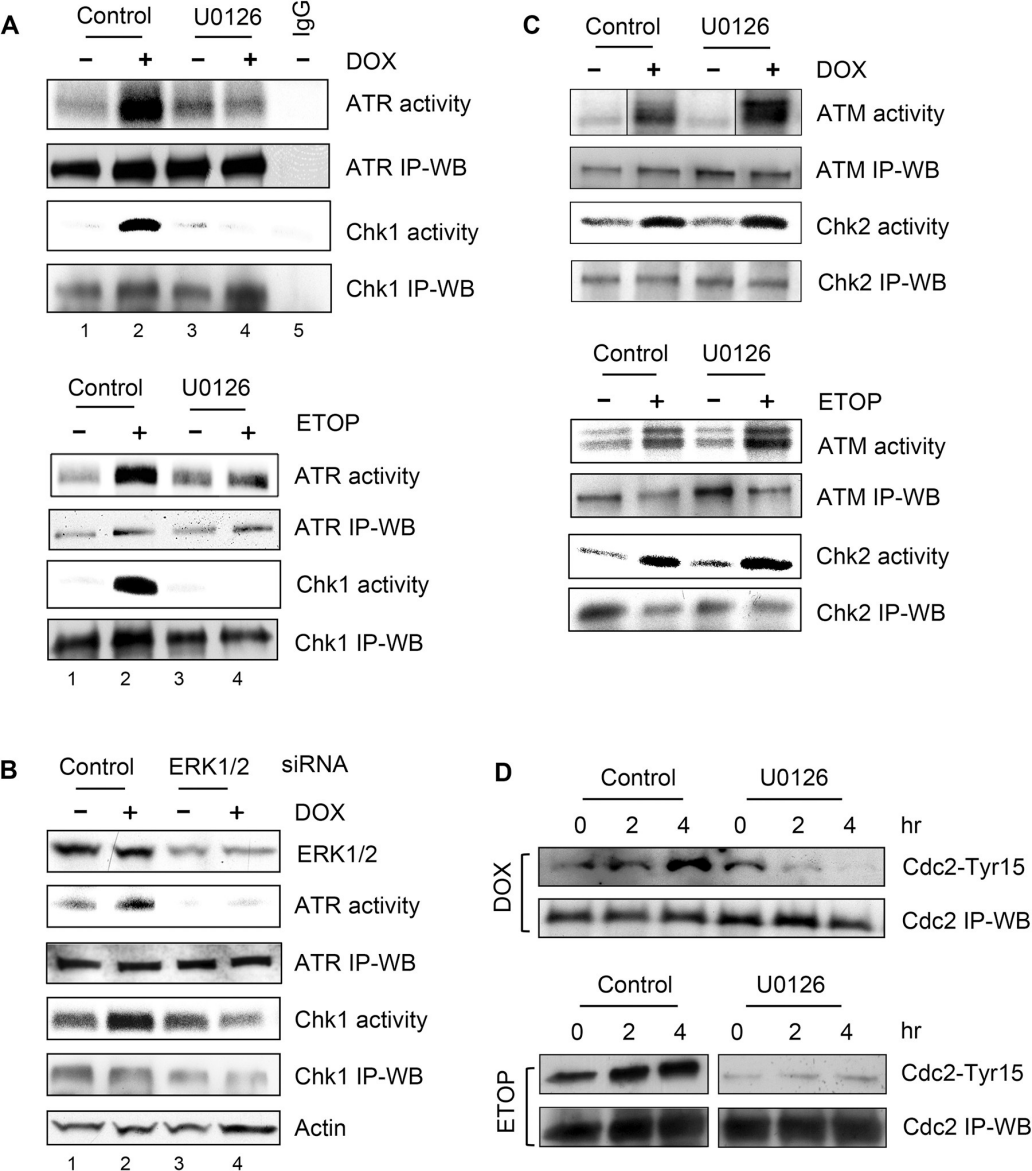
Effect of ERK1/2 inhibition on topo II poison-induced ATR and ATM signaling activation. (A) MCF-7 cells were treated for 2 hr with 1 µM DOX (upper panel) or 10 µM ETOP (lower panel) in the presence or absence of 50 µM U0126. The cells were washed and incubated in growth medium for additional 2 hr with the presence or absence of U0126. ATR and Chk1 kinase were respectively immunoprecipitated from the resulting cell lysates and assayed for kinase activity. ATR and Chk1 levels in immunoprecipitates were determined by immunoblotting (*ATR IP-WB* and *Chk1 IP-WB*). *IgG*, as a negative control, kinase assay was carried out using immunoprecipitates obtained by incubating control untreated cell lysate with non-immunized IgG. (B) Cells transfected with ERK1/2 specific or control siRNA were incubated for 2 days and treated with or without 0.5 µM DOX, as described above. ATR and Chk1 were respectively immunoprecipitated from the cell lysates and examined for kinase activity (*ATR activity* and *Chk1 activity*). ATR and Chk1 protein levels in immunoprecipitates were determined by immunoblotting (*ATR IP-WB* and *Chk1 IP-WB*). Levels of ERK1/2 and Actin in cell lysates were analyzed by immunoblotting (*ERK1/2* and *Actin*). (C) Cells were treated as described in (A). ATM and Chk2 were immunoprecipitated from the cell lysates and assayed for kinase activity (*ATM activity* and *Chk2 activity*). ATM and Chk2 levels in immunoprecipitates were determined by immunoblotting (*ATM IP-WB* and *Chk2 IP-WB*). Removed lanes are marked by black lines in Fig 6C. All the lanes of the ATM activity panel are from the same exposure of the same gel. D) MCF-7 cells were treated as described in (A) and incubated for the times indicated. Cdc2 was immunoprecipitated from cell lysate and analyzed for Cdc2-Tyr15 phosphorylation by immunoblotting (*Cdc2-Tyr15*). Cdc2 in the immunoprecipitates was quantified by immunoblotting (*Cdc-2 IP-WB*). Different parts of the gels are shown in separate, adjacent panels in 6D.

### Fig 8

In the DOX and ETOP ERK 1/2 panels, lanes were removed to simplify the presentation. Additionally, in the ETOP ERK 1/2 panel, lanes 1–3 are incorrect and overlap with lanes 3 and 4 of the DOX ERK 1/2 panel, having been taken from the wrong part of the blot. Here the authors provide a corrected Fig 8, which contains all lanes on the original blots.

**Fig 8:**
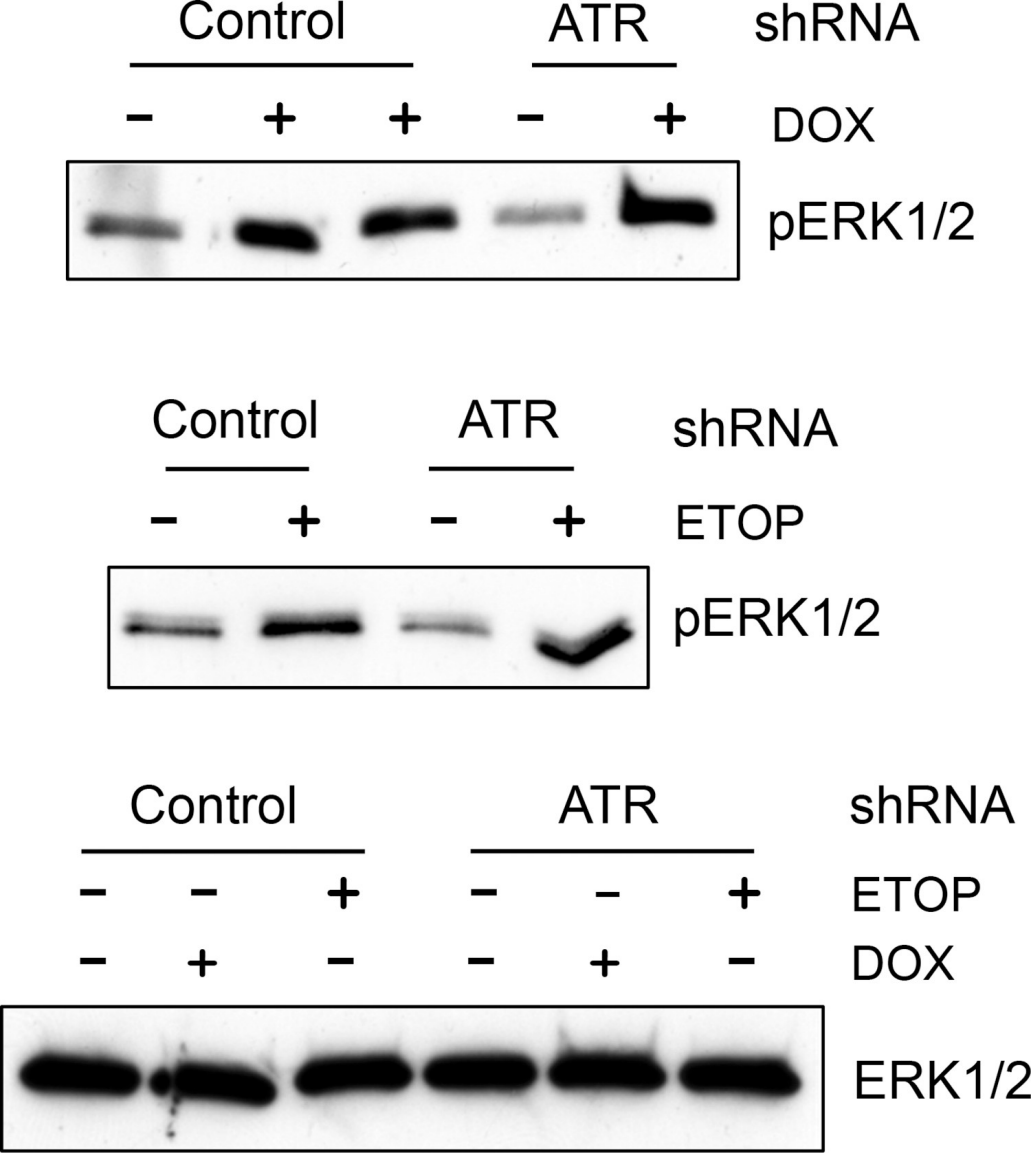
Decrease of ATR level by shRNA had no effect on DOX-induced ERK1/2 activation. MCF-7 cells expressing ATR specific or control shRNA were treated with or without 1 µM DOX (upper panel) or 10 µM ETOP (lower panel) for 2 hr and analyzed for phospho-ERK1/2 (*pERK1/2*) and total ERK1/2 (*ERK1/2*) by immunoblotting.

### Fig 11

Some bands in the originally published figure are duplicated across panels intentionally because of the experimental design.

Specifically:

- For the Control Caspase 8 and DOX Caspase 8 panels lanes 1 and 2 were used for the Control Caspase 8 panel, while lanes 1, 3, and 4 of the same blot were used for the DOX Caspase 8 panel.
- The Control PARP and ETOP PARP panels are assembled from the same blot. Lanes 1 and 2 were used for the Control PARP panel, while lanes 1, 5, and 6 were used for the ETOP PARP panel.
- The Dox PARP panel was assembled from lanes 1, 3, and 4 of a different blot which was originally probed for PARP and then re-probed for Actin. The Control, DOX, and ETOP Actin panels are all assembled from this same blot. Lanes 1 and 2 were used for the Control Actin panel; lanes 1, 3, and 4 were used for the DOX Actin panel; and lanes 1, 5, and 6 were used for the ETOP Actin panel.

Actin was not used as a loading control in this experiment, but rather as an internal control that is expected to remain unaffected by the different treatments.

Here the authors provide a revised Fig 11 in which each group (Control, DOX, and ETOP) is presented as an individual panel with a box around it, and the duplicated control lanes have been removed.

**Fig 11:**
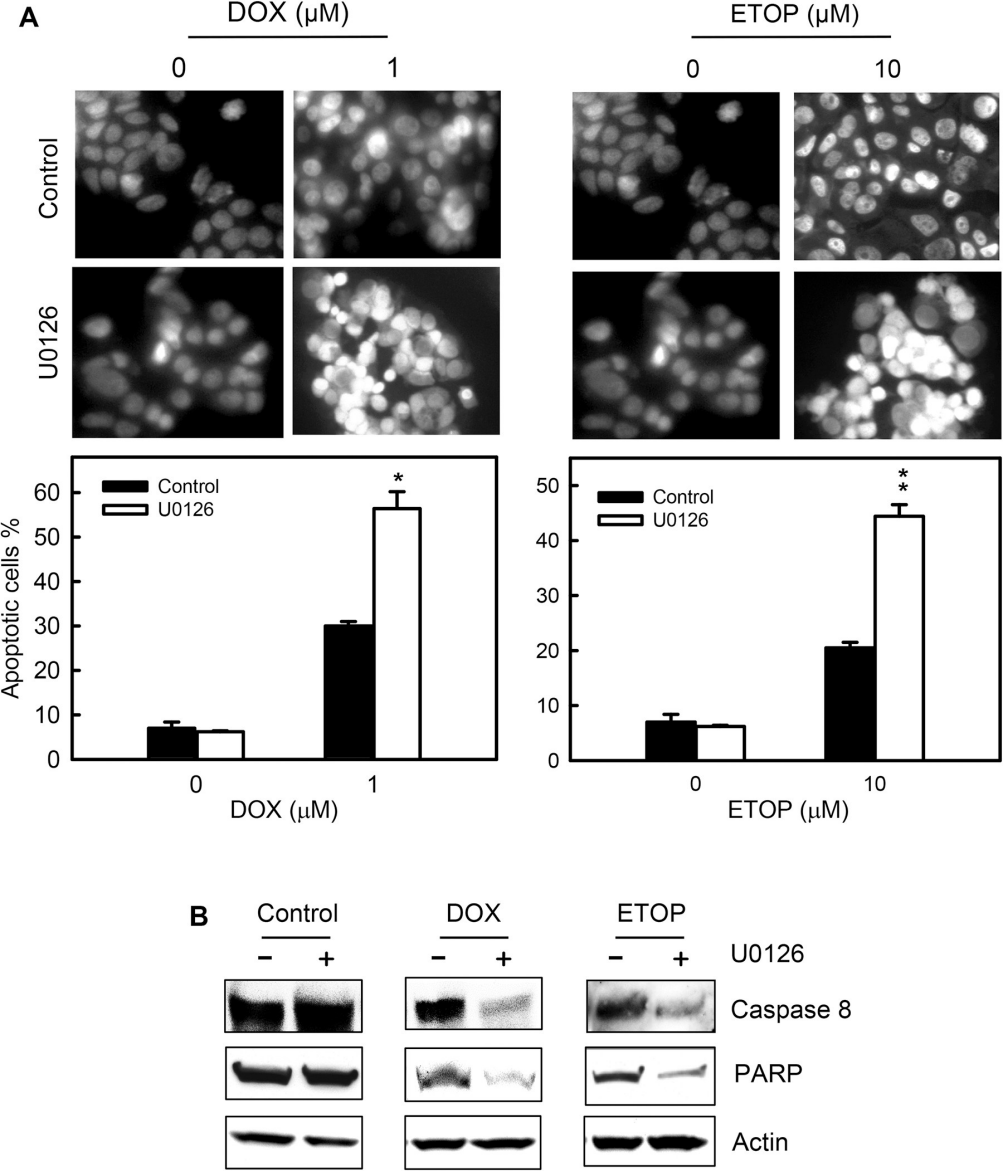
Inhibition of ERK1/2 signaling increases topo II poison-induced apoptosis. ( A) MCF-7 cells were treated for 2 hr with or without 1 µM DOX or 10 µM ETOP in the presence or absence of 50 µM U0126. Following treatment, the cells were washed, incubated for 3 days with/without presence of U0126 and analyzed for apoptosis by DAPI staining and fluorescence microcopy. Upper panels: images shown are the DAPI staining of the resulting cells. Lower panels: the percentage of apoptotic cells is shown as mean ± s.d of quadruplicate samples. *p<0.01 (n  =  4), significant difference from cells treated with DOX alone. **p<0.005 (n  =  4), significant difference from cells treated with ETOP alone. (B) The cells obtained above were analyzed for levels of full-length PARP and Caspase 8 by Western blot analysis. The protein loading were assessed by immunoblotting for Actin levels (*Actin*). Each experimental group (Control, DOX, and ETOP; +/- U0126) were cropped from the whole gel image and presented as individual panels in separate boxes.

### S3 Fig

Duplicates of lanes 1–2 of the DOX ERK 1/2 panel of Fig 6B and lanes 3–4 of the Actin panel of Fig 6B were reported in this figure in error. A corrected S3 Fig is provided in File S6. Additionally, the captions for S2 Fig and S3 Fig were incorrectly swapped during the publishing process.

### S2 Fig. Treatment with U0126 has no effect on the cell cycle profile of MCF-7 cells

MCF-7 cells were incubated in the presence or absence of U0126 for 24 hr and analyzed for DNA content by FACS. Histograms shown are DNA content analyses for the indicated cell samples.

### S3 Fig. Transfection of non-targeting control siRNA had no effect on DOX-induced G2/M cell cycle arrest in MCF-7 cells

MCF-7 cells were transfected with control non-targeting siRNA or left untransfected and incubated for 2 days. (A) Left panel: the cells were analyzed for protein levels of ERK1/2 and Actin by Western blotting. Right panel: Immunoblot densities of ERK1/2 and Actin were quantified using ImageJ software and relative ERK1/2 expression versus Actin determined. (B) The cells were then treated with 0.5 µM DOX, incubated for 24 hr and analyzed for DNA content by FACS. Histograms shown are DNA content analyses for the indicated cell samples.

## Supporting information

S1 File Fig 1 Underlying Data. (ZIP)

S2 File Fig 2 Underlying Data. Note that the underlying blot for Fig 2A ETOP Chk1 IP-WB may be a different exposure than that used for the figure.
(ZIP)

S3 File Fig 6 Underlying Data. Note that the underlying blots for Fig 6A ETOP ATR activity, 6B ATR activity, and 6C DOX Chk2 activity are available only as cropped images. The underlying 6C ETOP ATM activity blots are from a shorter exposure than that used for the figure.
(ZIP)

S4 File Fig 8 Underlying Data. (ZIP)

S5 File Fig 11 Underlying Data. The underlying blot for the image shown in the original figure for the Control Caspase 8 and DOX Caspase 8 panels is not available; however, the authors provide an image of a shorter exposure of the same blot. Lanes 1 and 2 were used for the Control Caspase 8 panel, while lanes 1, 3, and 4 of the same blot were used for the DOX Caspase 8 panel. Additional Actin replicates are provided for the experiment shown in Fig 11B. One blot was probed first for the DOX PARP panel, and then re-probed for Actin. The blot showing PARP bands alone is not available, but the PARP bands remain visible on the blot after re-probing for Actin.
(ZIP)

S6 File Revised Fig S3 and Underlying Data. (ZIP)
