# Supplementary figures and images for: ERK1/2 Signaling Plays an Important Role in Topoisomerase II Poison-Induced G2/M Checkpoint Activation
Source: PLoS One. 2012 Nov 16;7(11):e50281. doi: 10.1371/journal.pone.0050281 (PMC3500378; doi:10.1371/journal.pone.0050281)

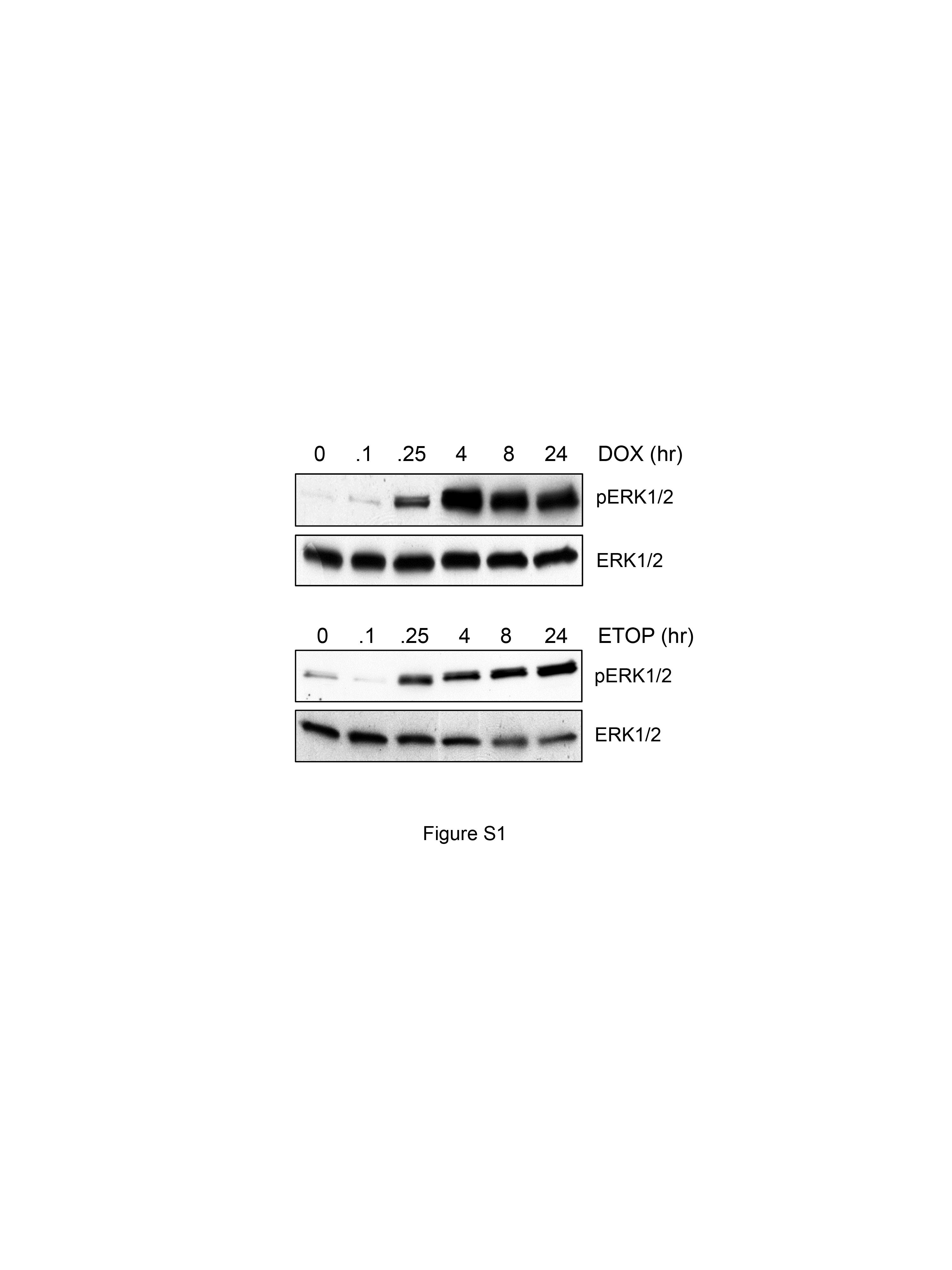

Supplement: Figure S1 — DOX and ETOP induce ERK1/2 activation in MCF-7 breast cancer cells. MCF-7 cells were incubated in the presence of 1 µM DOX or 10 µM ETOP for the hours indicated and analyzed for phospho-ERK1/2 and total-ERK1/2 by immunoblotting. (TIF) [file pone.0050281.s001.tif]

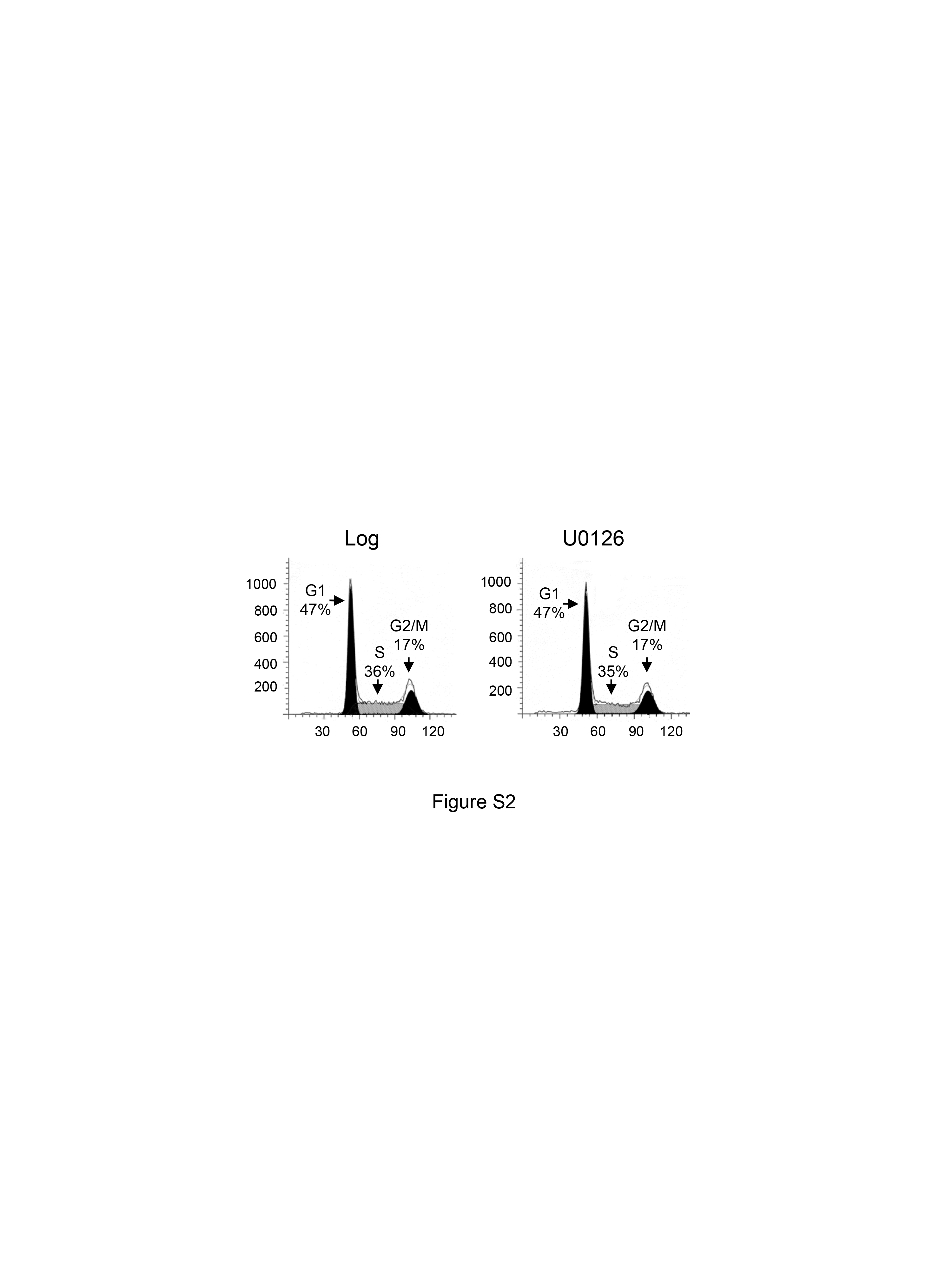

Supplement: Figure S2 — Transfection of non-targeting control siRNA had no effect on DOX-induced G2/M cell cycle arrest in MCF-7 cells. MCF-7 cells were transfected with control non-targeting siRNA or left untransfected and incubated for 2 days. (A) Left panel: the cells were analyzed for protein levels of ERK1/2 and Actin by Western blotting. Right panel: Immunoblot densities of ERK1/2 and Actin were quantified using ImageJ software and relative ERK1/2 expression versus Actin determined. (B) The cells were then treated with 0.5 µM DOX, incubated for 24 hr and analyzed for DNA content by FACS. Histograms shown are DNA content analyses for the indicated cell samples. (TIF) [file pone.0050281.s002.tif]

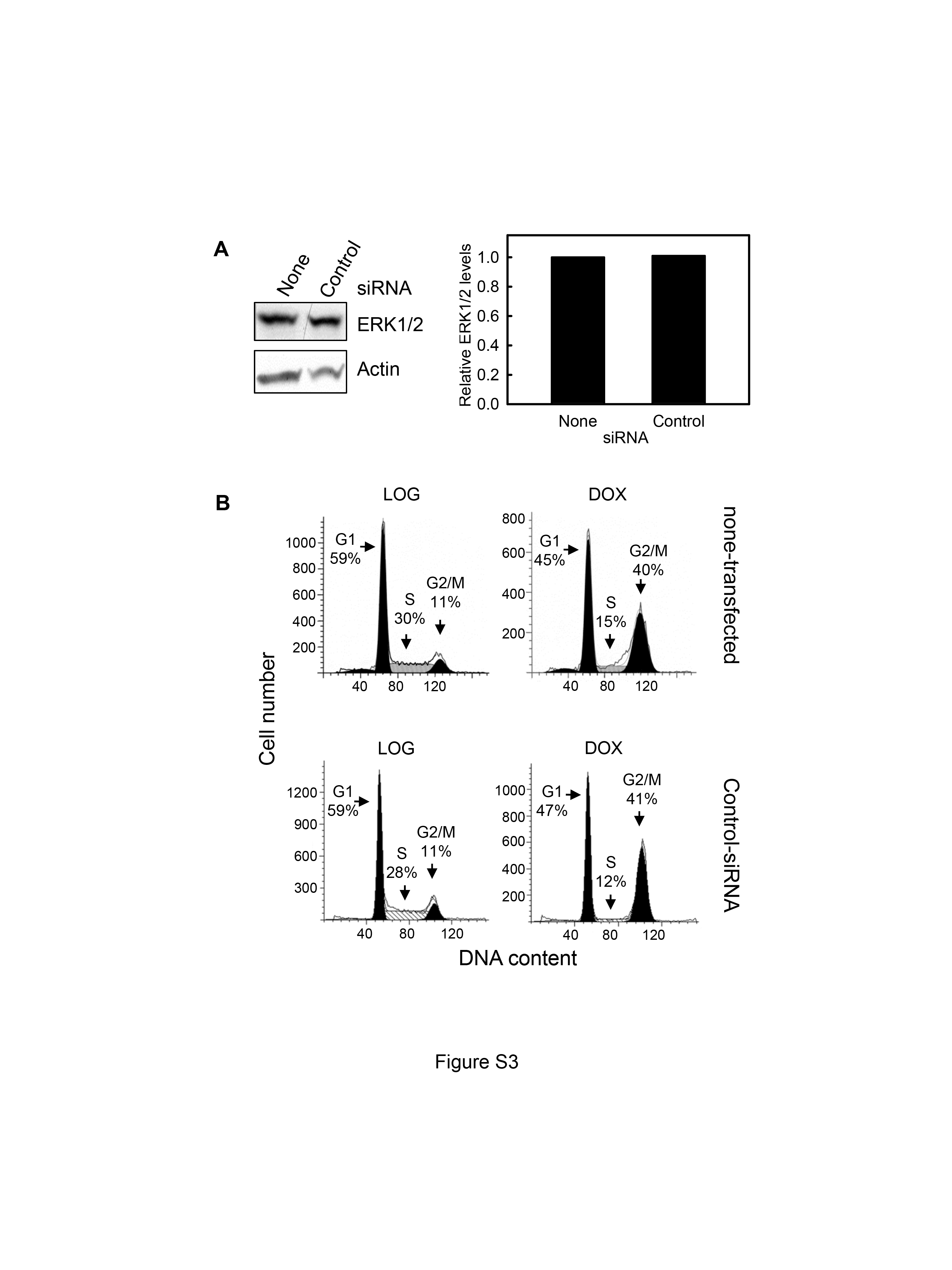

Supplement: Figure S3 — Treatment with U0126 has no effect on the cell cycle profile of MCF-7 cells. MCF-7 cells were incubated in the presence or absence of U0126 for 24 hr and analyzed for DNA content by FACS. Histograms shown are DNA content analyses for the indicated cell samples. (TIF) [file pone.0050281.s003.tif]

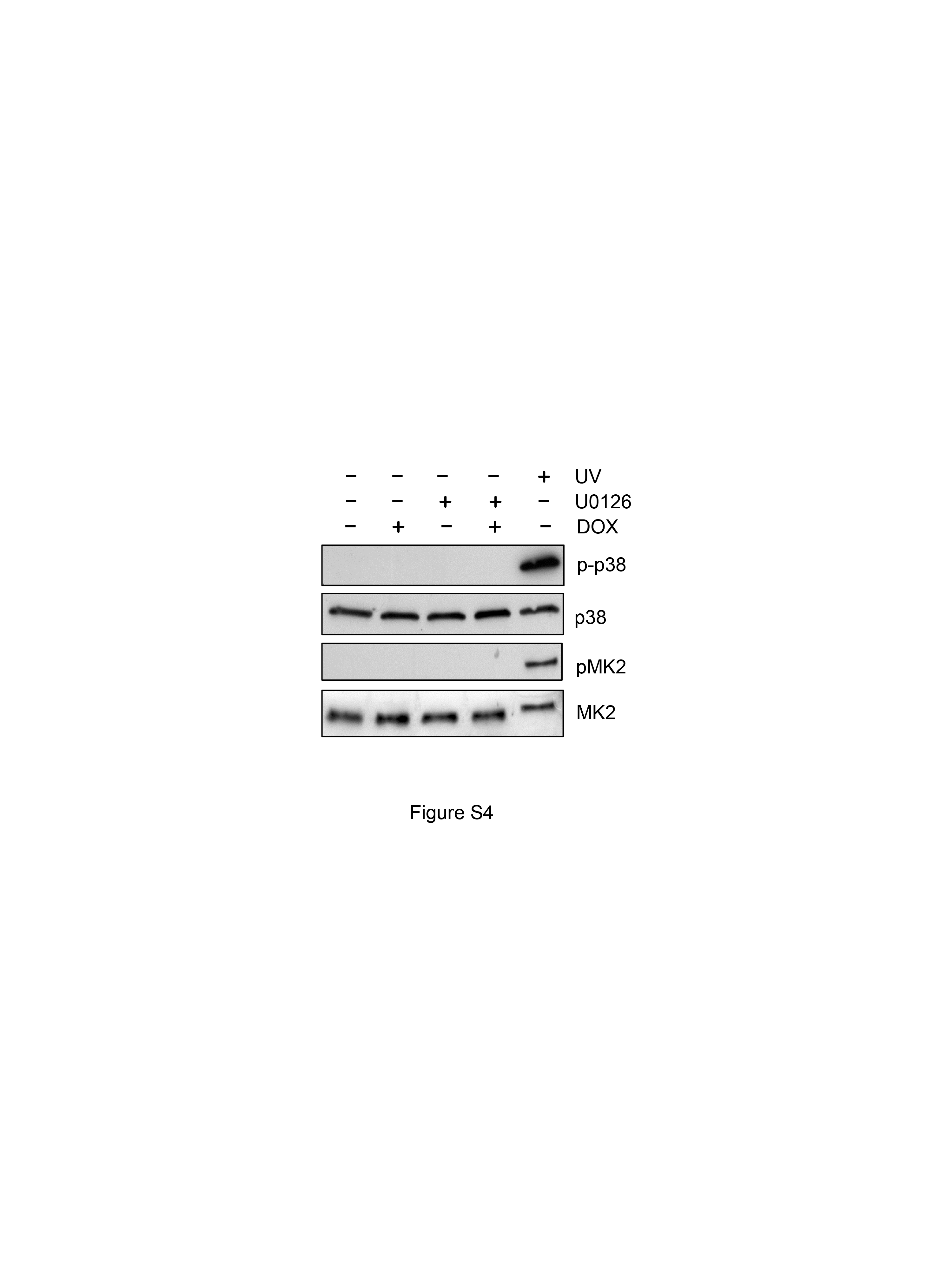

Supplement: Figure S4 — Treatment of T47D breast cancer cells with DOX does not activate p38 and MK2 kinases. T47D cells were treated for 2 hr with or without 1 μM DOX in the presence or absence of 50 μM U0126. As a positive control for p38 and MK2 activation, a cell sample was exposed to UV at 100 J/m2 and incubated for 1 hr at 37°C. The resulting cells were analyzed for levels of phospho-p38 (p-p38), total p38 (p38), phospho-MK2 (pMK2) and total MK2 (MK2) by immunoblotting. (TIF) [file pone.0050281.s004.tif]
