# Supplementary material for: Correction: ERK1/2 Signaling Plays an Important Role in Topoisomerase II Poison-Induced G2/M Checkpoint Activation
Source: PLoS One. 2023 Sep 28;18(9):e0292423. doi: 10.1371/journal.pone.0292423 (PMC10538782; doi:10.1371/journal.pone.0292423)
Supplement: S1 File — (ZIP) [file pone.0292423.s001.zip › Figure 1A/ADR-1 uM.pdf]

1 uM Adr, MCF7

SAMPLE ID: ADR 1.0 A

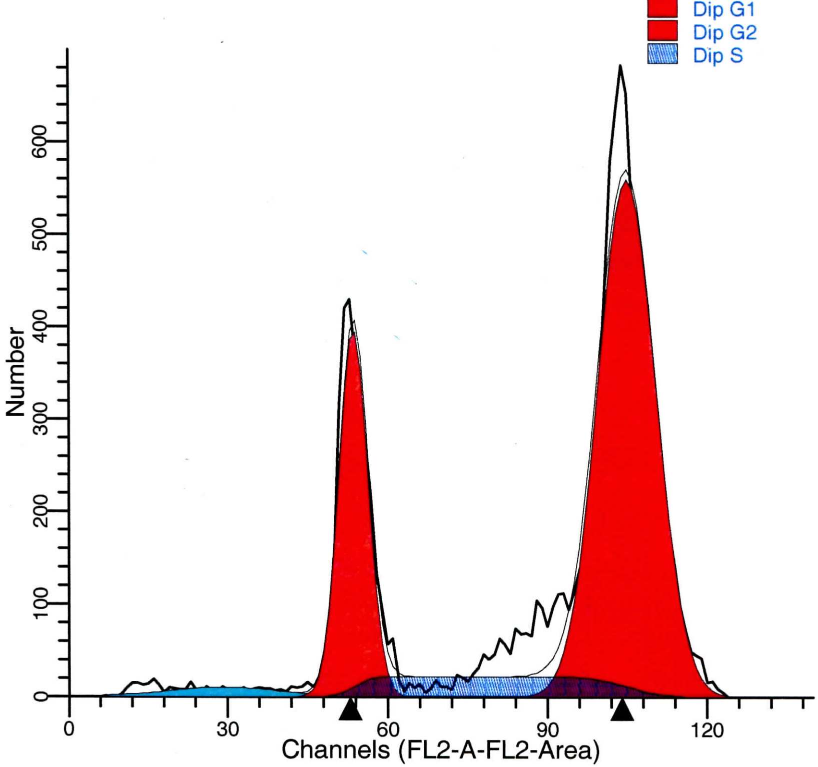

File analyzed: RK27U09.007
Date analyzed: 3-Sep-2009
Model: 1nn0A_DSF
Analysis type: Manual analysis

Diploid: 100.00 %
Dip G1: 24.69 % at 53.66
Dip G2: 66.34 % at 105.00
Dip S: 8.97 % G2/G1: 1.96
%CV: 5.05

Total S-Phase: 9.97 %
Total B.A.D.: 0.00 % no debris no aggs

Apoptosis: 2.40 % Mean: 29.38

Debris: %
Aggregates: 0.00 %
Modeled events: 11742
All cycle events: 11460
Cycle events per channel: 219
RCS: 7.670
