# Supplementary material for: Correction: ERK1/2 Signaling Plays an Important Role in Topoisomerase II Poison-Induced G2/M Checkpoint Activation
Source: PLoS One. 2023 Sep 28;18(9):e0292423. doi: 10.1371/journal.pone.0292423 (PMC10538782; doi:10.1371/journal.pone.0292423)
Supplement: S1 File — (ZIP) [file pone.0292423.s001.zip › Figure 1A/ADR-FACS, 6-26-2009.pdf]

SAMPLE ID: Log A

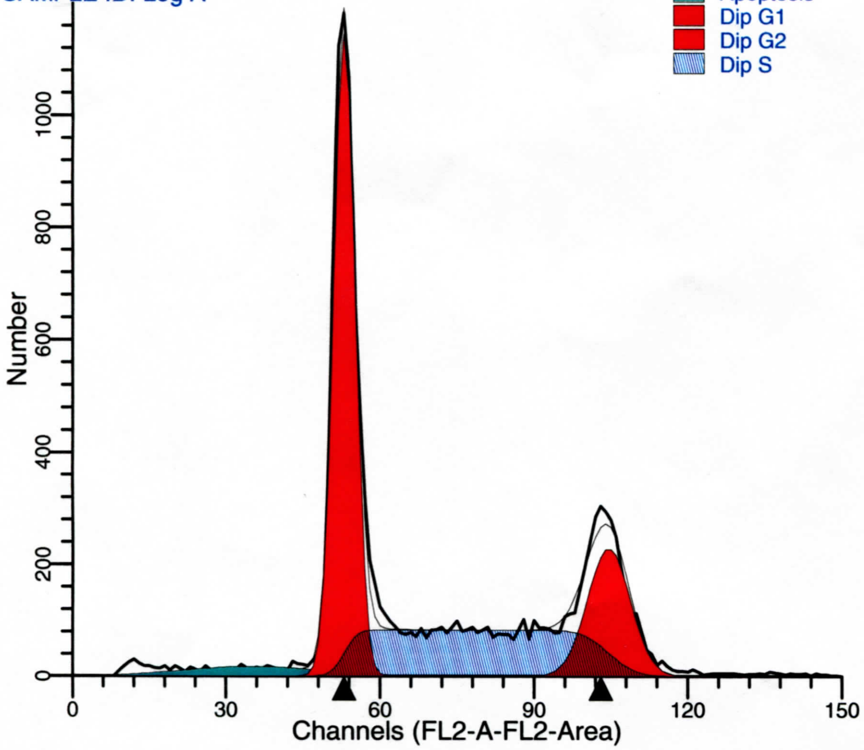


File analyzed: RK25U09.001
Date analyzed: 25-Jun-2009
Model: 1nn0A_DSF
Analysis type: Manual analysis

Diploid: 100.00 %
Dip G1: 47.37 % at 53.07
Dip G2: 18.83 % at 104.60
Dip S: 33.80 % G2/G1: 1.97
%CV: 3.90

Total S-Phase: 33.80 %
Total B.A.D.: 0.00 % no debris no aggs

Apoptosis: 4.33 % Mean: 35.10

Debris: %
Aggregates: 0.00 %
Modeled events: 13254
All cycle events: 12681
Cycle events per channel: 241
RCS: 4.022

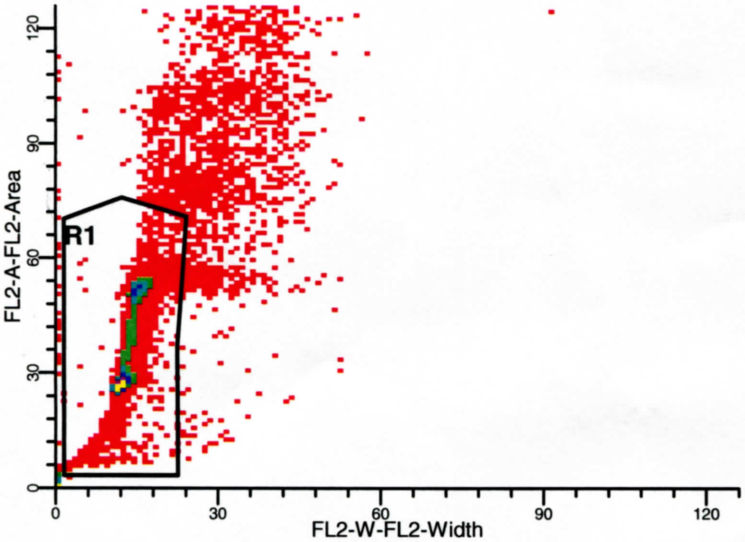

SAMPLE ID: Log B

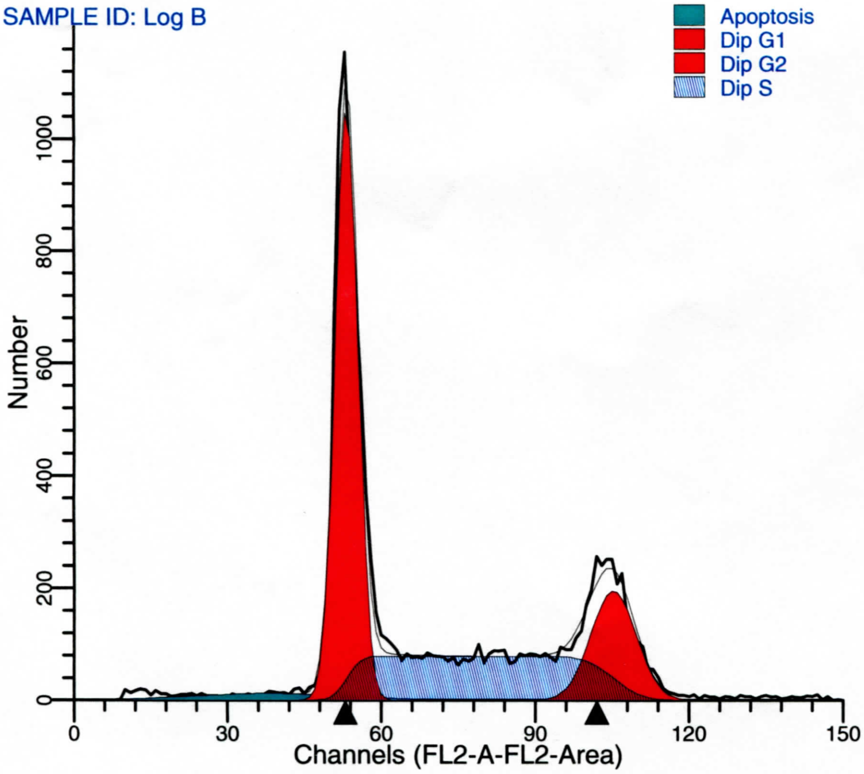


File analyzed: RK25U09.002
Date analyzed: 25-Jun-2009
Model: 1nn0A_DSF
Analysis type: Manual analysis

Diploid: 100.00 %
Dip G1: 48.39 % at 53.29
Dip G2: 17.77 % at 105.29
Dip S: 33.84 % G2/G1: 1.98
%CV: 4.13

Total S-Phase: 33.84 %
Total B.A.D.: 0.00 % no debris no aggs

Apoptosis: 3.72 % Mean: 38.17

Debris: %
Aggregates: 0.00 %
Modeled events: 12624
All cycle events: 12154
Cycle events per channel: 229
RCS: 3.703

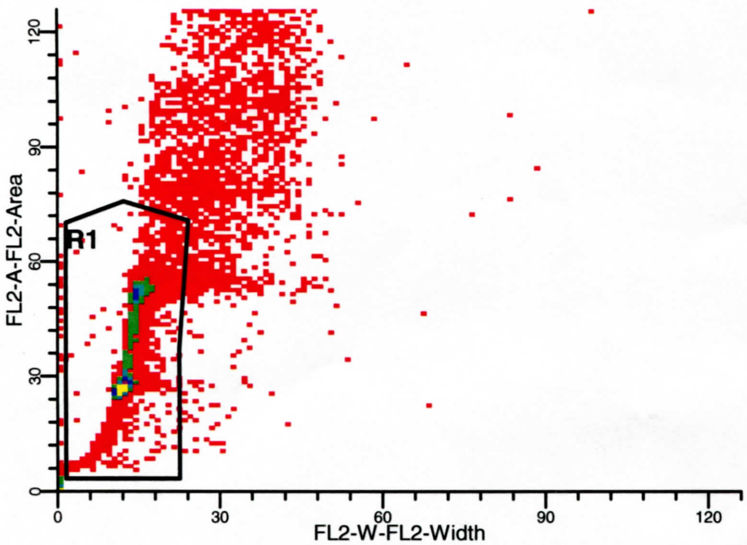


ModFitLT V3.0(Win32)

SAMPLE ID: ADR 0.2 A

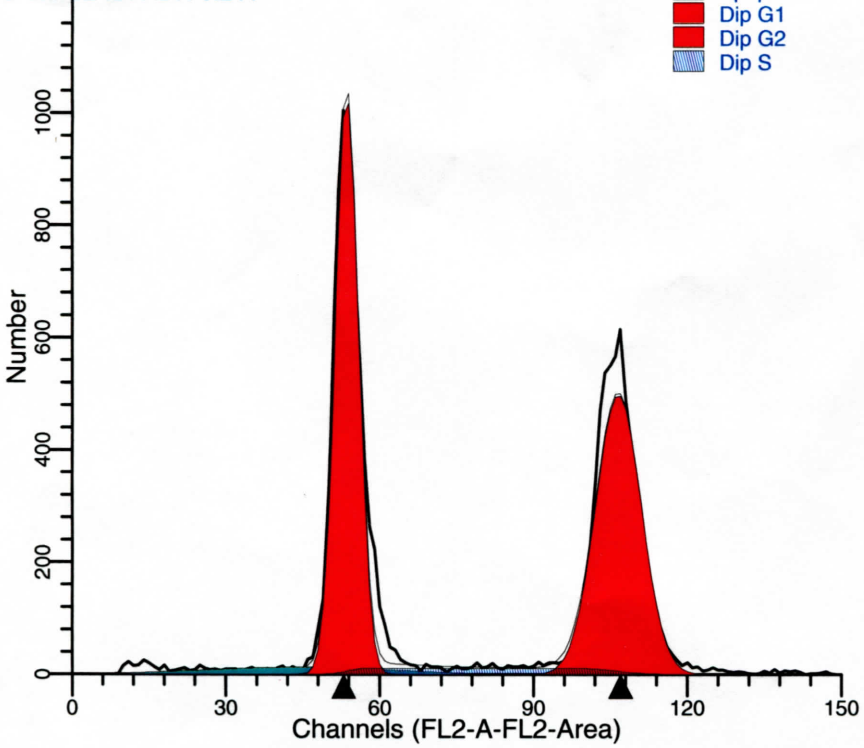


File analyzed: RK25U09.003
Date analyzed: 25-Jun-2009
Model: 1nn0A_DSF
Analysis type: Manual analysis

Diploid: 100.00 %
Dip G1: 48.43 % at 53.62
Dip G2: 46.48 % at 106.57
Dip S: 5.09 %   G2/G1: 1.99
%CV: 4.14

Total S-Phase: 5.09 %
Total B.A.D.: 0.00 %   no debris no aggs

Apoptosis: 4.62 %   Mean: 43.96

Debris:  %
Aggregates: 0.00 %
Modeled events: 12535
All cycle events: 11957
Cycle events per channel: 222
RCS: 6.816

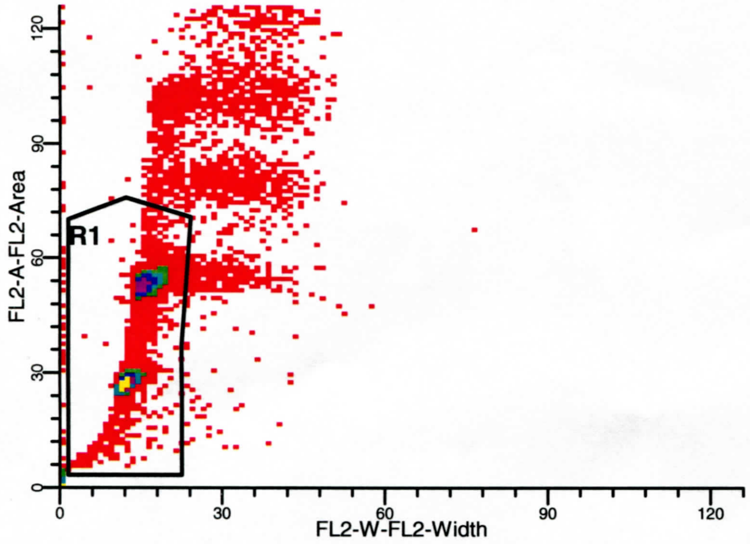

SAMPLE ID: ADR 0.2 B

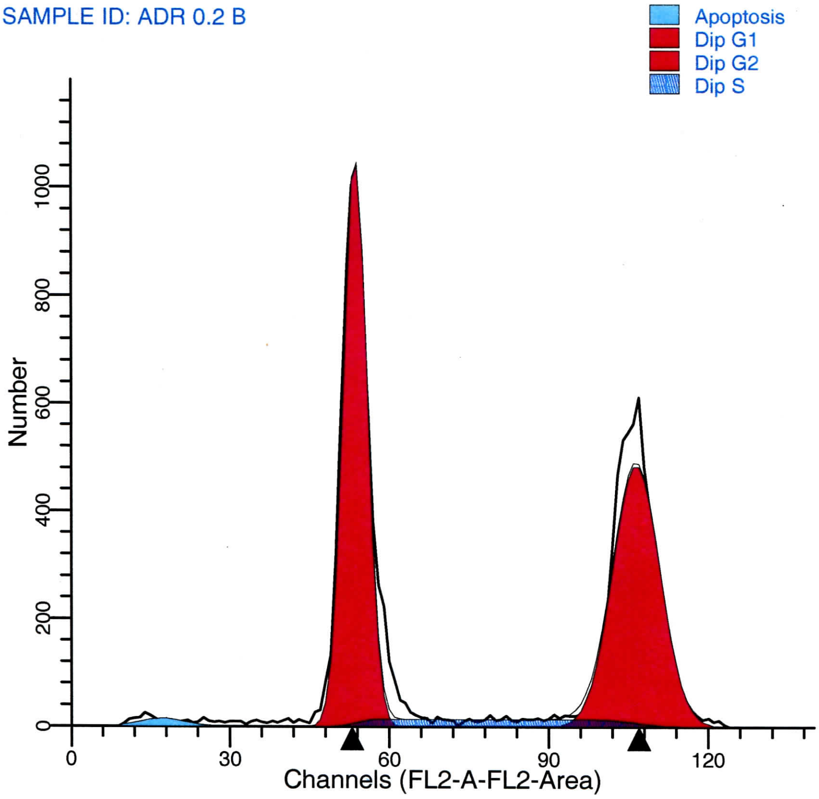


File analyzed: RK25U09.004
Date analyzed: 25-Jun-2009
Model: 1nn0A_DSF
Analysis type: Manual analysis

Diploid: 100.00 %
Dip G1: 49.41 % at 53.62
Dip G2: 43.42 % at 106.50
Dip S: 7.17 %   G2/G1: 1.99
%CV: 5.12

Total S-Phase: 7.17 %
Total B.A.D.: 0.00 %   no debris no aggs

Apoptosis: 1.29 %   Mean: 17.10

Debris:  %
Aggregates: 0.00 %
Modeled events: 12162
All cycle events: 12005
Cycle events per channel: 223
RCS: 7.800

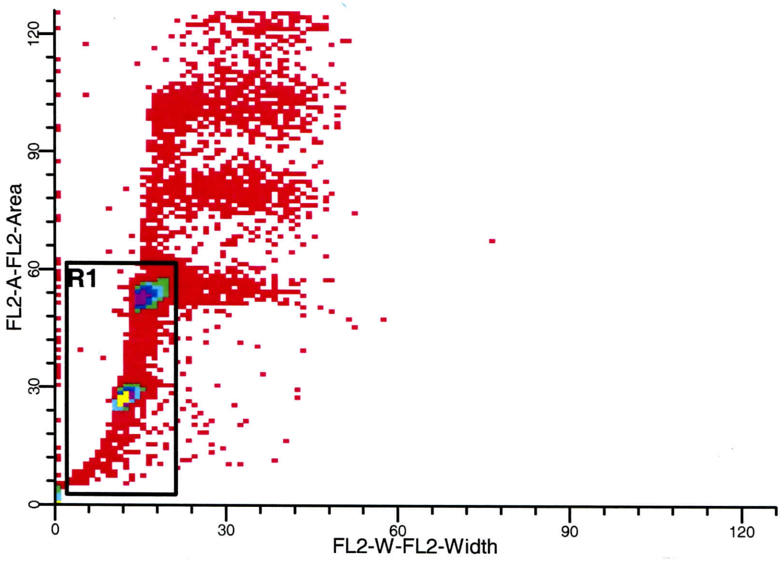


ModFitLT V3.0(Win32)

SAMPLE ID: ADR 0.5 A

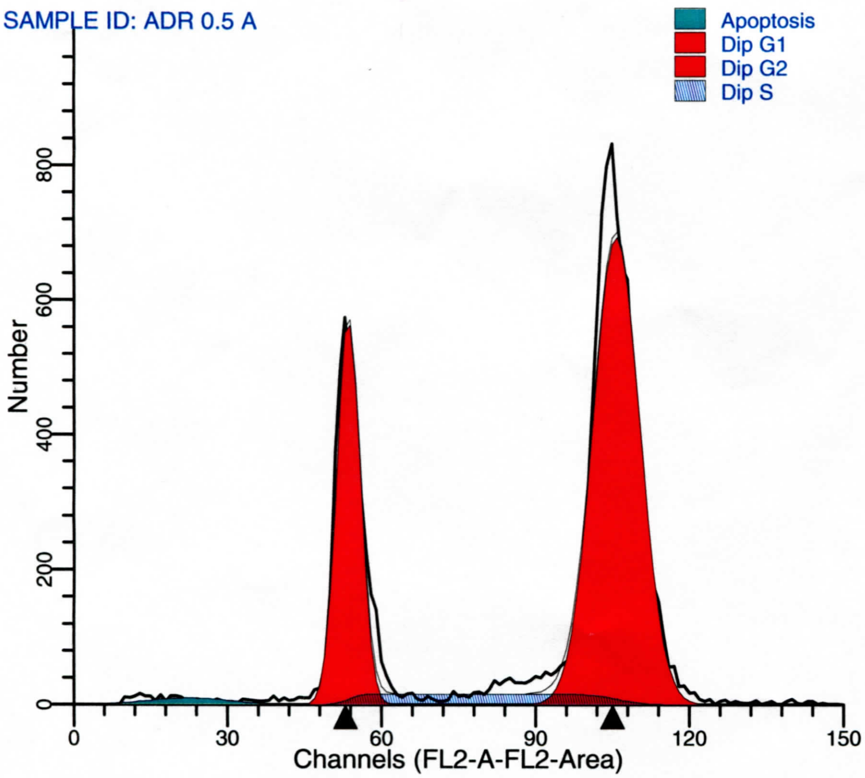


File analyzed: RK25U09.005
Date analyzed: 25-Jun-2009
Model: 1nn0A_DSF
Analysis type: Manual analysis

Diploid: 100.00 %
Dip G1: 27.24 % at 53.61
Dip G2: 65.81 % at 105.82
Dip S: 6.95 %   G2/G1: 1.97
%CV: 4.23

Total S-Phase: 6.95 %
Total B.A.D.: 0.00 %   no debris no aggs

Apoptosis: 1.55 %   Mean: 22.48

Debris:  %
Aggregates: 0.00 %
Modeled events: 12214
All cycle events: 12025
Cycle events per channel: 226
RCS: 6.550

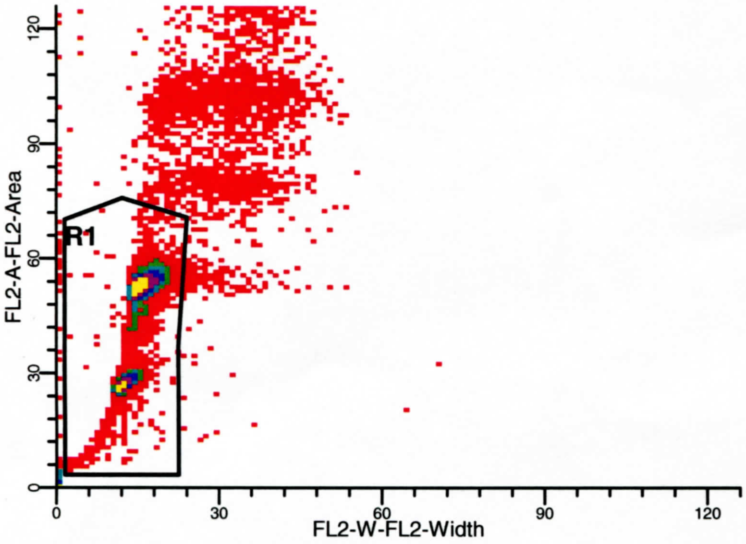


ModFitLT V3.0(Win32)

SAMPLE ID: ADR 0.5 B

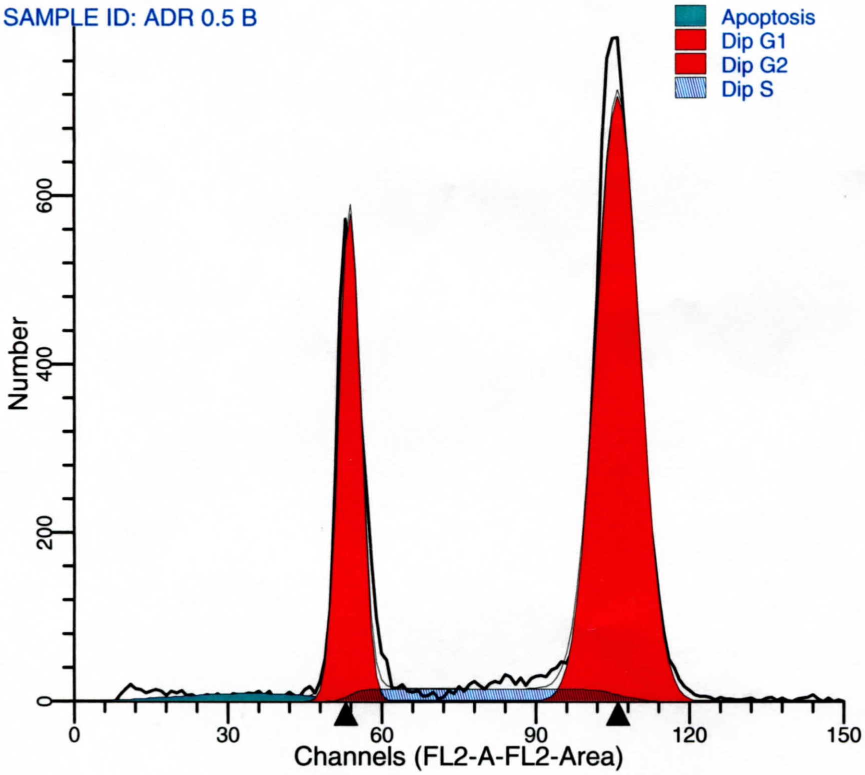


File analyzed: RK25U09.006
Date analyzed: 25-Jun-2009
Model: 1nn0A_DSF
Analysis type: Manual analysis

Diploid: 100.00 %
Dip G1: 26.91 % at 53.82
Dip G2: 66.11 % at 106.07
Dip S: 6.98 % G2/G1: 1.97
%CV: 3.92

Total S-Phase: 6.98 %
Total B.A.D.: 0.00 % no debris no aggs

Apoptosis: 2.83 % Mean: 34.16

Debris: %
Aggregates: 0.00 %
Modeled events: 11867
All cycle events: 11531
Cycle events per channel: 217
RCS: 5.018

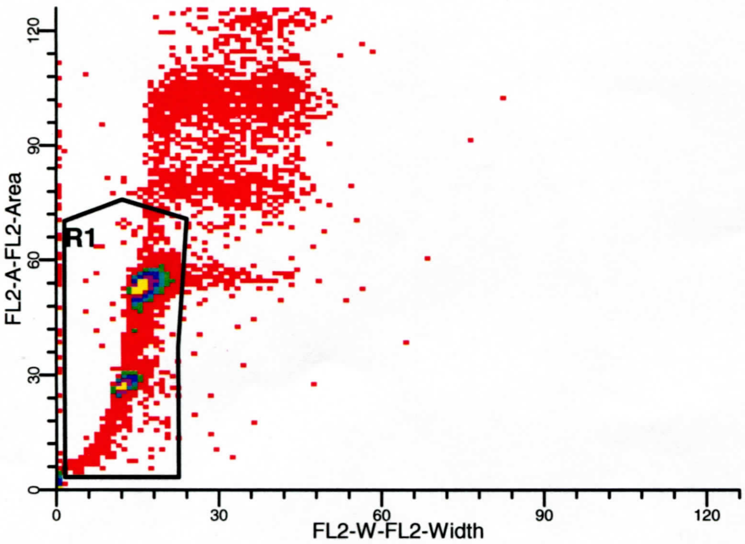

SAMPLE ID: ADR 1.0 A

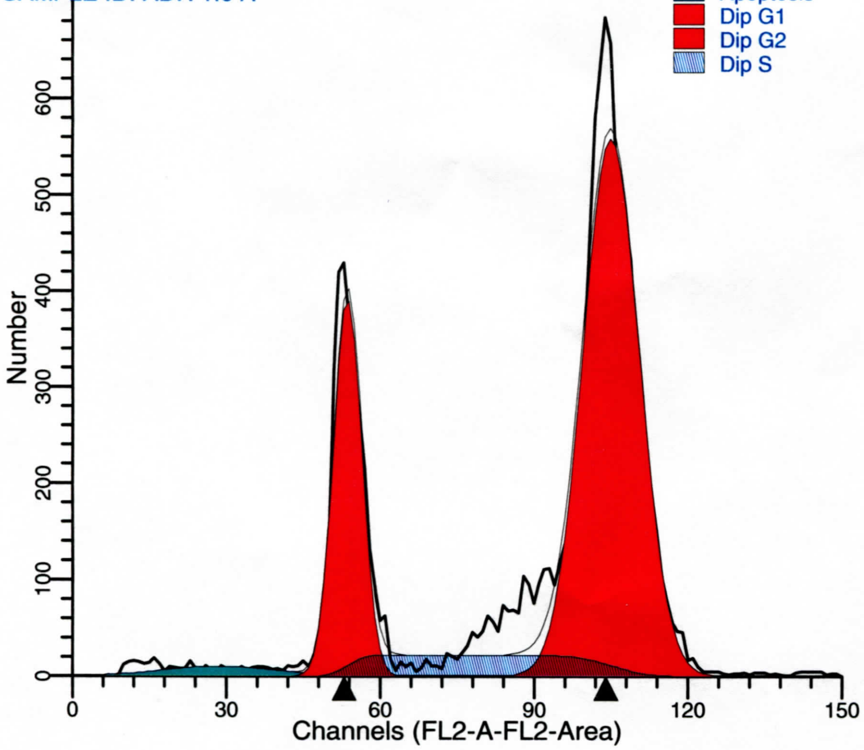


File analyzed: RK25U09.007
Date analyzed: 25-Jun-2009
Model: 1nn0A_DSF
Analysis type: Manual analysis

Diploid: 100.00 %
Dip G1: 23.51 % at 53.68
Dip G2: 66.66 % at 105.08
Dip S: 9.83 %   G2/G1: 1.96
%CV: 5.12

Total S-Phase: 9.83 %
Total B.A.D.: 0.00 %   no debris no aggs

Apoptosis: 2.45 %   Mean: 28.65

Debris:  %
Aggregates: 0.00 %
Modeled events: 11867
All cycle events: 11576
Cycle events per channel: 221
RCS: 7.779

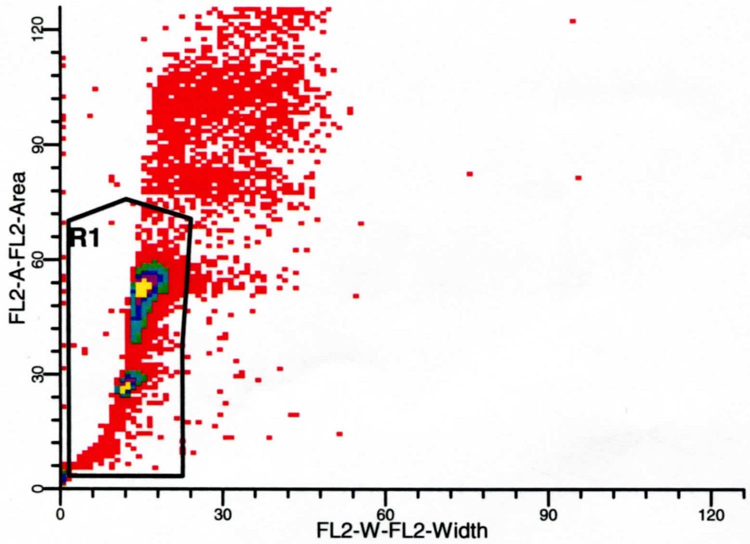

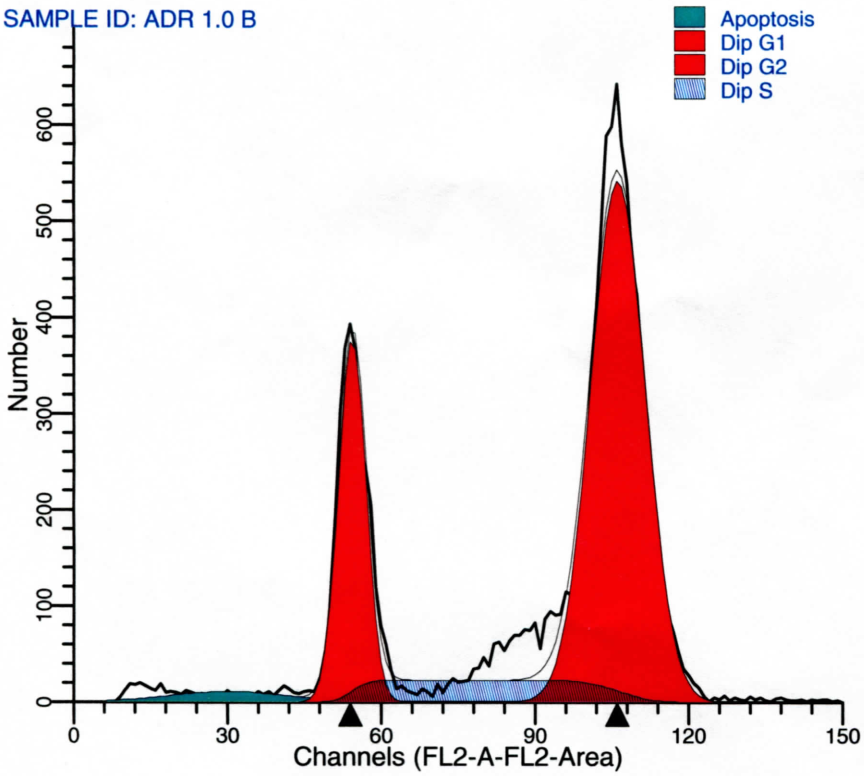


File analyzed: RK25U09.008
Date analyzed: 25-Jun-2009
Model: 1nn0A_DSF
Analysis type: Manual analysis

Diploid: 100.00 %
Dip G1: 23.15 % at 54.37
Dip G2: 65.95 % at 106.20
Dip S: 10.91 % G2/G1: 1.95
%CV: 4.92

Total S-Phase: 10.91 %
Total B.A.D.: 0.00 % no debris no aggs

Apoptosis: 2.86 % Mean: 30.34

Debris: %
Aggregates: 0.00 %
Modeled events: 11358
All cycle events: 11033
Cycle events per channel: 209
RCS: 6.228

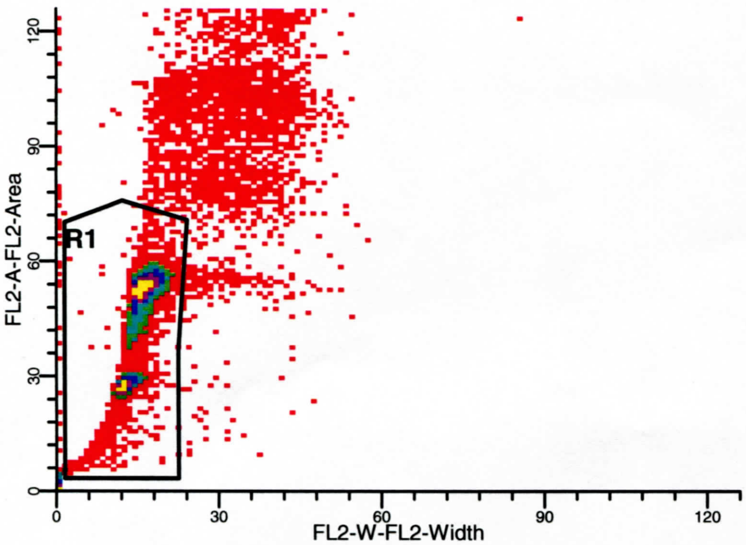


ModFitLT V3.0(Win32)

SAMPLE ID: ADR 2A

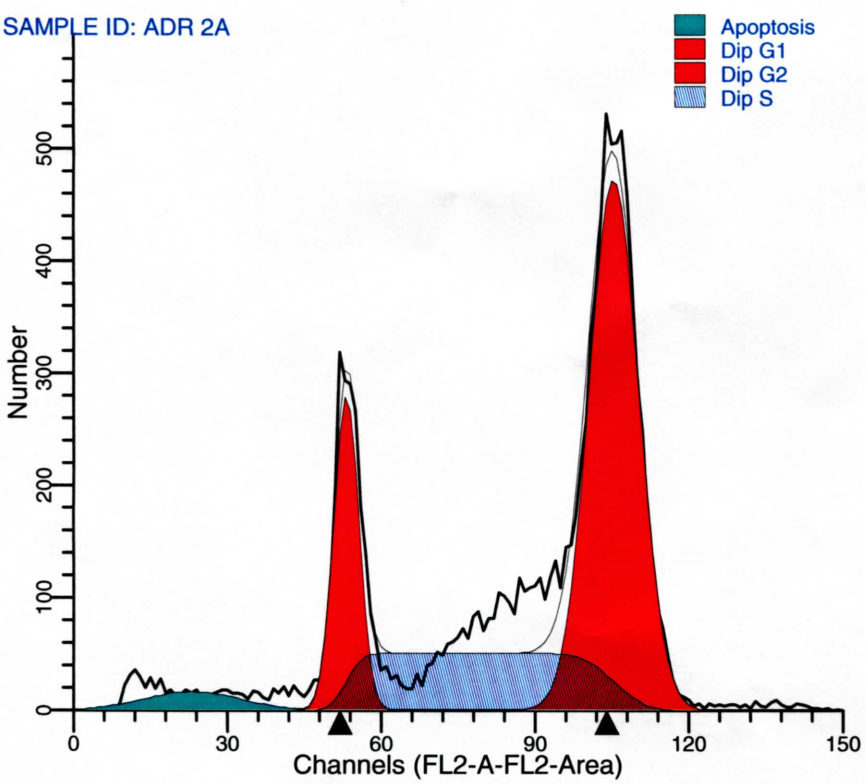


File analyzed: RK25U09.009
Date analyzed: 25-Jun-2009
Model: 1nn0A_DSF
Analysis type: Manual analysis

Diploid: 100.00 %
Dip G1: 16.94 % at 53.24
Dip G2: 56.89 % at 105.38
Dip S: 26.17 %   G2/G1: 1.98
%CV: 4.56

Total S-Phase: 26.17 %
Total B.A.D.: 0.00 %   no debris no aggs

Apoptosis: 3.69 %   Mean: 23.04

Debris:  %
Aggregates: 0.00 %
Modeled events: 10527
All cycle events: 10139
Cycle events per channel: 191
RCS: 8.184

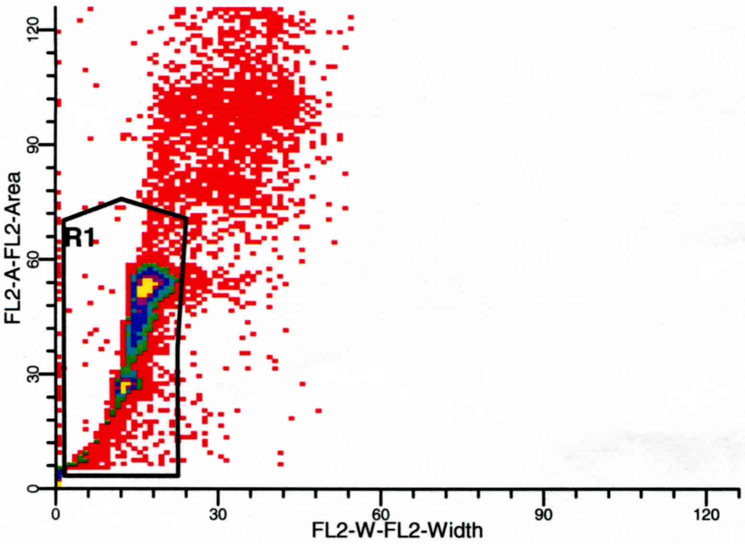


ModFitLT V3.0(Win32)

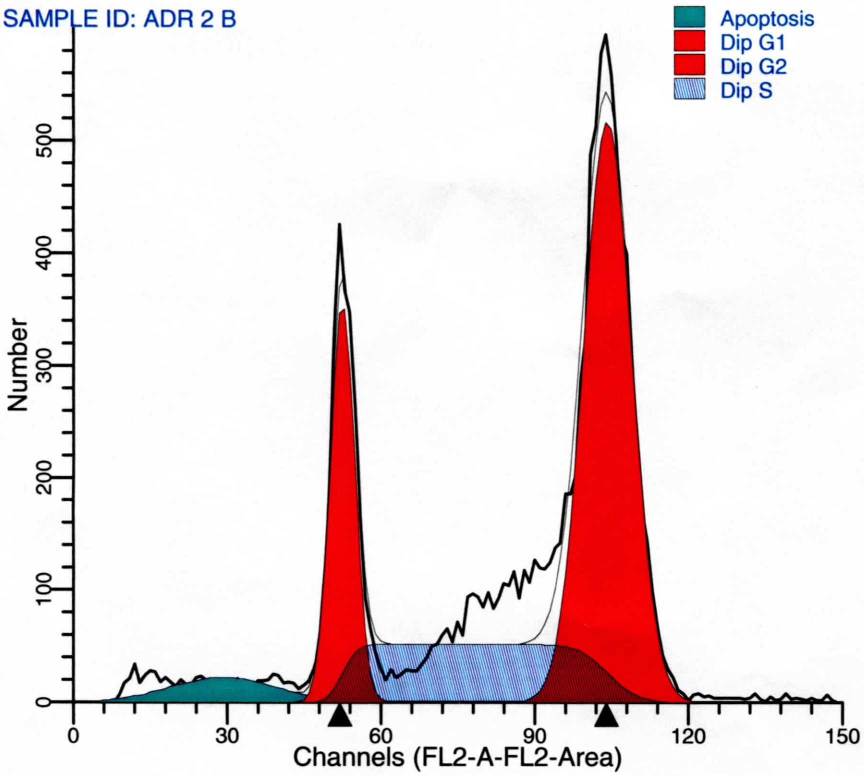


File analyzed: RK25U09.010
Date analyzed: 25-Jun-2009
Model: 1nn0A_DSF
Analysis type: Manual analysis

Diploid: 100.00 %
Dip G1: 19.46 % at 52.55
Dip G2: 56.16 % at 104.24
Dip S: 24.39 % G2/G1: 1.98
%CV: 4.50

Total S-Phase: 24.39 %
Total B.A.D.: 0.00 % no debris no aggs

Apoptosis: 4.95 % Mean: 29.22

Debris: %
Aggregates: 0.00 %
Modeled events: 11522
All cycle events: 10951
Cycle events per channel: 208
RCS: 7.549

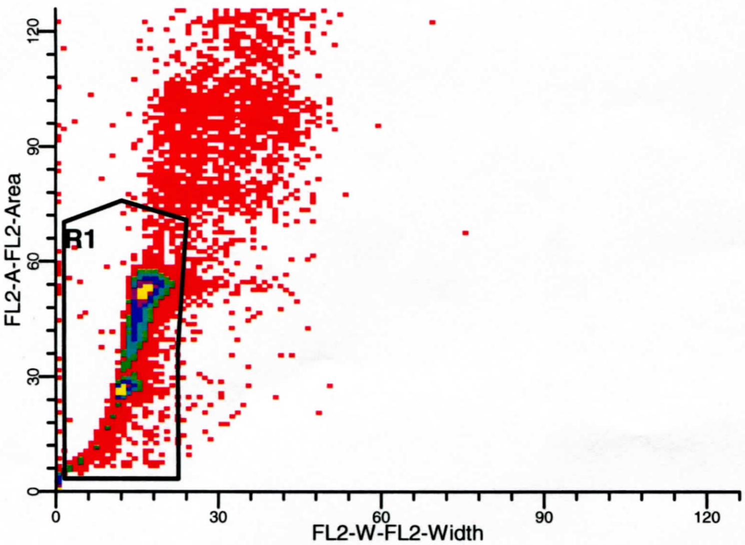

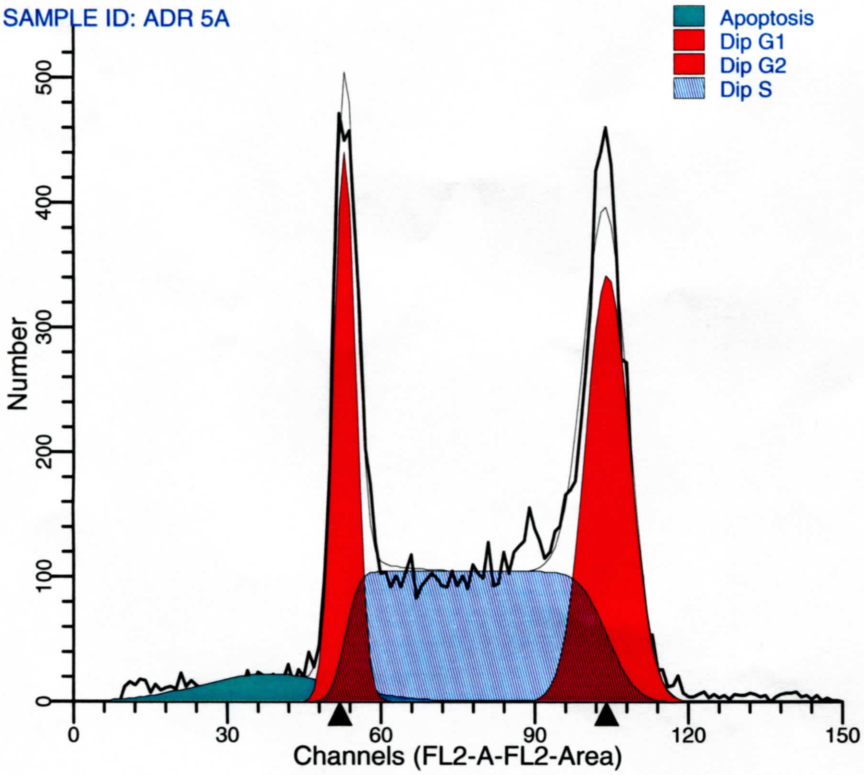


File analyzed: RK25U09.011
Date analyzed: 25-Jun-2009
Model: 1nn0A_DSF
Analysis type: Manual analysis

Diploid: 100.00 %
Dip G1: 20.95 % at 53.03
Dip G2: 32.31 % at 104.25
Dip S: 46.73 % G2/G1: 1.97
%CV: 4.07

Total S-Phase: 46.73 %
Total B.A.D.: 0.00 % no debris no aggs

Apoptosis: 6.09 % Mean: 38.43

Debris: %
Aggregates: 0.00 %
Modeled events: 12223
All cycle events: 11479
Cycle events per channel: 220
RCS: 3.415

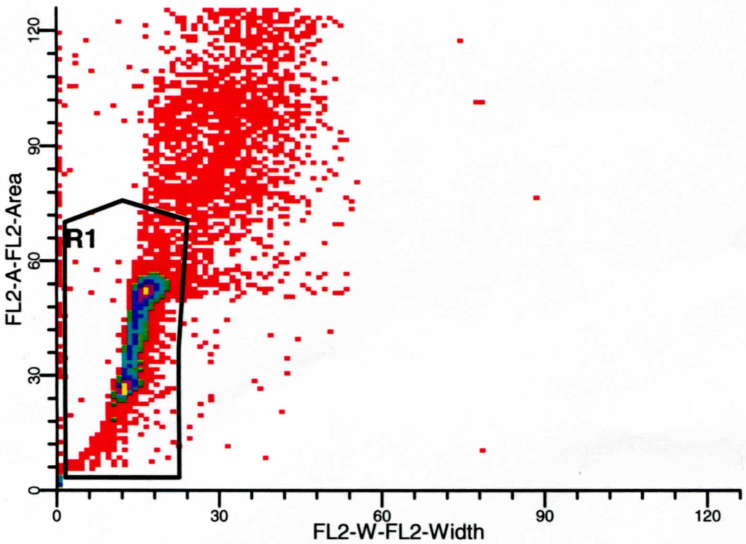


ModFitLT V3.0(Win32)

SAMPLE ID: ADR 5B

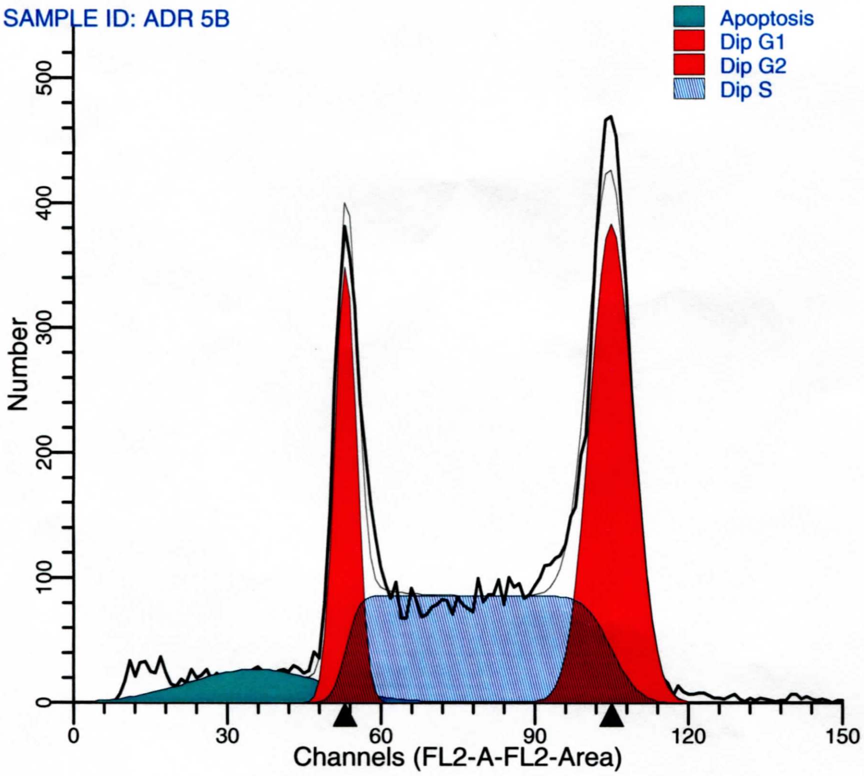


File analyzed: RK25U09.012
Date analyzed: 25-Jun-2009
Model: 1nn0A_DSF
Analysis type: Manual analysis

Diploid: 100.00 %
Dip G1: 17.95 % at 53.16
Dip G2: 39.06 % at 105.02
Dip S: 42.99 % G2/G1: 1.98
%CV: 3.96

Total S-Phase: 42.99 %
Total B.A.D.: 0.00 % no debris no aggs

Apoptosis: 7.84 % Mean: 35.69

Debris: %
Aggregates: 0.00 %
Modeled events: 11278
All cycle events: 10394
Cycle events per channel: 197
RCS: 3.469

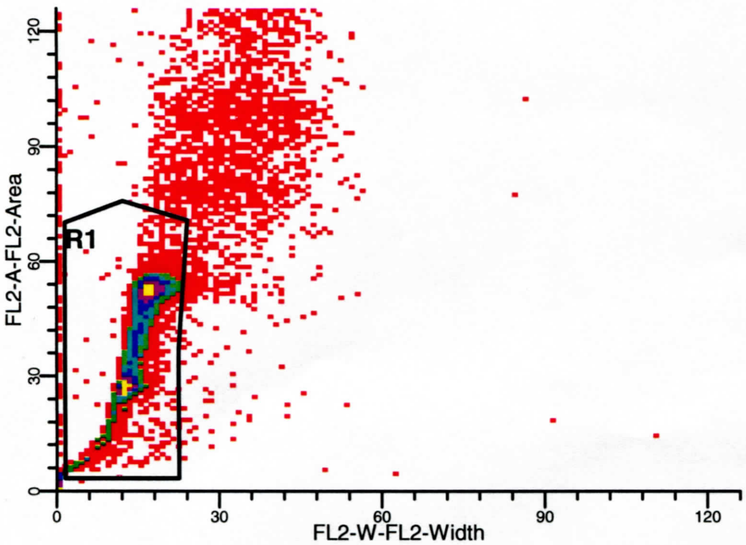


ModFitLT V3.0(Win32)

SAMPLE ID: ADR 10 A

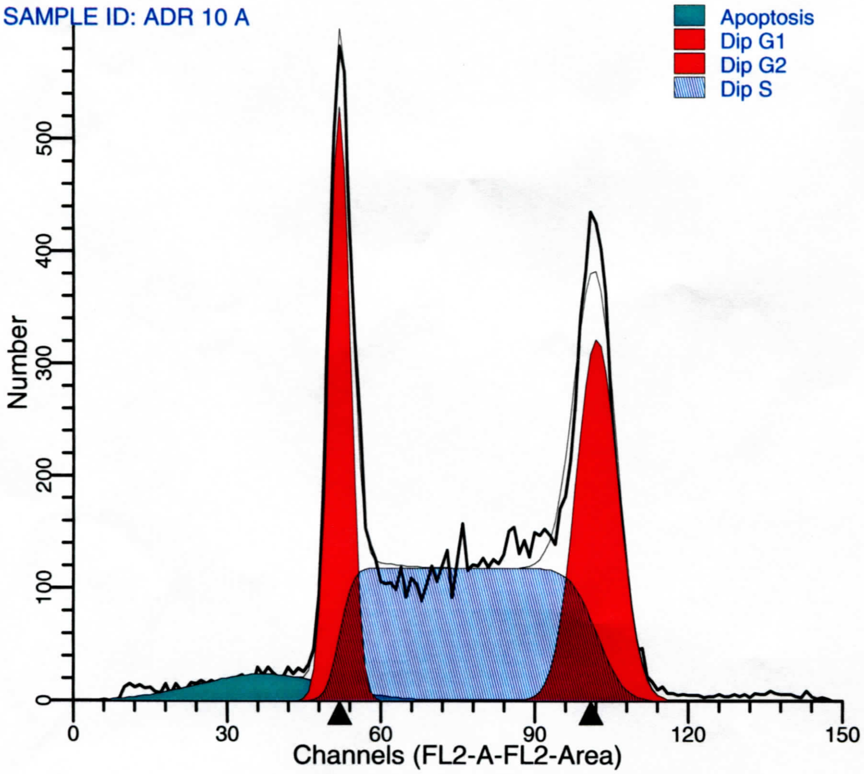


File analyzed: RK25U09.013
Date analyzed: 25-Jun-2009
Model: 1nn0A_DSF
Analysis type: Manual analysis

Diploid: 100.00 %
Dip G1: 22.92 % at 52.02
Dip G2: 27.71 % at 102.18
Dip S: 49.37 % G2/G1: 1.96
%CV: 3.95

Total S-Phase: 49.37 %
Total B.A.D.: 0.00 % no debris no aggs

Apoptosis: 5.93 % Mean: 37.04

Debris: %
Aggregates: 0.00 %
Modeled events: 12754
All cycle events: 11998
Cycle events per channel: 235
RCS: 3.115

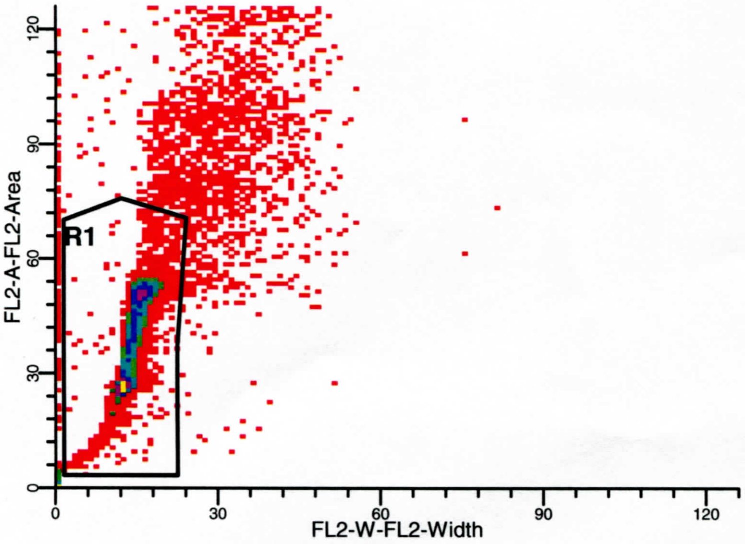

SAMPLE ID: ADR 10 B

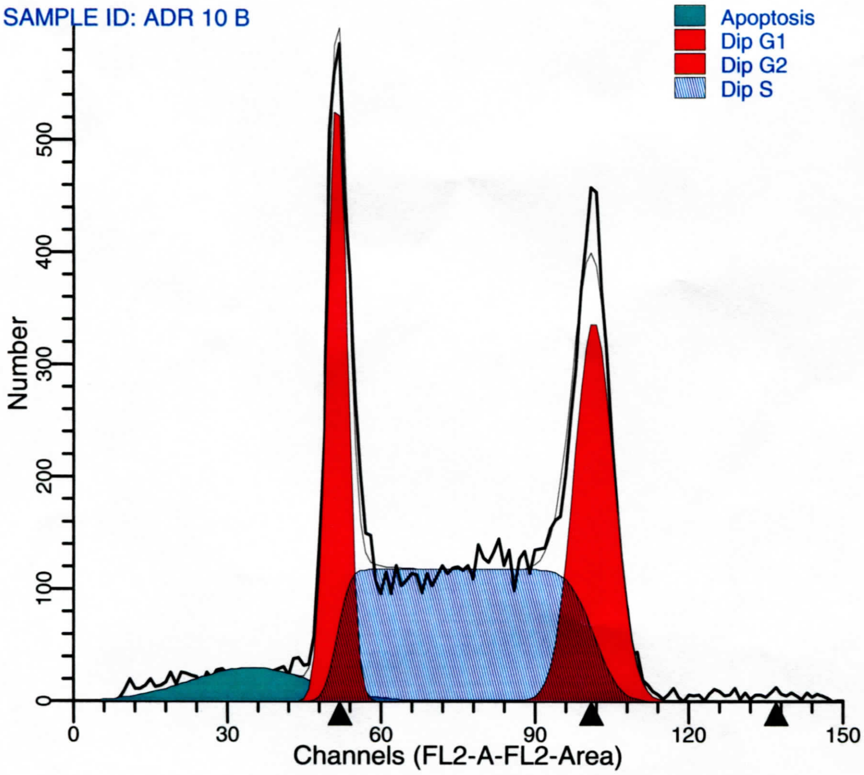


File analyzed: RK25U09.014
Date analyzed: 25-Jun-2009
Model: 1nn0A_DSF
Analysis type: Manual analysis

Diploid: 100.00 %
Dip G1: 22.44 % at 51.48
Dip G2: 27.83 % at 101.50
Dip S: 49.73 %  G2/G1: 1.97
%CV: 3.76

Total S-Phase: 49.73 %
Total B.A.D.: 0.00 %  no debris no aggs

Apoptosis: 7.06 %  Mean: 34.68

Debris: %
Aggregates: 0.00 %
Modeled events: 12738
All cycle events: 11838
Cycle events per channel: 232
RCS: 3.423

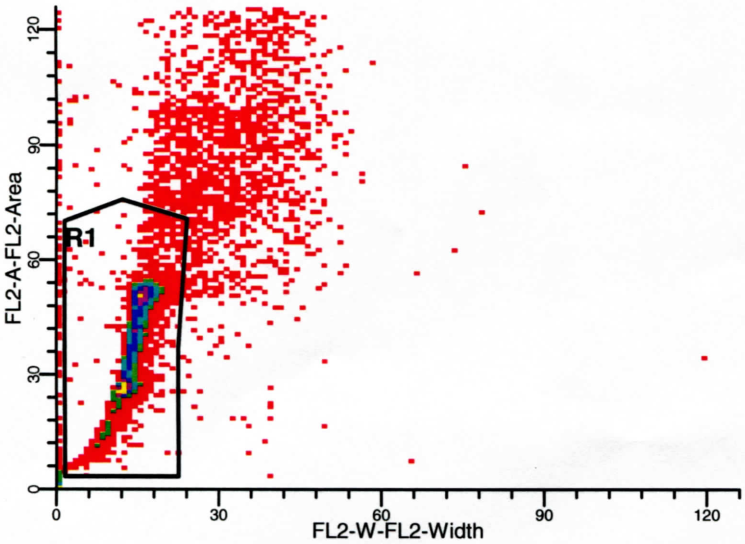


ModFitLT V3.0(Win32)

SAMPLE ID: ADR 20A

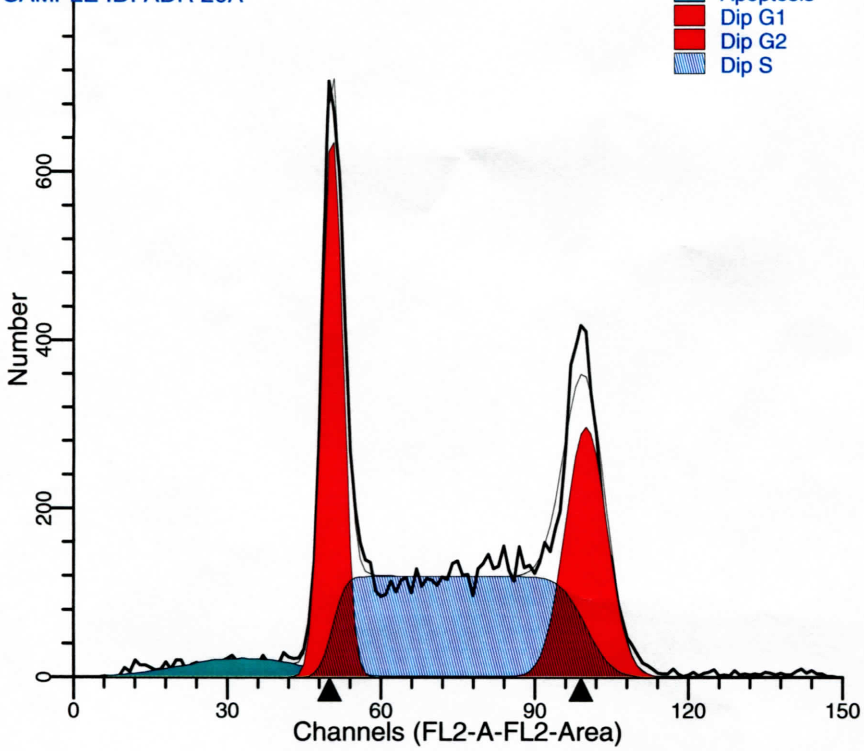


File analyzed: RK25U09.015
Date analyzed: 25-Jun-2009
Model: 1nn0A_DSF
Analysis type: Manual analysis

Diploid: 100.00 %
Dip G1: 27.03 % at 50.57
Dip G2: 24.56 % at 100.01
Dip S: 48.41 % G2/G1: 1.98
%CV: 3.97

Total S-Phase: 48.41 %
Total B.A.D.: 0.00 % no debris no aggs

Apoptosis: 5.05 % Mean: 33.03

Debris: %
Aggregates: 0.00 %
Modeled events: 12862
All cycle events: 12212
Cycle events per channel: 242
RCS: 3.023

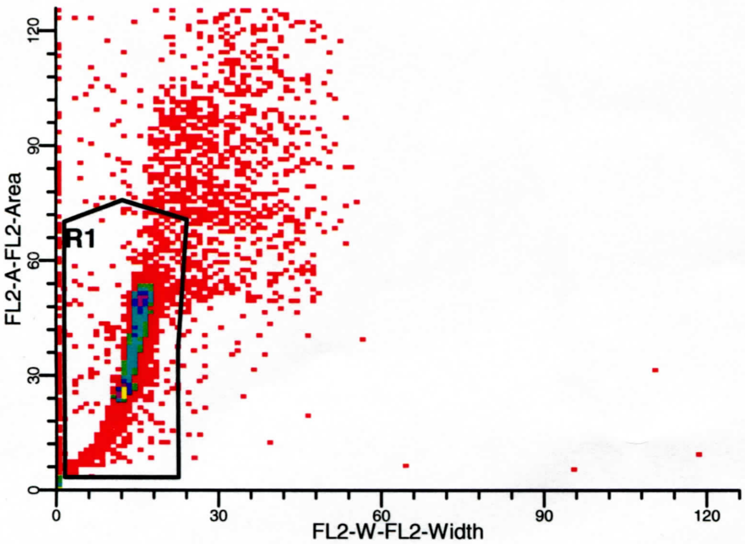

SAMPLE ID: ADR 20B

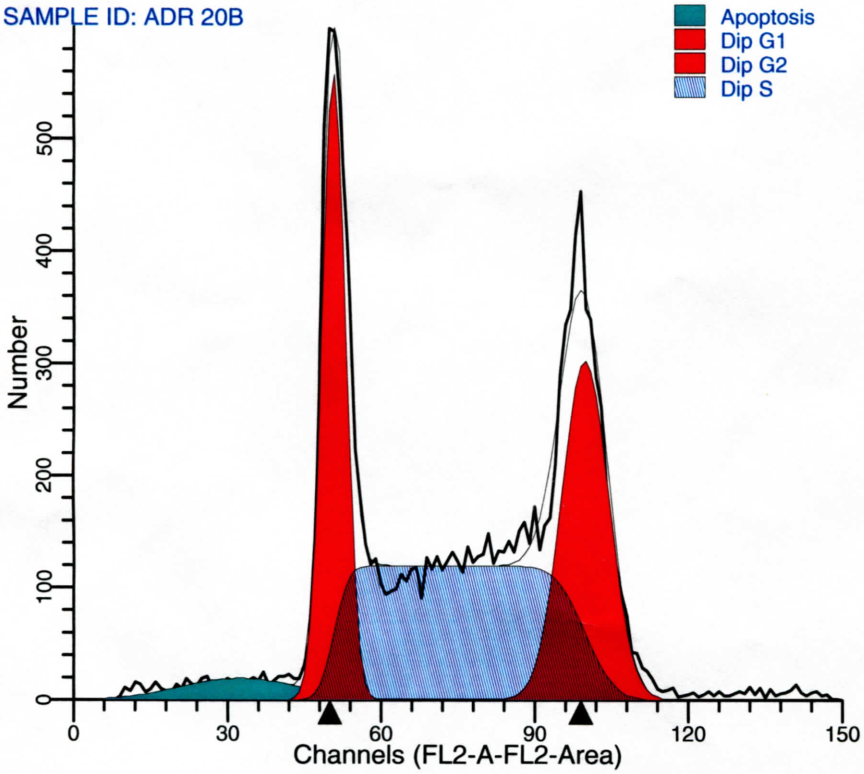


File analyzed: RK25U09.016
Date analyzed: 25-Jun-2009
Model: 1nn0A_DSF
Analysis type: Manual analysis

Diploid: 100.00 %
Dip G1: 25.66 % at 50.82
Dip G2: 27.62 % at 99.86
Dip S: 46.72 % G2/G1: 1.97
%CV: 4.49

Total S-Phase: 46.72 %
Total B.A.D.: 0.00 % no debris no aggs

Apoptosis: 4.24 % Mean: 32.35

Debris: %
Aggregates: 0.00 %
Modeled events: 13134
All cycle events: 12577
Cycle events per channel: 251
RCS: 3.538

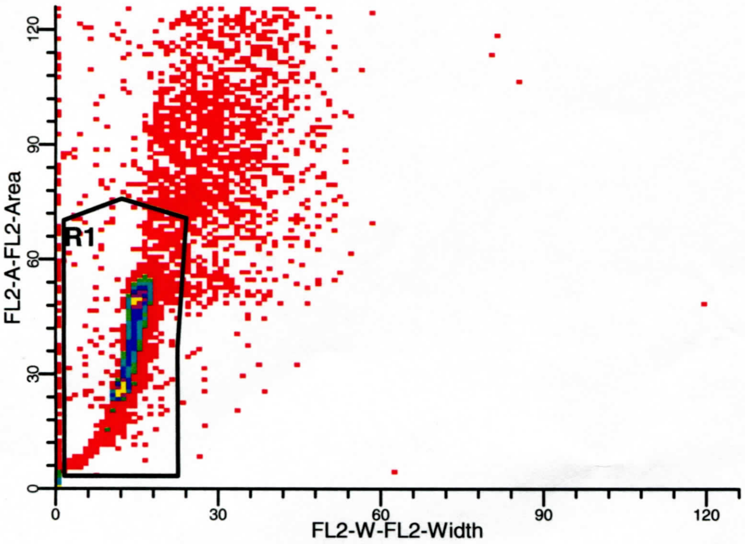


ModFitLT V3.0(Win32)
