# Supplementary material for: Correction: ERK1/2 Signaling Plays an Important Role in Topoisomerase II Poison-Induced G2/M Checkpoint Activation
Source: PLoS One. 2023 Sep 28;18(9):e0292423. doi: 10.1371/journal.pone.0292423 (PMC10538782; doi:10.1371/journal.pone.0292423)
Supplement: S1 File — (ZIP) [file pone.0292423.s001.zip › Figure 1A/ETOP-10 uM.pdf]

10 uM VP16, MCF7

SAMPLE ID: VP10 A

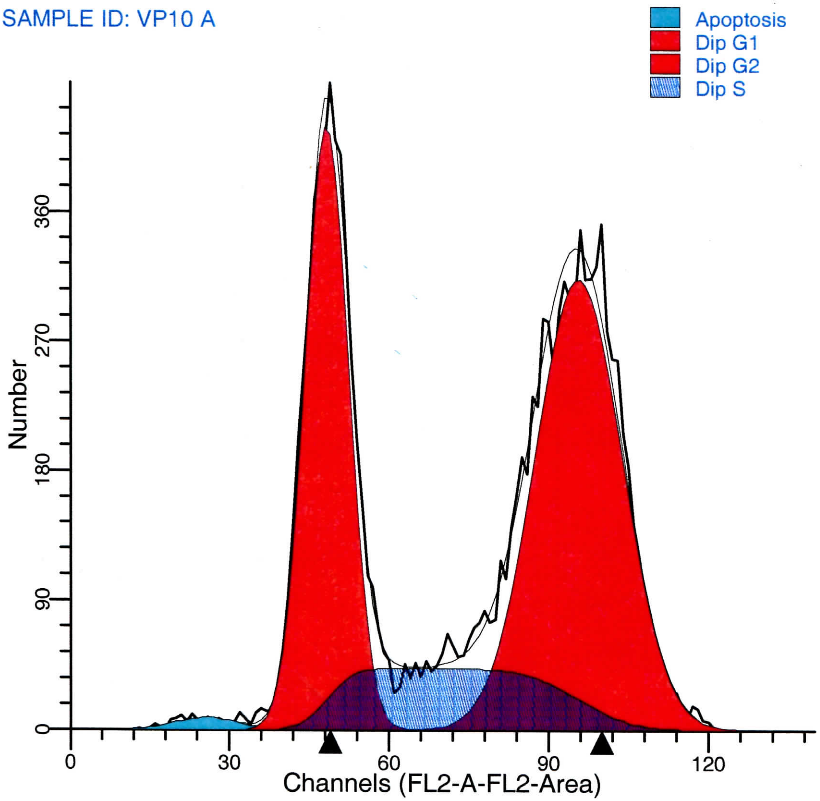

File analyzed: RK27U09.005
Date analyzed: 3-Sep-2009
Model: 1nn0A_DSF
Analysis type: Manual analysis

Diploid: 100.00 %
Dip G1: 31.77 % at 48.32
Dip G2: 50.27 % at 95.63 Dip
S: 15.96 % G2/G1: 1.98 %
CV: 8.48

Total S-Phase: 15.96 %
Total B.A.D.: 0.00 % no debris no aggs

Apoptosis: 1.02 % Mean: 25.66

Debris: %
Aggregates: 0.00 %
Modeled events: 12933
All cycle events: 12801
Cycle events per channel: 265
RCS: 1.502
