# Supplementary material for: Correction: ERK1/2 Signaling Plays an Important Role in Topoisomerase II Poison-Induced G2/M Checkpoint Activation
Source: PLoS One. 2023 Sep 28;18(9):e0292423. doi: 10.1371/journal.pone.0292423 (PMC10538782; doi:10.1371/journal.pone.0292423)
Supplement: S1 File — (ZIP) [file pone.0292423.s001.zip › Figure 1A/ETOP-FACS, 6-22-19.pdf]

Log A

SAMPLE ID: Log A

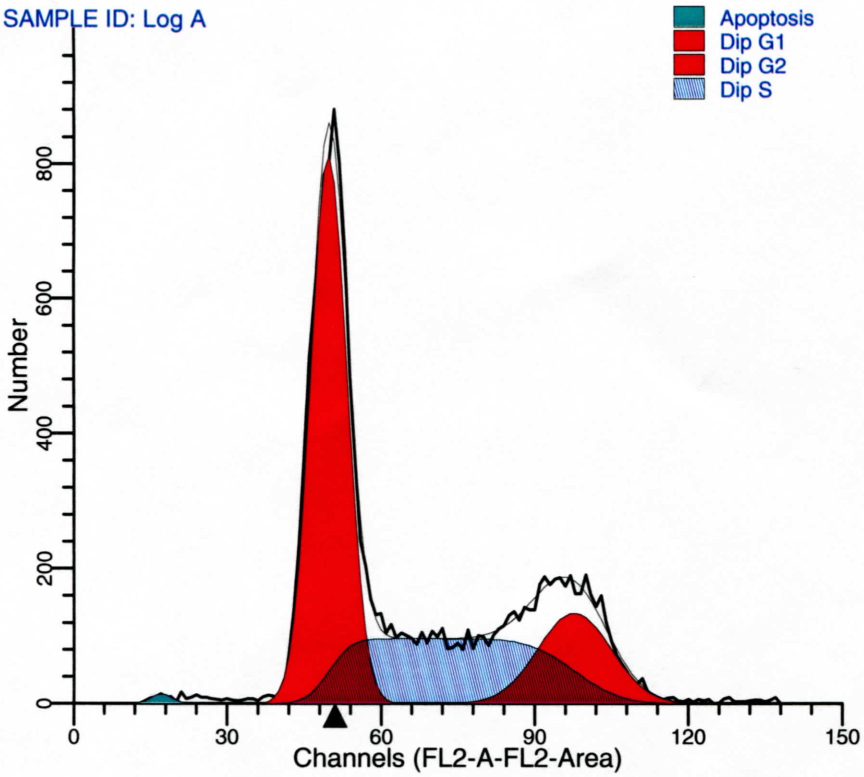

File analyzed: RK22U09.001
Date analyzed: 22-Jun-2009
Model: 1nn0A_DSF
Analysis type: Manual analysis

Diploid: 100.00 %
Dip G1: 50.81 % at 49.85
Dip G2: 16.78 % at 97.77
Dip S: 32.41 % G2/G1: 1.96
%CV: 7.12

Total S-Phase: 32.41 %
Total B.A.D.: 0.00 % no debris no aggs

Apoptosis: 0.49 % Mean: 17.13

Debris: %
Aggregates: 0.00 %
Modeled events: 14339
All cycle events: 14269
Cycle events per channel: 292
RCS: 2.609

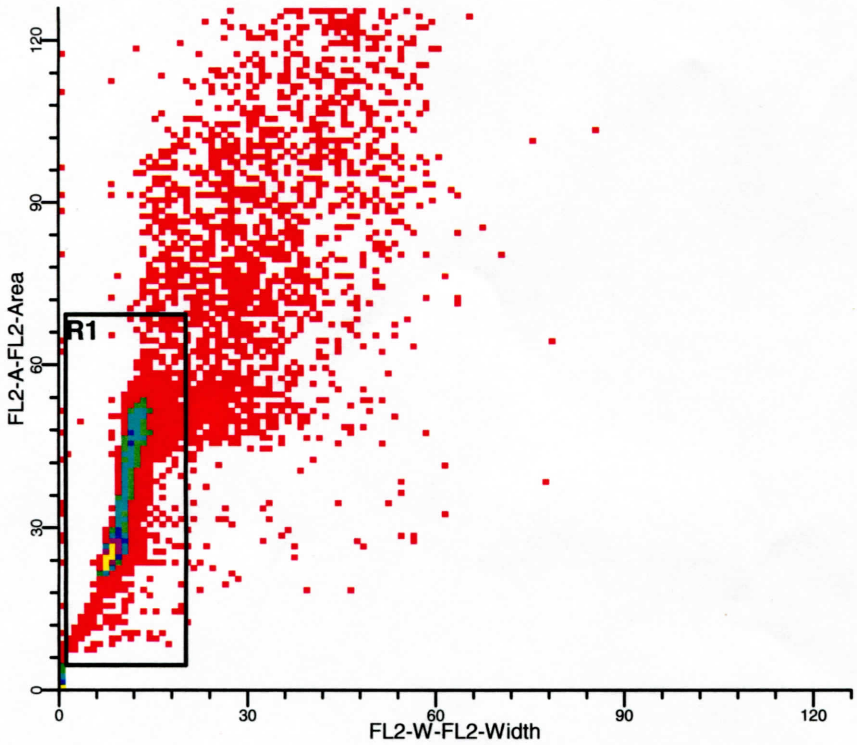

ModFitLT V3.0(Win32)

Log B

SAMPLE ID: Log B

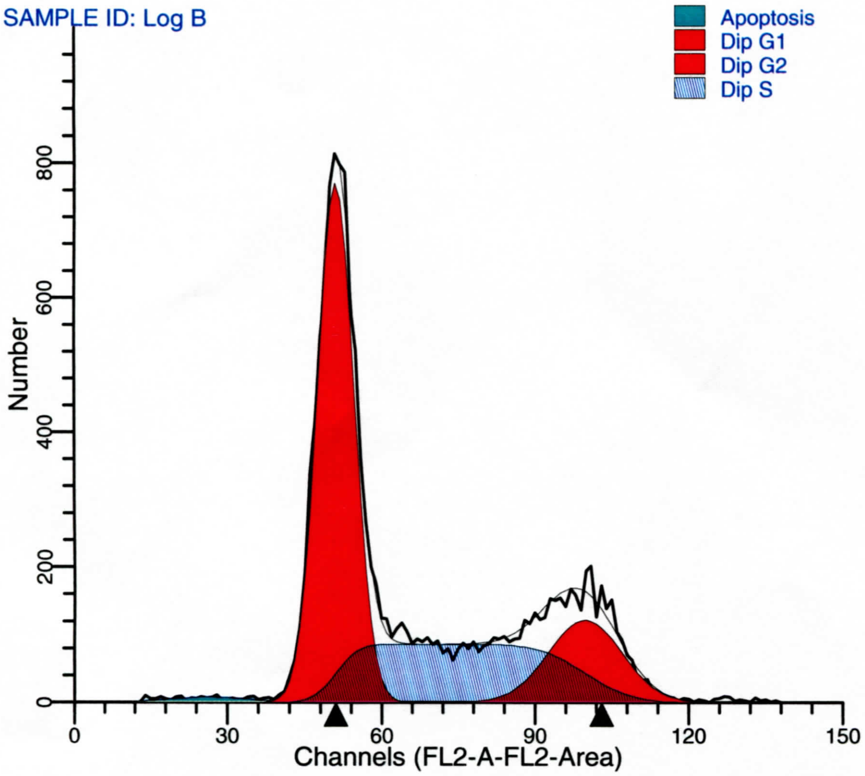

File analyzed: RK22U09.002
Date analyzed: 22-Jun-2009
Model: 1nn0A_DSF
Analysis type: Manual analysis

Diploid: 100.00 %
Dip G1: 52.01 % at 51.07
Dip G2: 16.40 % at 99.81
Dip S: 31.59 % G2/G1: 1.95
%CV: 7.01

Total S-Phase: 31.59 %
Total B.A.D.: 0.00 % no debris no aggs

Apoptosis: 1.09 % Mean: 26.38

Debris: %
Aggregates: 0.00 %
Modeled events: 13485
All cycle events: 13338
Cycle events per channel: 268
RCS: 1.983

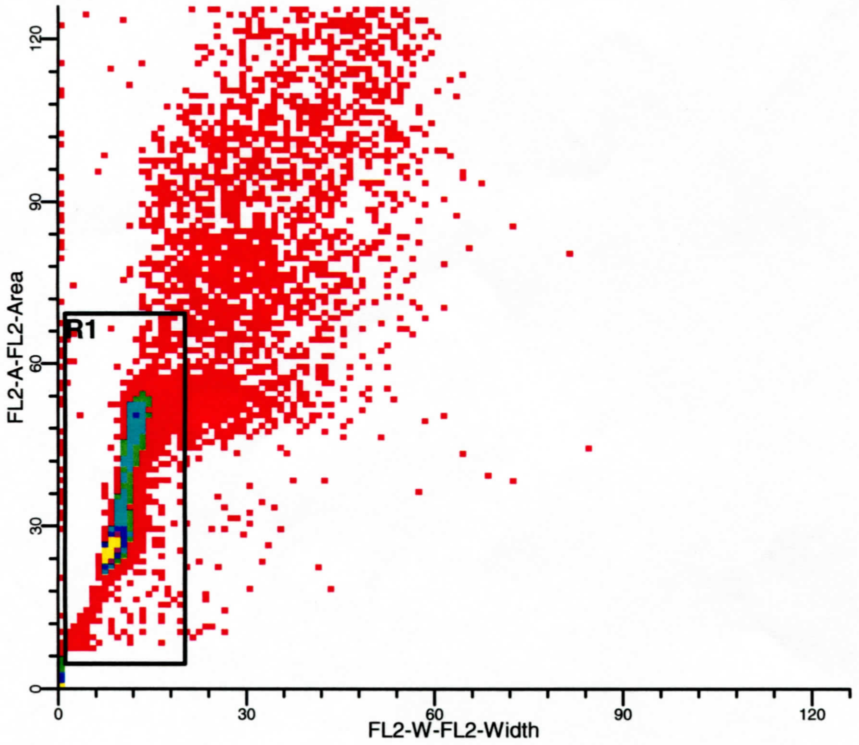

5uM VP-16-A

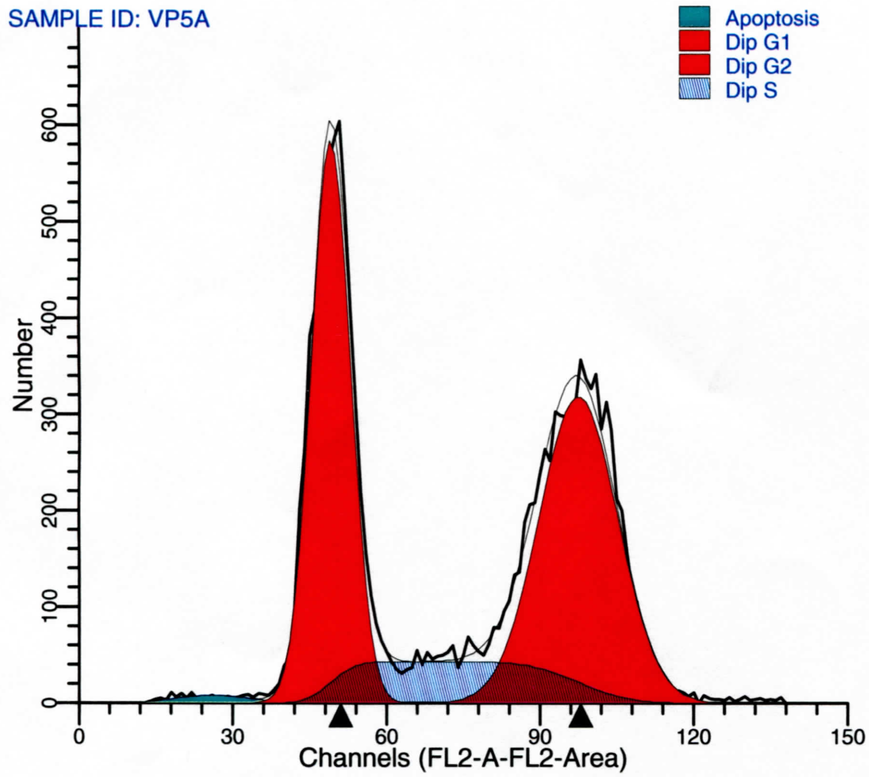

File analyzed: RK22U09.003
Date analyzed: 22-Jun-2009
Model: 1nn0A_DSF
Analysis type: Manual analysis

Diploid: 100.00 %
Dip G1: 40.68 % at 49.16
Dip G2: 44.26 % at 97.50
Dip S: 15.06 % G2/G1: 1.98
%CV: 7.83

Total S-Phase: 15.06 %
Total B.A.D.: 0.00 % no debris no aggs

Apoptosis: 1.02 % Mean: 26.14

Debris: %
Aggregates: 0.00 %
Modeled events: 14044
All cycle events: 13901
Cycle events per channel: 282
RCS: 2.019

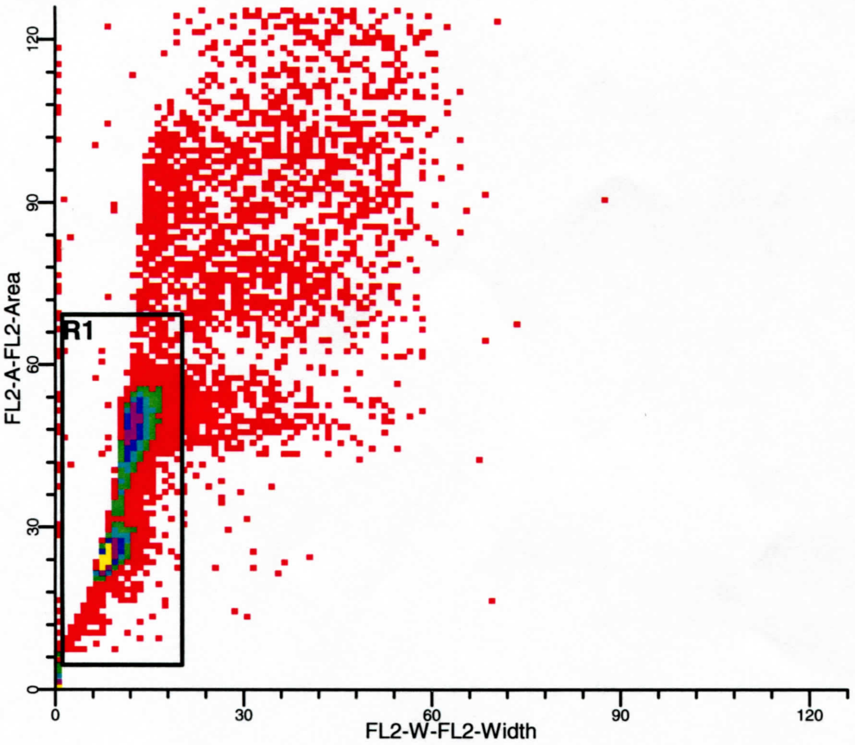

ModFitLT V3.0(Win32)

5 μM VP16-B

SAMPLE ID: VP5B

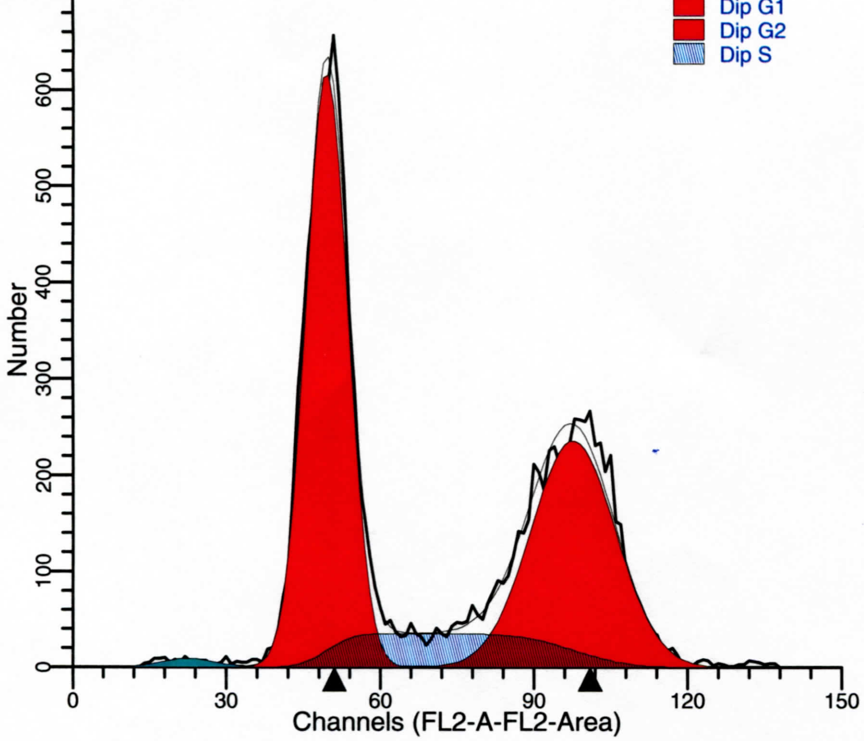

File analyzed: RK22U09.004
Date analyzed: 22-Jun-2009
Model: 1nn0A_DSF
Analysis type: Manual analysis

Diploid: 100.00 %
Dip G1: 49.35 % at 49.60
Dip G2: 37.63 % at 97.67
Dip S: 13.02 % G2/G1: 1.97
%CV: 8.30

Total S-Phase: 13.02 %
Total B.A.D.: 0.00 % no debris no aggs

Apoptosis: 0.91 % Mean: 22.61

Debris: %
Aggregates: 0.00 %
Modeled events: 13097
All cycle events: 12978
Cycle events per channel: 264
RCS: 1.872

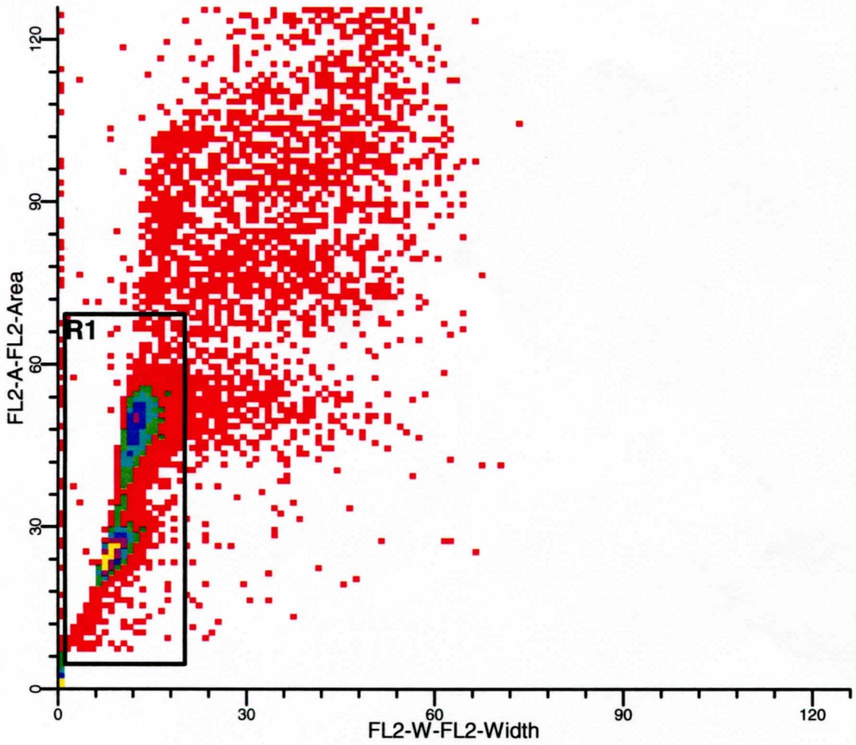

ModFitLT V3.0(Win32)

10 uM VP16-A

SAMPLE ID: VP10 A

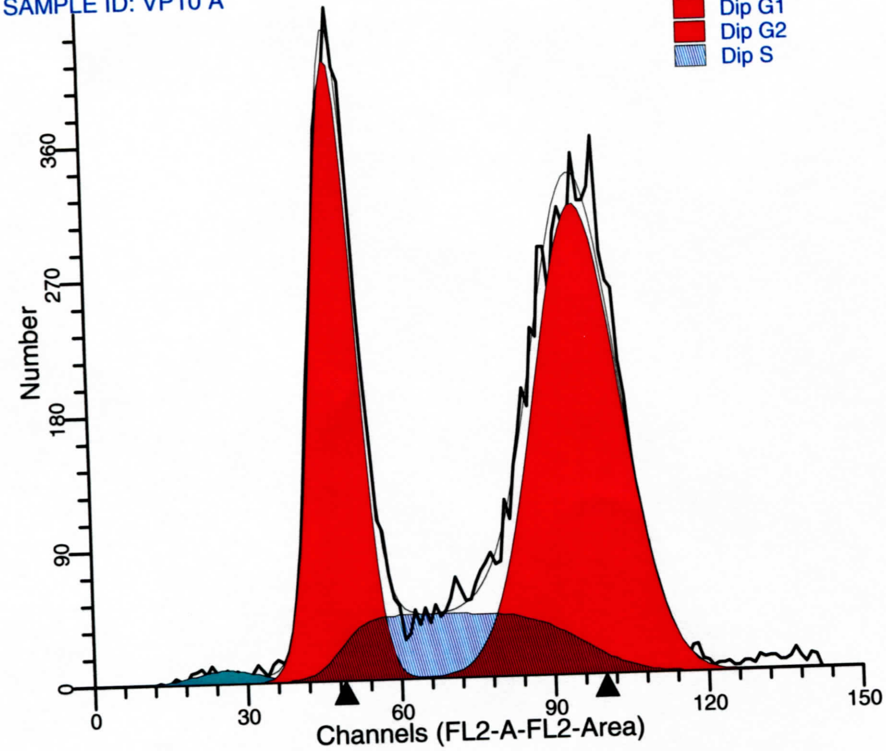

File analyzed: RK22U09.005
Date analyzed: 23-Jun-2009
Model: 1nn0A_DSF
Analysis type: Manual analysis

Diploid: 100.00 %
Dip G1: 33.67 % at 48.34
Dip G2: 50.65 % at 95.71
Dip S: 15.68 % G2/G1: 1.98
%CV: 8.58

Total S-Phase: 15.68 %
Total B.A.D.: 0.00 % no debris no aggs

Apoptosis: 1.04 % Mean: 26.13

Debris: %
Aggregates: 0.00 %
Modeled events: 13016
All cycle events: 12880
Cycle events per channel: 266
RCS: 2.235

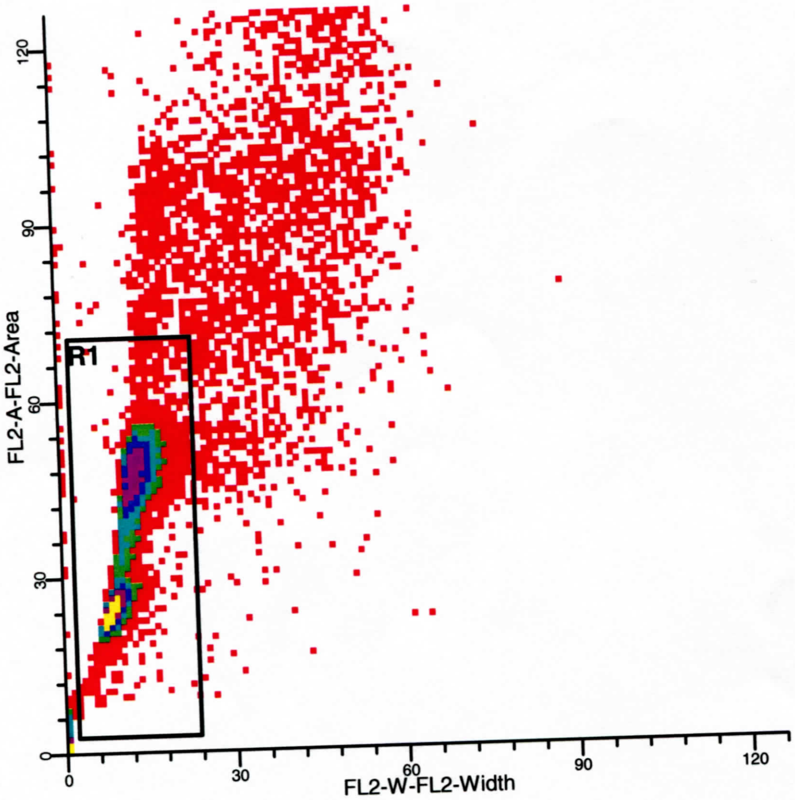

10μM VP16-B

SAMPLE ID: VP10 B

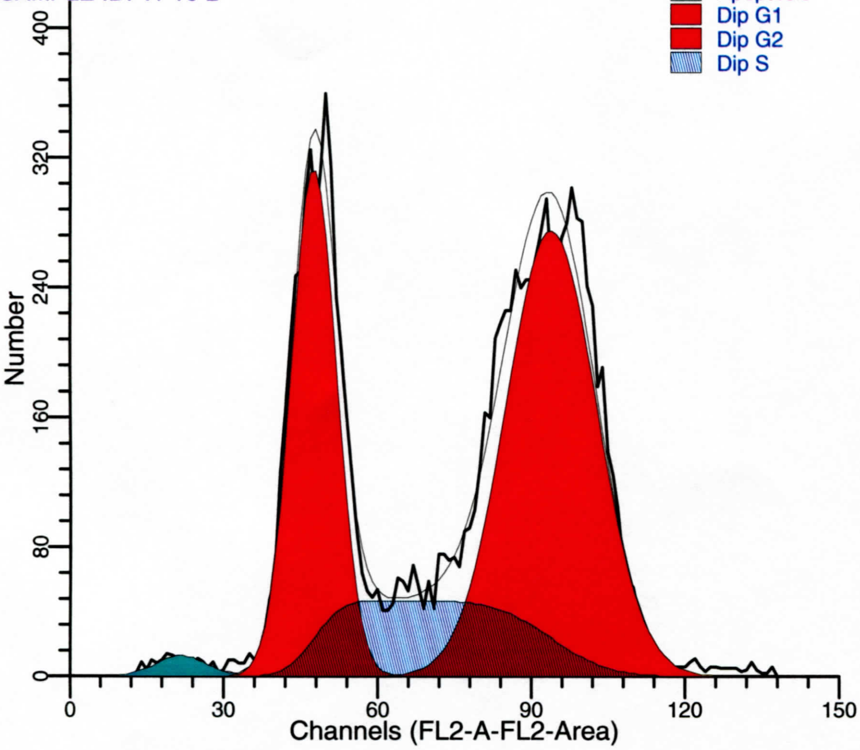

File analyzed: RK22U09.006
Date analyzed: 22-Jun-2009
Model: 1nn0A_DSF
Analysis type: Manual analysis

Diploid: 100.00 %
Dip G1: 29.60 % at 47.58
Dip G2: 51.86 % at 93.94
Dip S: 18.54 % G2/G1: 1.97
%CV: 9.34

Total S-Phase: 18.54 %
Total B.A.D.: 0.00 % no debris no aggs

Apoptosis: 1.32 % Mean: 21.79

Debris: %
Aggregates: 0.00 %
Modeled events: 11992
All cycle events: 11833
Cycle events per channel: 250
RCS: 2.355

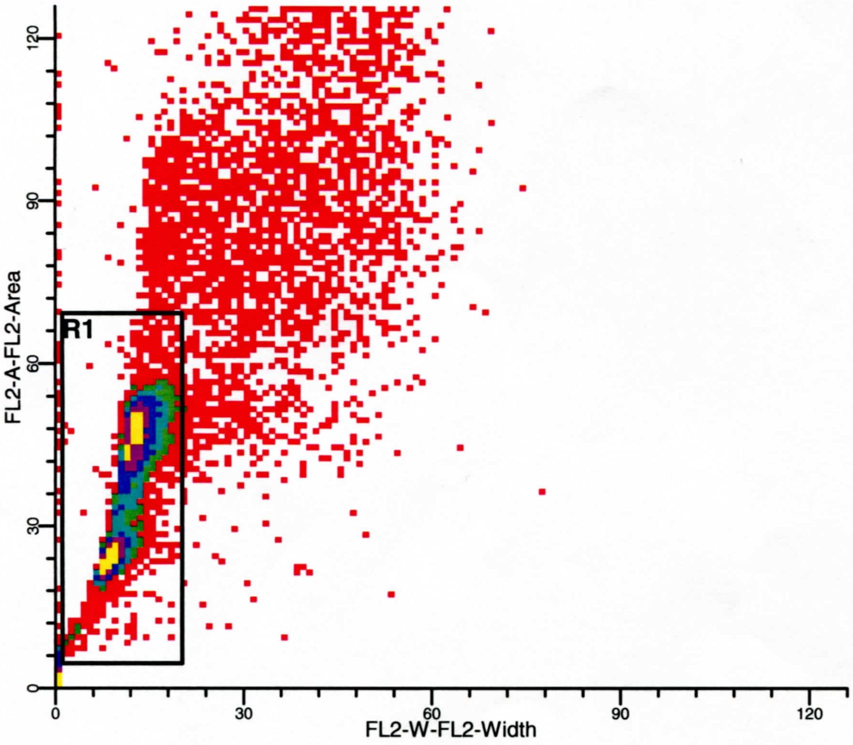

ModFitLT V3.0(Win32)

20uM VP16-A

SAMPLE ID: VP 20 A

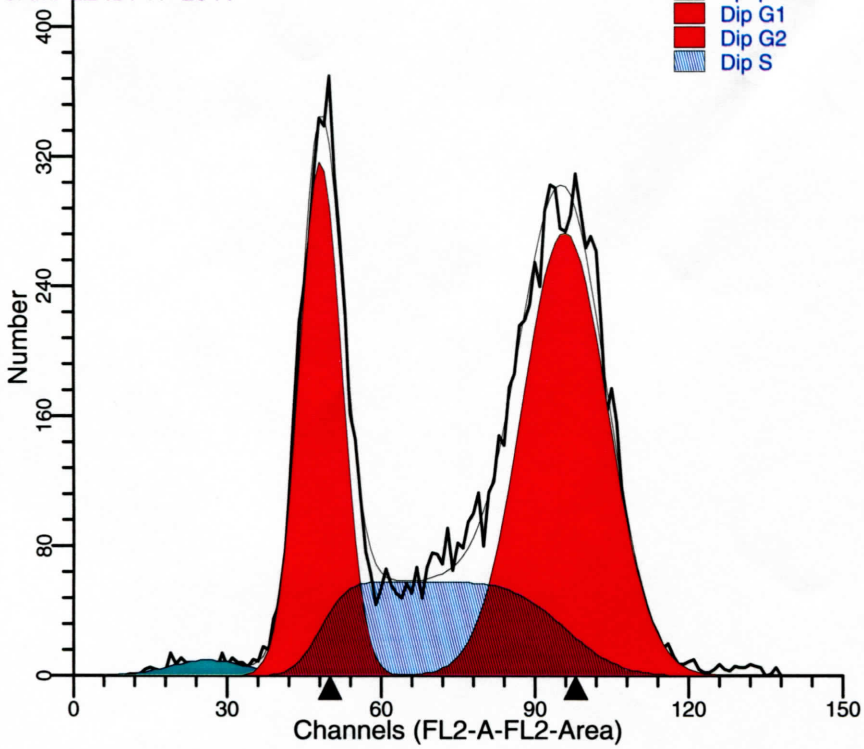

File analyzed: RK22U09.007
Date analyzed: 22-Jun-2009
Model: 1nn0A_DSF
Analysis type: Manual analysis

Diploid: 100.00 %
Dip G1: 28.10 % at 48.20
Dip G2: 48.36 % at 95.86
Dip S: 23.53 % G2/G1: 1.99
%CV: 8.66

Total S-Phase: 23.53 %
Total B.A.D.: 0.00 % no debris no aggs

Apoptosis: 1.50 % Mean: 26.36

Debris: %
Aggregates: 0.00 %
Modeled events: 12009
All cycle events: 11829
Cycle events per channel: 243
RCS: 1.560

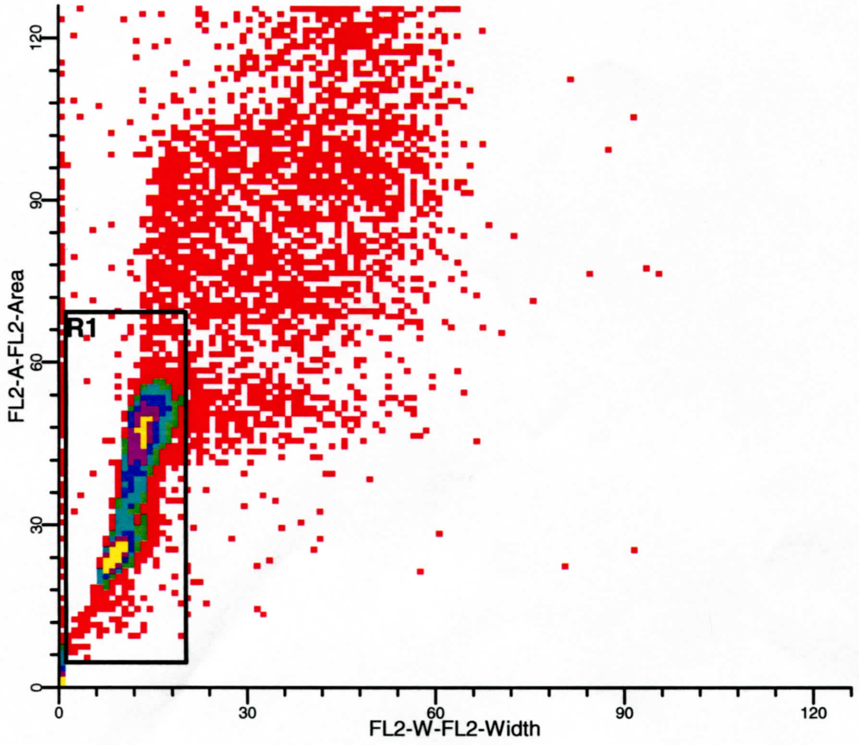

ModFitLT V3.0(Win32)

20 uM VP16-B

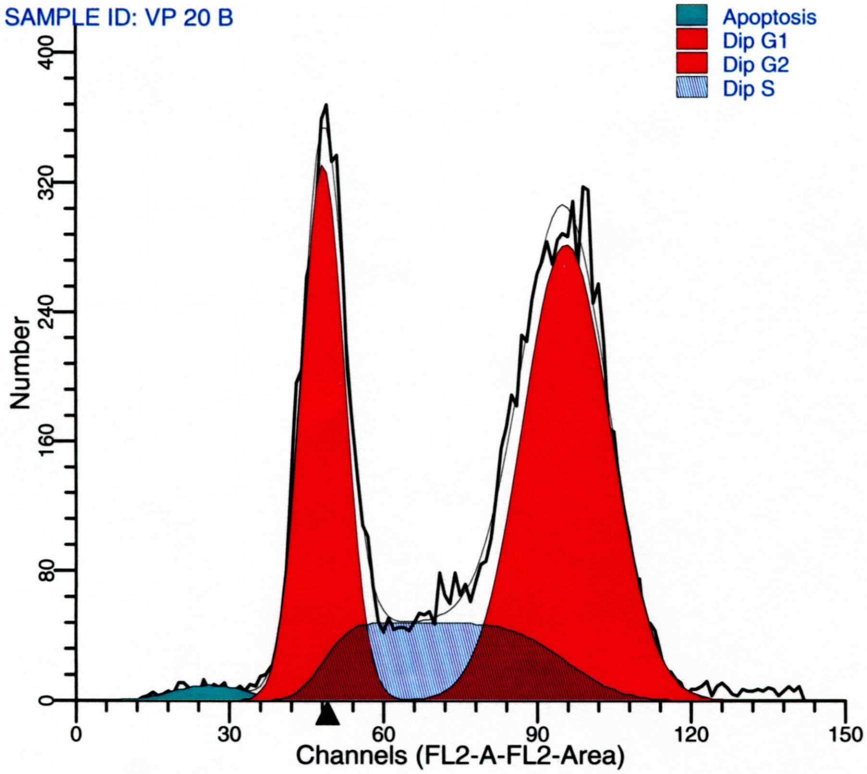

File analyzed: RK22U09.008
Date analyzed: 23-Jun-2009
Model: 1nn0A_DSF
Analysis type: Manual analysis

Diploid: 100.00 %
Dip G1: 29.89 % at 48.25
Dip G2: 50.83 % at 95.69
Dip S: 19.28 % G2/G1: 1.98
%CV: 8.85

Total S-Phase: 19.28 %
Total B.A.D.: 0.00 % no debris no aggs

Apoptosis: 1.40 % Mean: 26.15

Debris: %
Aggregates: 0.00 %
Modeled events: 12054
All cycle events: 11885
Cycle events per channel: 245
RCS: 2.259

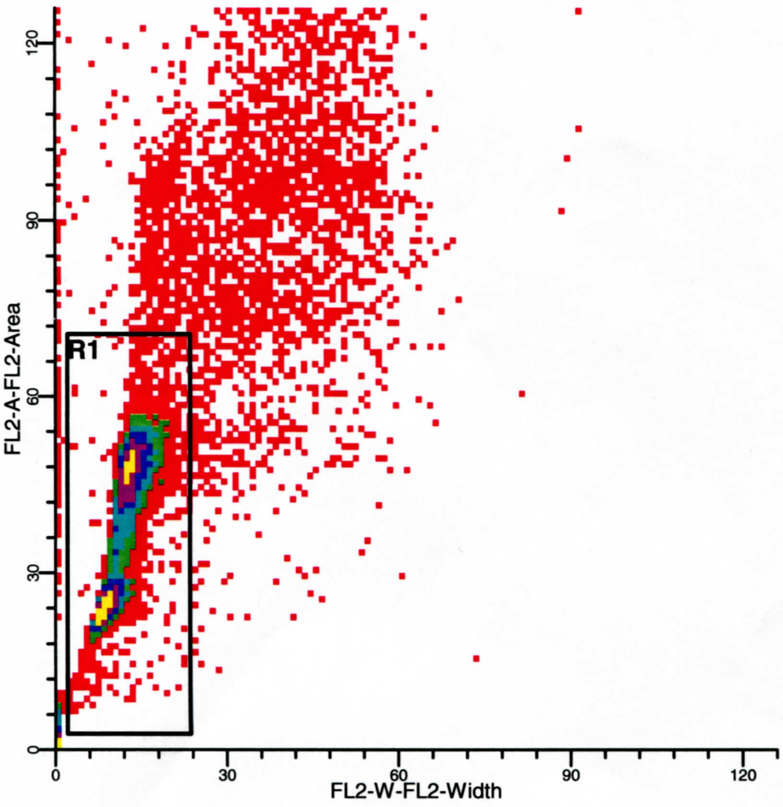

ModFitLT V3.0(Win32)

'40 μM VP16-A

SAMPLE ID: VP 40 A

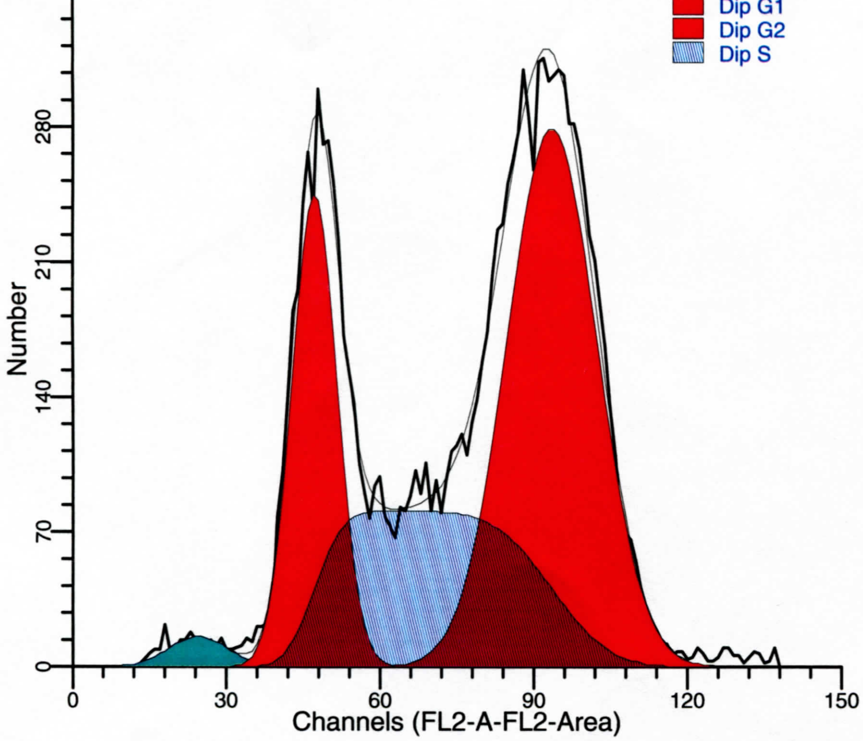

File analyzed: RK22U09.009
Date analyzed: 22-Jun-2009
Model: 1nn0A_DSF
Analysis type: Manual analysis

Diploid: 100.00 %
Dip G1: 21.47 % at 47.24
Dip G2: 48.83 % at 93.59
Dip S: 29.70 % G2/G1: 1.98
%CV: 9.33

Total S-Phase: 29.70 %
Total B.A.D.: 0.00 % no debris no aggs

Apoptosis: 1.77 % Mean: 24.49

Debris: %
Aggregates: 0.00 %
Modeled events: 12874
All cycle events: 12645
Cycle events per channel: 267
RCS: 1.757

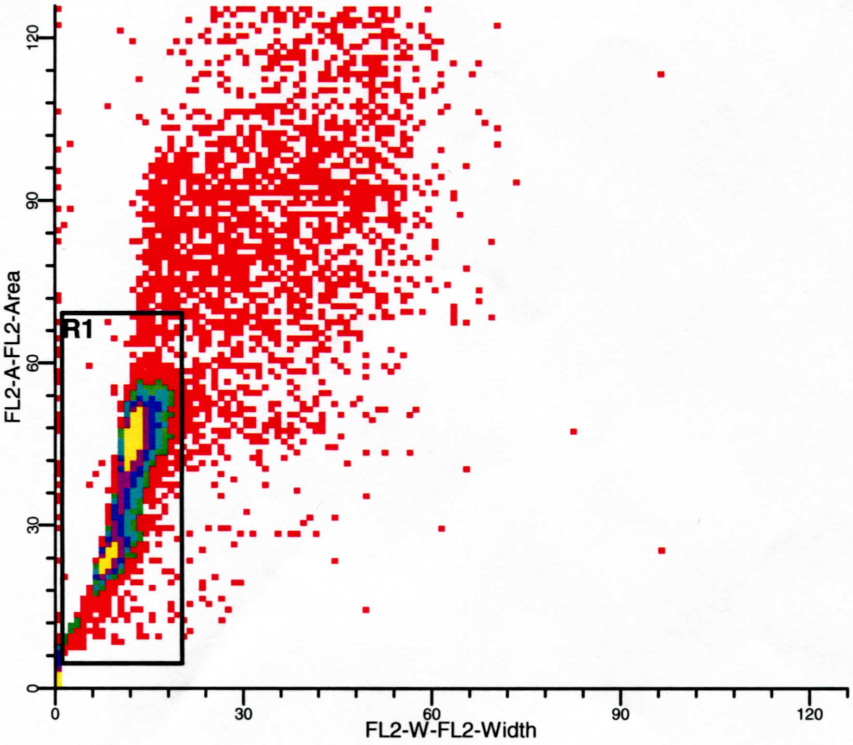

ModFitLT V3.0(Win32)

40 μM VP16-B

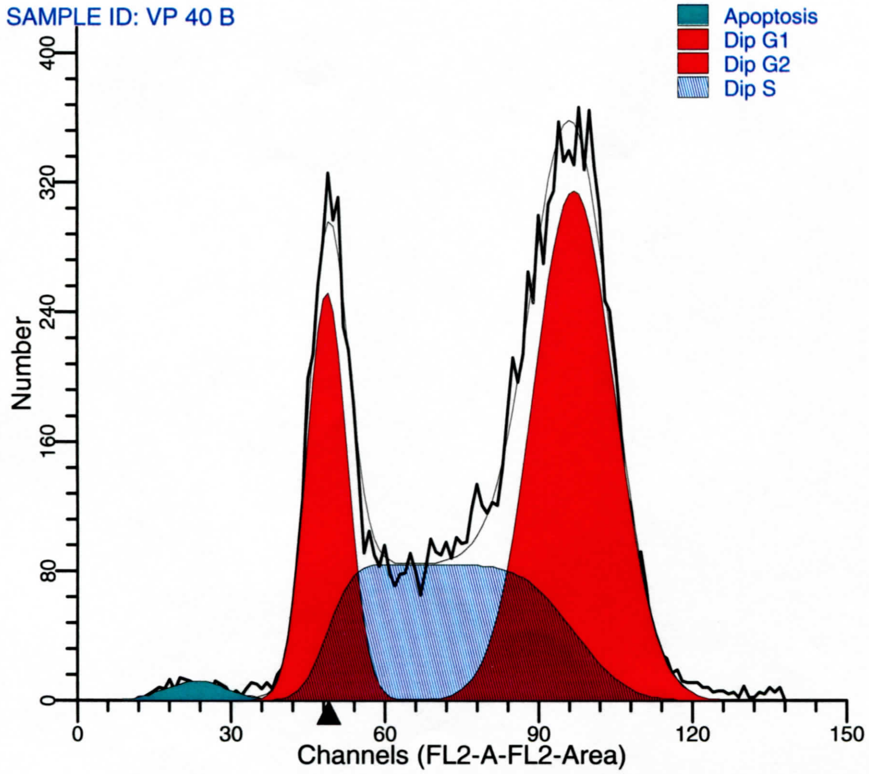

File analyzed: RK22U09.010
Date analyzed: 22-Jun-2009
Model: 1nn0A_DSF
Analysis type: Manual analysis

Diploid: 100.00 %
Dip G1: 19.55 % at 48.76
Dip G2: 48.76 % at 96.94
Dip S: 31.69 % G2/G1: 1.99
%CV: 8.12

Total S-Phase: 31.69 %
Total B.A.D.: 0.00 % no debris no aggs

Apoptosis: 1.37 % Mean: 23.24

Debris: %
Aggregates: 0.00 %
Modeled events: 13020
All cycle events: 12842
Cycle events per channel: 261
RCS: 1.922

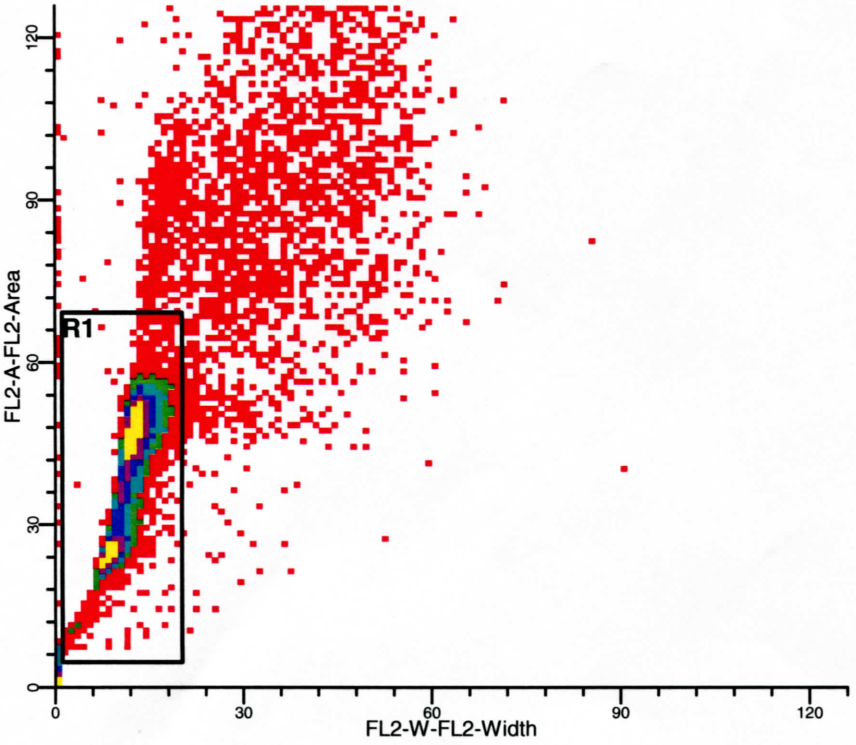

ModFitLT V3.0(Win32)

60µM VP16-A

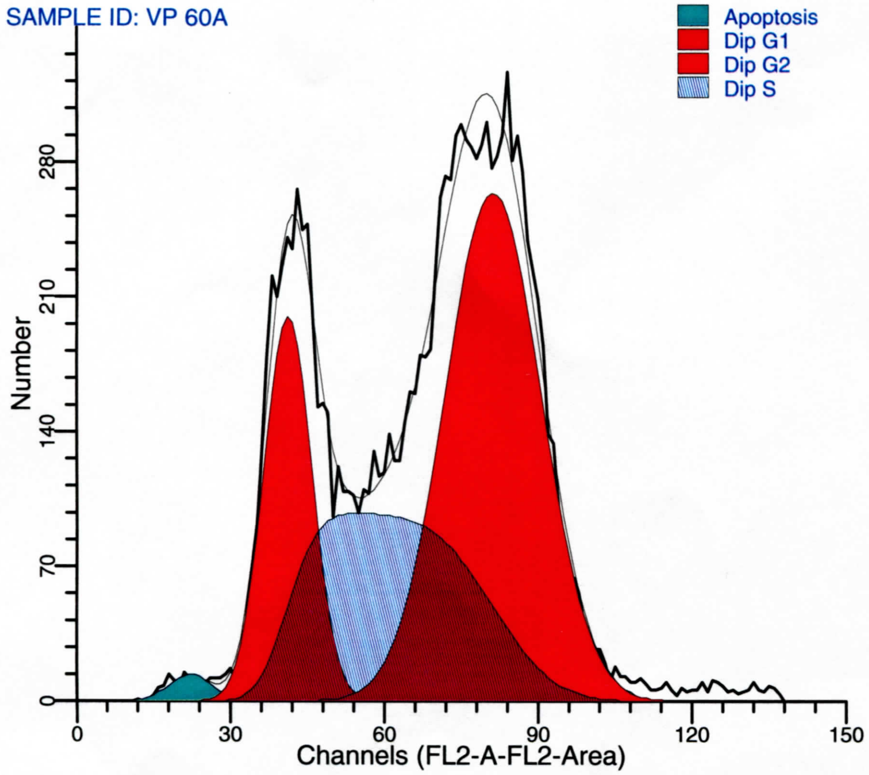

File analyzed: RK22U09.011
Date analyzed: 22-Jun-2009
Model: 1nn0A_DSF
Analysis type: Manual analysis

Diploid: 100.00 %
Dip G1: 18.83 % at 41.16
Dip G2: 49.68 % at 81.21
Dip S: 31.49 % G2/G1: 1.97
%CV: 11.39

Total S-Phase: 31.49 %
Total B.A.D.: 0.00 % no debris no aggs

Apoptosis: 1.13 % Mean: 22.15

Debris: %
Aggregates: 0.00 %
Modeled events: 12651
All cycle events: 12508
Cycle events per channel: 305
RCS: 2.377

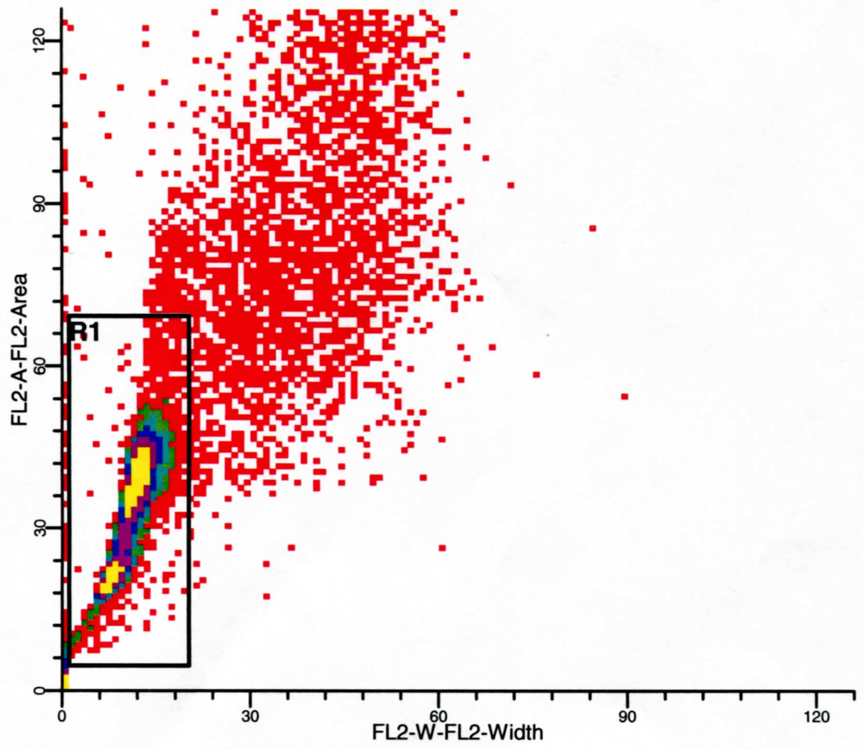

ModFitLT V3.0(Win32)

60 μM VP16-B

SAMPLE ID: VP 60B

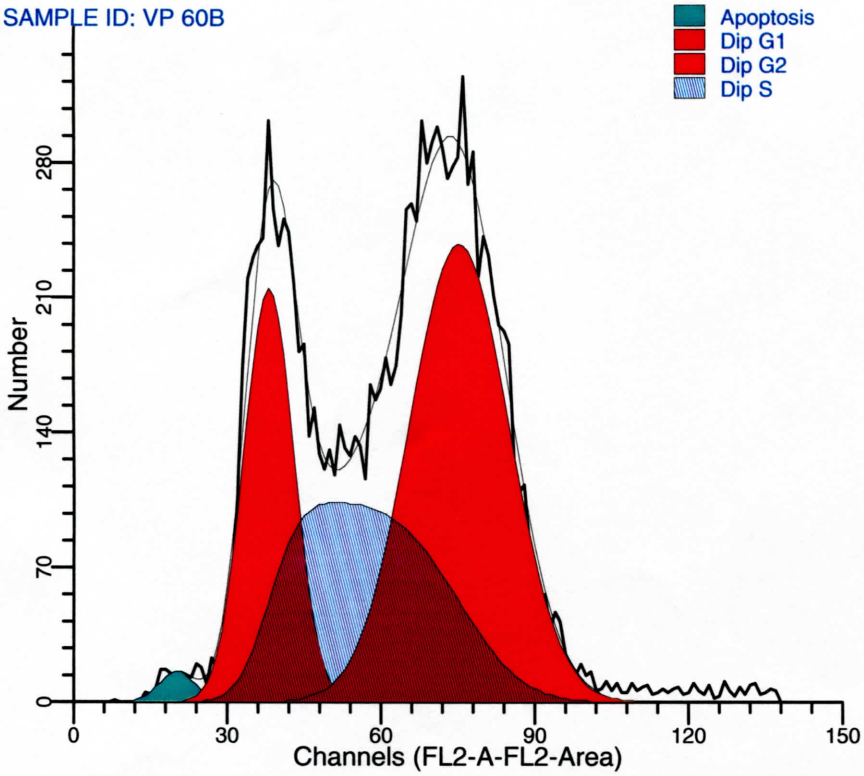

File analyzed: RK22U09.012
Date analyzed: 22-Jun-2009
Model: 1nn0A_DSF
Analysis type: Manual analysis

Diploid: 100.00 %
Dip G1: 21.28 % at 38.12
Dip G2: 47.07 % at 75.26
Dip S: 31.65 % G2/G1: 1.97
%CV: 12.76

Total S-Phase: 31.65 %
Total B.A.D.: 0.00 % no debris no aggs

Apoptosis: 1.10 % Mean: 20.20

Debris: %
Aggregates: 0.00 %
Modeled events: 12462
All cycle events: 12325
Cycle events per channel: 323
RCS: 2.656

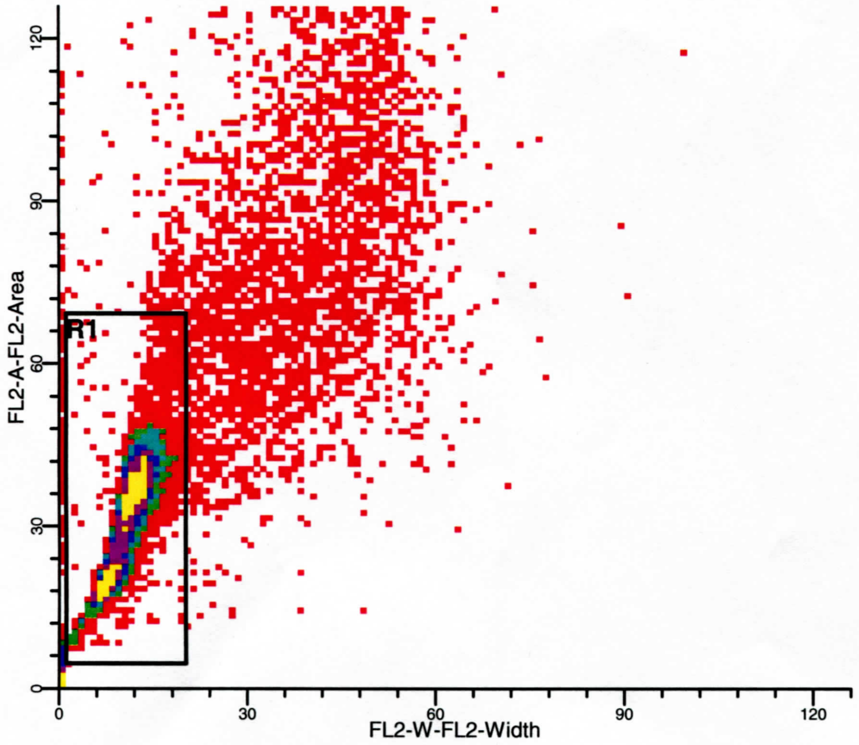

ModFitLT V3.0(Win32)

80MM VP16-A

SAMPLE ID: VP 80A

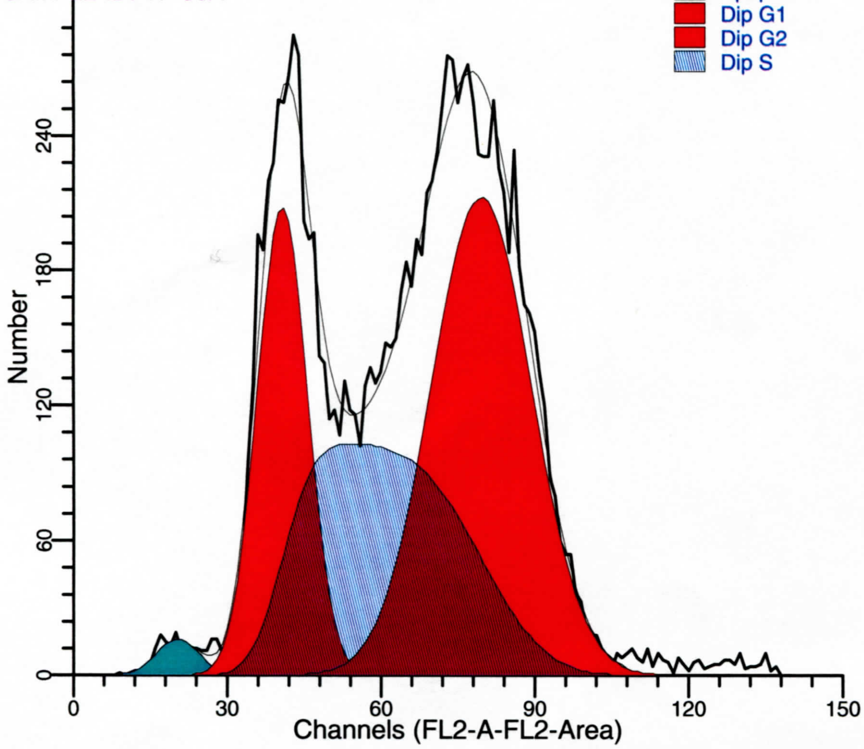

File analyzed: RK22U09.013
Date analyzed: 22-Jun-2009
Model: 1nn0A_DSF
Analysis type: Manual analysis

Diploid: 100.00 %
Dip G1: 21.66 % at 40.75
Dip G2: 44.17 % at 79.59
Dip S: 34.17 % G2/G1: 1.95
%CV: 12.02

Total S-Phase: 34.17 %
Total B.A.D.: 0.00 % no debris no aggs

Apoptosis: 1.39 % Mean: 20.37

Debris: %
Aggregates: 0.00 %
Modeled events: 12001
All cycle events: 11835
Cycle events per channel: 297
RCS: 2.135

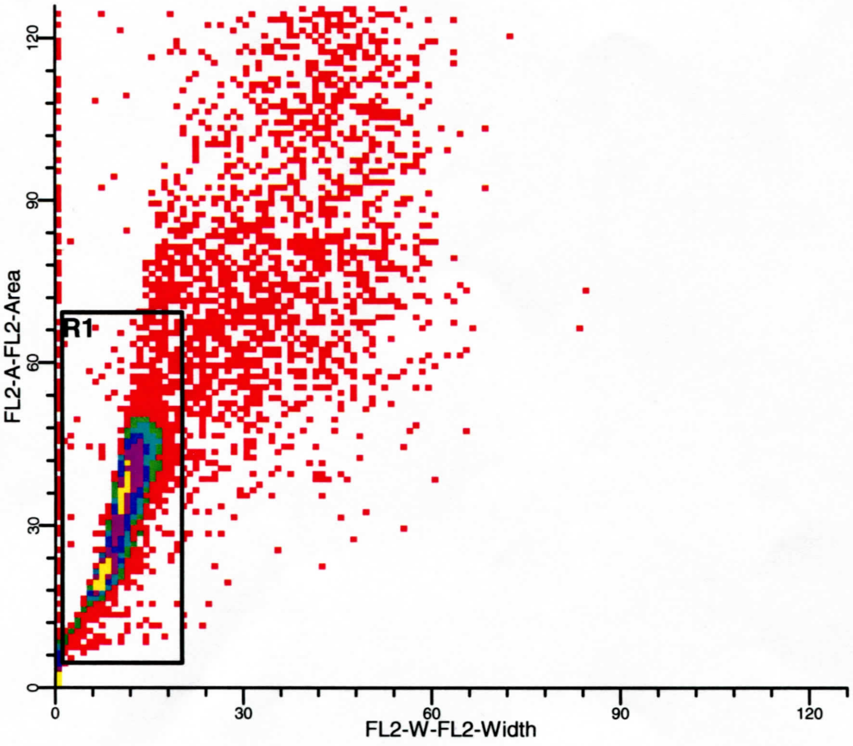

ModFitLT V3.0(Win32)

80MM VP16-B

SAMPLE ID: VP 80B

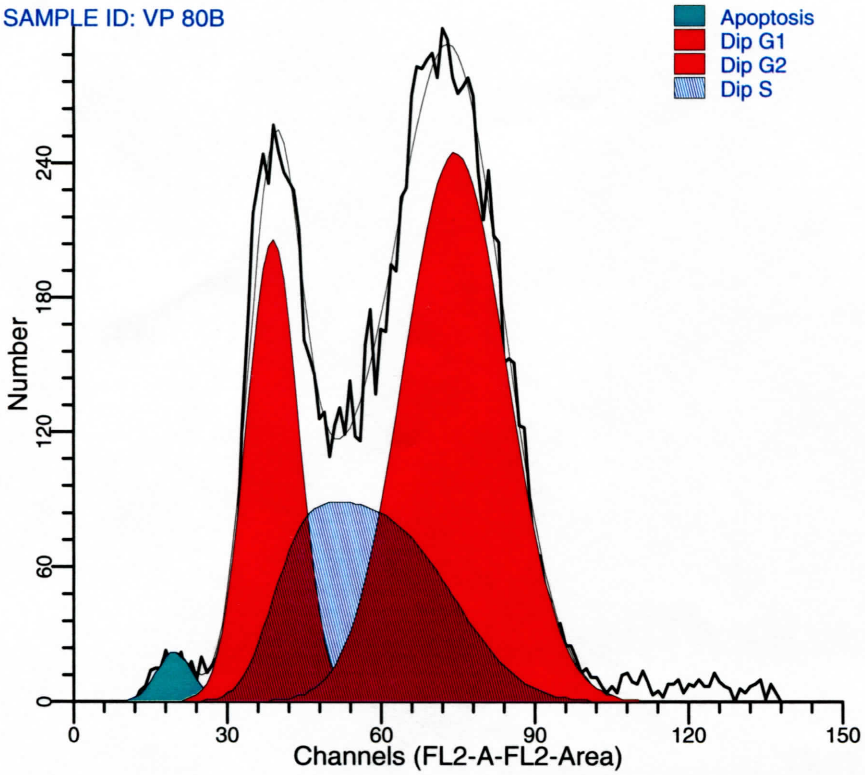

File analyzed: RK22U09.014
Date analyzed: 22-Jun-2009
Model: 1nn0A_DSF
Analysis type: Manual analysis

Diploid: 100.00 %
Dip G1: 21.69 % at 38.83
Dip G2: 51.45 % at 74.47
Dip S: 26.86 % G2/G1: 1.92
%CV: 13.01

Total S-Phase: 26.86 %
Total B.A.D.: 0.00 % no debris no aggs

Apoptosis: 1.56 % Mean: 19.67

Debris: %
Aggregates: 0.00 %
Modeled events: 12240
All cycle events: 12049
Cycle events per channel: 329
RCS: 2.449

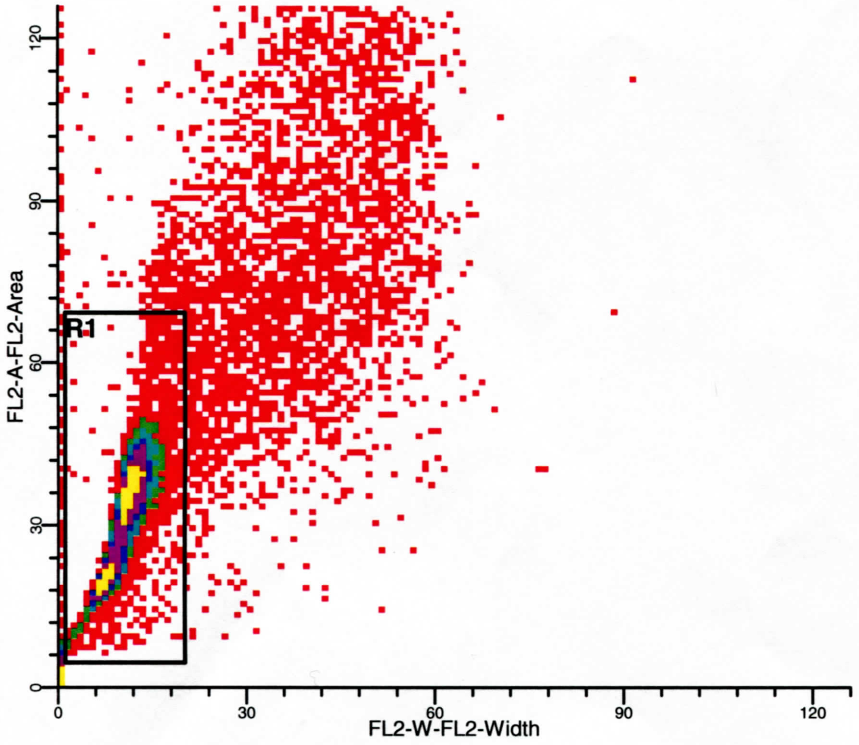

ModFitLT V3.0(Win32)

100 μM VP16-A

SAMPLE ID: VP 100A

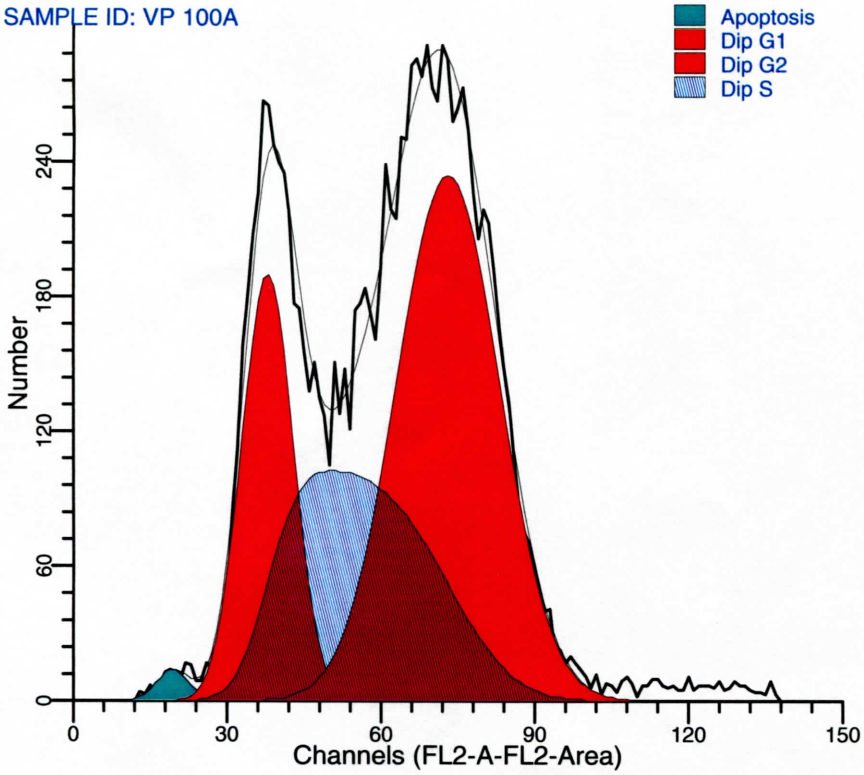

File analyzed: RK22U09.015
Date analyzed: 22-Jun-2009
Model: 1nn0A_DSF
Analysis type: Manual analysis

Diploid: 100.00 %
Dip G1: 20.03 % at 37.85
Dip G2: 49.25 % at 73.07
Dip S: 30.72 % G2/G1: 1.93
%CV: 13.29

Total S-Phase: 30.72 %
Total B.A.D.: 0.00 % no debris no aggs

Apoptosis: 0.92 % Mean: 19.44

Debris: %
Aggregates: 0.00 %
Modeled events: 12108
All cycle events: 11996
Cycle events per channel: 331
RCS: 2.441

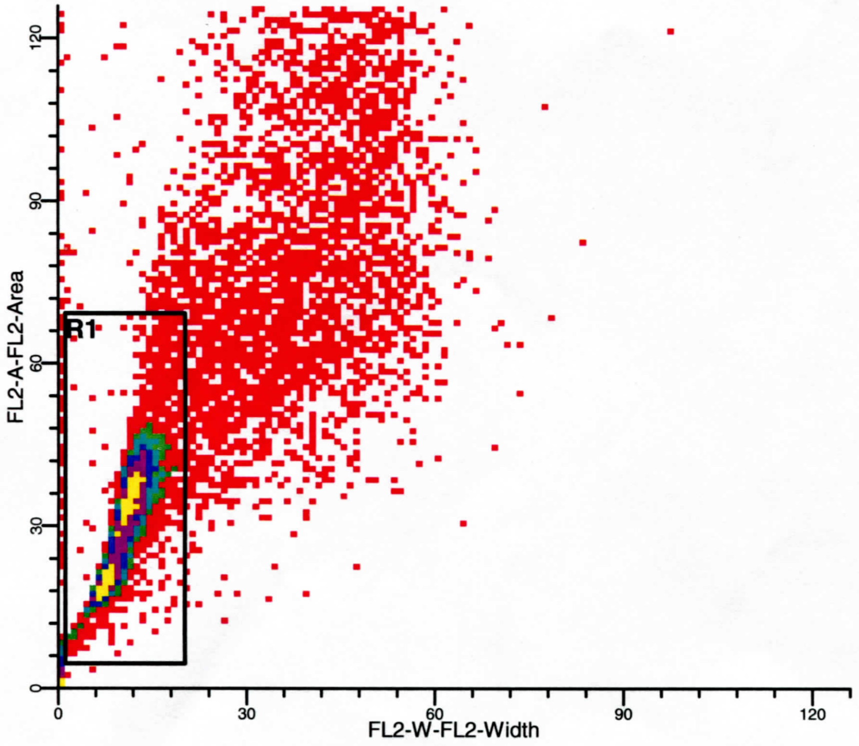

ModFitLT V3.0(Win32)

100MM VP16-B

SAMPLE ID: VP 100B

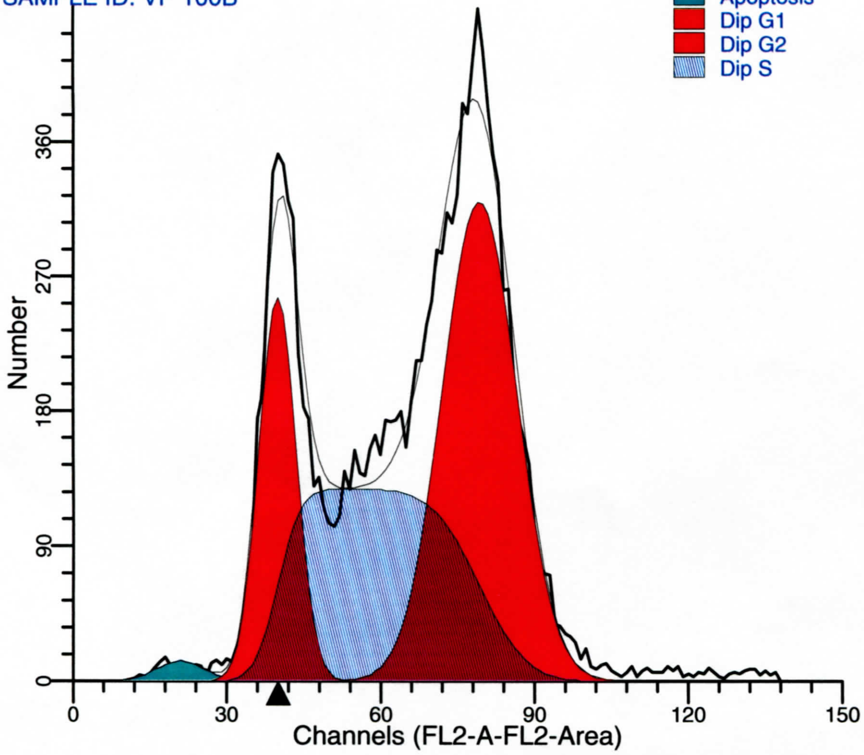

File analyzed: RK22U09.016
Date analyzed: 22-Jun-2009
Model: 1nn0A_DSF
Analysis type: Manual analysis

Diploid: 100.00 %
Dip G1: 17.68 % at 39.91
Dip G2: 44.17 % at 79.29
Dip S: 38.15 % G2/G1: 1.99
%CV: 9.10

Total S-Phase: 38.15 %
Total B.A.D.: 0.00 % no debris no aggs

Apoptosis: 1.17 % Mean: 20.98

Debris: %
Aggregates: 0.00 %
Modeled events: 13385
All cycle events: 13228
Cycle events per channel: 328
RCS: 3.075

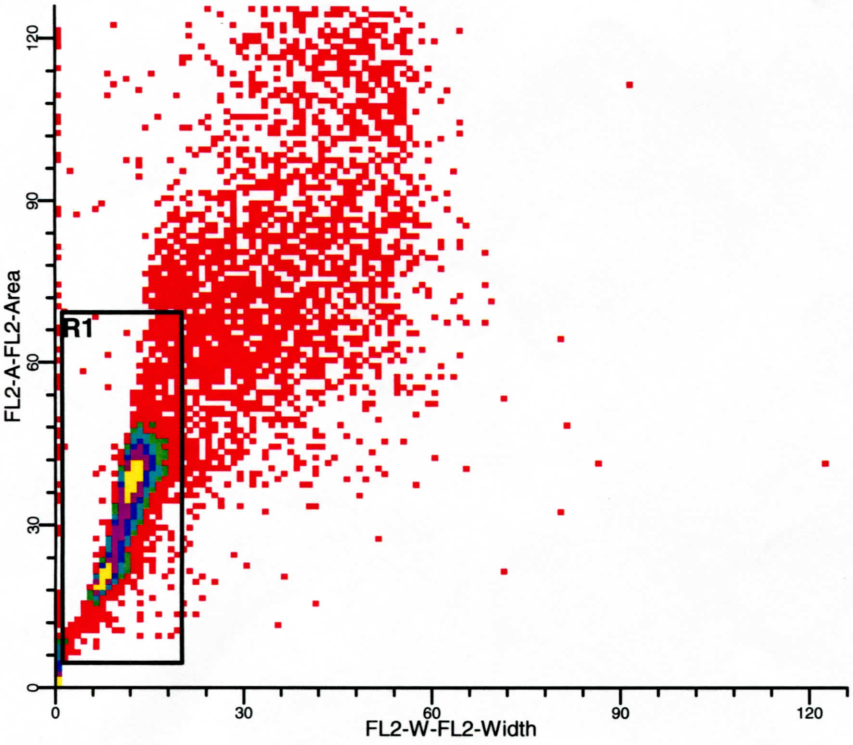

ModFitLT V3.0(Win32)
