# Supplementary material for: Correction: ERK1/2 Signaling Plays an Important Role in Topoisomerase II Poison-Induced G2/M Checkpoint Activation
Source: PLoS One. 2023 Sep 28;18(9):e0292423. doi: 10.1371/journal.pone.0292423 (PMC10538782; doi:10.1371/journal.pone.0292423)
Supplement: S1 File — (ZIP) [file pone.0292423.s001.zip › Figure 1A/LOG.pdf]

None, MCF-7

SAMPLE ID: Log B

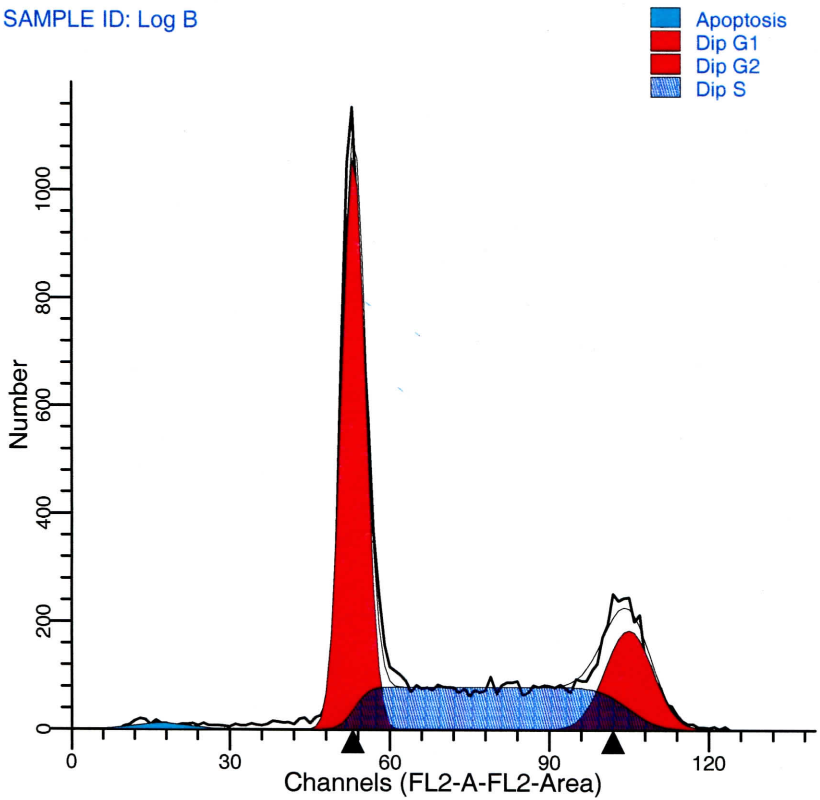

File analyzed: RK27U09.002
Date analyzed: 3-Sep-2009
Model: 1nn0A_DSF
Analysis type: Manual analysis

Diploid: 100.00 %
Dip G1: 51.21 % at 53.26
Dip G2: 16.09 % at 104.93
Dip S: 31.80 % G2/G1: 1.97
%CV: 4.16

Total S-Phase: 31.80 %
Total B.A.D.: 0.00 % no debris no aggs

Apoptosis: 1.29 % Mean: 17.10

Debris: %
Aggregates: 0.00 %
Modeled events: 12294
All cycle events: 12136
Cycle events per channel: 230
RCS: 3.985
