# Supplementary figures and images for: Correction: ERK1/2 Signaling Plays an Important Role in Topoisomerase II Poison-Induced G2/M Checkpoint Activation
Source: PLoS One. 2023 Sep 28;18(9):e0292423. doi: 10.1371/journal.pone.0292423 (PMC10538782; doi:10.1371/journal.pone.0292423)

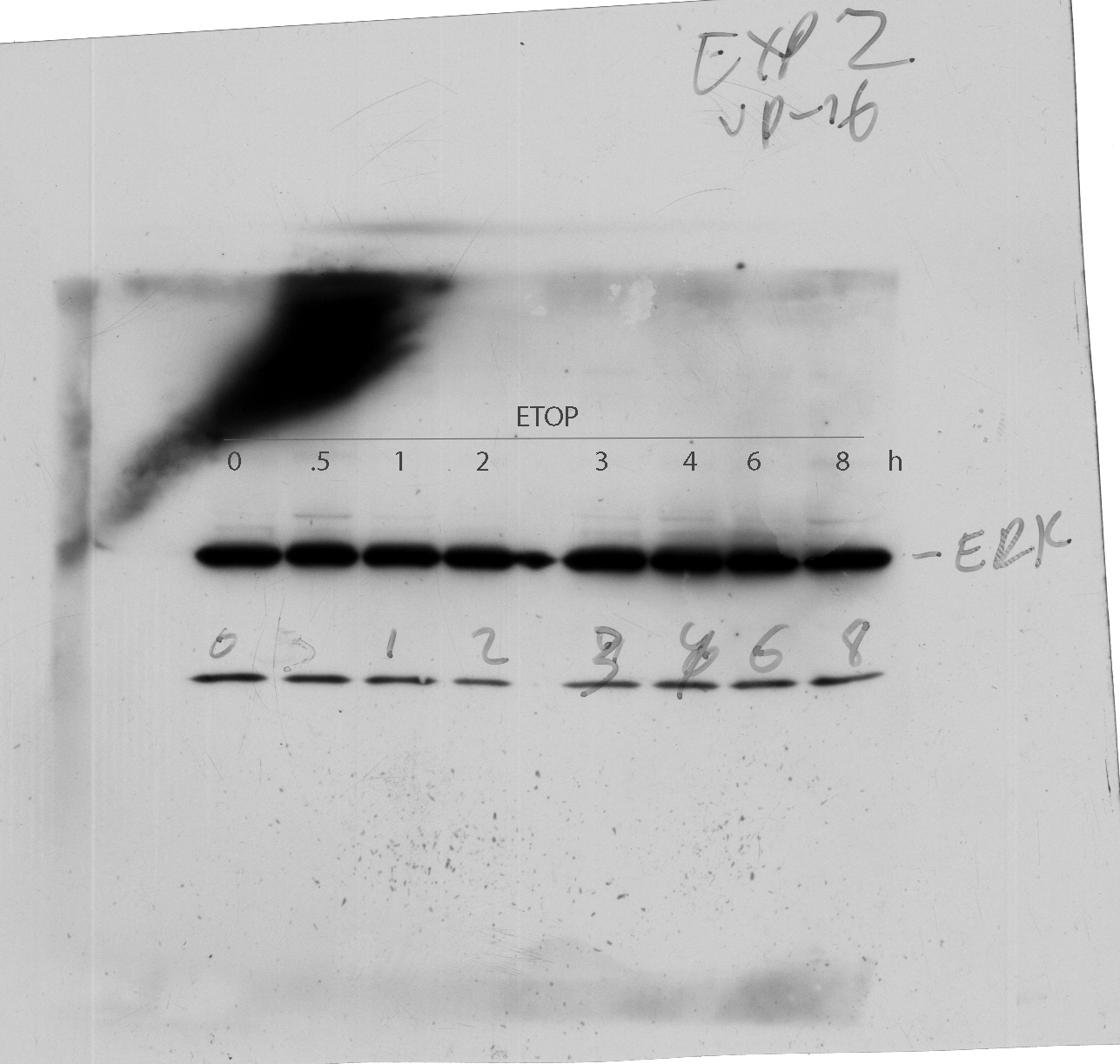

Supplement: S1 File — (ZIP) [file pone.0292423.s001.zip › Figure 1B/Fig 1B Etop- ERK-F.tif]

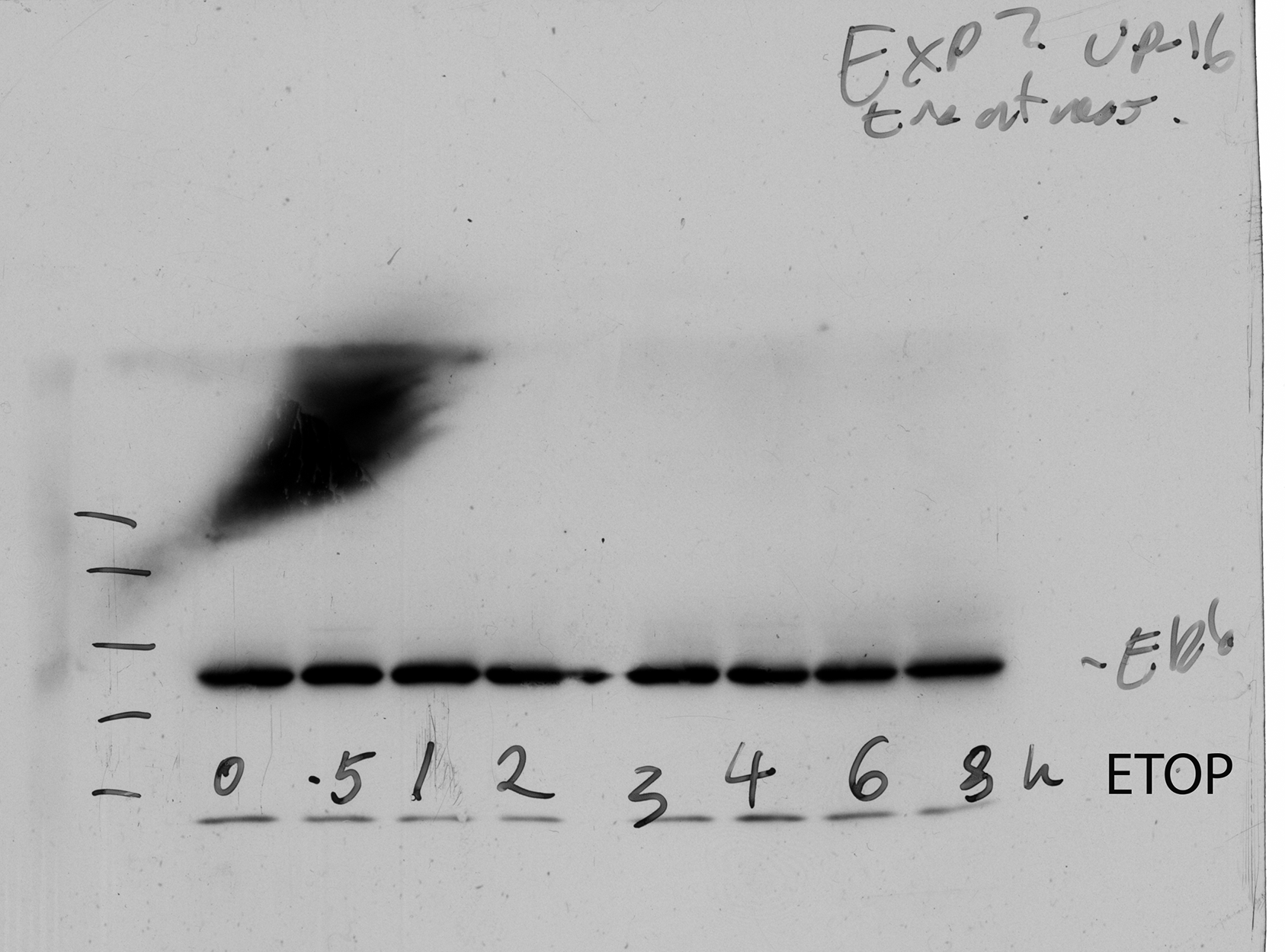

Supplement: S1 File — (ZIP) [file pone.0292423.s001.zip › Figure 1B/Fig 1B Etop-ERK-F2.tif]

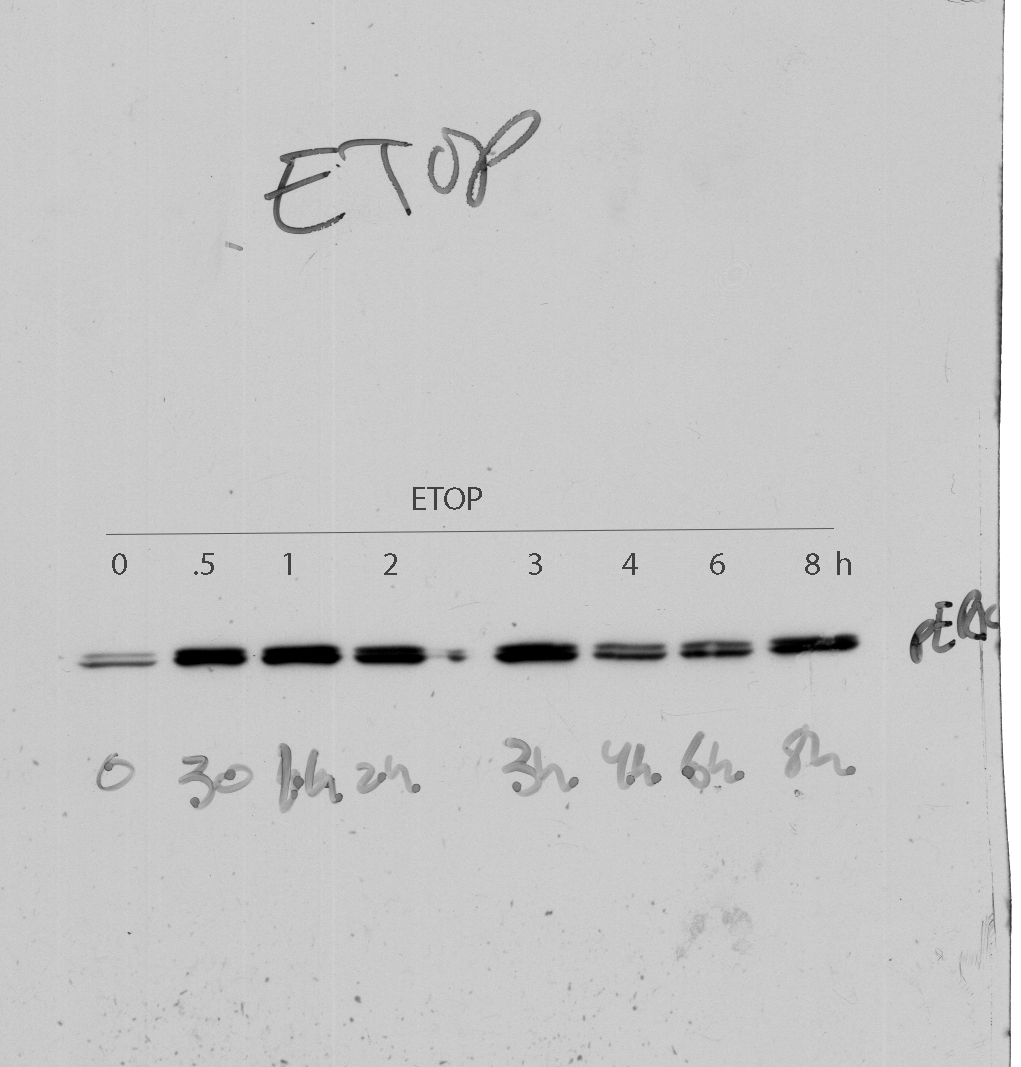

Supplement: S1 File — (ZIP) [file pone.0292423.s001.zip › Figure 1B/Fig 1B ETOP-pERK-F.tif]

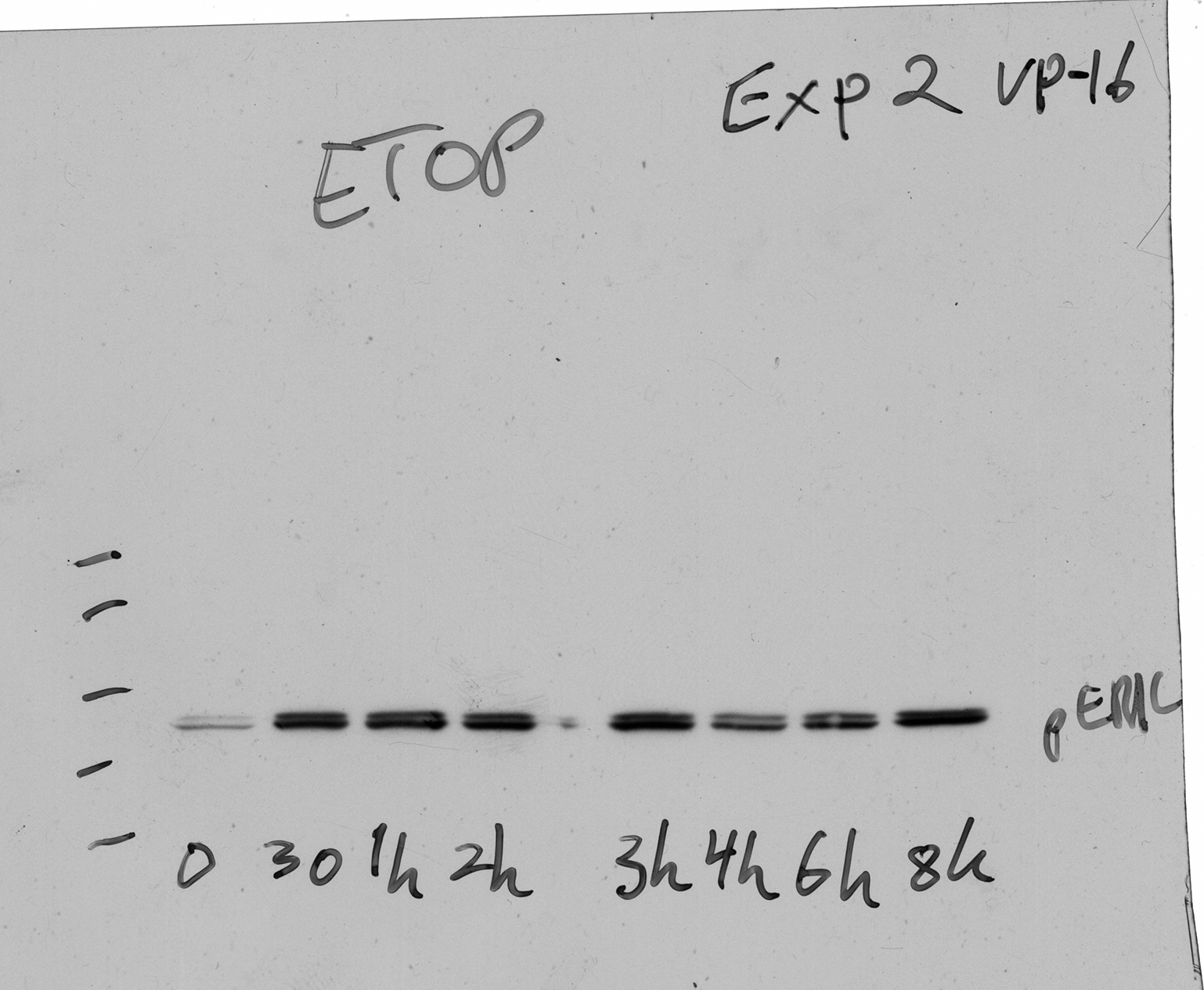

Supplement: S1 File — (ZIP) [file pone.0292423.s001.zip › Figure 1B/Fig 1B Etop-pERK-F2.tif]

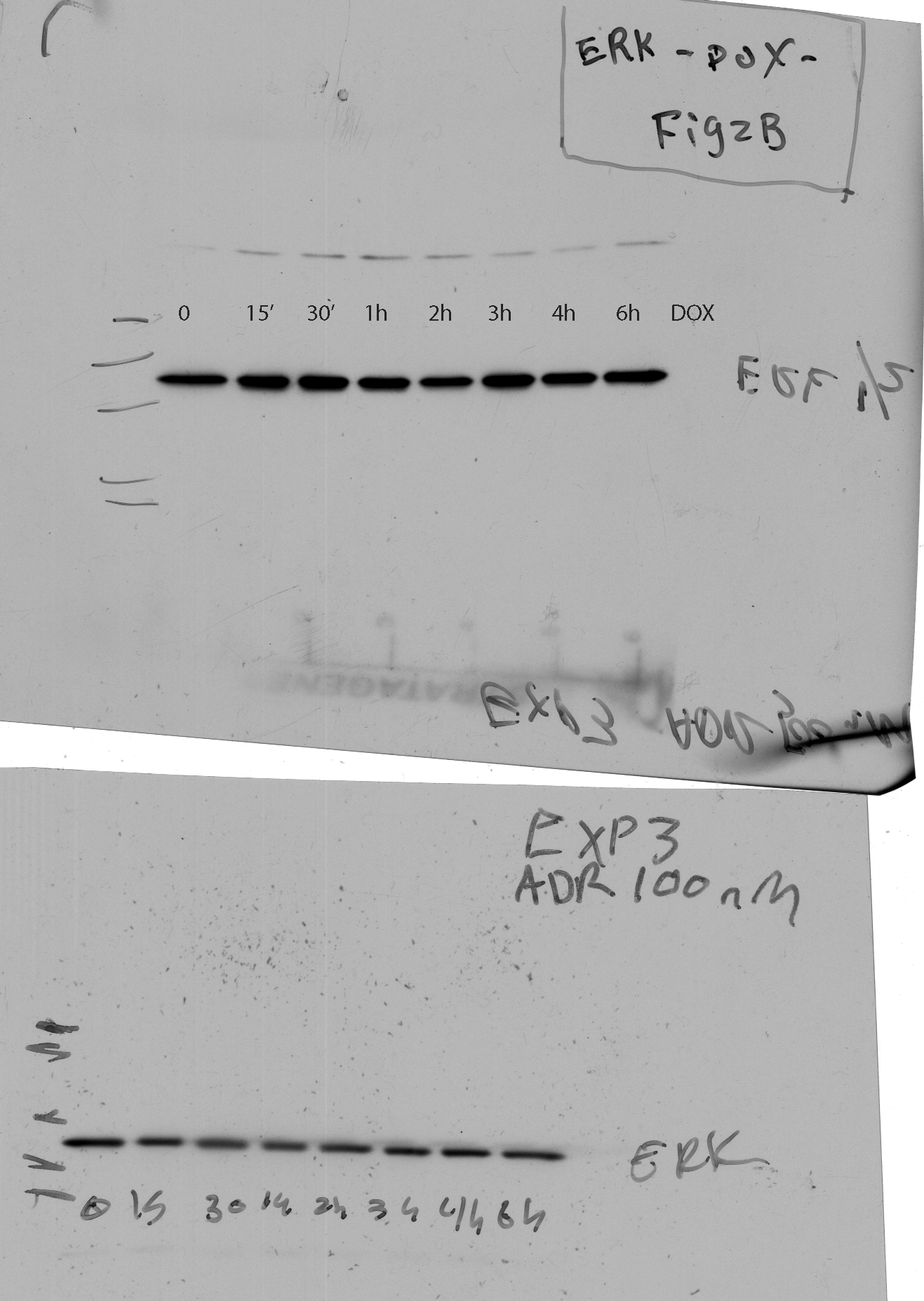

Supplement: S1 File — (ZIP) [file pone.0292423.s001.zip › Figure 1B/Fig 1B, DOX-ERK.tif]

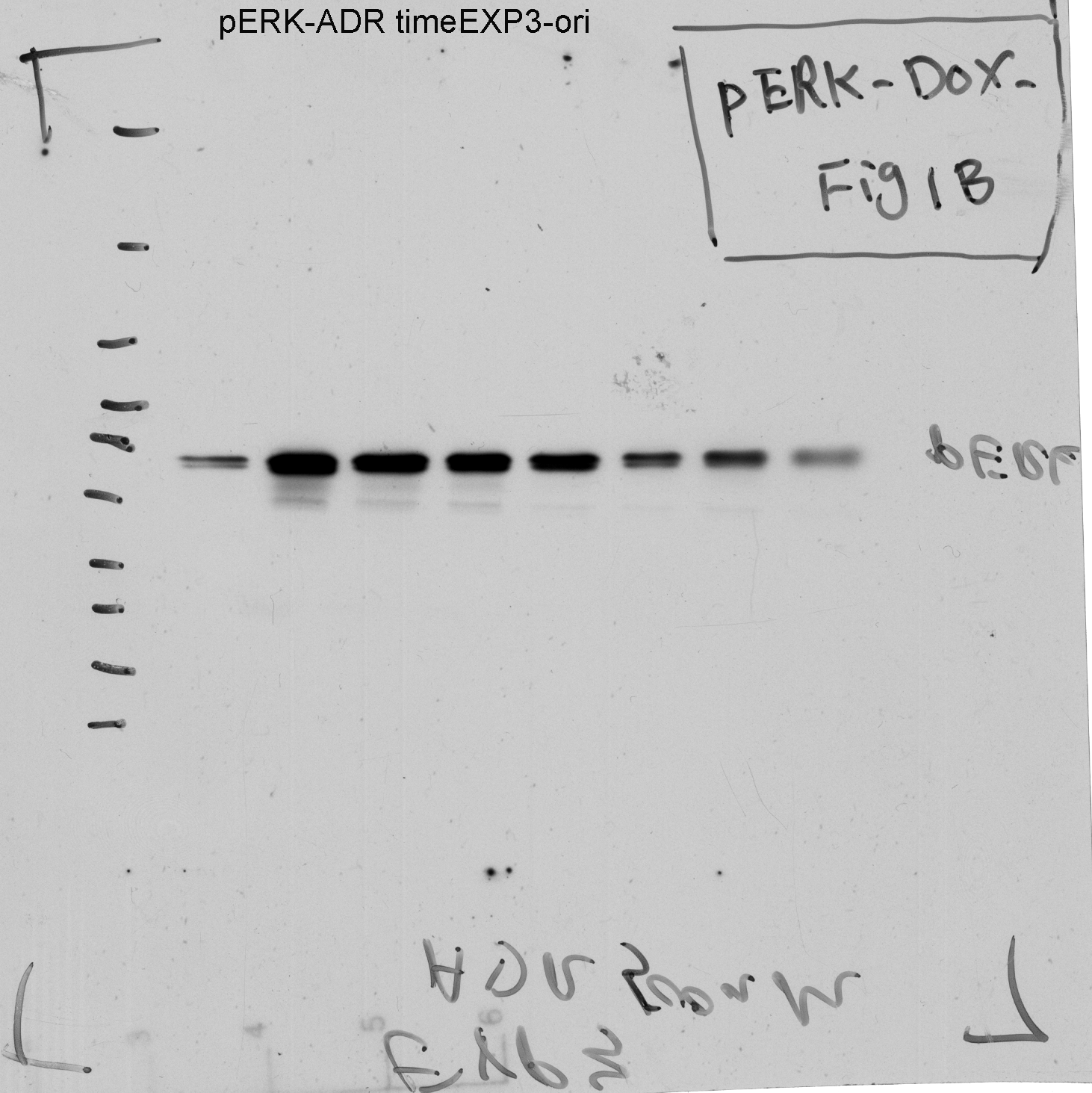

Supplement: S1 File — (ZIP) [file pone.0292423.s001.zip › Figure 1B/pERK-ADR timeEXP3-ori.tif]

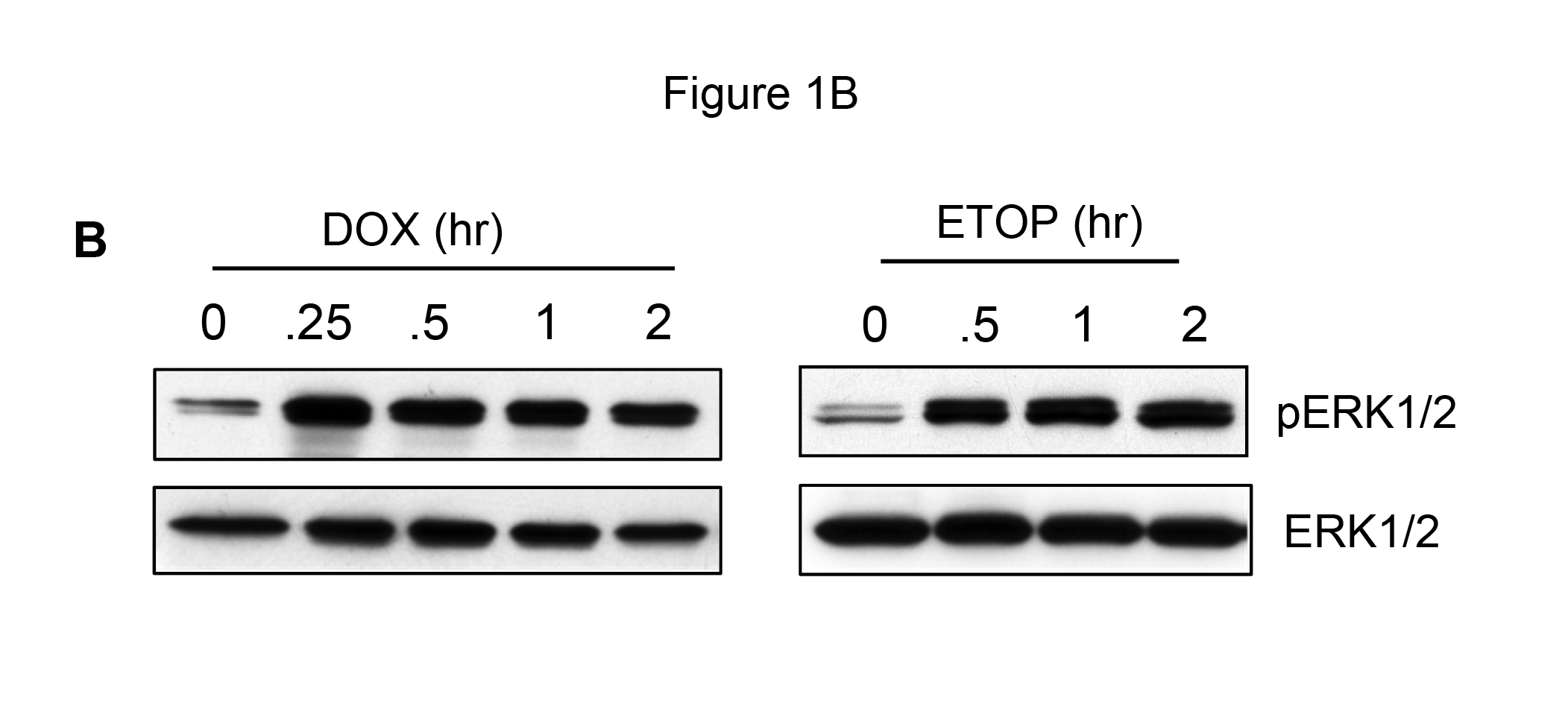

Supplement: S1 File — (ZIP) [file pone.0292423.s001.zip › Figure 1B/Revised Fig 1B.tif]

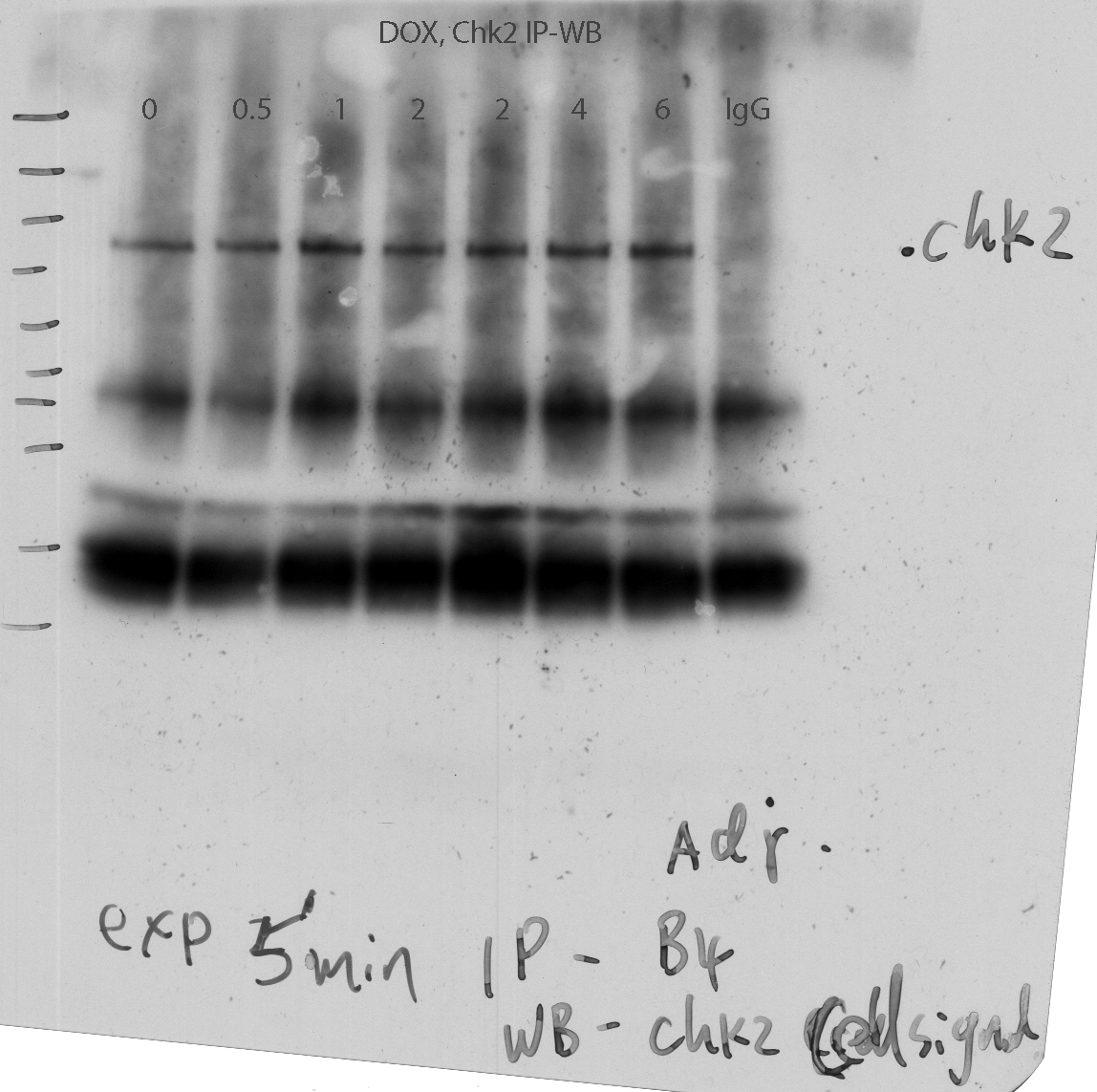

Supplement: S2 File — Note that the underlying blot for Fig 2A ETOP Chk1 IP-WB may be a different exposure than that used for the figure. (ZIP) [file pone.0292423.s002.zip › S2 File/Figure 2/Fig 2A DOX chk2 IP-WB F.tif]

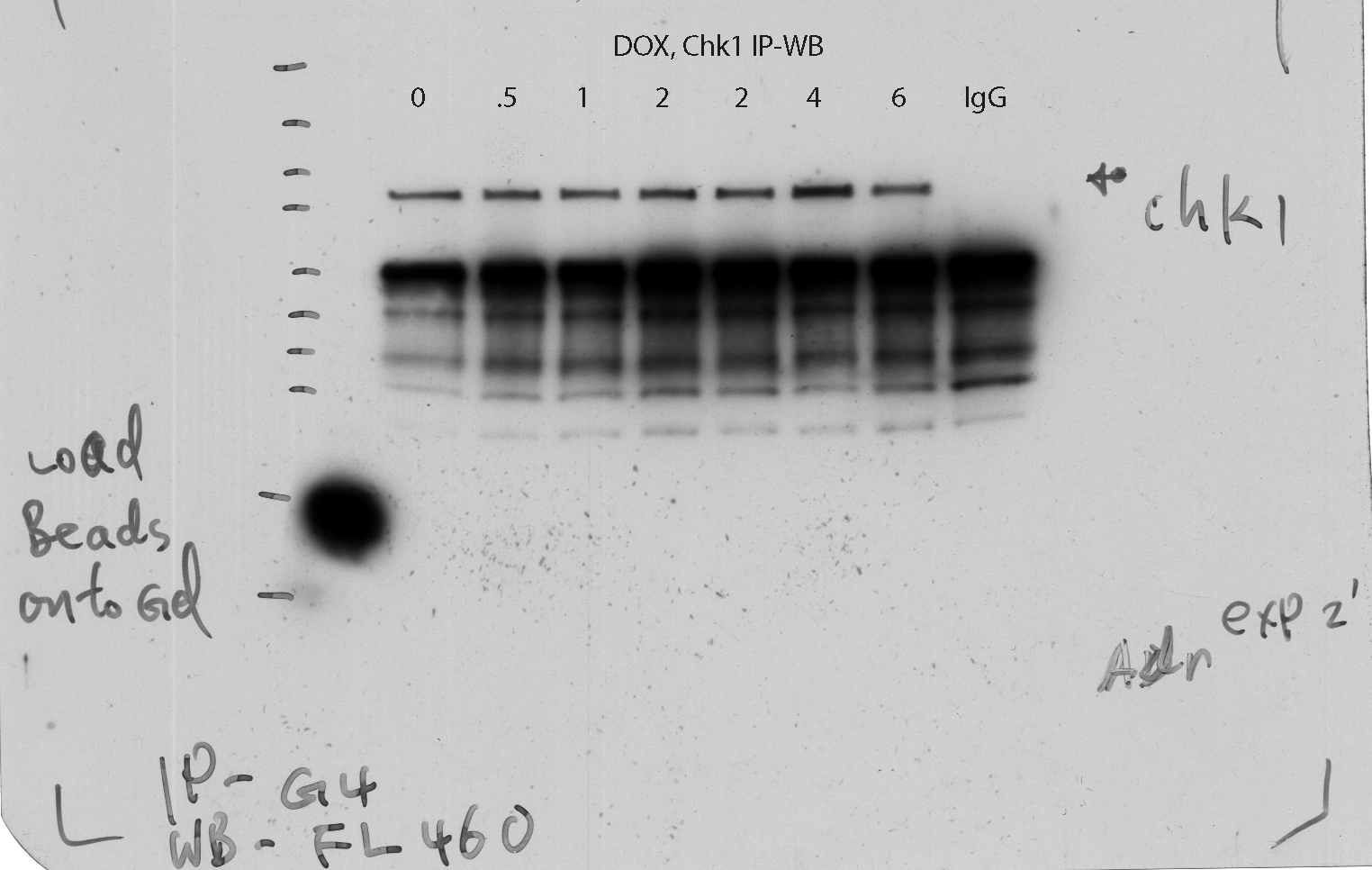

Supplement: S2 File — Note that the underlying blot for Fig 2A ETOP Chk1 IP-WB may be a different exposure than that used for the figure. (ZIP) [file pone.0292423.s002.zip › S2 File/Figure 2/Fig 2A DOX-Chk1 IP-WB F.tif]

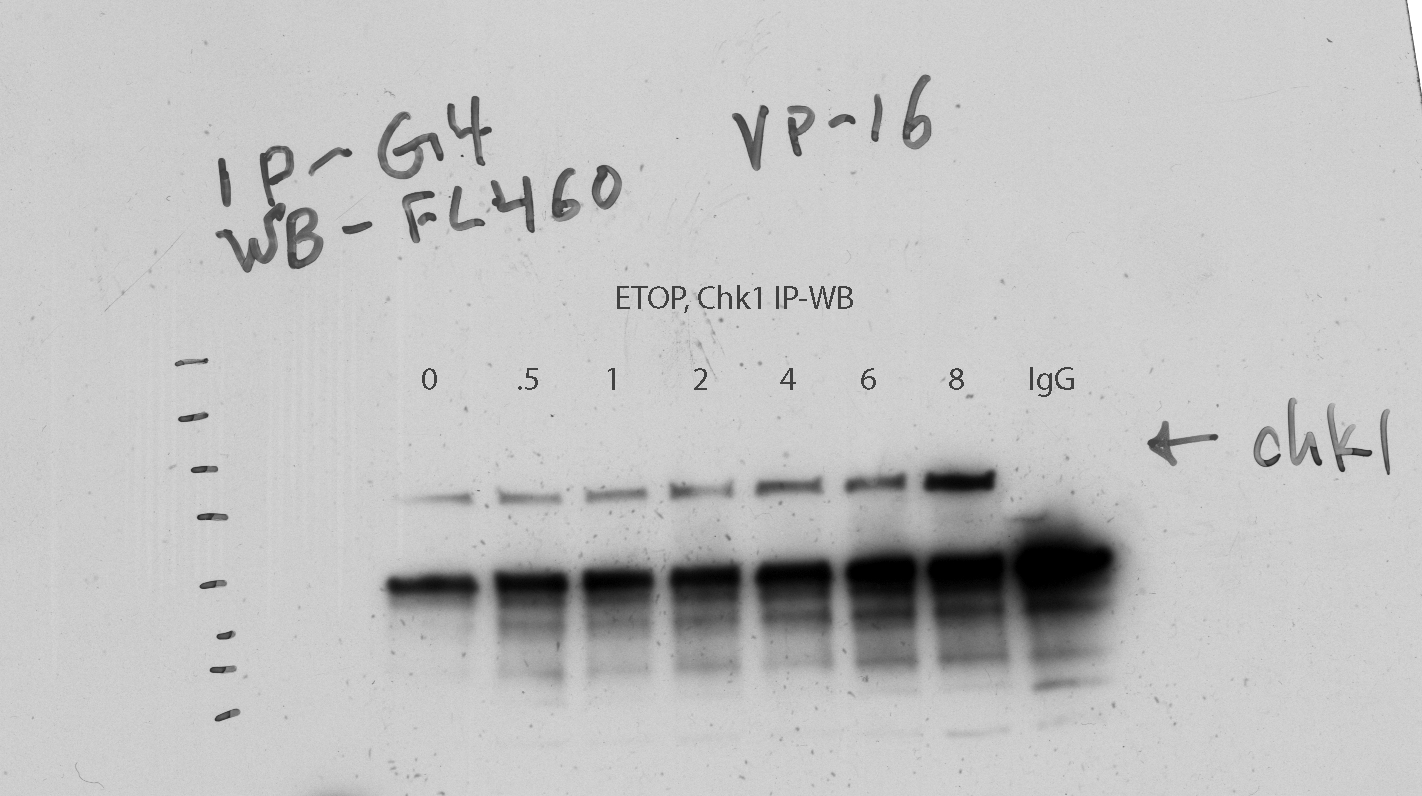

Supplement: S2 File — Note that the underlying blot for Fig 2A ETOP Chk1 IP-WB may be a different exposure than that used for the figure. (ZIP) [file pone.0292423.s002.zip › S2 File/Figure 2/Fig 2A ETOP Chk1 IP-WB F.tif]

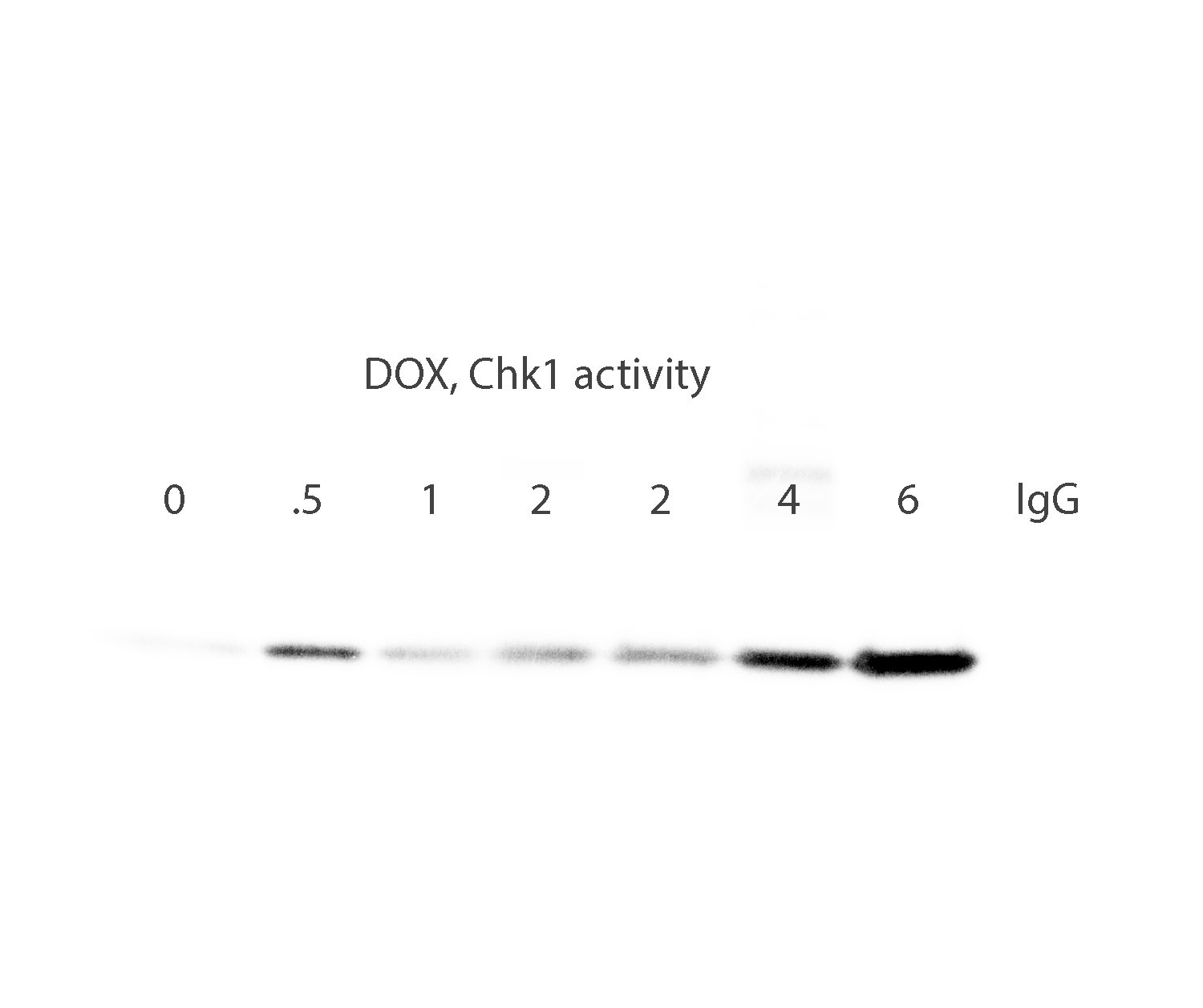

Supplement: S2 File — Note that the underlying blot for Fig 2A ETOP Chk1 IP-WB may be a different exposure than that used for the figure. (ZIP) [file pone.0292423.s002.zip › S2 File/Figure 2/Fig 2A, Dox, Chk1-Activity-labled.tif]

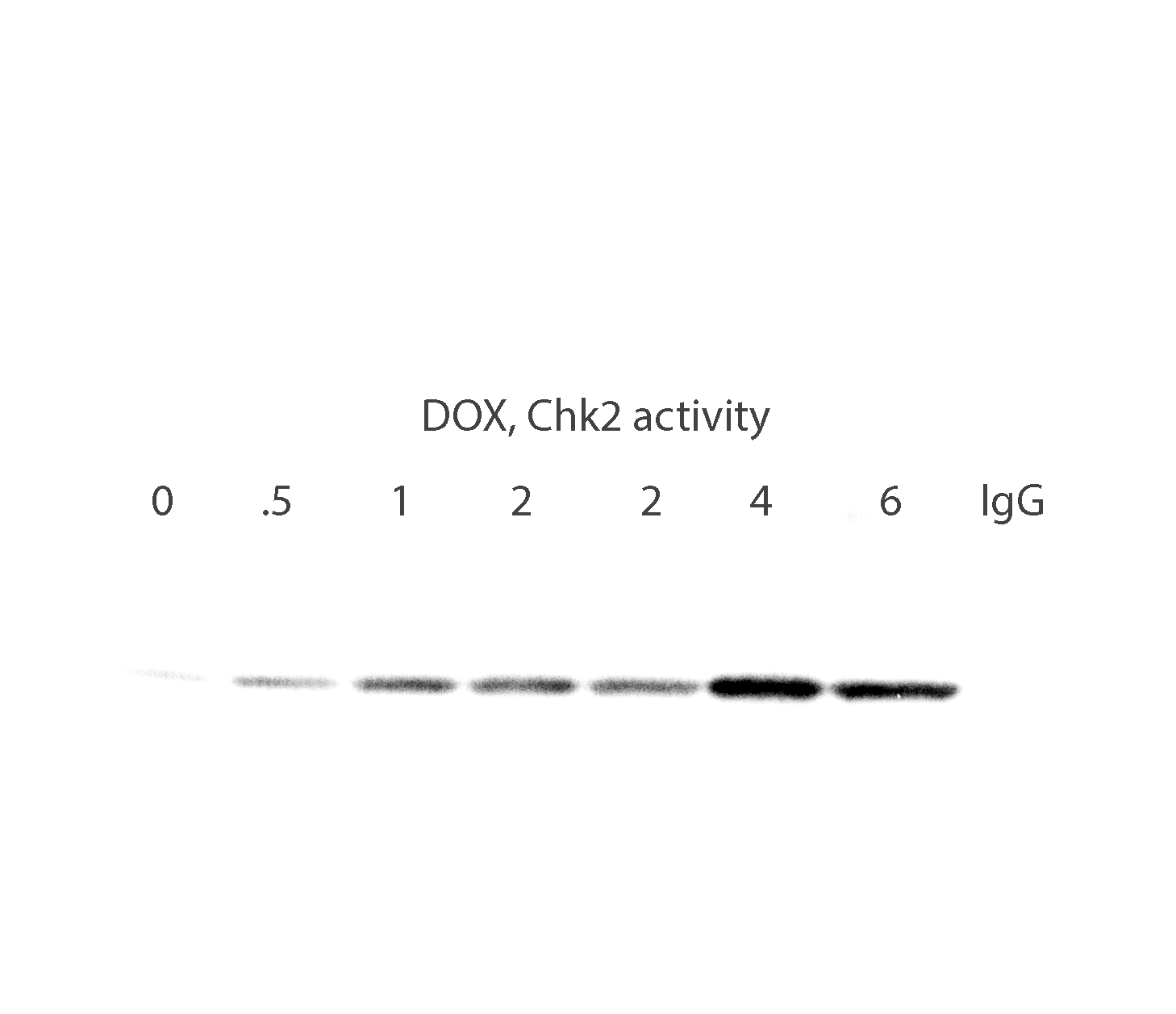

Supplement: S2 File — Note that the underlying blot for Fig 2A ETOP Chk1 IP-WB may be a different exposure than that used for the figure. (ZIP) [file pone.0292423.s002.zip › S2 File/Figure 2/Fig 2A, Dox, Chk2-Activity, labled.tif]

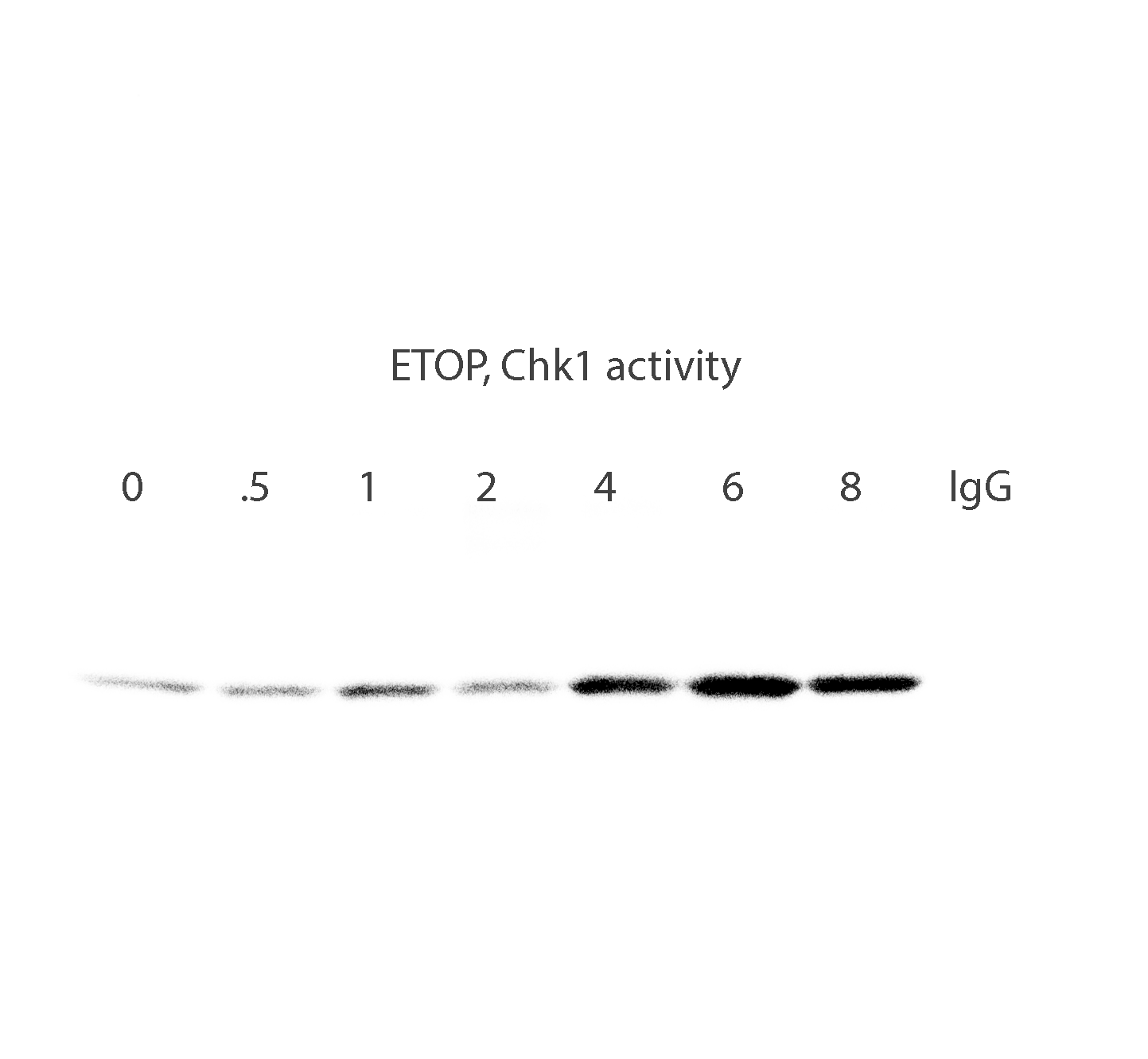

Supplement: S2 File — Note that the underlying blot for Fig 2A ETOP Chk1 IP-WB may be a different exposure than that used for the figure. (ZIP) [file pone.0292423.s002.zip › S2 File/Figure 2/Fig 2A, ETOP, Chk1-activity, labeled.tif]

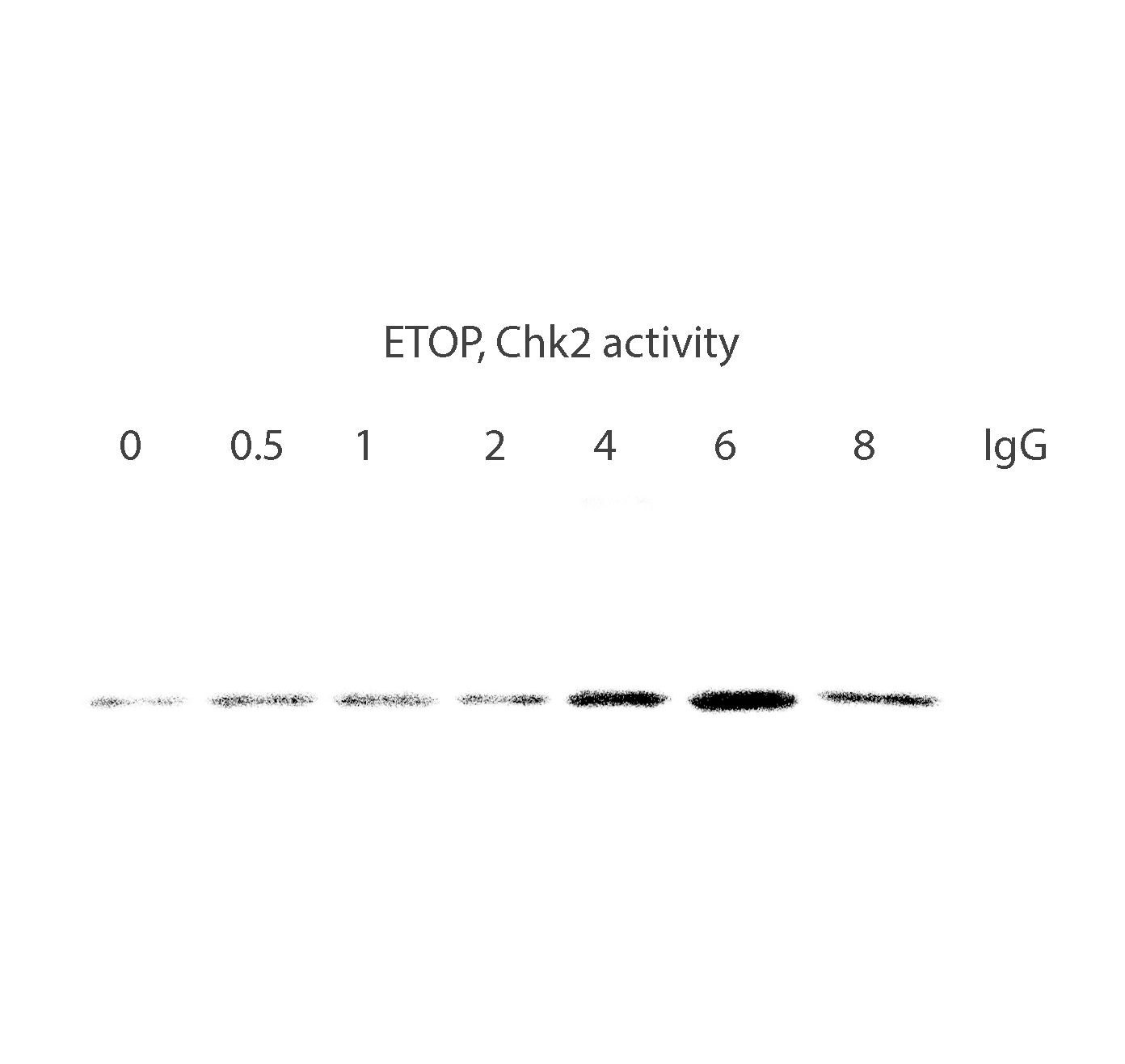

Supplement: S2 File — Note that the underlying blot for Fig 2A ETOP Chk1 IP-WB may be a different exposure than that used for the figure. (ZIP) [file pone.0292423.s002.zip › S2 File/Figure 2/Fig 2A, ETOP, Chk2-activity-labled.tif]

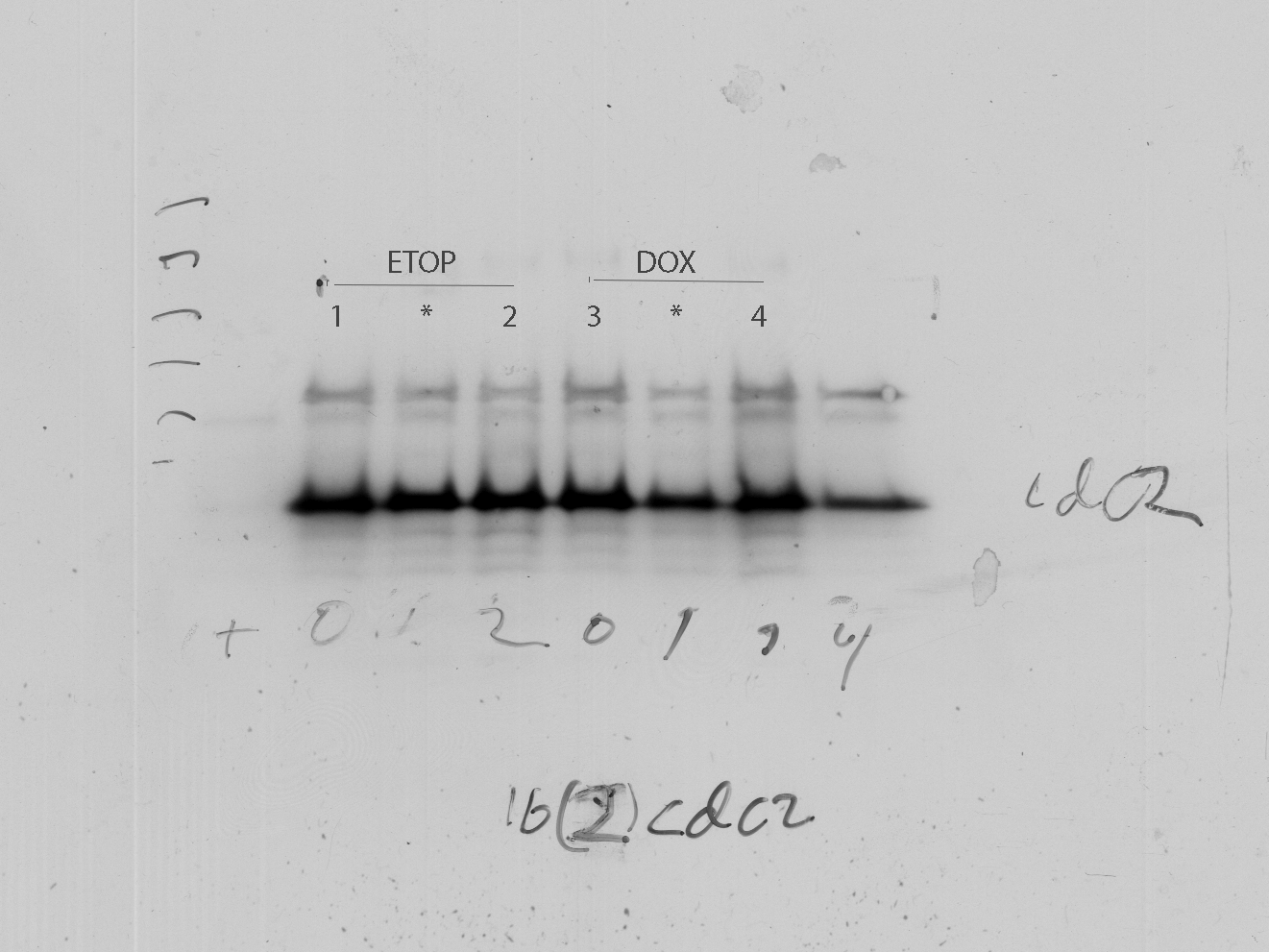

Supplement: S2 File — Note that the underlying blot for Fig 2A ETOP Chk1 IP-WB may be a different exposure than that used for the figure. (ZIP) [file pone.0292423.s002.zip › S2 File/Figure 2/Fig 2B ETOP DOX-Cdc2-F.tif]

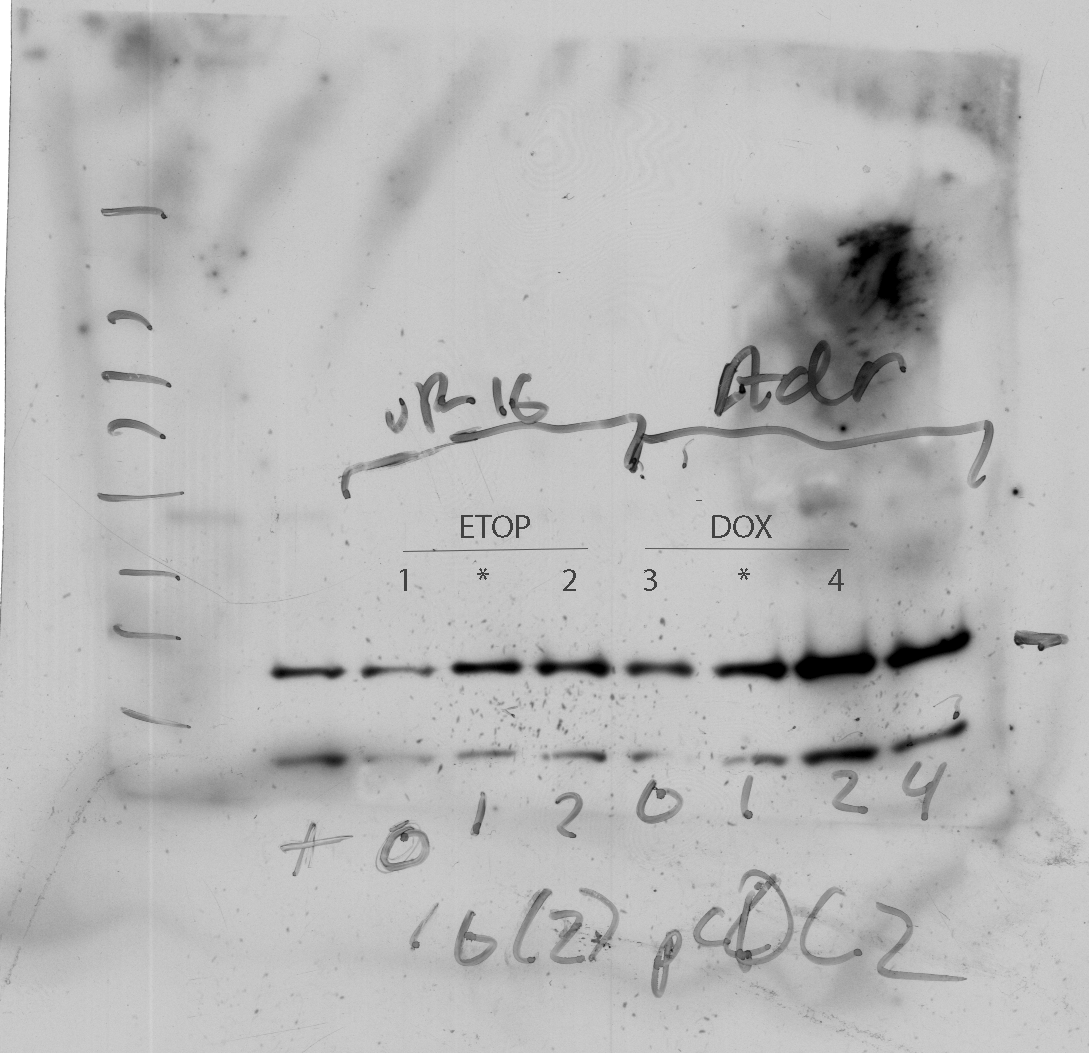

Supplement: S2 File — Note that the underlying blot for Fig 2A ETOP Chk1 IP-WB may be a different exposure than that used for the figure. (ZIP) [file pone.0292423.s002.zip › S2 File/Figure 2/Fig 2B ETOP DOX-pCDC2-F.tif]

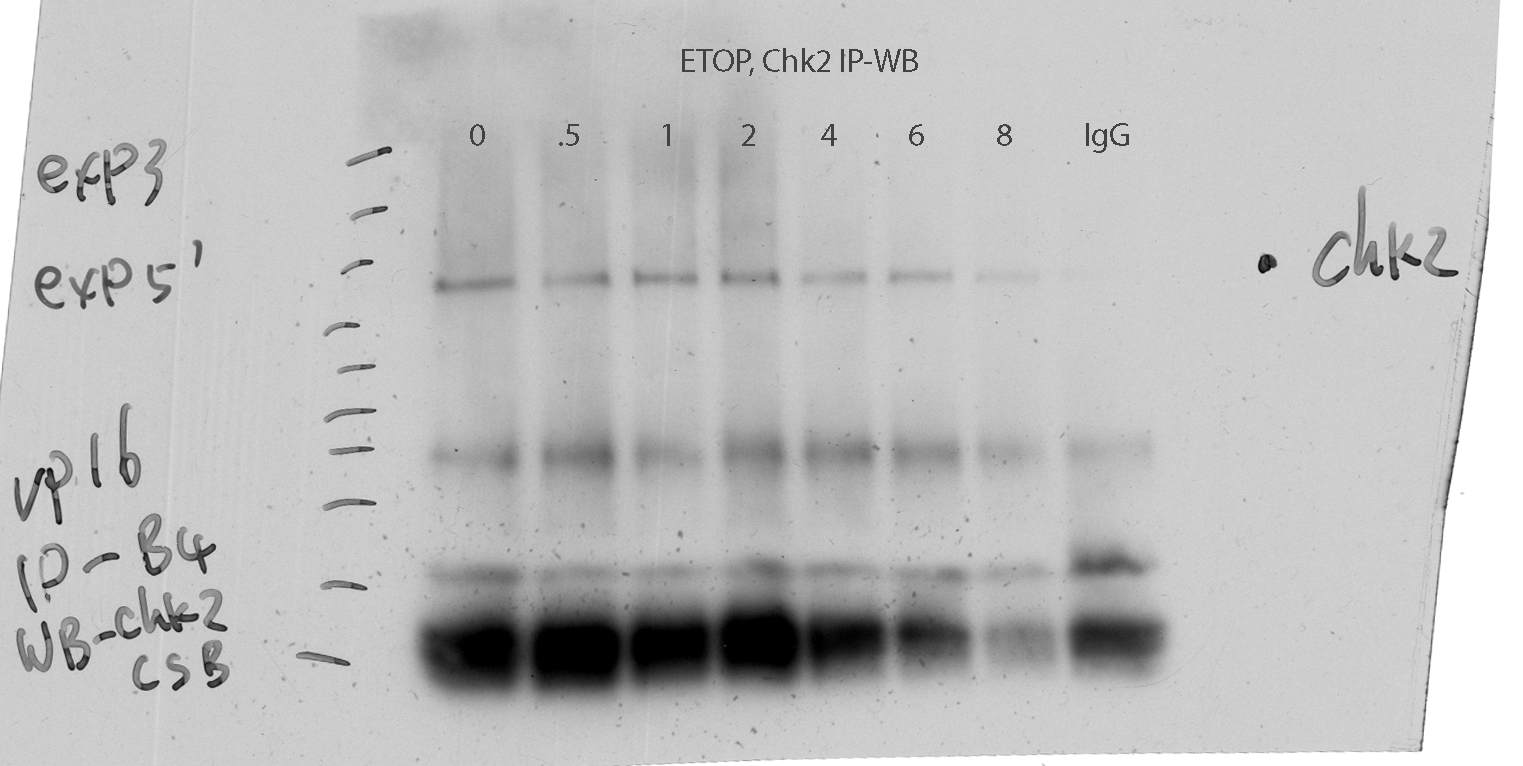

Supplement: S2 File — Note that the underlying blot for Fig 2A ETOP Chk1 IP-WB may be a different exposure than that used for the figure. (ZIP) [file pone.0292423.s002.zip › S2 File/Figure 2/Fig2A ETOP CHK2 IP-WB-F.tif]

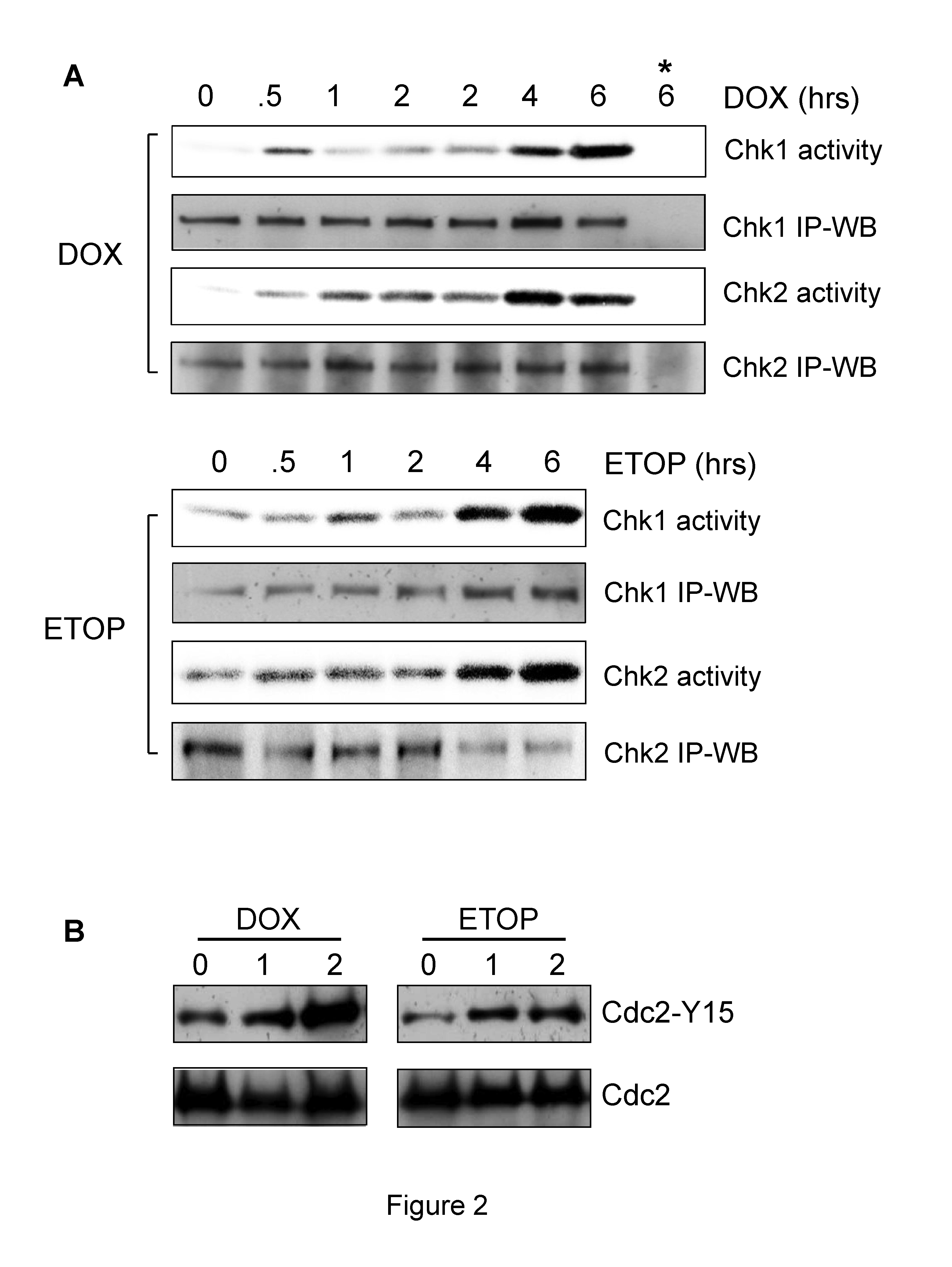

Supplement: S2 File — Note that the underlying blot for Fig 2A ETOP Chk1 IP-WB may be a different exposure than that used for the figure. (ZIP) [file pone.0292423.s002.zip › S2 File/Figure 2/Revised Figure 2.tif]

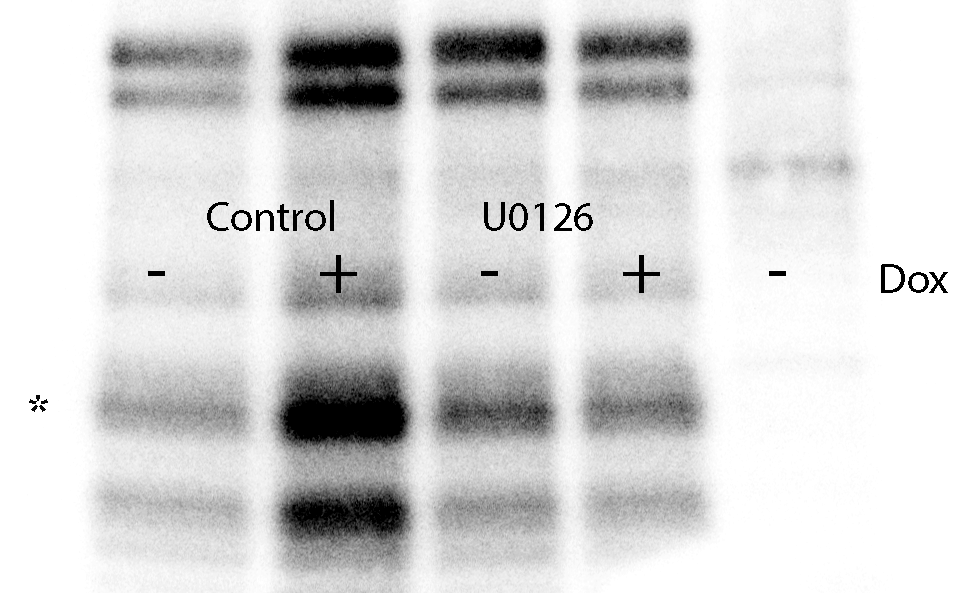

Supplement: S3 File — Note that the underlying blots for Fig 6A ETOP ATR activity, 6B ATR activity, and 6C DOX Chk2 activity are available only as cropped images. The underlying 6C ETOP ATM activity blots are from a shorter exposure than that used for the figure. (ZIP) [file pone.0292423.s003.zip › S3 File/Figure 6/6A/Fig 6A, DOX ATR activity.tif]

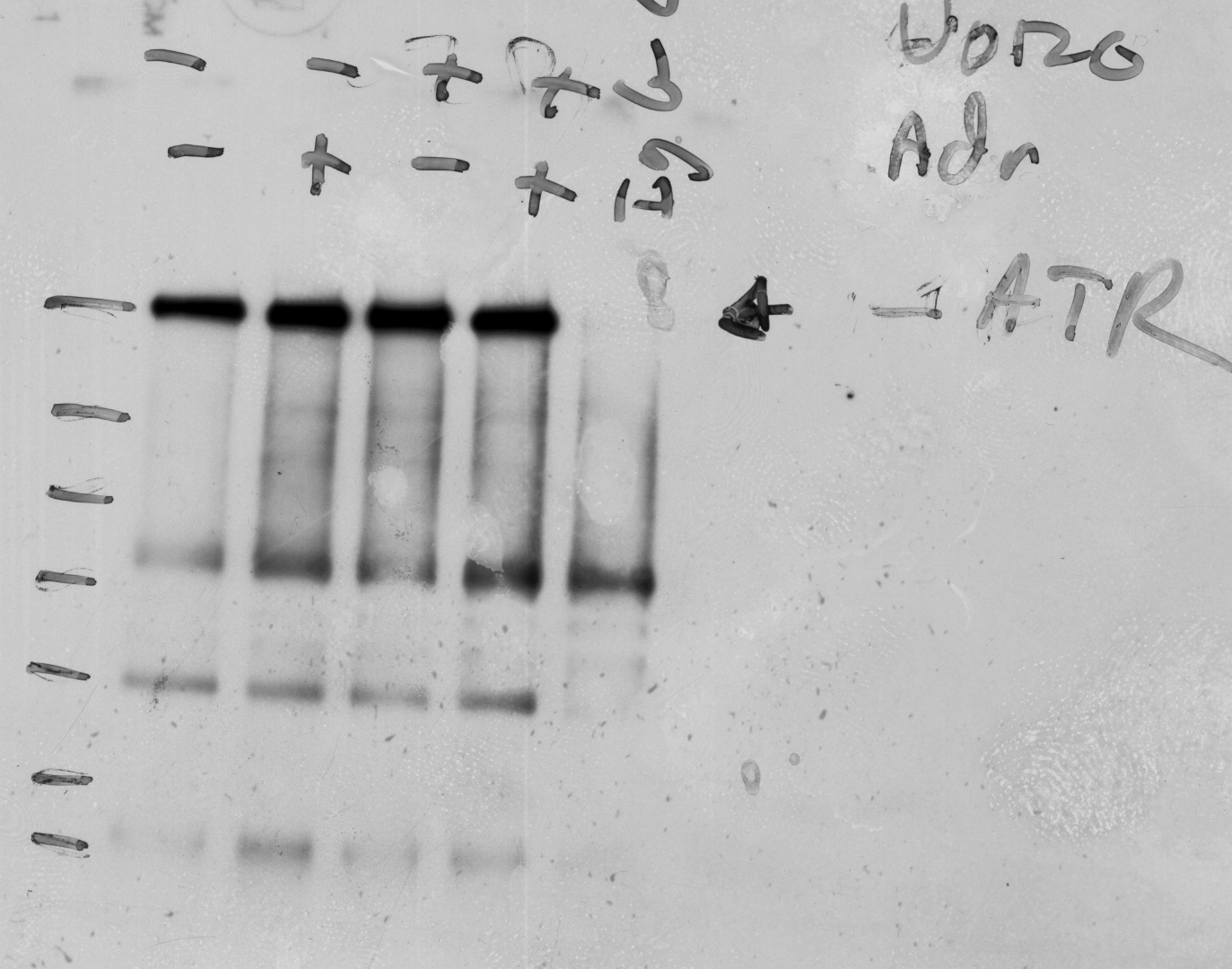

Supplement: S3 File — Note that the underlying blots for Fig 6A ETOP ATR activity, 6B ATR activity, and 6C DOX Chk2 activity are available only as cropped images. The underlying 6C ETOP ATM activity blots are from a shorter exposure than that used for the figure. (ZIP) [file pone.0292423.s003.zip › S3 File/Figure 6/6A/Fig 6A, DOX, ATR IP-WB.tif]

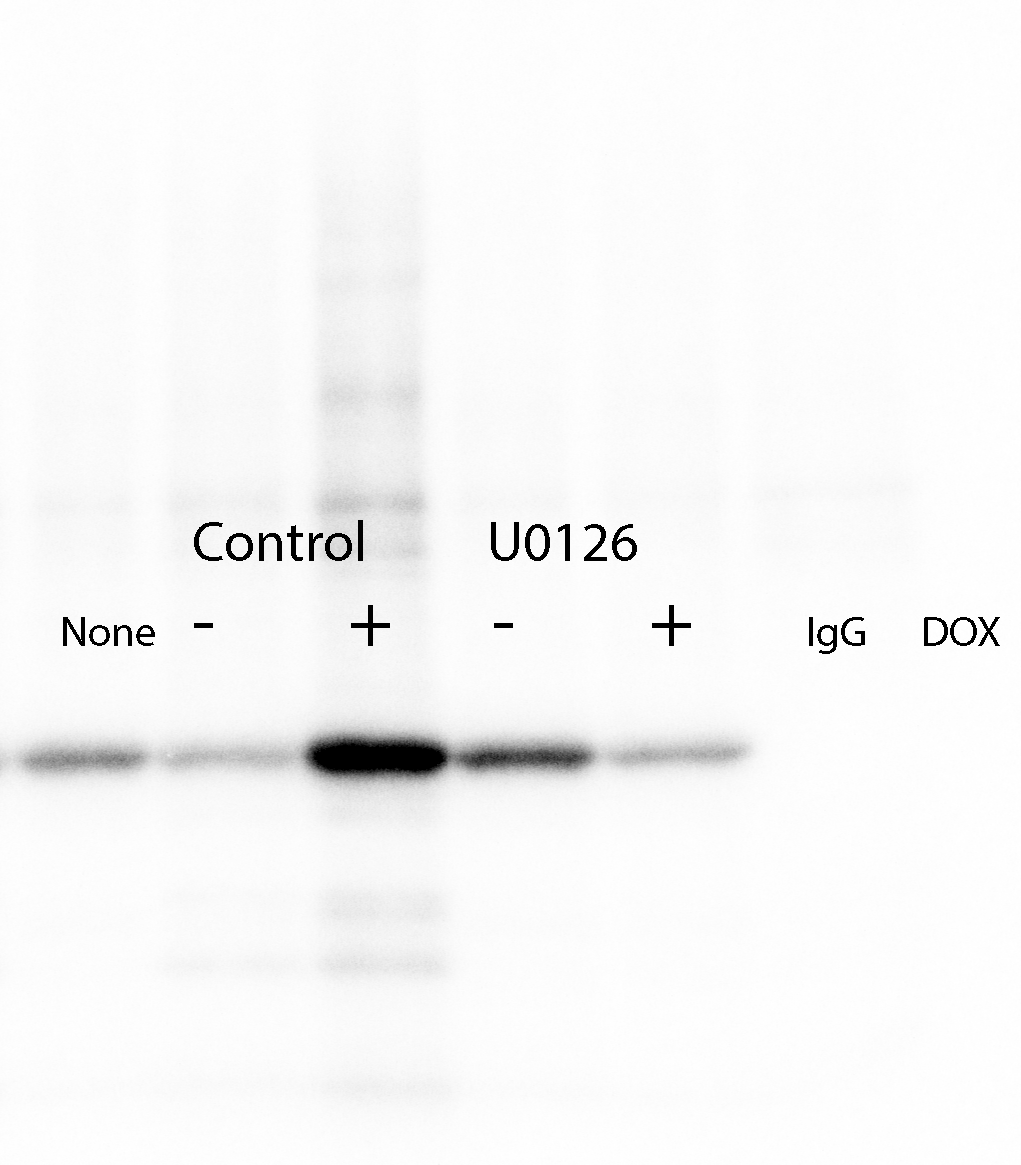

Supplement: S3 File — Note that the underlying blots for Fig 6A ETOP ATR activity, 6B ATR activity, and 6C DOX Chk2 activity are available only as cropped images. The underlying 6C ETOP ATM activity blots are from a shorter exposure than that used for the figure. (ZIP) [file pone.0292423.s003.zip › S3 File/Figure 6/6A/Fig 6A, DOX, Chk1 activity-2nd.tif]

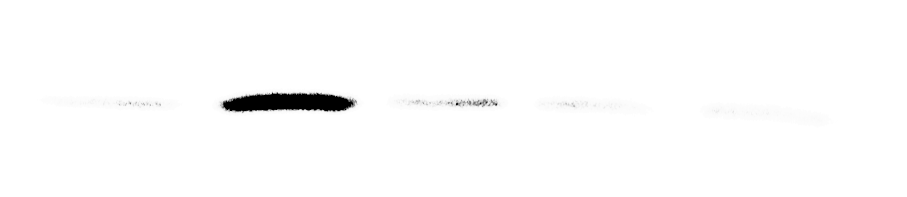

Supplement: S3 File — Note that the underlying blots for Fig 6A ETOP ATR activity, 6B ATR activity, and 6C DOX Chk2 activity are available only as cropped images. The underlying 6C ETOP ATM activity blots are from a shorter exposure than that used for the figure. (ZIP) [file pone.0292423.s003.zip › S3 File/Figure 6/6A/Fig 6A, Dox, Chk1 activity.tif]

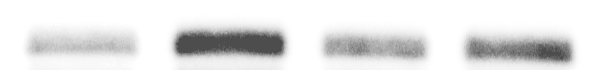

Supplement: S3 File — Note that the underlying blots for Fig 6A ETOP ATR activity, 6B ATR activity, and 6C DOX Chk2 activity are available only as cropped images. The underlying 6C ETOP ATM activity blots are from a shorter exposure than that used for the figure. (ZIP) [file pone.0292423.s003.zip › S3 File/Figure 6/6A/Fig 6A, ETOP ATR, activity.tif]

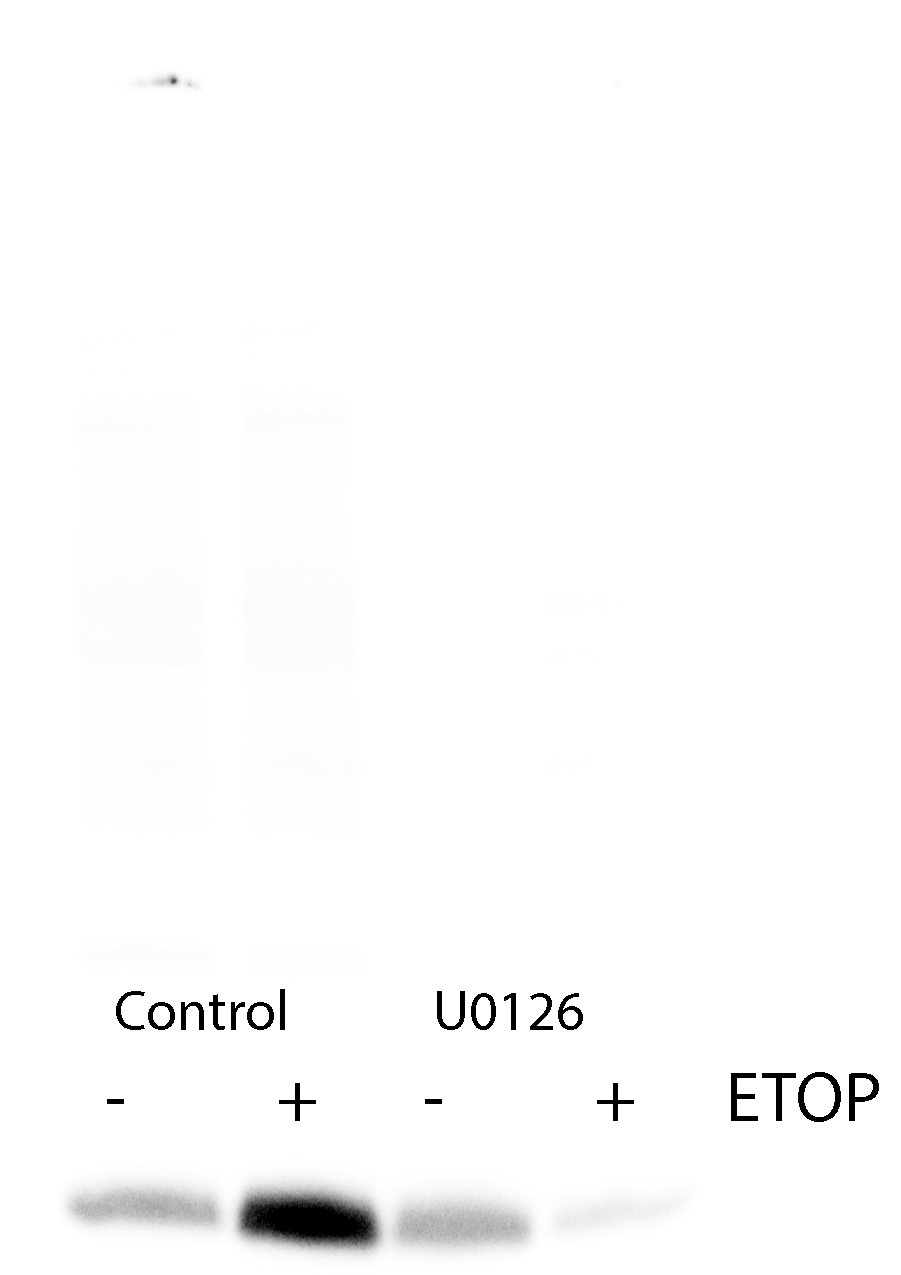

Supplement: S3 File — Note that the underlying blots for Fig 6A ETOP ATR activity, 6B ATR activity, and 6C DOX Chk2 activity are available only as cropped images. The underlying 6C ETOP ATM activity blots are from a shorter exposure than that used for the figure. (ZIP) [file pone.0292423.s003.zip › S3 File/Figure 6/6A/Fig 6A, ETOP, Chk1 activity.tif]

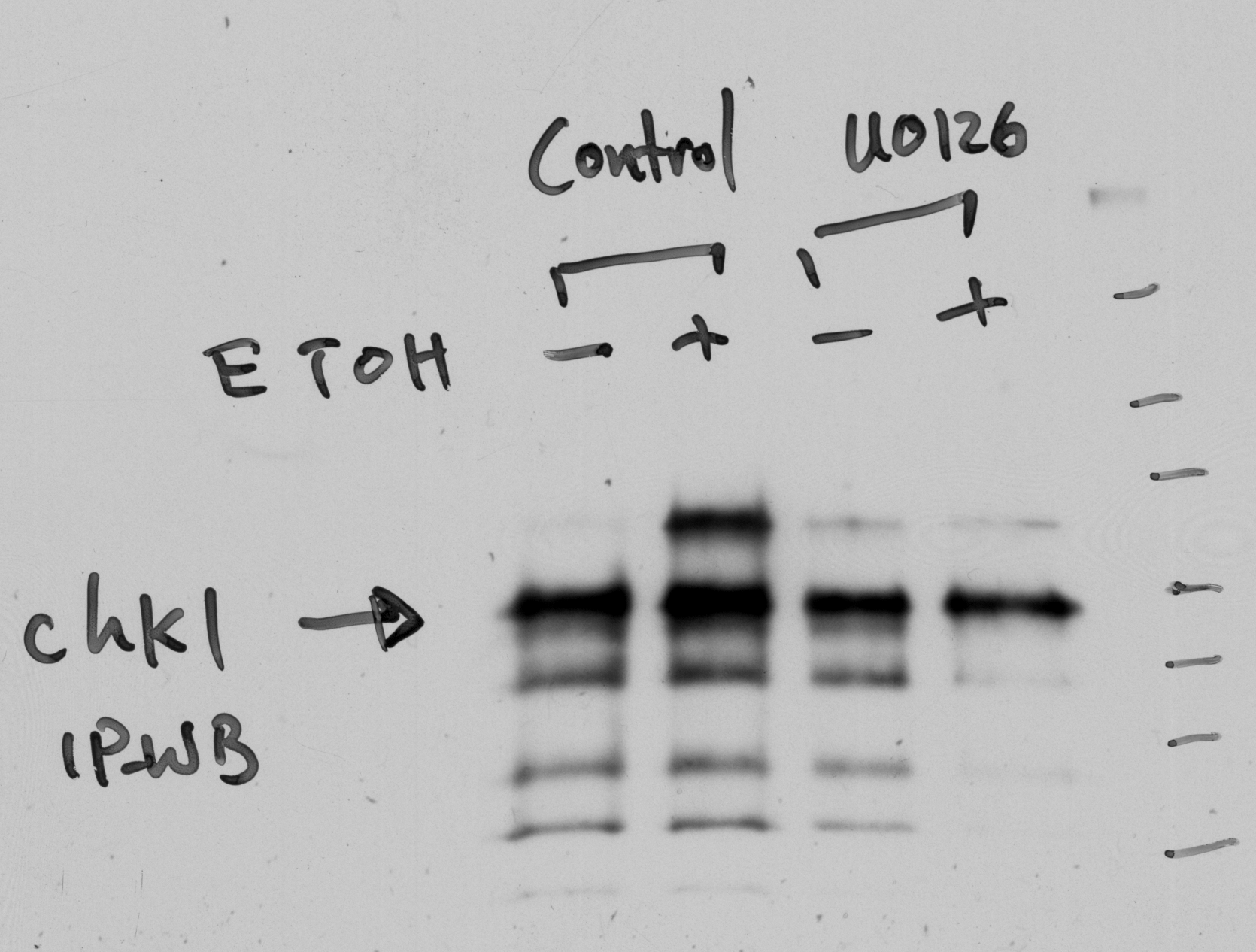

Supplement: S3 File — Note that the underlying blots for Fig 6A ETOP ATR activity, 6B ATR activity, and 6C DOX Chk2 activity are available only as cropped images. The underlying 6C ETOP ATM activity blots are from a shorter exposure than that used for the figure. (ZIP) [file pone.0292423.s003.zip › S3 File/Figure 6/6A/Fig 6A, ETOP, Chk1 IP-WB.tif]

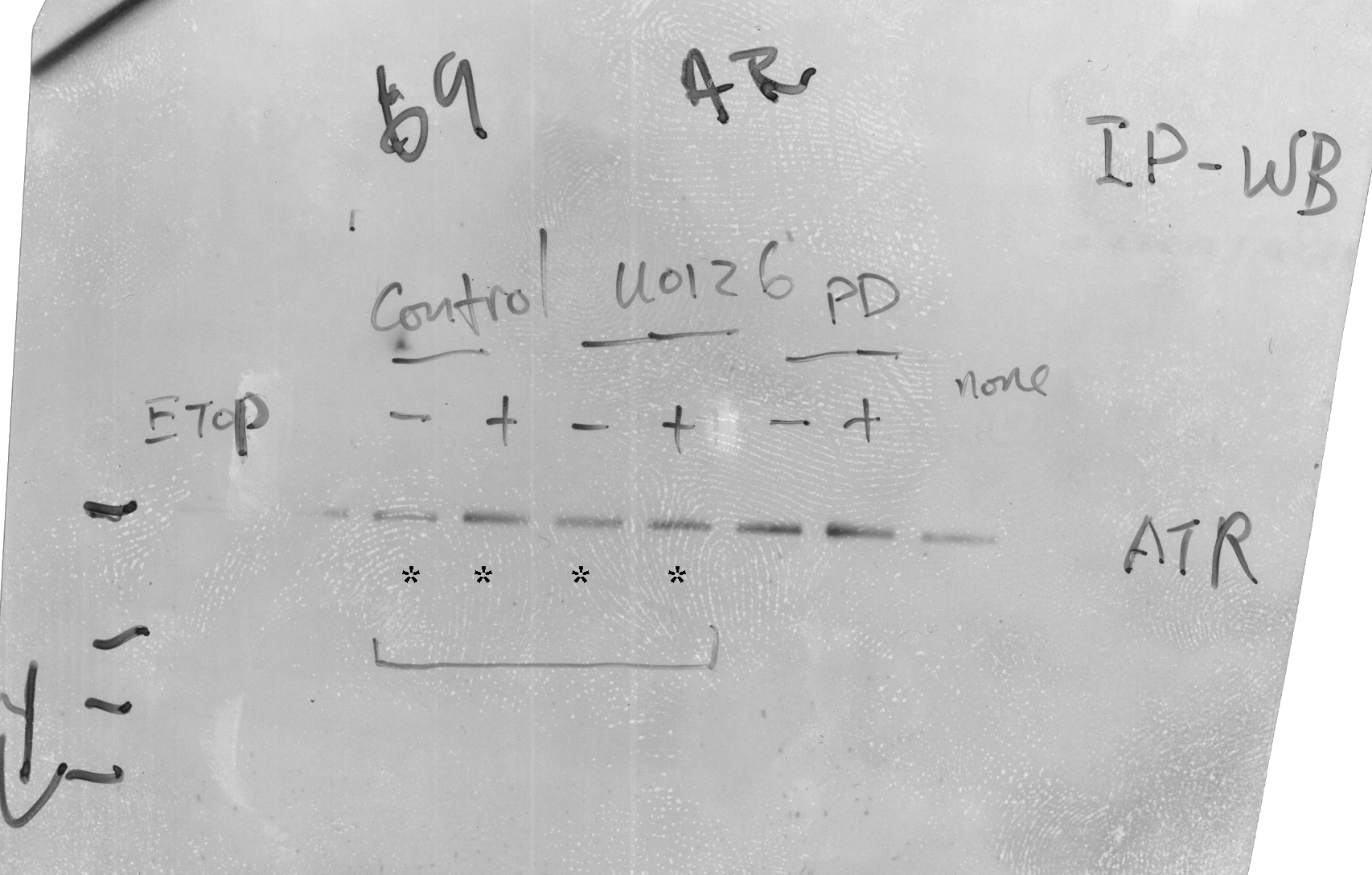

Supplement: S3 File — Note that the underlying blots for Fig 6A ETOP ATR activity, 6B ATR activity, and 6C DOX Chk2 activity are available only as cropped images. The underlying 6C ETOP ATM activity blots are from a shorter exposure than that used for the figure. (ZIP) [file pone.0292423.s003.zip › S3 File/Figure 6/6A/Fig 6A, ETOP-ATR IP-WB.tif]

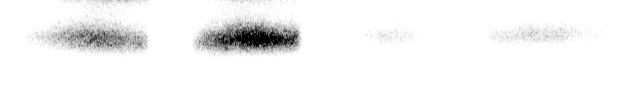

Supplement: S3 File — Note that the underlying blots for Fig 6A ETOP ATR activity, 6B ATR activity, and 6C DOX Chk2 activity are available only as cropped images. The underlying 6C ETOP ATM activity blots are from a shorter exposure than that used for the figure. (ZIP) [file pone.0292423.s003.zip › S3 File/Figure 6/6B/ATR activity-on p53 substrate.tif]

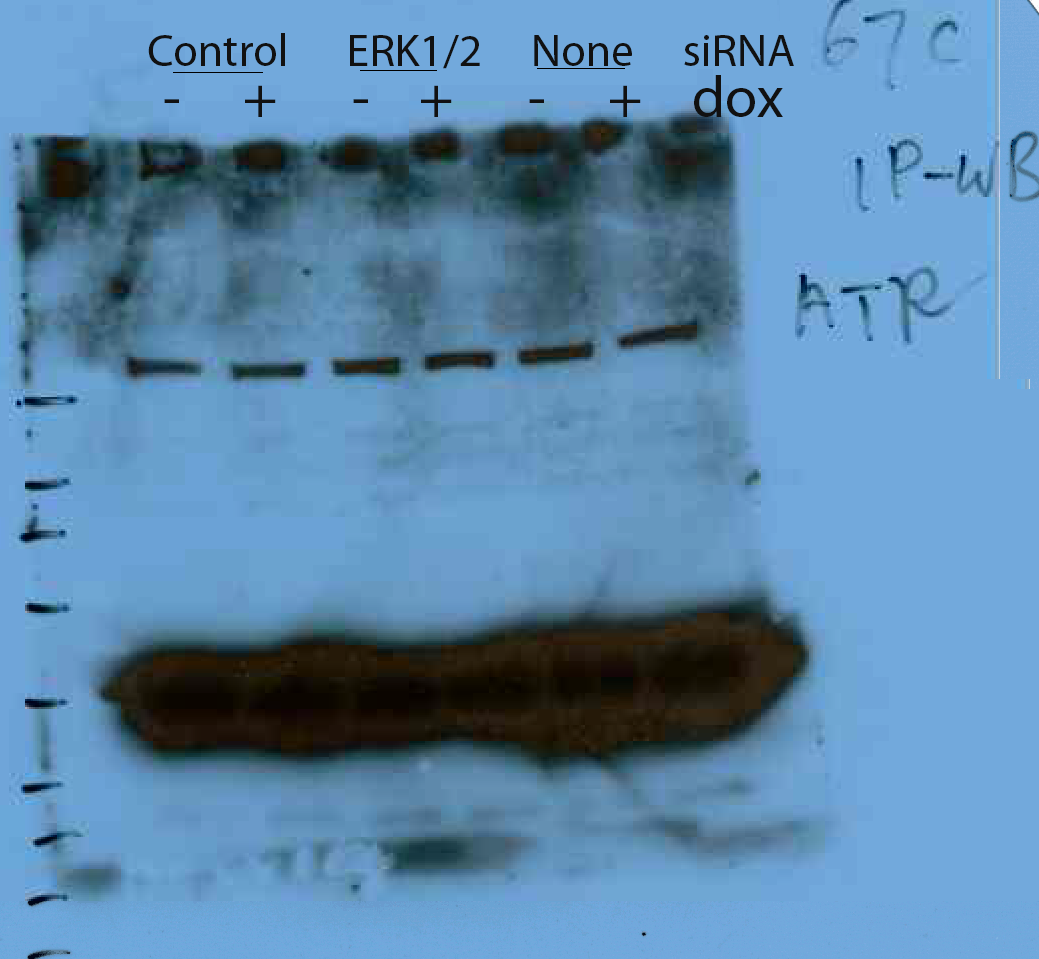

Supplement: S3 File — Note that the underlying blots for Fig 6A ETOP ATR activity, 6B ATR activity, and 6C DOX Chk2 activity are available only as cropped images. The underlying 6C ETOP ATM activity blots are from a shorter exposure than that used for the figure. (ZIP) [file pone.0292423.s003.zip › S3 File/Figure 6/6B/ATR-IP-WB of Fig 6B.tif]

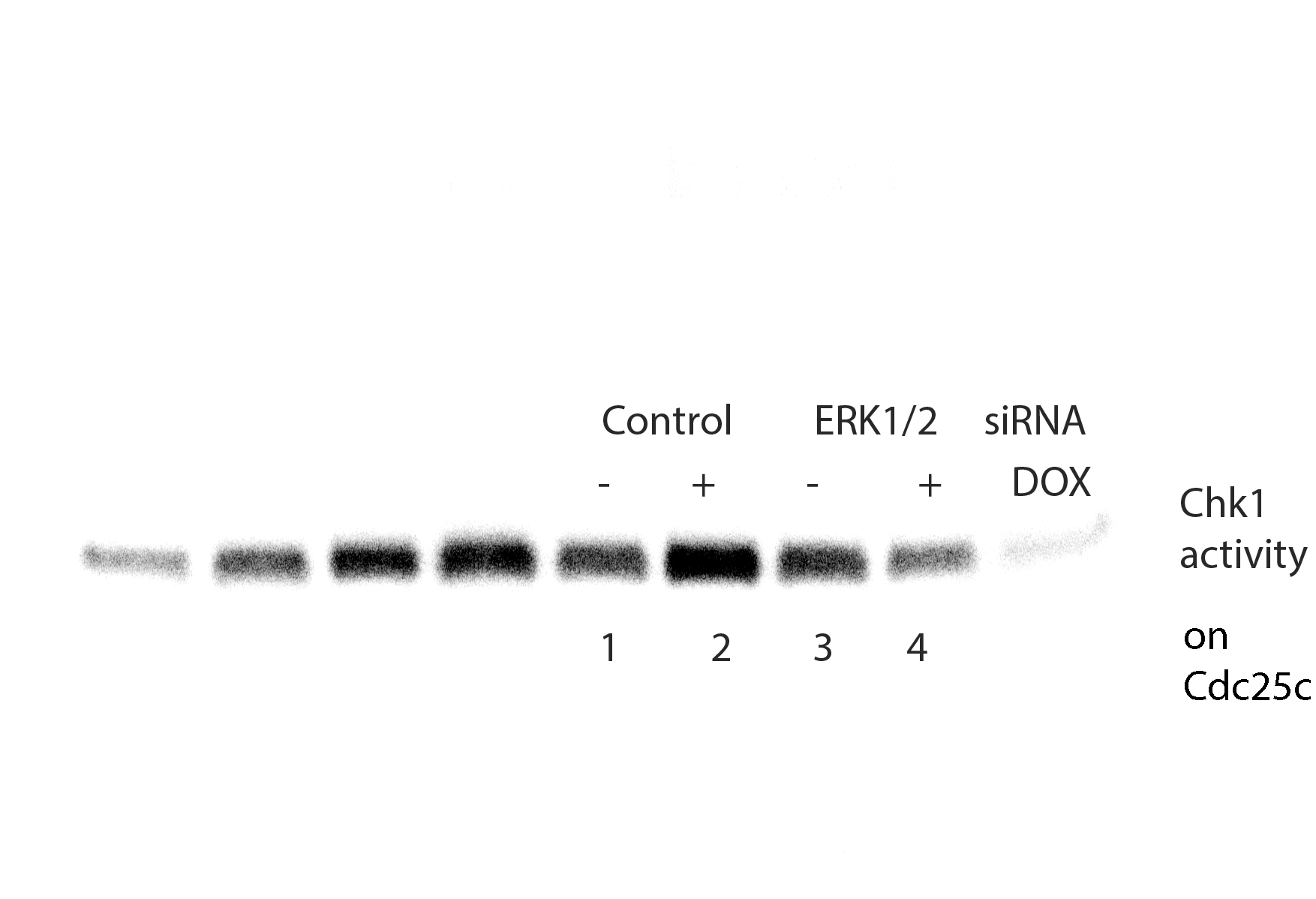

Supplement: S3 File — Note that the underlying blots for Fig 6A ETOP ATR activity, 6B ATR activity, and 6C DOX Chk2 activity are available only as cropped images. The underlying 6C ETOP ATM activity blots are from a shorter exposure than that used for the figure. (ZIP) [file pone.0292423.s003.zip › S3 File/Figure 6/6B/Chk1 activity-on Cdc25C substrate.tif]

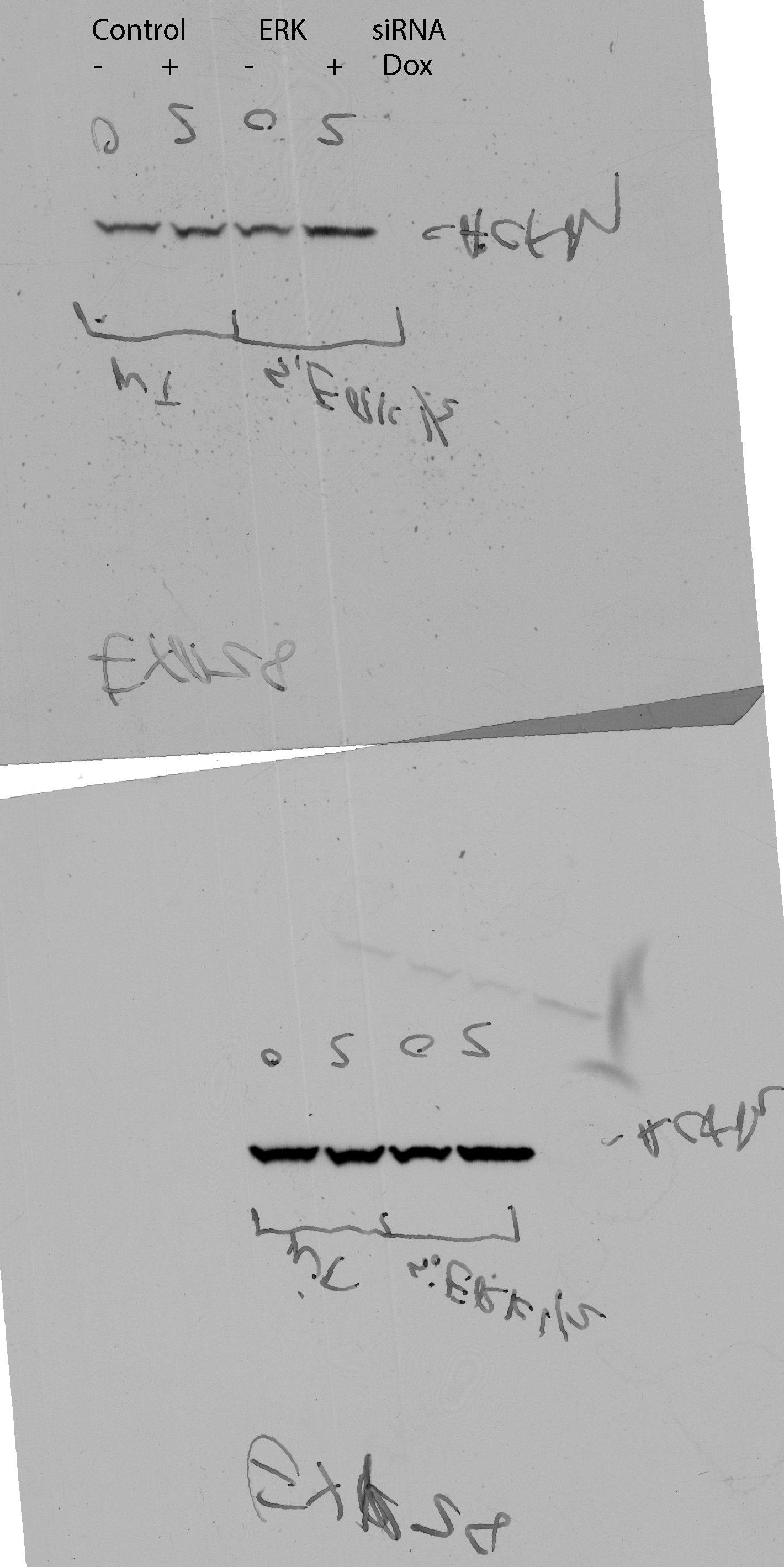

Supplement: S3 File — Note that the underlying blots for Fig 6A ETOP ATR activity, 6B ATR activity, and 6C DOX Chk2 activity are available only as cropped images. The underlying 6C ETOP ATM activity blots are from a shorter exposure than that used for the figure. (ZIP) [file pone.0292423.s003.zip › S3 File/Figure 6/6B/Fig6B- Actin.tif]

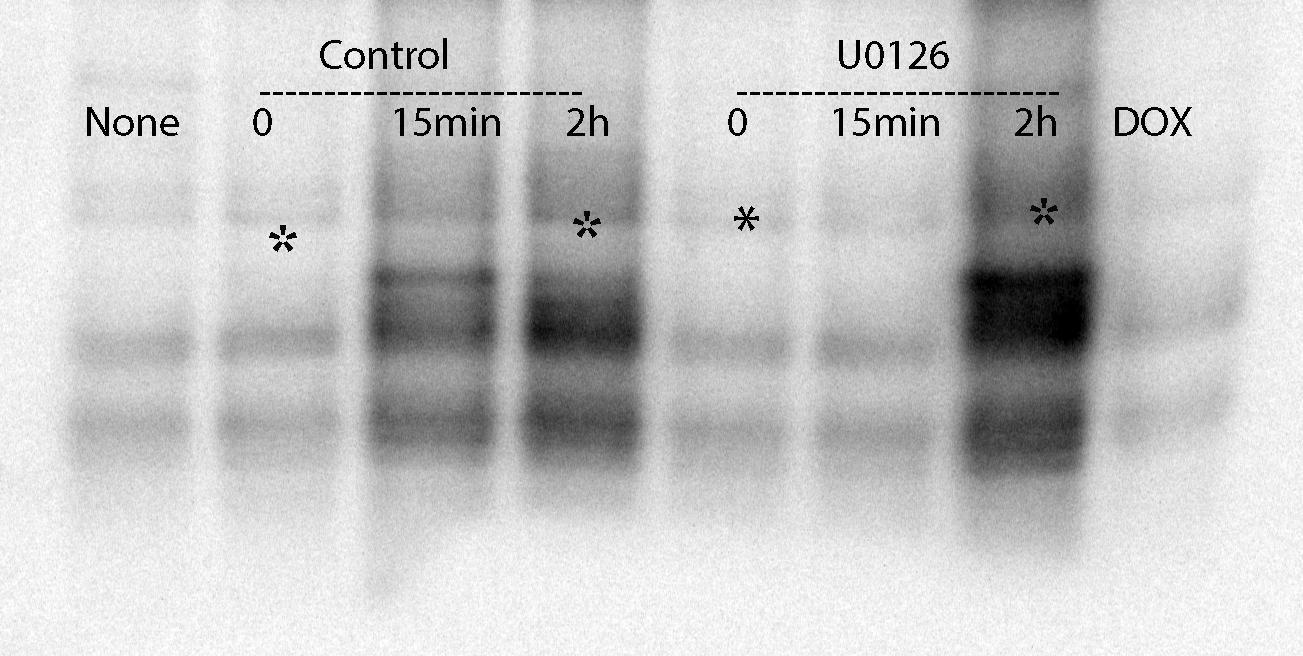

Supplement: S3 File — Note that the underlying blots for Fig 6A ETOP ATR activity, 6B ATR activity, and 6C DOX Chk2 activity are available only as cropped images. The underlying 6C ETOP ATM activity blots are from a shorter exposure than that used for the figure. (ZIP) [file pone.0292423.s003.zip › S3 File/Figure 6/6C/Fig 6C, DOX, ATM activity.tif]

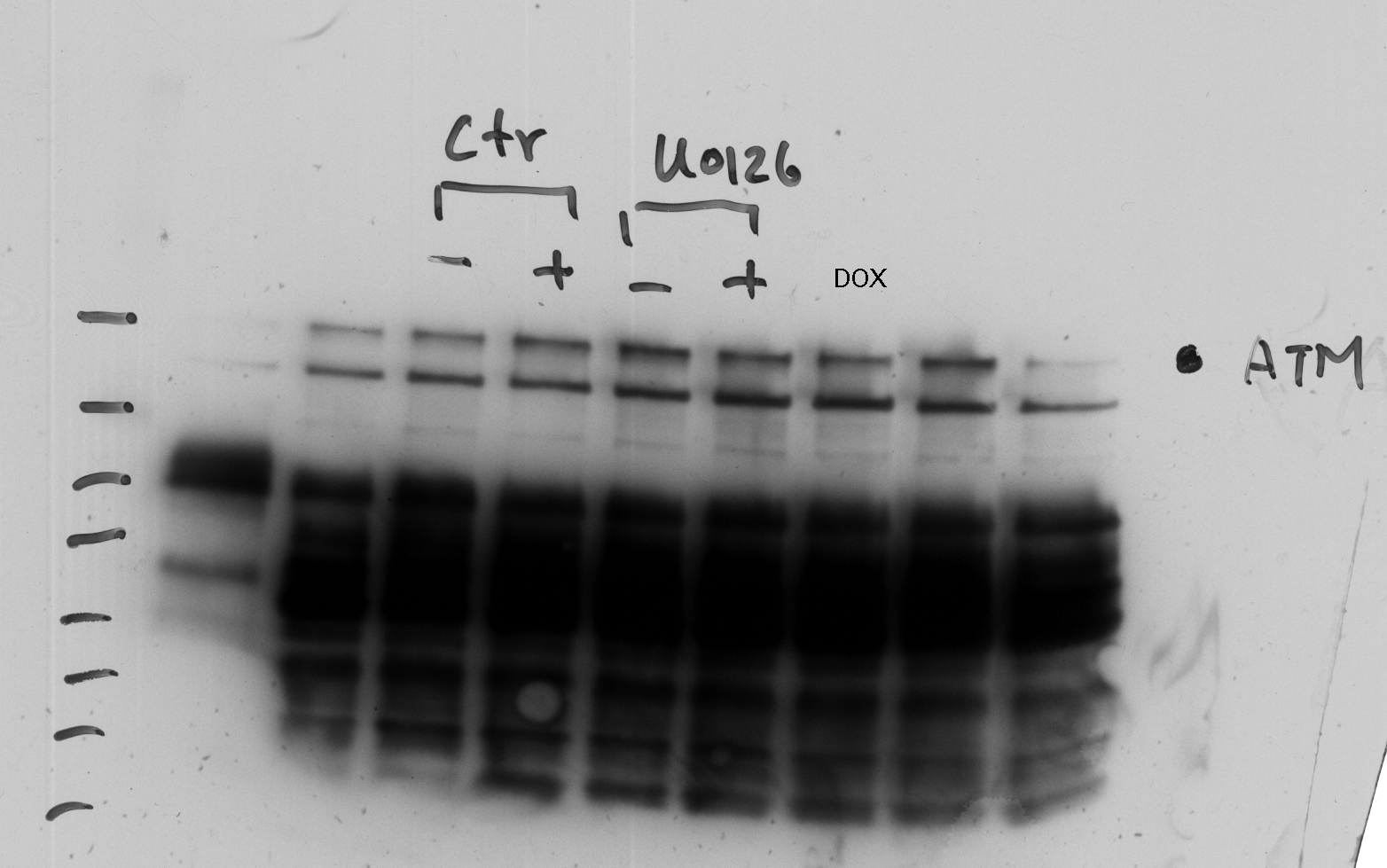

Supplement: S3 File — Note that the underlying blots for Fig 6A ETOP ATR activity, 6B ATR activity, and 6C DOX Chk2 activity are available only as cropped images. The underlying 6C ETOP ATM activity blots are from a shorter exposure than that used for the figure. (ZIP) [file pone.0292423.s003.zip › S3 File/Figure 6/6C/Fig 6C, DOX, ATM IP-WB.tif]

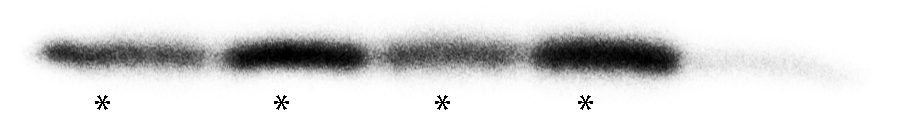

Supplement: S3 File — Note that the underlying blots for Fig 6A ETOP ATR activity, 6B ATR activity, and 6C DOX Chk2 activity are available only as cropped images. The underlying 6C ETOP ATM activity blots are from a shorter exposure than that used for the figure. (ZIP) [file pone.0292423.s003.zip › S3 File/Figure 6/6C/Fig 6C, DOX, Chk2 activity.tif]

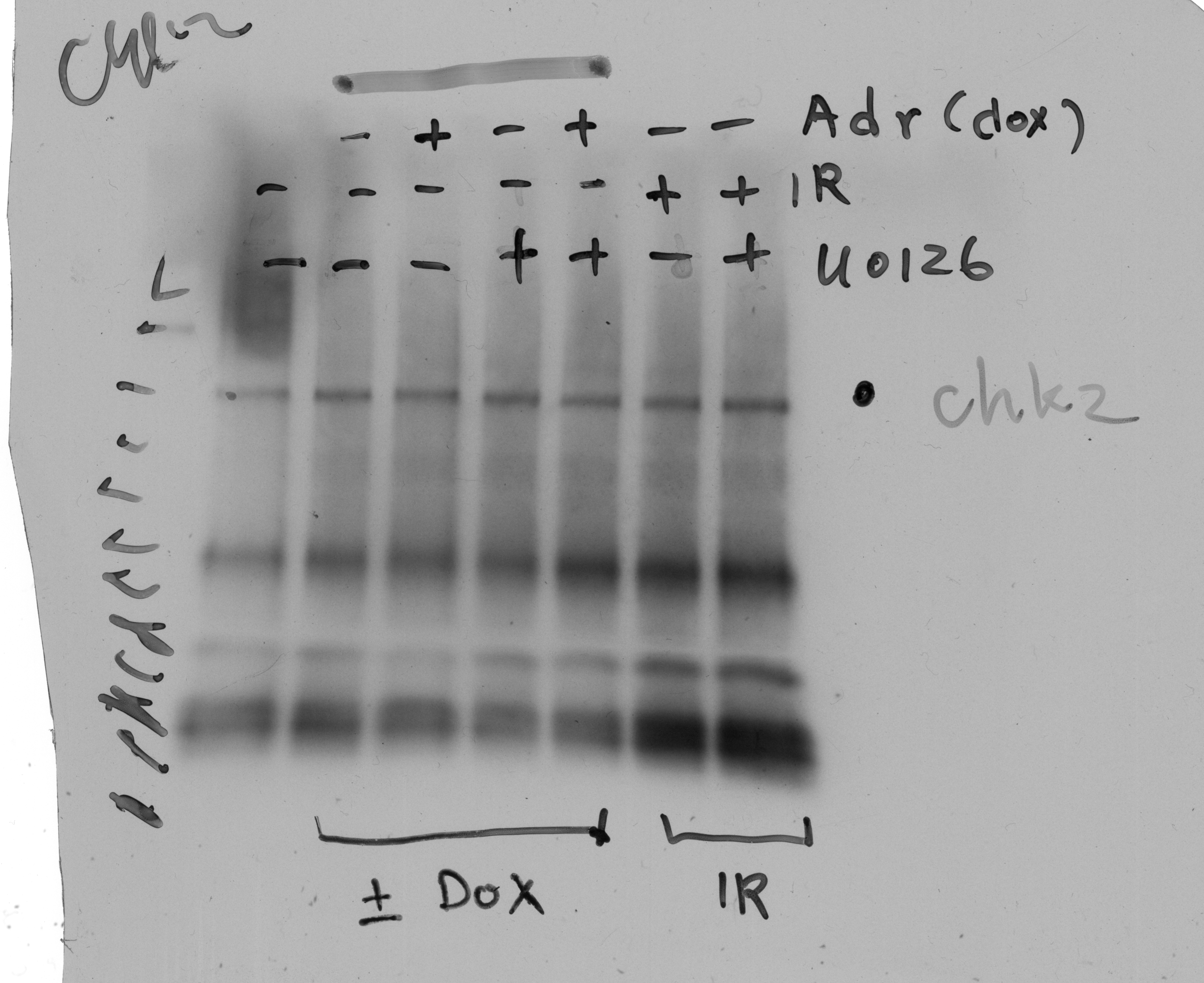

Supplement: S3 File — Note that the underlying blots for Fig 6A ETOP ATR activity, 6B ATR activity, and 6C DOX Chk2 activity are available only as cropped images. The underlying 6C ETOP ATM activity blots are from a shorter exposure than that used for the figure. (ZIP) [file pone.0292423.s003.zip › S3 File/Figure 6/6C/Fig 6C, DOX, Chk2 IP-WB-Ori.tif]

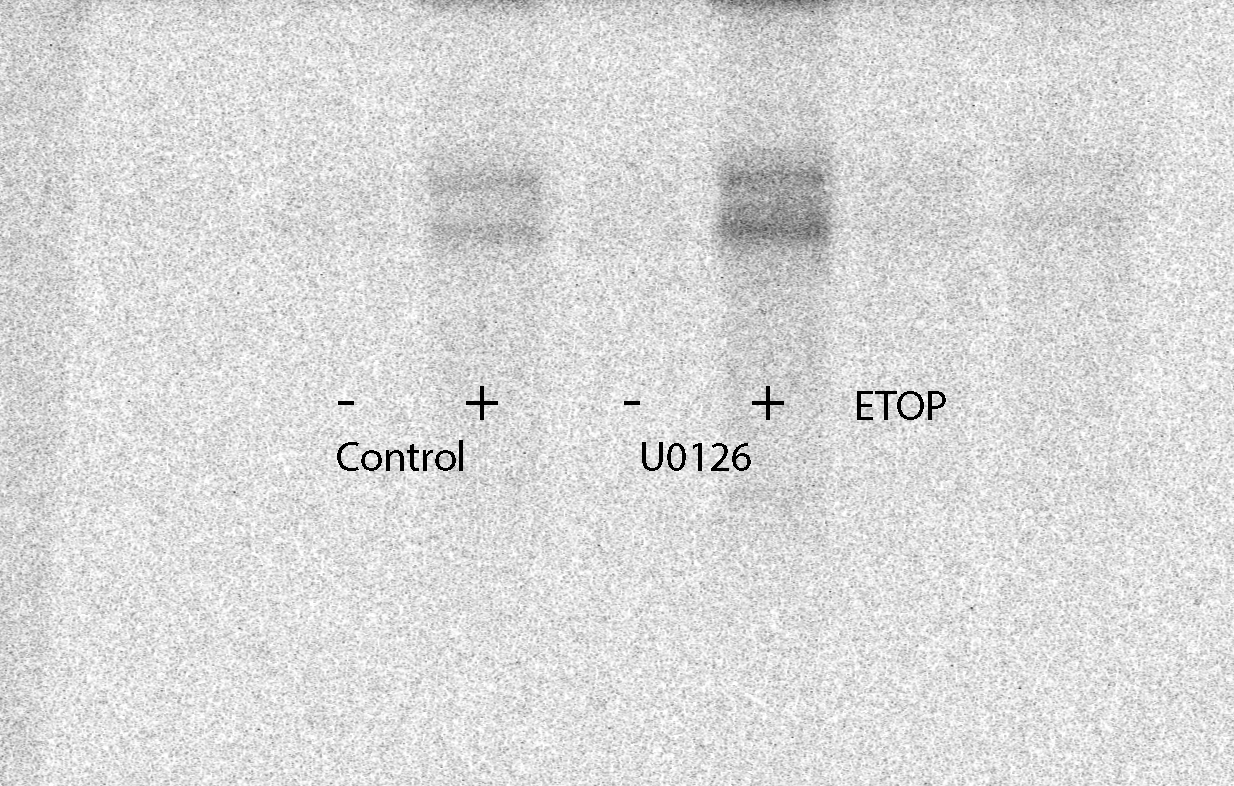

Supplement: S3 File — Note that the underlying blots for Fig 6A ETOP ATR activity, 6B ATR activity, and 6C DOX Chk2 activity are available only as cropped images. The underlying 6C ETOP ATM activity blots are from a shorter exposure than that used for the figure. (ZIP) [file pone.0292423.s003.zip › S3 File/Figure 6/6C/Fig 6C, ETOP, ATM activity-light expose1 (1).tif]

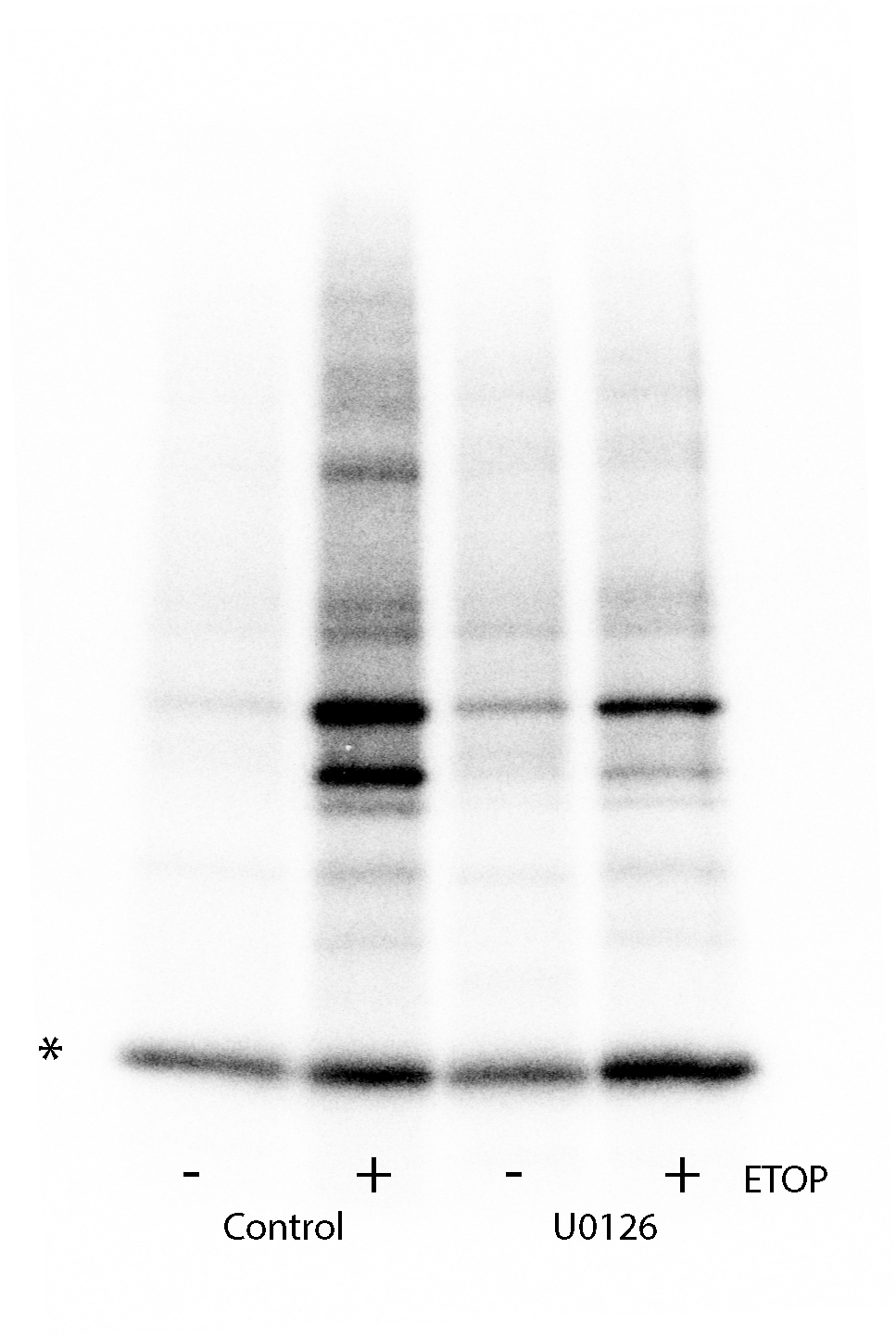

Supplement: S3 File — Note that the underlying blots for Fig 6A ETOP ATR activity, 6B ATR activity, and 6C DOX Chk2 activity are available only as cropped images. The underlying 6C ETOP ATM activity blots are from a shorter exposure than that used for the figure. (ZIP) [file pone.0292423.s003.zip › S3 File/Figure 6/6C/Fig 6C, ETOP, Chk2 activity.tif]

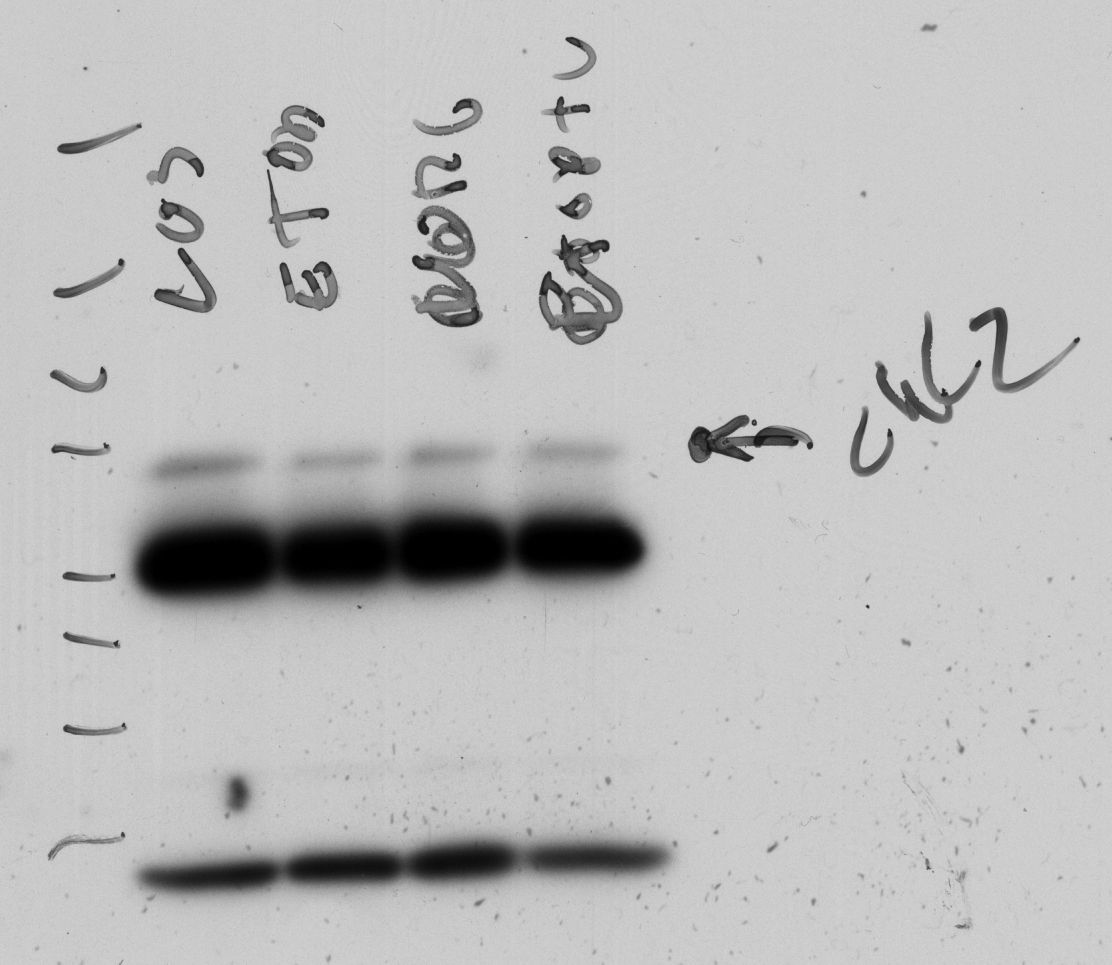

Supplement: S3 File — Note that the underlying blots for Fig 6A ETOP ATR activity, 6B ATR activity, and 6C DOX Chk2 activity are available only as cropped images. The underlying 6C ETOP ATM activity blots are from a shorter exposure than that used for the figure. (ZIP) [file pone.0292423.s003.zip › S3 File/Figure 6/6C/Fig 6C, ETOP, Chk2 IP-WB.tif]

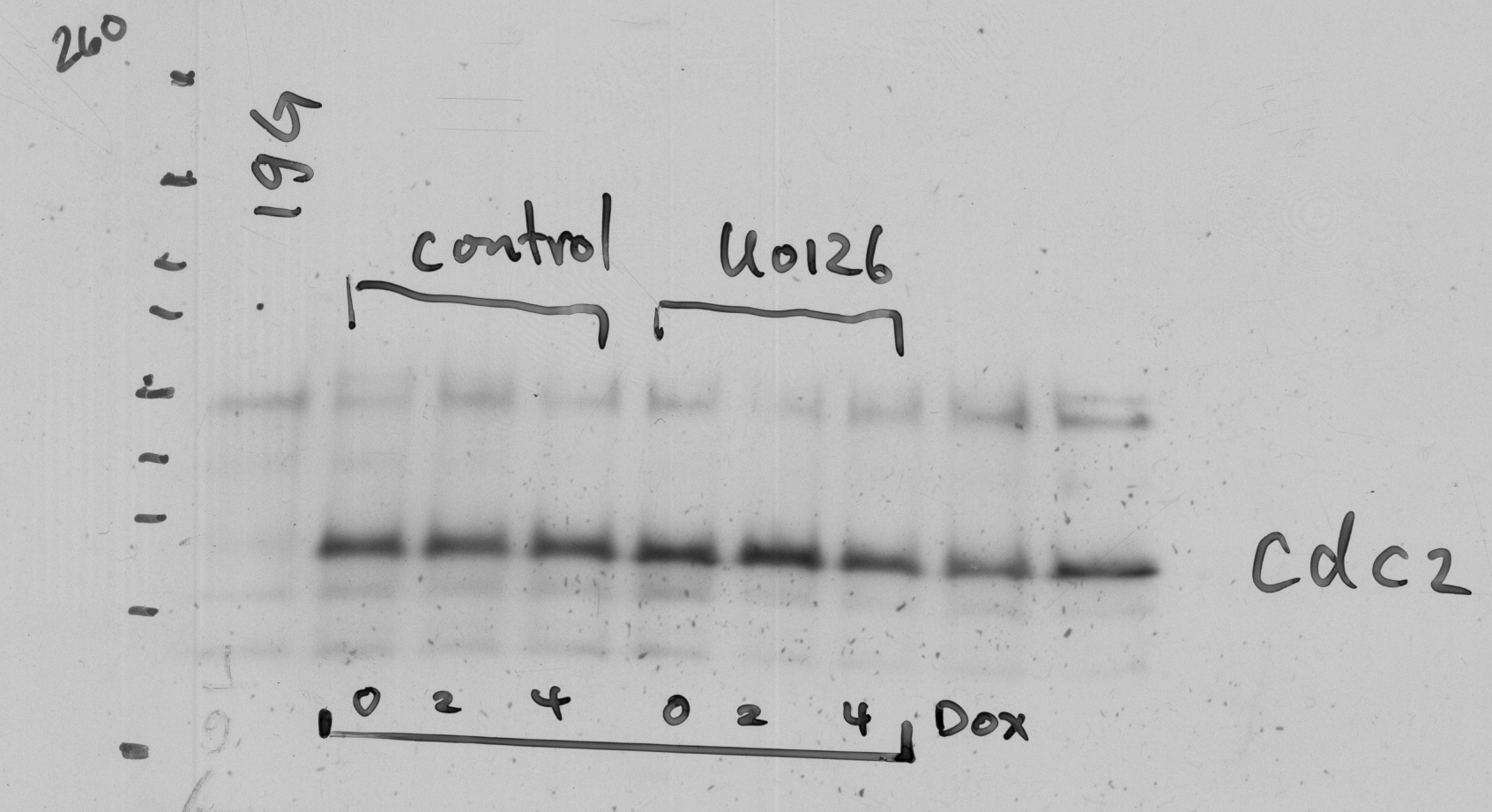

Supplement: S3 File — Note that the underlying blots for Fig 6A ETOP ATR activity, 6B ATR activity, and 6C DOX Chk2 activity are available only as cropped images. The underlying 6C ETOP ATM activity blots are from a shorter exposure than that used for the figure. (ZIP) [file pone.0292423.s003.zip › S3 File/Figure 6/6D/Fig 6D, DOX, Cdc2, IP-WB.tif]

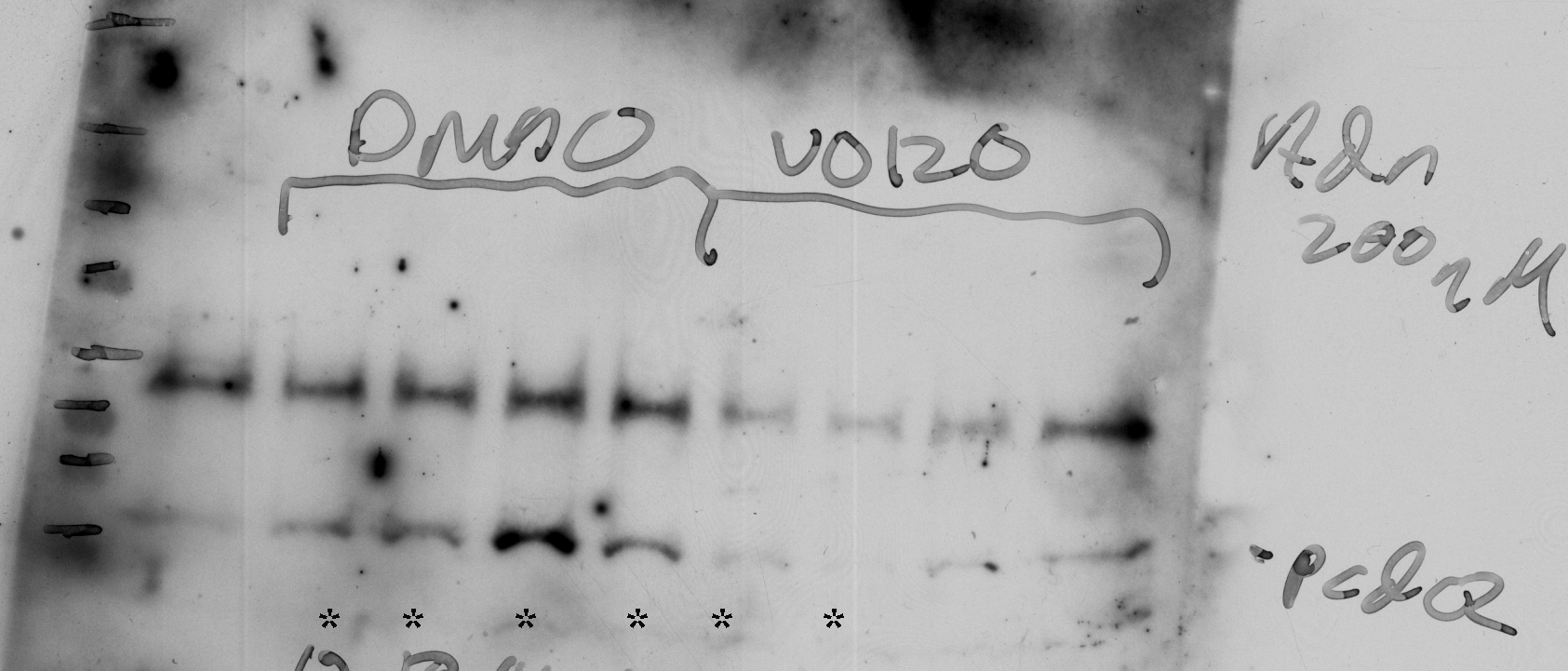

Supplement: S3 File — Note that the underlying blots for Fig 6A ETOP ATR activity, 6B ATR activity, and 6C DOX Chk2 activity are available only as cropped images. The underlying 6C ETOP ATM activity blots are from a shorter exposure than that used for the figure. (ZIP) [file pone.0292423.s003.zip › S3 File/Figure 6/6D/Fig 6D, DOX, Cdc2-Y15.tif]

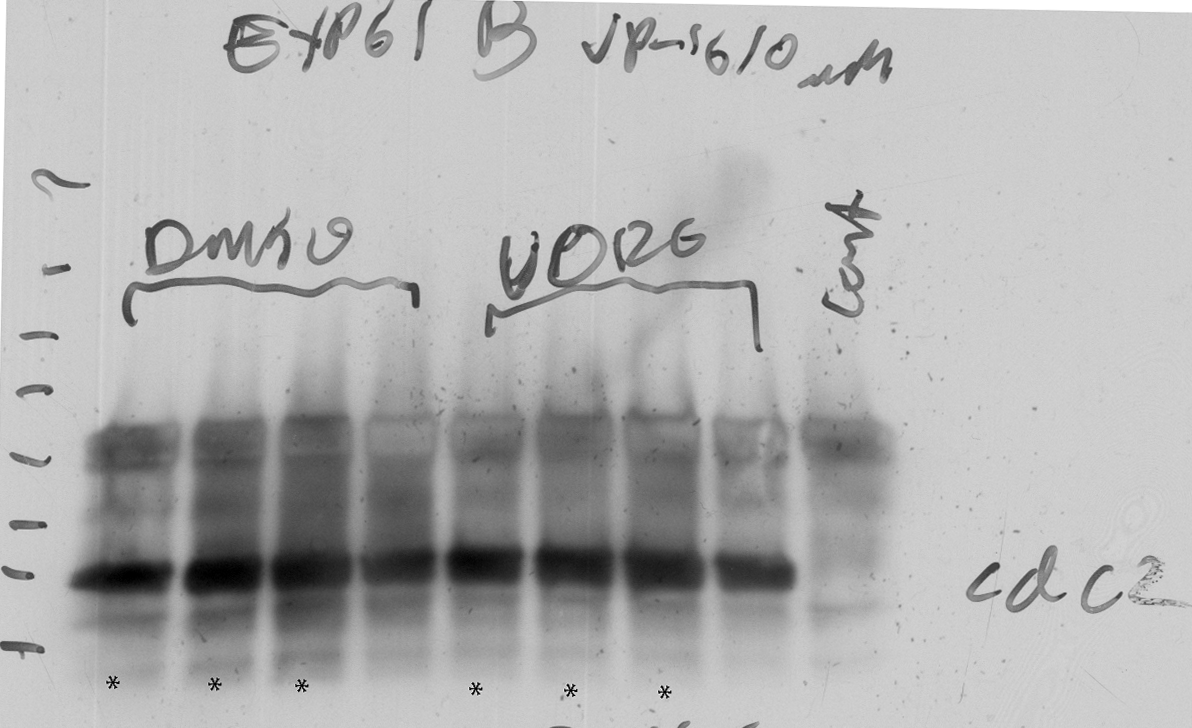

Supplement: S3 File — Note that the underlying blots for Fig 6A ETOP ATR activity, 6B ATR activity, and 6C DOX Chk2 activity are available only as cropped images. The underlying 6C ETOP ATM activity blots are from a shorter exposure than that used for the figure. (ZIP) [file pone.0292423.s003.zip › S3 File/Figure 6/6D/Fig 6D, ETOP, Cdc2 IP-WB RK.tif]

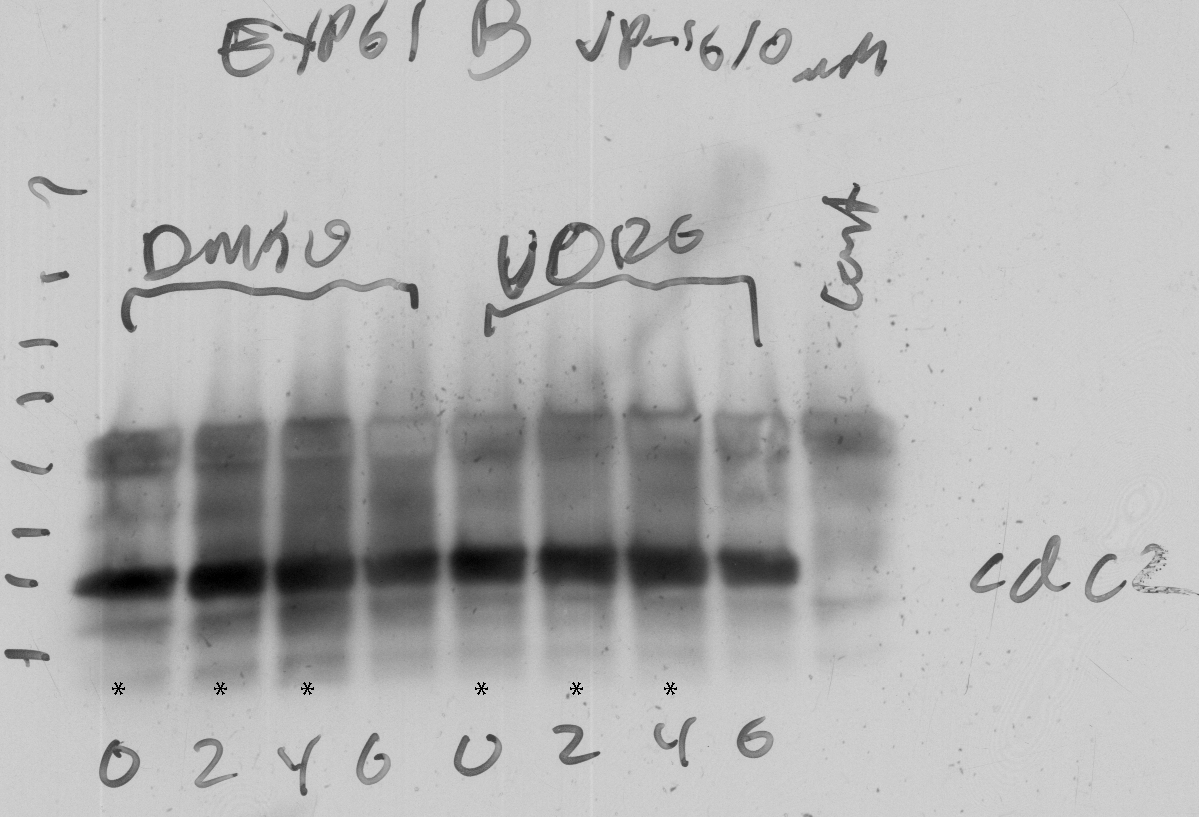

Supplement: S3 File — Note that the underlying blots for Fig 6A ETOP ATR activity, 6B ATR activity, and 6C DOX Chk2 activity are available only as cropped images. The underlying 6C ETOP ATM activity blots are from a shorter exposure than that used for the figure. (ZIP) [file pone.0292423.s003.zip › S3 File/Figure 6/6D/Fig 6D, ETOP, Cdc2 IP-WB.tif]

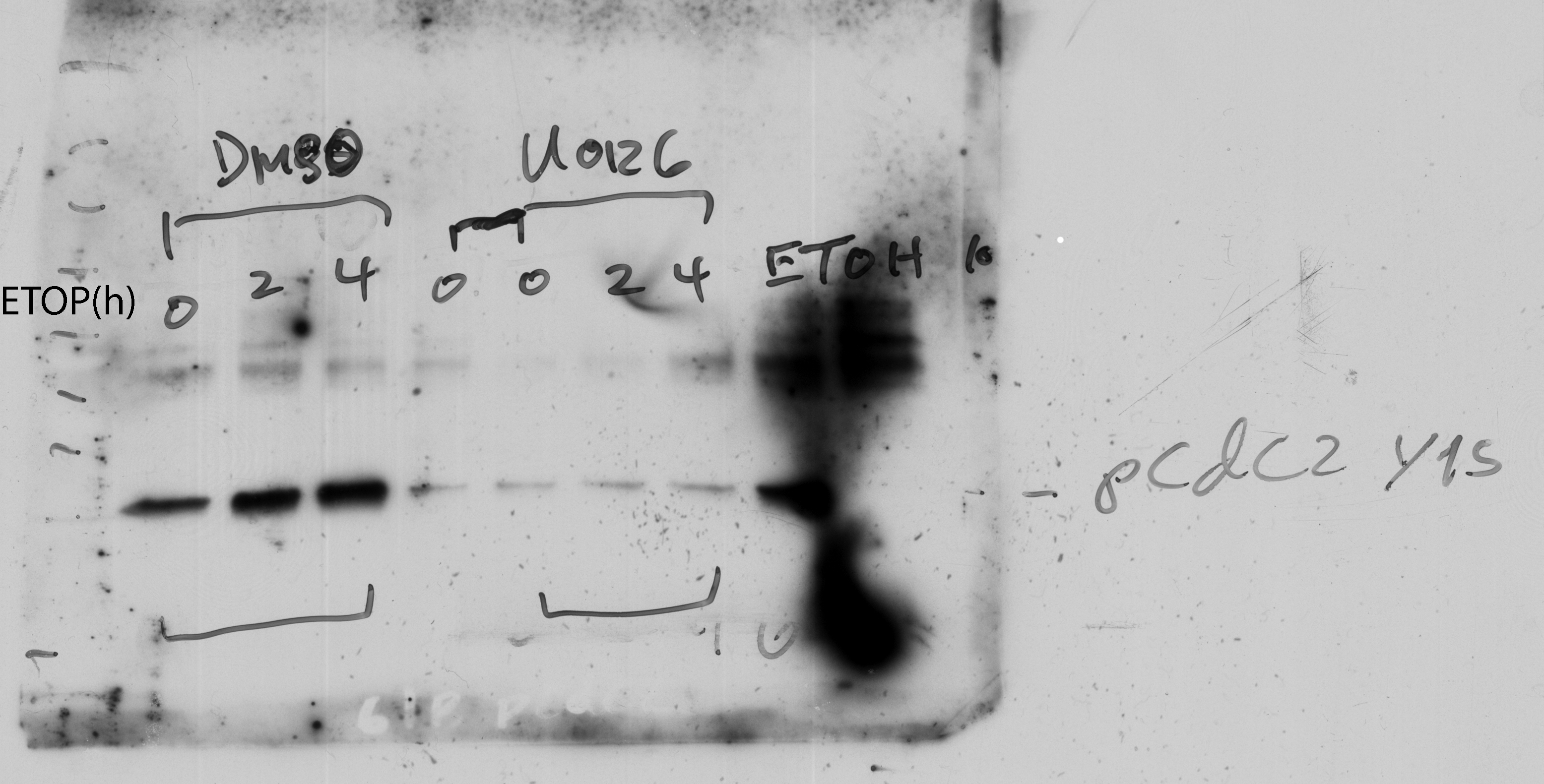

Supplement: S3 File — Note that the underlying blots for Fig 6A ETOP ATR activity, 6B ATR activity, and 6C DOX Chk2 activity are available only as cropped images. The underlying 6C ETOP ATM activity blots are from a shorter exposure than that used for the figure. (ZIP) [file pone.0292423.s003.zip › S3 File/Figure 6/6D/Fig 6D, ETOP, Cdc2-Y15.tif]

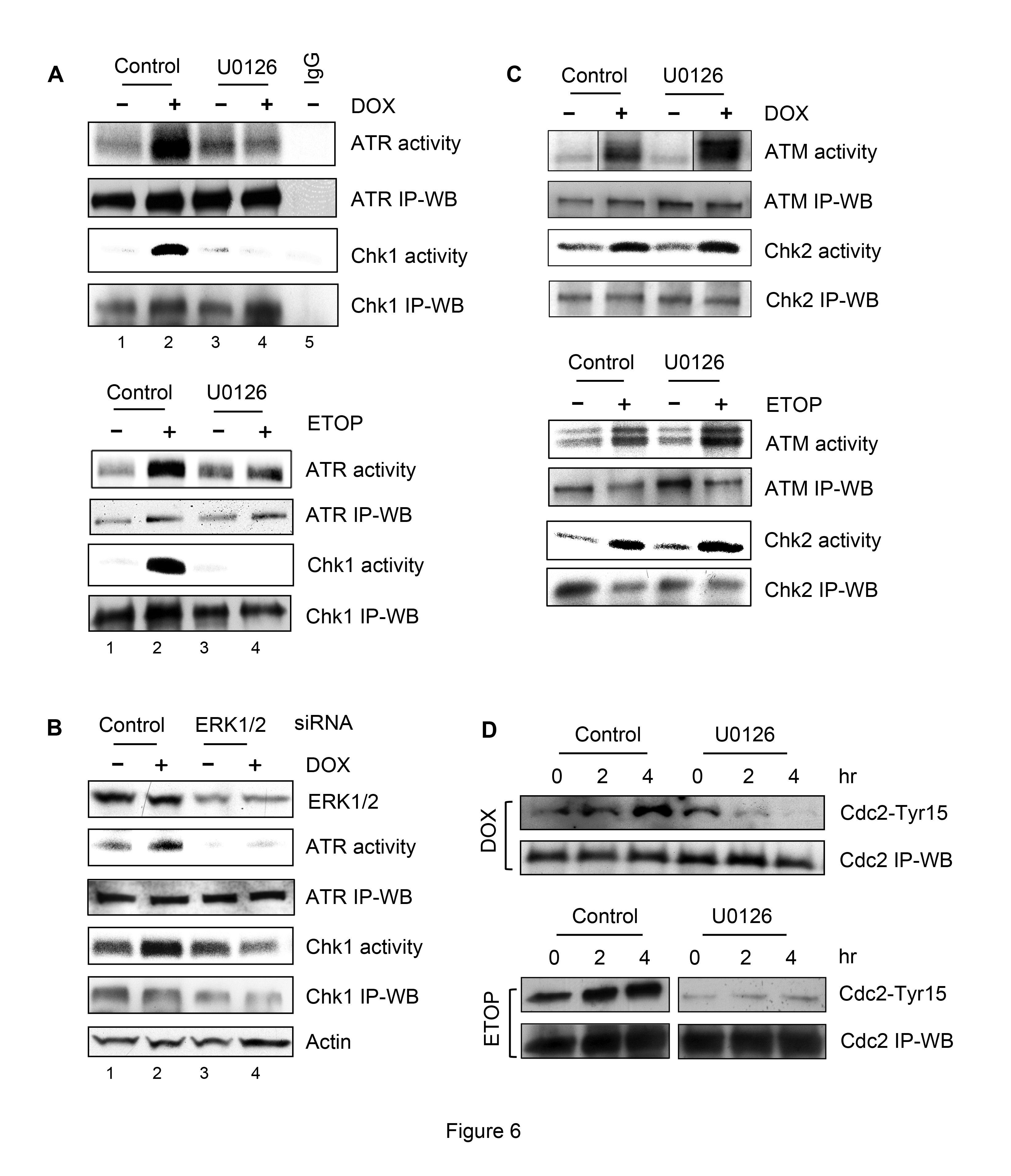

Supplement: S3 File — Note that the underlying blots for Fig 6A ETOP ATR activity, 6B ATR activity, and 6C DOX Chk2 activity are available only as cropped images. The underlying 6C ETOP ATM activity blots are from a shorter exposure than that used for the figure. (ZIP) [file pone.0292423.s003.zip › S3 File/Figure 6/Revised Figure 6-corrected.tif]

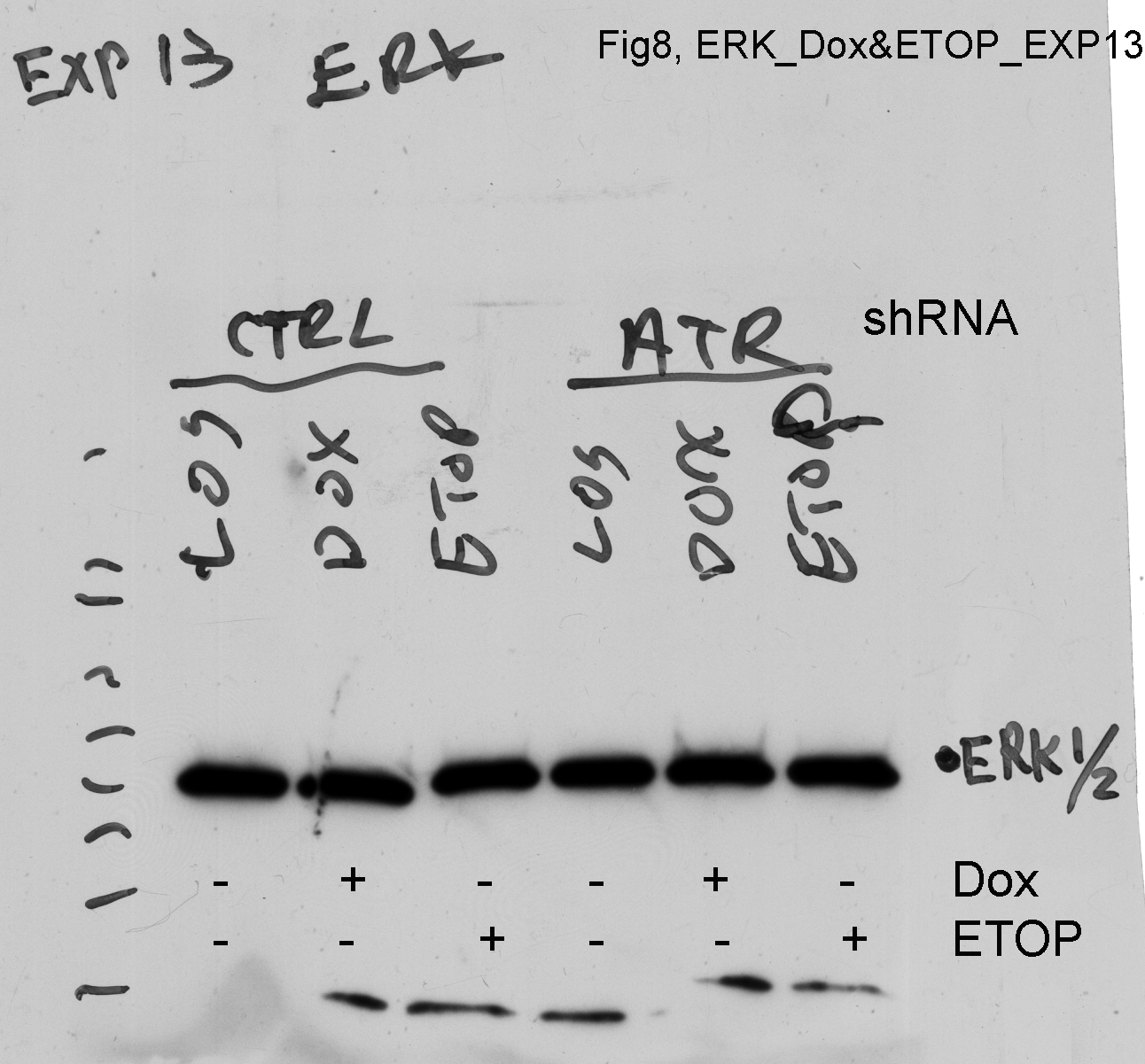

Supplement: S4 File — (ZIP) [file pone.0292423.s004.zip › Figure 8/Fig8, ERK_Dox&ETOP_EXP13.tif]

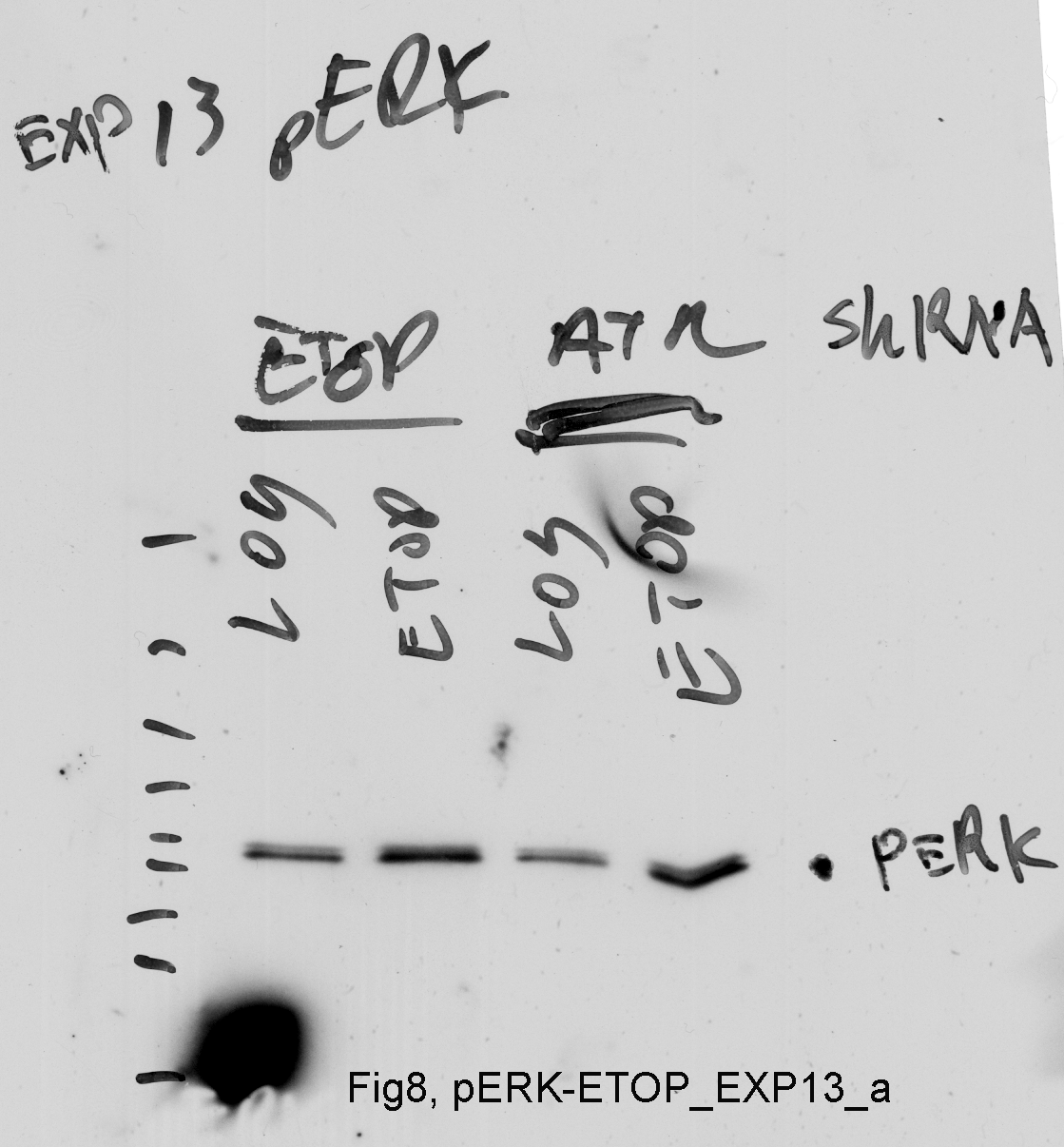

Supplement: S4 File — (ZIP) [file pone.0292423.s004.zip › Figure 8/Fig8, pERK-ETOP_EXP13_a.tif]

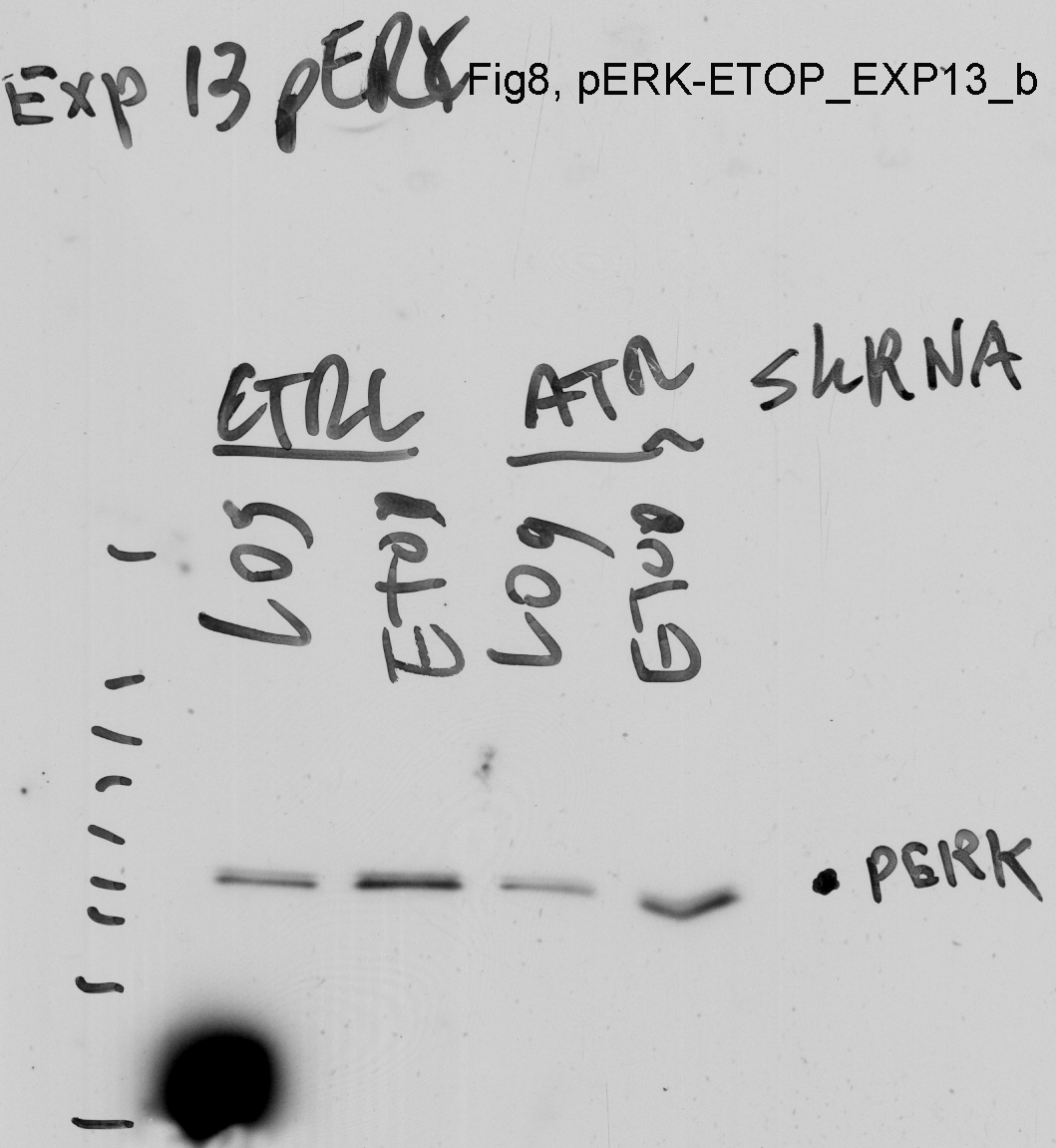

Supplement: S4 File — (ZIP) [file pone.0292423.s004.zip › Figure 8/Fig8, pERK-ETOP_EXP13_b.tif]

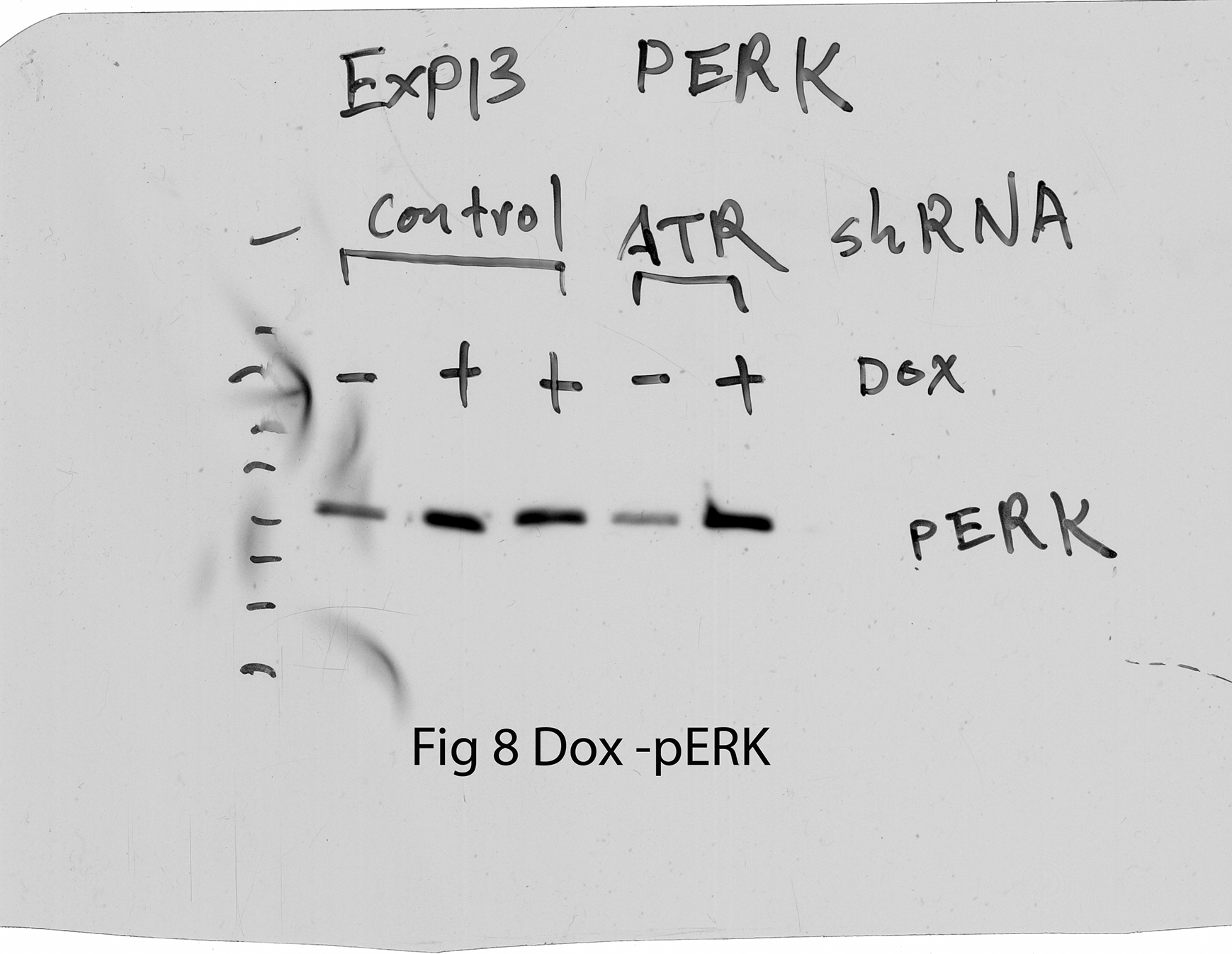

Supplement: S4 File — (ZIP) [file pone.0292423.s004.zip › Figure 8/Figure 8 DOX pERK.tif]

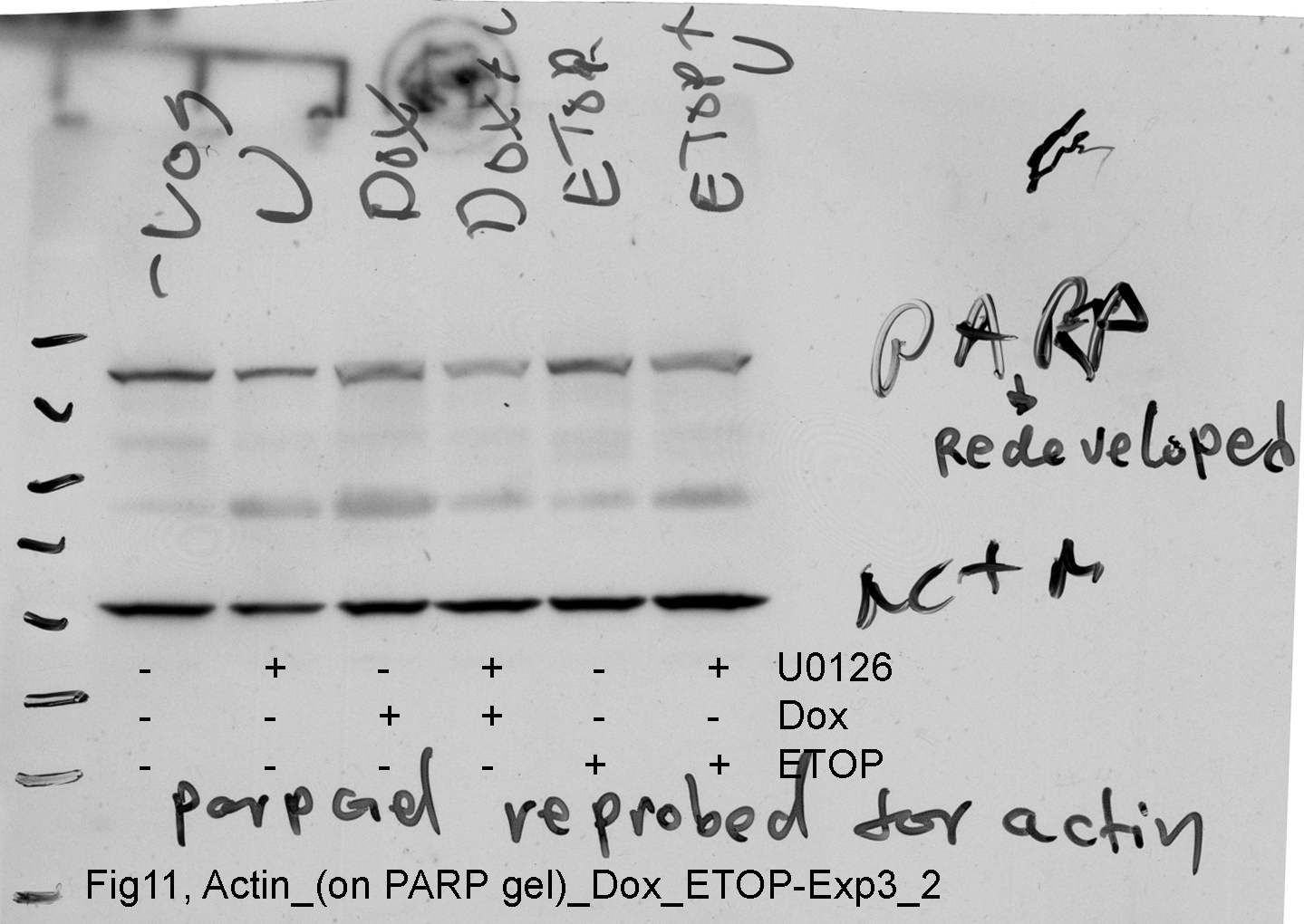

Supplement: S5 File — The underlying blot for the image shown in the original figure for the Control Caspase 8 and DOX Caspase 8 panels is not available; however, the authors provide an image of a shorter exposure of the same blot. Lanes 1 and 2 were used for the Control Caspase 8 panel, while lanes 1, 3, and 4 of the same blot were used for the DOX Caspase 8 panel. Additional Actin replicates are provided for the experiment shown in Fig 11B. One blot was probed first for the DOX PARP panel, and then re-probed for Actin. The blot showing PARP bands alone is not available, but the PARP bands remain visible on the blot after re-probing for Actin. (ZIP) [file pone.0292423.s005.zip › Figure 11/Fig11, published Actin_(on PARP gel)_Dox_ETOP-Exp3_2.tif]

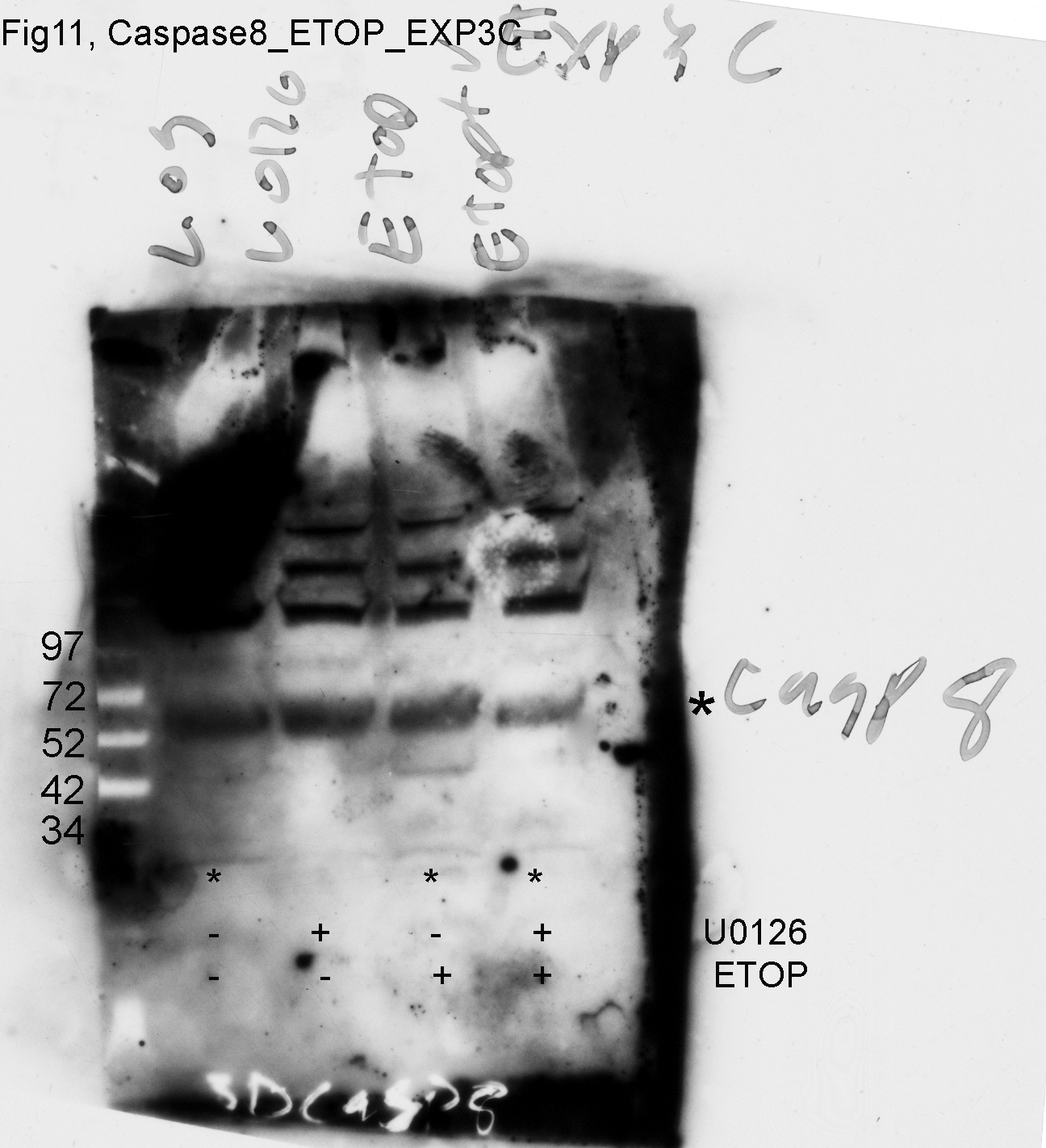

Supplement: S5 File — The underlying blot for the image shown in the original figure for the Control Caspase 8 and DOX Caspase 8 panels is not available; however, the authors provide an image of a shorter exposure of the same blot. Lanes 1 and 2 were used for the Control Caspase 8 panel, while lanes 1, 3, and 4 of the same blot were used for the DOX Caspase 8 panel. Additional Actin replicates are provided for the experiment shown in Fig 11B. One blot was probed first for the DOX PARP panel, and then re-probed for Actin. The blot showing PARP bands alone is not available, but the PARP bands remain visible on the blot after re-probing for Actin. (ZIP) [file pone.0292423.s005.zip › Figure 11/Fig11, published Caspase8_ETOP_EXP3C.tif]

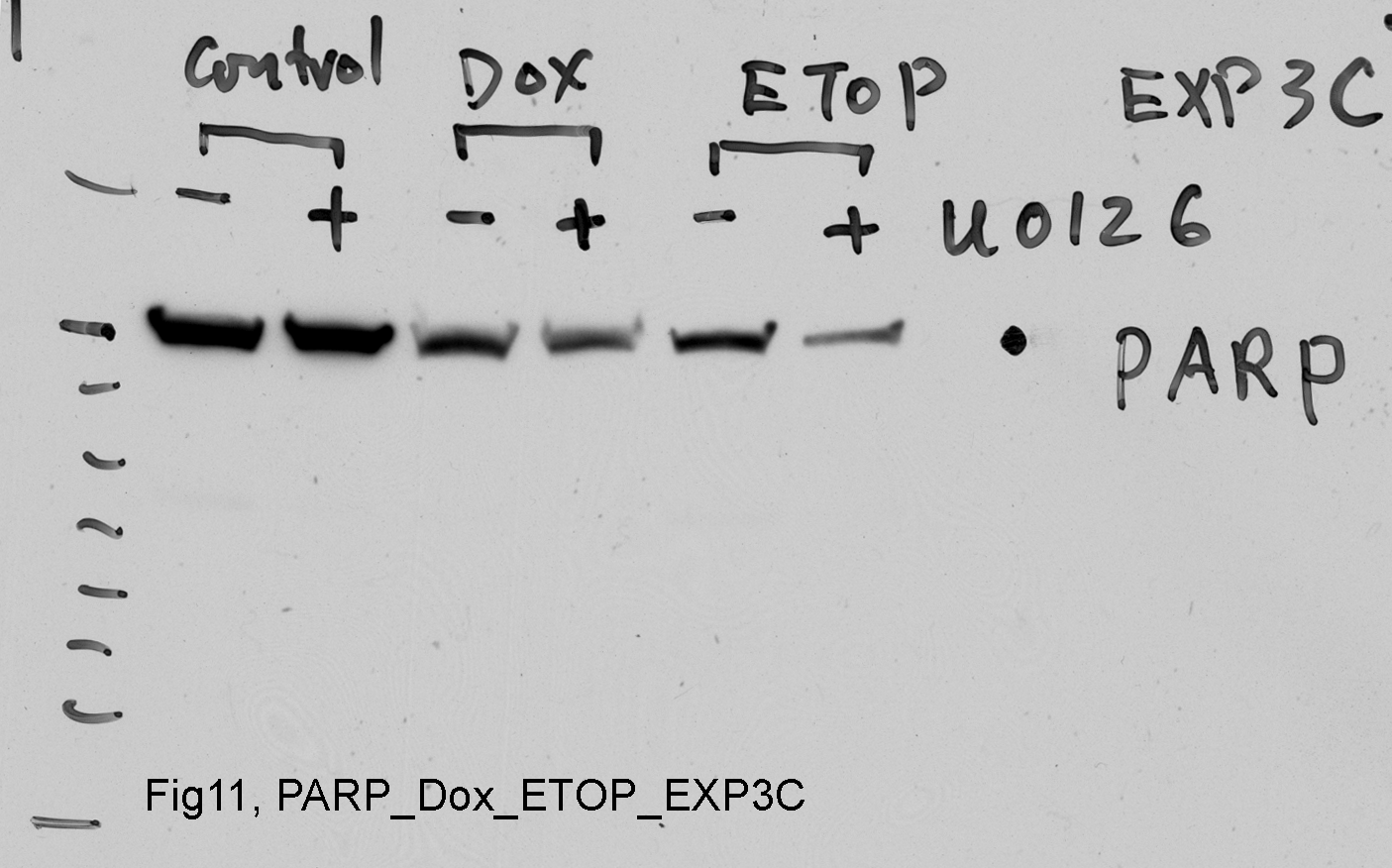

Supplement: S5 File — The underlying blot for the image shown in the original figure for the Control Caspase 8 and DOX Caspase 8 panels is not available; however, the authors provide an image of a shorter exposure of the same blot. Lanes 1 and 2 were used for the Control Caspase 8 panel, while lanes 1, 3, and 4 of the same blot were used for the DOX Caspase 8 panel. Additional Actin replicates are provided for the experiment shown in Fig 11B. One blot was probed first for the DOX PARP panel, and then re-probed for Actin. The blot showing PARP bands alone is not available, but the PARP bands remain visible on the blot after re-probing for Actin. (ZIP) [file pone.0292423.s005.zip › Figure 11/Fig11, published PARP_Dox_ETOP_EXP3C.tif]

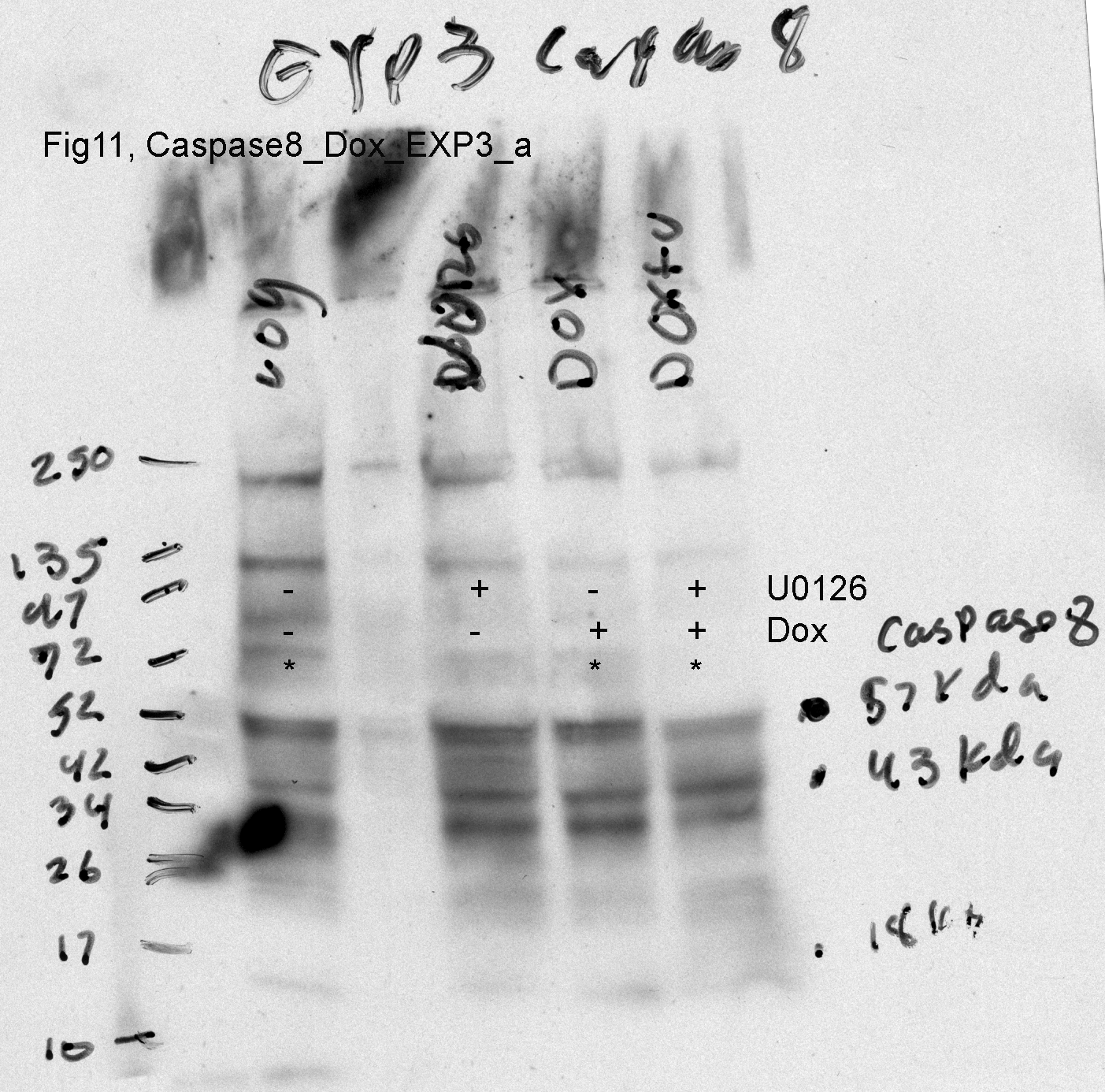

Supplement: S5 File — The underlying blot for the image shown in the original figure for the Control Caspase 8 and DOX Caspase 8 panels is not available; however, the authors provide an image of a shorter exposure of the same blot. Lanes 1 and 2 were used for the Control Caspase 8 panel, while lanes 1, 3, and 4 of the same blot were used for the DOX Caspase 8 panel. Additional Actin replicates are provided for the experiment shown in Fig 11B. One blot was probed first for the DOX PARP panel, and then re-probed for Actin. The blot showing PARP bands alone is not available, but the PARP bands remain visible on the blot after re-probing for Actin. (ZIP) [file pone.0292423.s005.zip › Figure 11/Fig11, shorter exposure published Caspase8_Dox_EXP3_a.tif]

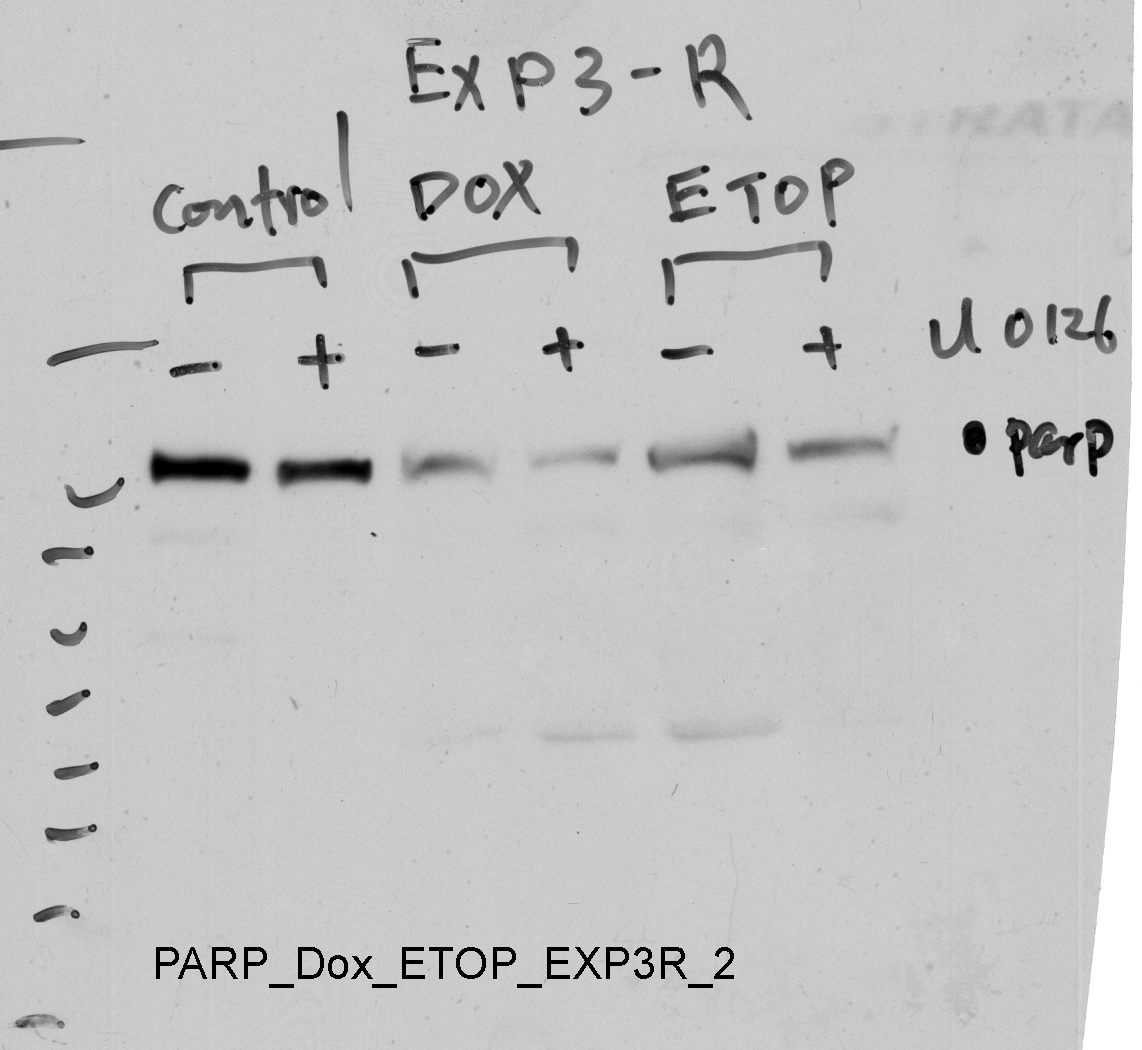

Supplement: S5 File — The underlying blot for the image shown in the original figure for the Control Caspase 8 and DOX Caspase 8 panels is not available; however, the authors provide an image of a shorter exposure of the same blot. Lanes 1 and 2 were used for the Control Caspase 8 panel, while lanes 1, 3, and 4 of the same blot were used for the DOX Caspase 8 panel. Additional Actin replicates are provided for the experiment shown in Fig 11B. One blot was probed first for the DOX PARP panel, and then re-probed for Actin. The blot showing PARP bands alone is not available, but the PARP bands remain visible on the blot after re-probing for Actin. (ZIP) [file pone.0292423.s005.zip › Figure 11/PARP_Dox_ETOP_EXP3R_2.tif]

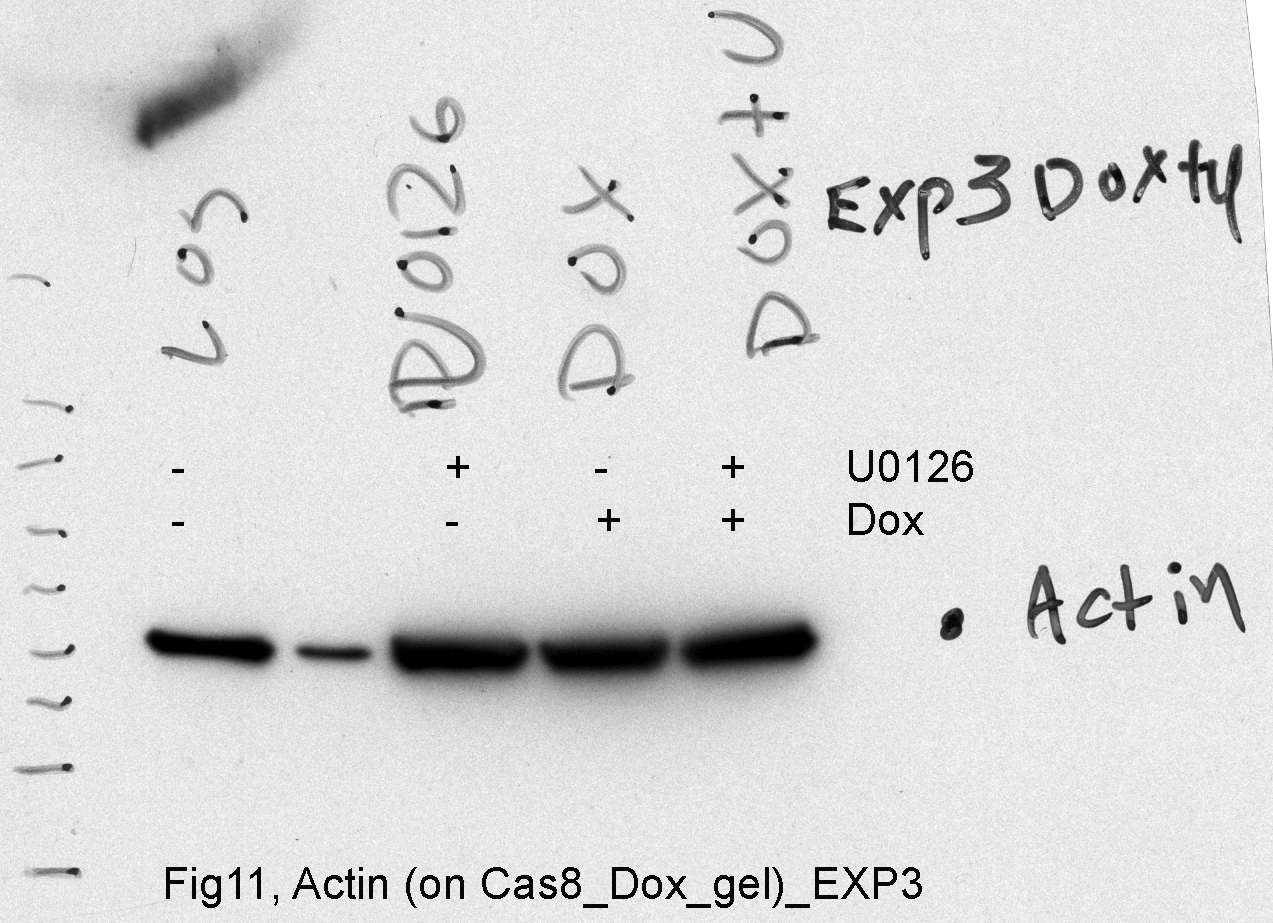

Supplement: S5 File — The underlying blot for the image shown in the original figure for the Control Caspase 8 and DOX Caspase 8 panels is not available; however, the authors provide an image of a shorter exposure of the same blot. Lanes 1 and 2 were used for the Control Caspase 8 panel, while lanes 1, 3, and 4 of the same blot were used for the DOX Caspase 8 panel. Additional Actin replicates are provided for the experiment shown in Fig 11B. One blot was probed first for the DOX PARP panel, and then re-probed for Actin. The blot showing PARP bands alone is not available, but the PARP bands remain visible on the blot after re-probing for Actin. (ZIP) [file pone.0292423.s005.zip › Figure 11/Replicate Fig11, Actin (on Cas8_Dox_gel)_EXP3.tif]

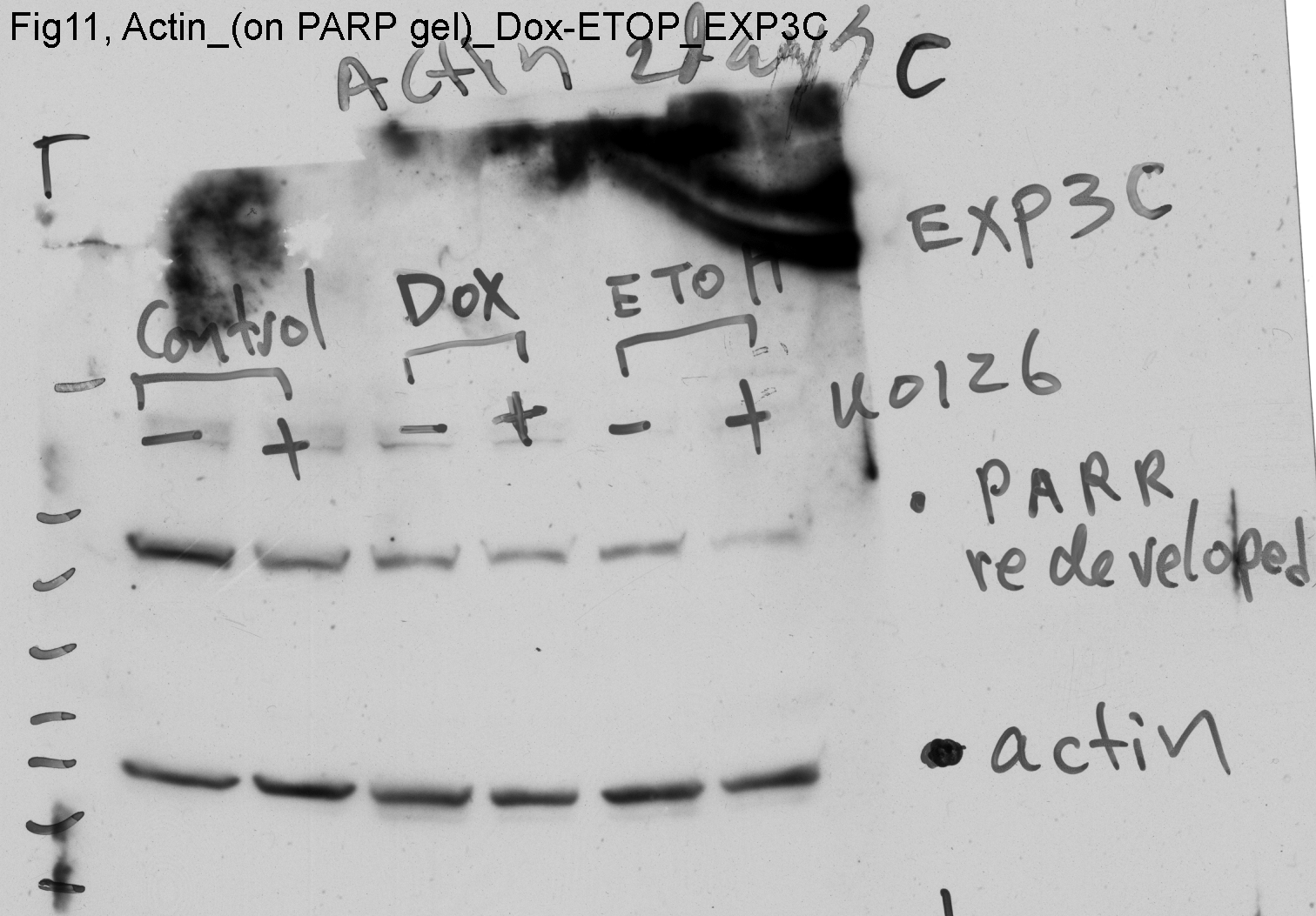

Supplement: S5 File — The underlying blot for the image shown in the original figure for the Control Caspase 8 and DOX Caspase 8 panels is not available; however, the authors provide an image of a shorter exposure of the same blot. Lanes 1 and 2 were used for the Control Caspase 8 panel, while lanes 1, 3, and 4 of the same blot were used for the DOX Caspase 8 panel. Additional Actin replicates are provided for the experiment shown in Fig 11B. One blot was probed first for the DOX PARP panel, and then re-probed for Actin. The blot showing PARP bands alone is not available, but the PARP bands remain visible on the blot after re-probing for Actin. (ZIP) [file pone.0292423.s005.zip › Figure 11/Replicate Fig11, Actin_(on PARP gel)_Dox-ETOP_EXP3C.tif]

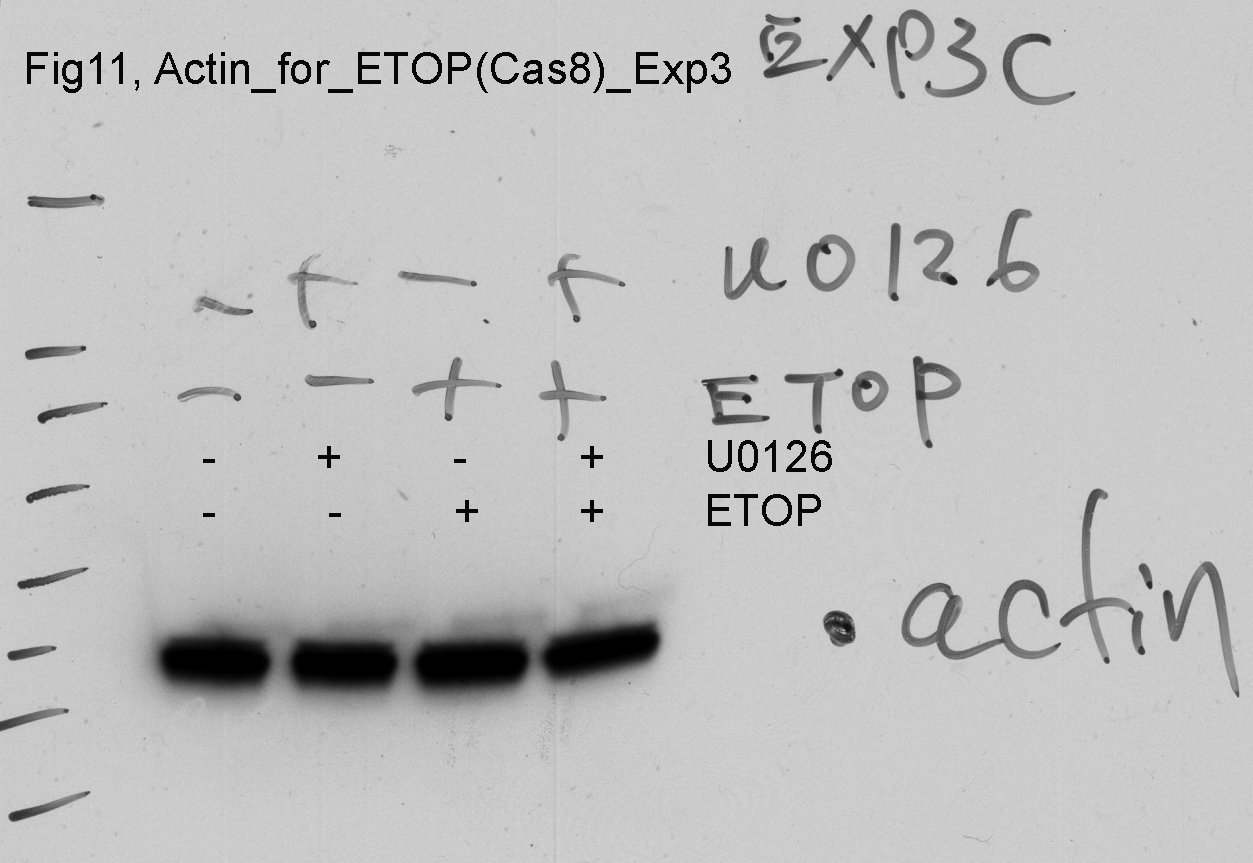

Supplement: S5 File — The underlying blot for the image shown in the original figure for the Control Caspase 8 and DOX Caspase 8 panels is not available; however, the authors provide an image of a shorter exposure of the same blot. Lanes 1 and 2 were used for the Control Caspase 8 panel, while lanes 1, 3, and 4 of the same blot were used for the DOX Caspase 8 panel. Additional Actin replicates are provided for the experiment shown in Fig 11B. One blot was probed first for the DOX PARP panel, and then re-probed for Actin. The blot showing PARP bands alone is not available, but the PARP bands remain visible on the blot after re-probing for Actin. (ZIP) [file pone.0292423.s005.zip › Figure 11/Replicate Fig11, Actin_for_ETOP(Cas8)_Exp3.tif]

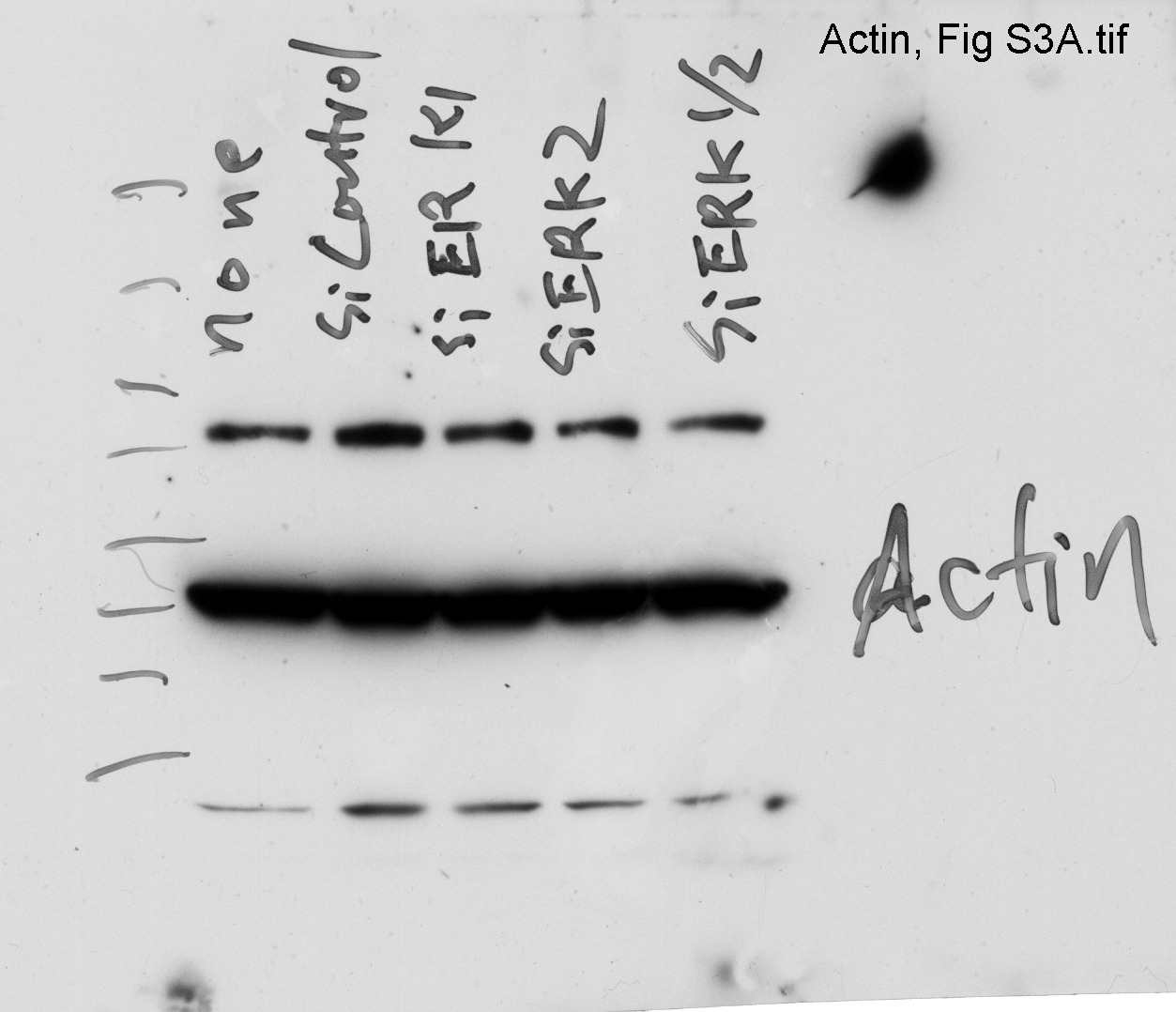

Supplement: S6 File — (ZIP) [file pone.0292423.s006.zip › Figure S3/Actin, Fig S3A.tif]

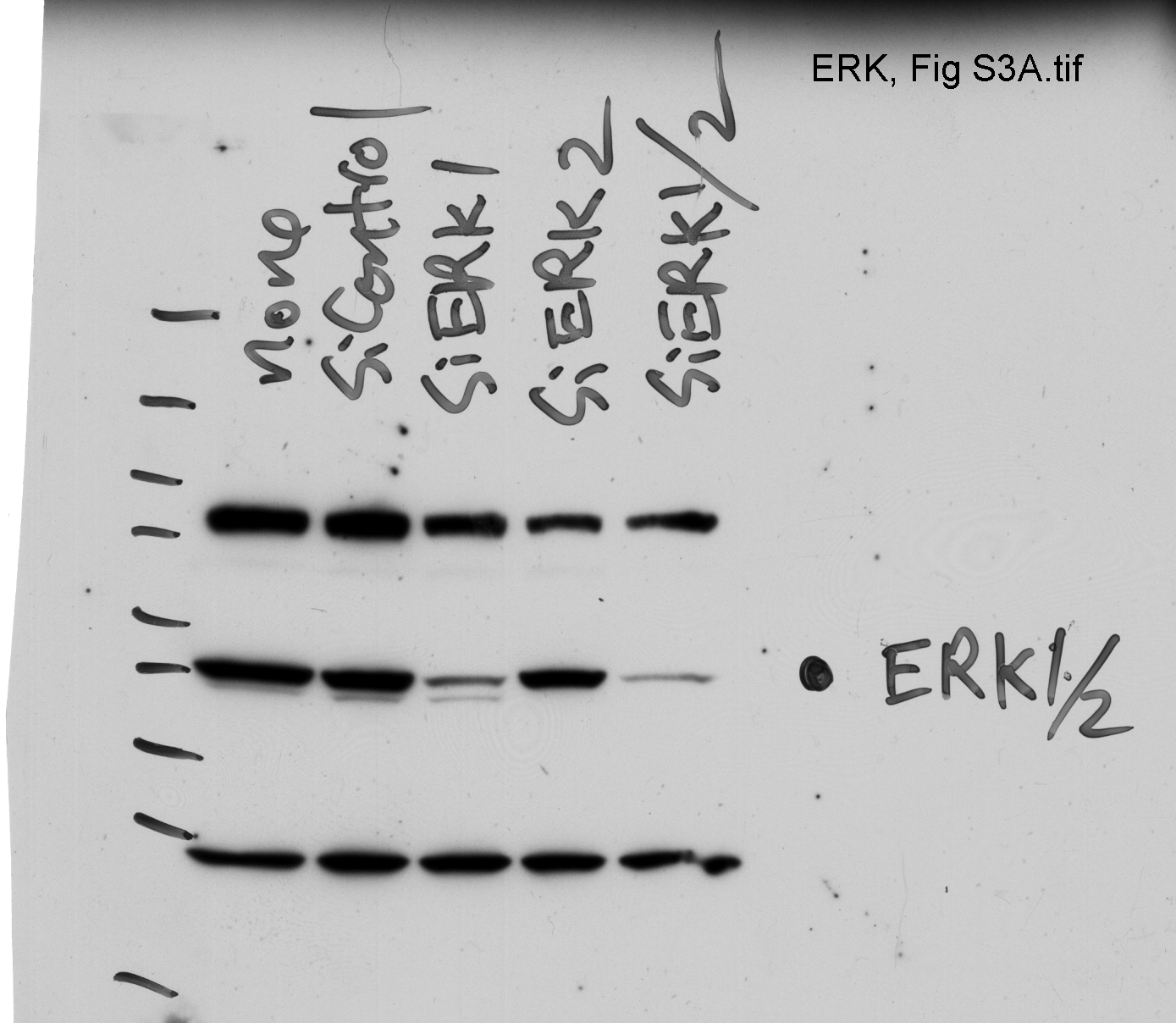

Supplement: S6 File — (ZIP) [file pone.0292423.s006.zip › Figure S3/ERK, Fig S3A.tif]

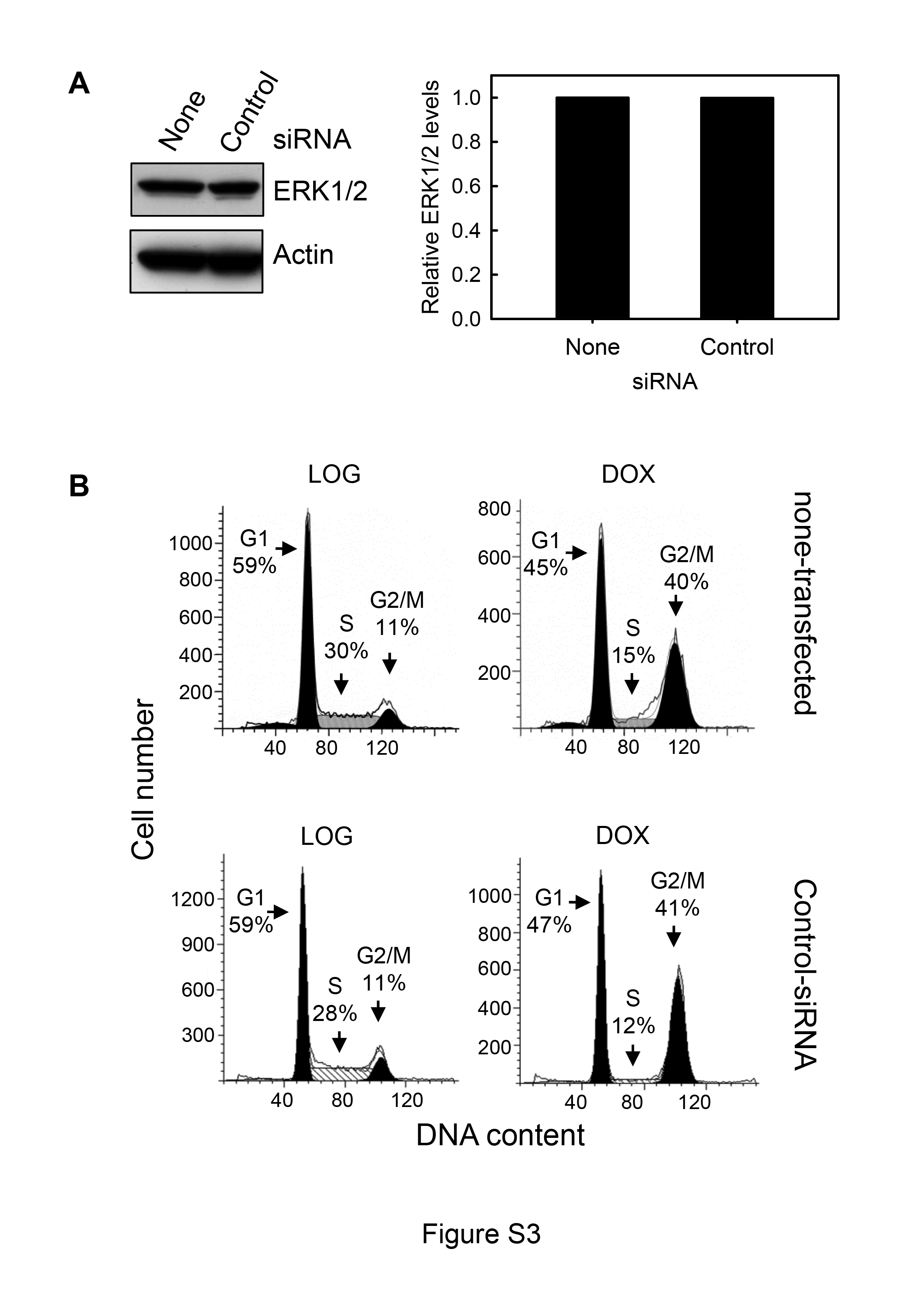

Supplement: S6 File — (ZIP) [file pone.0292423.s006.zip › Figure S3/Figure S3 Corrected.tif]
