# Supplementary material for: Correction: ERK1/2 Signaling Plays an Important Role in Topoisomerase II Poison-Induced G2/M Checkpoint Activation
Source: PLoS One. 2023 Sep 28;18(9):e0292423. doi: 10.1371/journal.pone.0292423 (PMC10538782; doi:10.1371/journal.pone.0292423)
Supplement: S2 File — Note that the underlying blot for Fig 2A ETOP Chk1 IP-WB may be a different exposure than that used for the figure. (ZIP) [file pone.0292423.s002.zip › S2 File/Figure Annotations.docx]

**Figure Annotations**

Figure 2B.

Blot: “fig 2B ETOP DOX-CDC2-f.tig”, “fig 2B ETOP DOX-pCDC2-F.tif”.

Lanes 2, 3 and 4 (0, 1 and 2 hr) were used for the ETOP (right) panel.

Lanes 5, 6 and 7 (0, 1 and 2 hr) were used for the DOX (left) panel.

Figure 6D.

Blot: DOX (upper) panel: “Fig 6D, DOX, CDC2-Y15.tif”, “Fig 6D, DOX, CDC2, IP-WB.tif”

ETOP (lower) panel: “Fig 6D, ETOP, Cdc2-Y15.tif”, “Fig 6D, ETOP, CDC2, IP-WB.tif”

Lanes 2-7 were used for DOX (upper) panel.

Lanes 1-3 and 5-7 were used for ETOP (lower) panel
